# Stereoselective synthesis of unnatural α-amino acid derivatives through photoredox catalysis†

**DOI:** 10.1039/d1sc00658d

**Published:** 2021-03-03

**Authors:** Andrey Shatskiy, Anton Axelsson, Elena V. Stepanova, Jian-Quan Liu, Azamat Z. Temerdashev, Bhushan P. Kore, Björn Blomkvist, James M. Gardner, Peter Dinér, Markus D. Kärkäs

**Affiliations:** Division of Organic Chemistry, Department of Chemistry, KTH Royal Institute of Technology SE-100 44 Stockholm Sweden karkas@kth.se; Tomsk Polytechnic University Lenin Avenue 30 634050 Tomsk Russia; Zelinsky Institute of Organic Chemistry of the Russian Academy of Sciences Leninsky Prospect 47 119991 Moscow Russia; Department of Analytical Chemistry, Kuban State University Stavropolskaya St. 149 350040 Krasnodar Russia; Division of Applied Physical Chemistry, Department of Chemistry, KTH Royal Institute of Technology SE-100 44 Stockholm Sweden

## Abstract

A protocol for stereoselective C-radical addition to a chiral glyoxylate-derived *N*-sulfinyl imine was developed through visible light-promoted photoredox catalysis, providing a convenient method for the synthesis of unnatural α-amino acids. The developed protocol allows the use of ubiquitous carboxylic acids as radical precursors without prior derivatization. The protocol utilizes near-stoichiometric amounts of the imine and the acid radical precursor in combination with a catalytic amount of an organic acridinium-based photocatalyst. Alternative mechanisms for the developed transformation are discussed and corroborated by experimental and computational studies.

## Abstract 2

A protocol for stereoselective C-radical addition to a chiral glyoxylate-derived *N*-sulfinyl imine was developed through visible light-promoted photoredox catalysis, providing a convenient method for the synthesis of unnatural α-amino acids.

## Graphical abstract

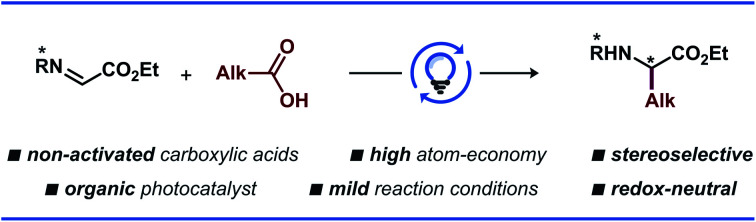

## Introduction

Unnatural α-amino acids constitute an important class of biologically relevant compounds that are widely used in both pharmaceutical industry and for fundamental research within molecular and structural biology.^1^ A number of pharmaceuticals based on unnatural α-amino acids are currently available, including angiotensin-converting enzyme (ACE) inhibitors for the treatment of cardiovascular and renal diseases,^2^ antiviral medicines,^3^ and others.^4^ Recently, peptidomimetic α-ketoamide inhibitors based on unnatural α-amino acids have received increased attention as drug candidates for treatment of COVID-19 disease caused by the SARS-CoV-2 coronavirus,^5^ highlighting the high demand for such building blocks.

**Fig. 1:**
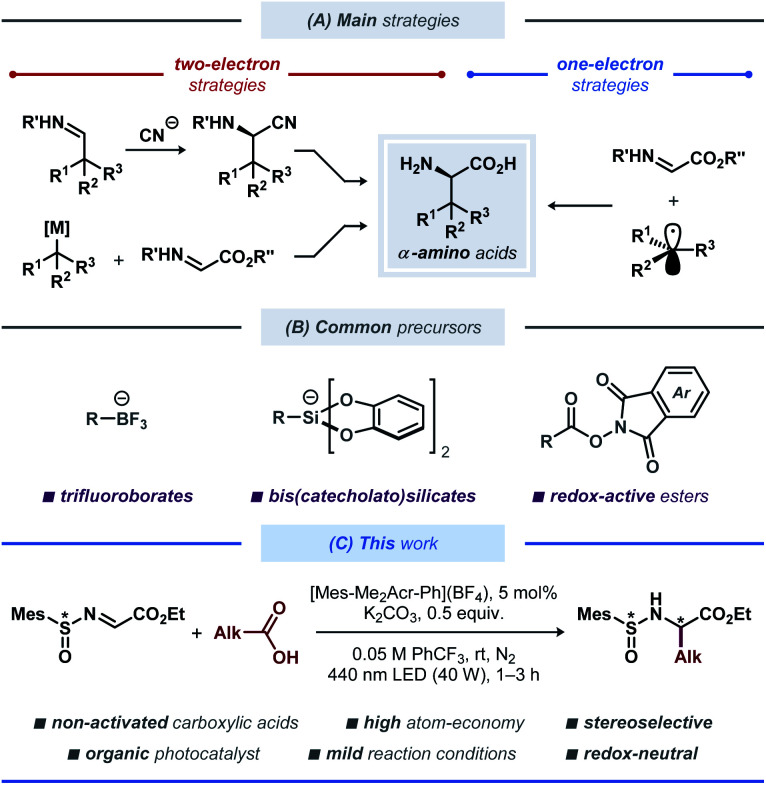
(A) Selected two- and one-electron strategies for synthesis of unnatural α-amino acids. (B) Common precursors for radical addition to imines employed in photoredox catalytic systems. (C) Diastereoselective decarboxylative alkylation of *N*-sulfinyl imines with non-activated carboxylic acids.

A variety of synthetic strategies to access unnatural amino acid derivatives have been developed over the years, with some notable methods being the catalytic asymmetric Strecker-type reactions, asymmetric hydrogenation of dehydroamino acids, and electrophilic and nucleophilic alkylation of glycine derivatives (Fig. 1A).^6^ Among these, functionalization or reduction of α-imino esters offers a straightforward route to various enantiomerically enriched α-amino acids.^7^ Traditionally, these strategies have employed polar retrosynthetic disconnections, which often require the use of (super)stoichiometric amounts of toxic and highly sensitive reagents at low temperatures, thereby limiting the substrate scope and practicality for scale up of these reactions. These limitations have recently been challenged by re-introduction of free-radical reaction manifolds, aided by the developments in base-metal catalysis,^8^ electrosynthesis^9^ and photoredox catalysis,^10^ leading to a vast array of strategies for light-induced modification and synthesis of amino acids and peptides.^11^ Among these, radical addition to imines through photoredox catalysis was demonstrated in symmetric^12^ and asymmetric^13^ fashion (Fig. 1B). In 2017, Alemán and co-workers reported a protocol for asymmetric radical addition to imines mediated by visible light.^13*a*^ The developed catalytic system made use of a chiral sulfoxide auxiliary group, commonly employed in the synthesis of chiral amines.^14^ Here, the C-centered radical was generated through visible light-mediated reductive cleavage of the N–O bond in a redox-active phthalimide ester, followed by radical addition to the *N*-sulfinyl imine. The reductive nature of this protocol required the use of a stoichiometric amount of a reducing agent (Hanztsch ester). More recently, a related Ni-based catalytic system was described by Baran and co-workers.^15^ This protocol employed a tetrachloro-substituted redox-active ester as the radical precursor, with Zn as a stoichiometric reducing agent and a Ni-based catalyst for mediating the C–C bond formation. Although this protocol displayed an impressive substrate scope, it is associated with moderate atom-economy, limiting its applicability for large-scale synthesis.

## Results and discussion

Inspired by the catalytic systems developed by the Alemán^13*a*^ and Baran^15^ groups, we sought to realize a protocol for diastereoselective decarboxylative radical addition to chiral *N*-sulfinyl imines that would utilize ubiquitous non-activated carboxylic acids as radical precursors.^16^ A related direct decarboxylative addition process was attempted by the Alemán group for a benzaldehyde-derived *N*-sulfinyl imine under reaction conditions reported by MacMillan;^17^ however, no formation of the desired product was observed (see the ESI to ref. 13*a*). Similarly, we observed no desired product with pivalic acid **2a** as the radical precursor and *N*-sulfinyl imine **1** as the radical acceptor when the reaction was conducted in DMSO with [Ir(dF(CF_3_)ppy)_2_(dtbbpy)](PF_6_) as photocatalyst (Table 1, entry 1), presumably due to fast decomposition of *N*-sulfinyl imine **1**. Gratifyingly, changing the solvent to α,α,α-trifluorotoluene (PhCF_3_) furnished the desired product **3a** in fairly good yield of 65%, although with poor diastereoselectivity (Table 1, entry 2). Using other bases in place of Cs_2_CO_3_ completely prohibited the reaction (for details on the optimization studies, see the ESI†), and the highly-oxidizing photocatalyst 4CzIPN^18^ failed to deliver the radical addition product (Table 1, entry 3). Fortunately, the highly-oxidizing organic acridinium-based photocatalyst [Mes-Acr-Me](BF_4_) delivered product **3a** with excellent diastereoselectivity, although in poor yield (Table 1, entry 4). Increasing the catalyst loading from 1 to 5 mol% and switching to the more stable *N*-phenyl-substituted photocatalysts [Mes-Acr-Ph](BF_4_) and [Mes-Me_2_Acr-Ph](BF_4_)^19^ dramatically increased the yield of the stereoselective radical addition product up to 78% (Table 1, entries 5–7). Conducting the reaction in more conventional solvents, such as MeCN, CH_2_Cl_2_, and 2,2,2-trifluoroethanol (TFE) in place of PhCF_3_ resulted in diminished yields (Table S1, see the ESI†), highlighting the documented inertness of PhCF_3_ towards free-radical intermediates.^20^ Changing the base to K_2_CO_3_ and increasing the base loading further improved the yield up to 85% (Table 1, entry 11). Finally, utilizing a slight excess of the acid radical precursor **2a** delivered the desired product **3a** in excellent yields (91% and 95% for 1.2 and 1.5 equiv. of **2a**, respectively; Table 1, entries 13 and 14). Consistently with the previous reports on radical additions to *N*-sulfinyl imines, the *tert*-butyl- and *para*-tolyl-substituted *N*-sulfinyl imines **4** and **5** proved to be inefficient as radical acceptors (Table 1, entries 15 and 16).^13*a*,15^ In case of *tert*-butyl-substituted *N*-sulfinyl imine **4**, it is likely that the transiently formed aminyl radical intermediate underwent decomposition to form an iminosulfanone (–N=S=O), thereby disrupting the catalytic cycle.^21^

**Table 1:** Optimization of the reaction conditions for the decarboxylative radical addition to a glyoxylate-derived *N*-sulfinyl imine^a^

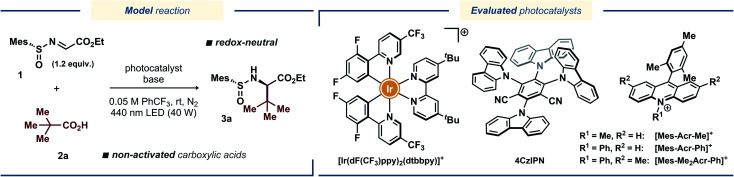					
Entry	Photocatalyst	Base	Time	Yield^b^	dr^b^
1^c^	[Ir(dF(CF_3_)ppy)_2_(dtbbpy)](PF_6_), 1 mol%	Cs_2_CO_3_, 0.2 equiv.	20 min	—	—
2	[Ir(dF(CF_3_)ppy)_2_(dtbbpy)](PF_6_), 1 mol%	Cs_2_CO_3_, 0.2 equiv.	20 min	65%	4 : 1
3	4CzIPN, 1 mol%	Cs_2_CO_3_, 0.2 equiv.	20 min	—	—
4	[Mes-Acr-Me](BF_4_), 1 mol%	Cs_2_CO_3_, 0.2 equiv.	20 min	27%	>95 : 5
5	[Mes-Acr-Me](BF_4_), 5 mol%	Cs_2_CO_3_, 0.2 equiv.	20 min	48%	>95 : 5
			60 min	66%	>95 : 5
6	[Mes-Acr-Ph](BF_4_), 5 mol%	Cs_2_CO_3_, 0.2 equiv.	60 min	73%	>95 : 5
7	[Mes-Me_2_Acr-Ph](BF_4_), 5 mol%	Cs_2_CO_3_, 0.2 equiv.	60 min	78%	>95 : 5
8	[Mes-Me_2_Acr-Ph](BF_4_), 5 mol%	K_3_PO_4_, 0.2 equiv.	60 min	80%	>95 : 5
9	[Mes-Me_2_Acr-Ph](BF_4_), 5 mol%	K_2_CO_3_, 0.2 equiv.	60 min	84%	>95 : 5
10	[Mes-Me_2_Acr-Ph](BF_4_), 5 mol%	K_2_CO_3_, 0.05 equiv.	20 min	<5%	—
11	[Mes-Me_2_Acr-Ph](BF_4_), 5 mol%	K_2_CO_3_, 0.5 equiv.	60 min	85%	>95 : 5
12^d^	[Mes-Me_2_Acr-Ph](BF_4_), 5 mol%	K_2_CO_3_, 0.5 equiv.	60 min	77%	>95 : 5
**13** e	**[Mes-Me**_**2**_ **Acr-Ph](BF**_**4**_ **), 5 mol%**	**K**_**2**_ **CO**_**3**_ **, 0.5 equiv.**	**60 min**	**91%**	**>95 : 5**
14^f^	[Mes-Me_2_Acr-Ph](BF_4_), 5 mol%	K_2_CO_3_, 0.5 equiv.	60 min	95%	>95 : 5
15	As entry 13, but with ^*t*^Bu-sulfinyl imine **4**		60 min	—	—
16	As entry 13, but with *p*-Tol-sulfinyl imine **5**		60 min	50%	7 : 1

**Deviations from the conditions in entry 13**					
17	Under air		60 min	12%	>95 : 5
18	No photocatalyst		60 min	—	—
19	No light		60 min	—	—

a The reactions were performed on 0.1 mmol scale: stock solutions of pivalic acid **2** and the photocatalyst (each in 1 mL of the solvent) were mixed with *N*-sulfinyl imine **1** and the base under anhydrous conditions, and stirred under irradiation with 440 nm blue LED light at room temperature (for details, see the ESI).

b Determined by ^1^H NMR of crude reaction mixture with 1,3,5-trimethoxybenzene as an internal standard.

c 0.1 M DMSO.

d 0.1 M PhCF_3_.

e 1.0 equiv. of *N*-sulfinyl imine **1** and 1.2 equiv. of pivalic acid **2**.

f 1.0 equiv. of *N*-sulfinyl imine **1** and 1.5 equiv. of pivalic acid **2**.

**Fig. 2:**
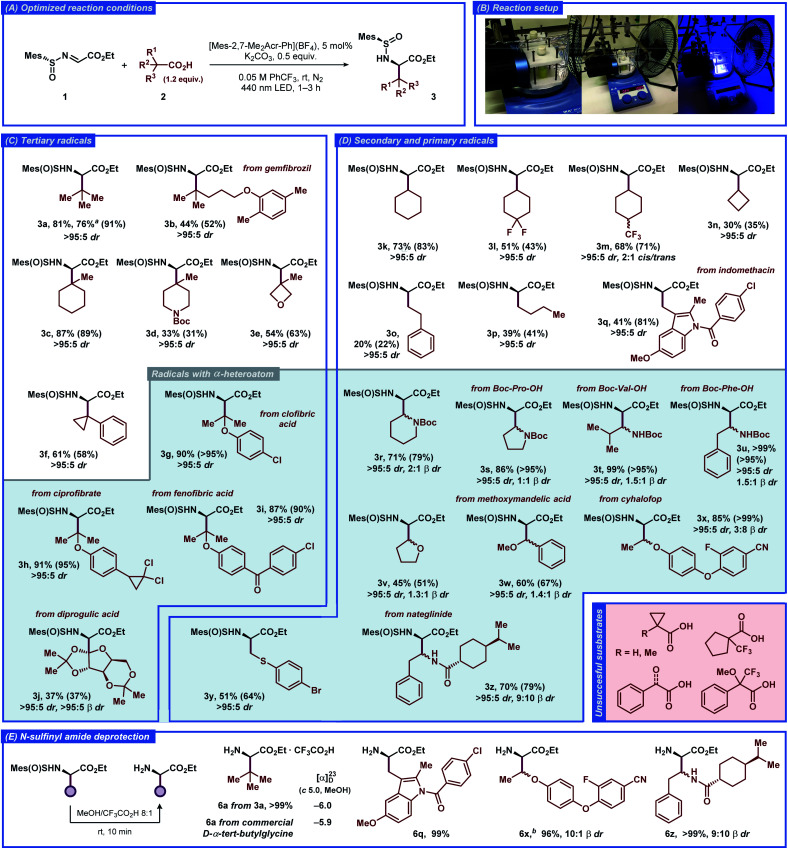
Substrate scope for the decarboxylative radical addition to glyoxylate-derived *N*-sulfinyl imine **1** to furnish the unnatural α-amino acid derivatives **3**. The isolated yields of the products and NMR-yields from the crude reaction mixtures (in parenthesis) are reported (for details, see the ESI†). ^*a*^1 mmol scale reaction. ^*b*^Synthesized from the **3x-2** β-diastereomer of **3x** (see the ESI†).

The substrate scope of the developed transformation was evaluated with a variety of non-functionalized and functionalized tertiary, secondary, and primary carboxylic acids (Fig. 2). For all of the amino acid derivatives excellent diastereoselectivity at the α-position was observed (>95 : 5 dr). The radical precursors producing tertiary and secondary alkyl-substituted radicals provided the expected products in generally good to high yields (**3a–e** and **3k–n**). The highly reactive primary alkyl radicals displayed lower selectivity for the addition reaction (**3o** and **3p**), consistent with previous reports featuring unstable free-radical intermediates under related conditions.^22^ Further optimization of the reaction conditions for the primary acids **2o** and **2p** did not result in improved yields (Tables S2 and S3†), illustrating the intrinsic instability of the respective radical intermediates and/or the photocatalyst under the employed conditions. Benzylic-type radicals were generally inefficient (see Fig. 2, unsuccessful substrates); however, a cyclopropyl-substituted benzylic radical and an indole-derived benzylic-type radical provided the expected products **3f** and **3q**, respectively, in satisfactory yields.

The carboxylic acid radical precursors that furnish stabilized α-heteroatom C-radicals generally provided the addition products in good to excellent yields. Gratifyingly, *N*-Boc-protected α-amino acid radical precursors based on pipecolic acid, proline, valine, and phenylalanine furnished the expected amino acid derivatives **3r–u** in generally excellent yields, exemplifying a prominent synthetic route to biologically active α,β-diamino acids.^23^ The α-*O*-substituted radicals derived from dialkyl (**3v**, **3w**) and alkyl aryl ethers (**3g–i**, **3x**) provided the expected products in moderate and excellent yields, respectively. To our delight, a primary α-*S*-substituted radical containing an aryl bromide functionality afforded the expected product **3y** in satisfactory yield despite combining several structural features that can be deleterious under free-radical conditions. The sterically-demanding carbohydrate-based radical derived from diprogulic acid **2j** delivered the monosaccharide-amino acid conjugate product **3j** in satisfactory yield and excellent diastereoselectivity at both the α- and β-stereocenters (>95 : 5 α dr, >95 : 5 β dr).

The developed transformation was successfully applied for late-stage derivatization of a number of complex biologically-active compounds, including gemfibrozil (**2b**), ciprofibrate (**2h**), indomethacin (**2q**), cyhalofop (**2x**), nateglinide (**2z**), and clofibric (**2g**), diprogulic (**2j**) and fenofibric acids (**2i**). Besides the aforementioned functional groups, the reaction tolerated alkyl and aryl chloro- (**3g–i**, **3q**) and fluorosubstituted substrates (**3l**, **3m**, **3x**), diaryl ether (**3x**), diaryl ketone (**3i**), chiral carboxamide (**3z**), and aryl cyanide (**3x**) functionalities, demonstrating the high versatility of the developed approach for stereoselective synthesis of unnatural α-amino acids. Notably, some of the products also displayed slight diastereoselectivity at the β-position, up to *ca.* 2 : 1 β dr for products **3r** and **3x**. Isolation of a single α,β-diastereomer could be achieved for several products with conventional chromatography (**3j**, **3m**, **3n**, **3r**, **3x**). In general, the acids with strong electron-withdrawing (CF_3_, carbonyl) or aromatic (phenyl) substituents at the α-position did not provide the desired products, likely due to the insufficient nucleophilic character of the formed C-radicals (see Fig. 2, unsuccessful substrates). The α-H- and α-methyl-substituted cyclopropanecarboxylic acids were also inefficient, despite successful reaction with the analogous α-phenyl-substituted substrate **2f**.

The *N*-sulfinyl amide functionality in product **3a** could be conveniently removed under mild acidic conditions in quantitative yield (Fig. 2E). The absolute configuration at the α-stereocenter in the obtained α-amino ester was then determined as (*R*), in complete agreement with the previous observations and the results of computational studies (for details, see the ESI†). Similarly, removal of the *N*-sulfinyl amide functionality was carried out for more complex products **3q**, **3x** and **3z**, and the corresponding α-amino esters **6q**, **6x** and **6z** were isolated in excellent yields (>95%).

**Fig. 3:**
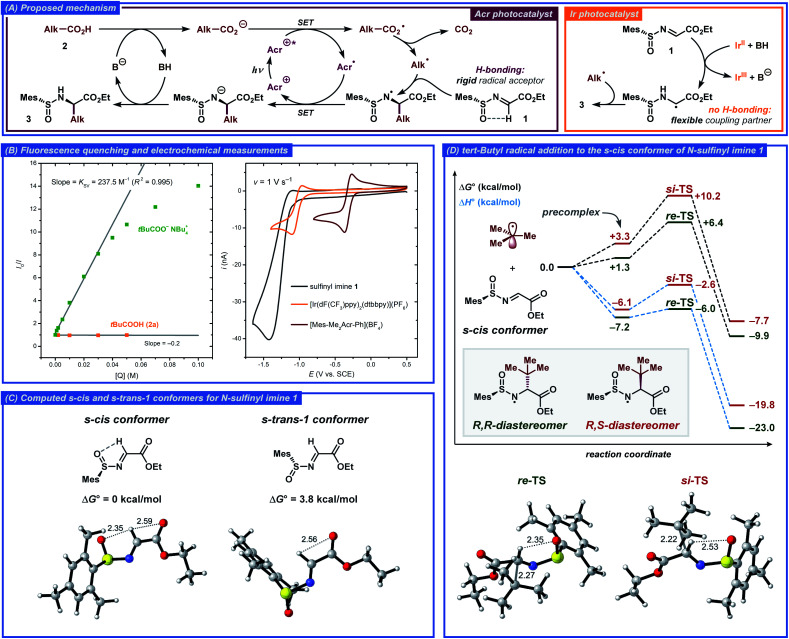
(A) Outline of the proposed mechanisms with the acridinium- (*left*) and Ir-based (*right*) photocatalysts. (B) Steady-state fluorescent quenching and electrochemical measurements. (C) Calculated structures with selected bond distances (Å) for the *s*-*cis* and *s*-*trans*-1 conformers of *N*-sulfinyl imine **1**. (D) Gibbs free energy and enthalpy diagrams for *tert*-butyl radical addition to **1**, and calculated structures for the *re*-TS and *si*-TS transition states.

Based on literature precedents, a mechanism for the developed transformation was proposed and corroborated by fluorescence quenching and computational studies (Fig. 3).^13*a*,24^ Initially, the acridinium photocatalyst [Mes-Me_2_Acr-Ph]^+^ (Acr^+^) is excited by visible light (*λ*_max_ ≈ 425 nm) to a highly oxidizing excited state Acr^+^* (*E*(Acr^+^*/Acr˙) ≈ 2.09 V *vs.* SCE).^25^ In this state, the photocatalyst can abstract an electron from the deprotonated carboxylic acid *via* a single-electron transfer (SET) event to generate a carboxylate radical while being reduced to the acridinium radical Acr˙. The steady-state and time-resolved fluorescence quenching measurements for tetrabutylammonium pivalate as the model radical precursor demonstrated efficient quenching of the excited acridinium photocatalyst with Stern–Volmer quenching constant *K*_SV_ = 237.5 M^−1^ and bimolecular quenching constant *k*_q_ = 6.8 × 10^9^ M^−1^ s^−1^, while no quenching was observed for the free pivalic acid (Fig. 3B, S3 and S4†). The carboxyl radical formed *via* SET then extrudes CO_2_ to yield a C-centered radical, which undergoes addition to the *N*-sulfinyl imine **1** in the key step of the reaction, forming an α-alkylated N-centered radical. Finally, the N-centered radical is reduced by acridinium radical Acr˙, closing the photocatalytic cycle and furnishing the desired product **3** upon protonation (Fig. 3A).

In order to gain better understanding of the stereodetermining C–C bond forming step in the proposed mechanism, DFT calculations were performed on the M062X-D3/6-311+G(d,p) level of theory (for details, see the ESI†). First, the structure of the *N*-sulfinyl imine radical acceptor **1** was evaluated. Previously, Alemán and co-workers tentatively suggested an *s*-*cis* conformation around the N–S bond as being more stable in such compounds due to the hydrogen bonding between the imine proton and the sulfoxide oxygen.^13*a*^ Such a conformational preference would then lead to the α-(*R*) product when the S(*R*)-sulfinyl imine is employed as the radical acceptor. This stereochemical outcome was indeed observed for both Alemán's and our catalytic system. The calculations confirmed that the *s*-*cis* conformer is more stable compared to the *s*-*trans*-1 conformer by 3.8 kcal mol^−1^, corresponding to >99.8 : 0.2 ratio between the conformers from the Boltzmann distribution at room temperature (Fig. 3C). In the *s-cis* conformer, the hydrogen bonding between the imine hydrogen and the sulfoxide oxygen could be observed from the non-covalent interaction (NCI) plots, while no hydrogen bonding was present in the *s*-*trans*-1 conformer (for a detailed discussion, see the ESI†).

Subsequently, the radical addition step was evaluated for the *tert*-butyl radical donor and the *N*-sulfinyl imine radical acceptor **1**. The computed Gibbs free energy and enthalpy diagrams for the reaction are presented in Fig. 3D. The formation of the (*R*,*R*)-diastereomer of **3a** was found to be favored both kinetically and thermodynamically and the computed activation barrier was found to be 3.8 kcal mol^−1^ smaller for the *re*-addition compared to the *si*-addition, while the (*R*,*R*)-diastereomer product is 2.5 kcal mol^−1^ more stable compared to the (*R*,*S*)-diastereomer. Interestingly, the difference in the computed activation barriers for the *re*- and *si*-addition reactions originated almost exclusively from the enthalpic terms (ΔΔ*G*^‡^ = 3.8 kcal mol^−1^, ΔΔ*H*^‡^ = 3.4 kcal mol^−1^). The better stabilization of the *re*-TS is in part due to the stronger hydrogen bonding between the imine hydrogen and the sulfoxide oxygen for this transition state, as evident from the calculated bond distances and the NCI plots (Fig. 3D and S7†). Additionally, significant steric crowding occurs in the *si*-TS, where the incoming *tert*-butyl radical requires the mesityl group to become almost completely coplanar to the sulfoxide S=O bond. In contrast, the mesityl group and the S=O bond in the *re*-TS are out of plane by 50° while the incoming *tert*-butyl radical experiences no steric crowding.

An alternative mechanism for a related radical addition to imine derivatives was proposed by Ooi and co-workers.^26^ In this mechanism, the key C–C bond-forming step was found to proceed through radical–radical coupling between a C-centered radical and an α-amino radical formed by one-electron one-proton reduction of an imine substrate. However, under our conditions such a mechanistic pathway seems unlikely due to weak reducing ability of the one-electron reduced form of the employed acridinium photocatalyst (*E*_1/2_(Acr^+^/Acr˙) ≈ −0.32 V *vs.* SCE, Fig. 3B). As opposed to the conditions reported by Ooi and co-workers, where strongly reducing [Ir(ppy)_2_(bpy)]^+^-type photocatalysts (*E*(Ir^III^/Ir^II^) ≈ −1.5 V *vs.* SCE) were used, electron transfer from Acr˙ to *N*-sulfinyl imine **1** (*E*_p/2_ ≈ −1.24 V *vs.* SCE, Fig. 3B) should not be favored. However, a contribution from the radical–radical coupling pathway would explain the low diastereoselectivity (4 : 1 dr) during formation of product **3a** when the reaction was conducted with the [Ir(dF(CF_3_)ppy)_2_(dtbbpy)](PF_6_) photocatalyst (Table 1, entry 2). Indeed, this photocatalyst displayed relatively low reduction potential (*E*_1/2_(Ir^III^/Ir^II^) = −1.10 V *vs.* SCE, Fig. 3B), sufficient to reduce the *N*-sulfinyl imine substrate **1** to the corresponding α-amino radical. The conformation analysis of this radical then revealed nearly free rotation around the N–S bond with a barrier of *ca.* 2.5 kcal mol^−1^, while *N*-sulfinyl imine **1** displayed a significantly higher rotation barrier of *ca.* 8.0 kcal mol^−1^ and only one dominant conformer. Addition of the *tert*-butyl radical to the α-amino radical would therefore be expected to proceed with low, if any, diastereoselectivity. The low diastereoselectivity could also be explained by product epimerization during the reaction; however, no epimerization was observed when product **3a** was subjected to the comparable reaction conditions with the Ir-based photocatalyst.

## Conclusions

In conclusion, a practical protocol for stereoselective synthesis of various α-amino acids has been developed, employing ubiquitous carboxylic acids as radical precursors and an organic photocatalyst under visible light irradiation. This protocol allows for synthesis of highly functionalized α-amino acids, which are challenging to prepare through traditional two-electron reaction manifolds. The protocol utilizes near-stoichiometric amounts of reagents and does not produce large quantities of waste, which is an intrinsic disadvantage of the previously described systems utilizing redox-active esters as radical precursors.

## Author contributions

A. S. performed optimization studies, major part of substrate scope investigation, electrochemical and spectroscopic studies, and wrote the manuscript. A. A. performed the computational studies and part of the substrate scope investigation. E. V. S., J.-Q. L., and B. B. performed part of the substrate scope investigation. A. Z. T. performed part of the analytic measurements. B. P. K. and J. M. G. assisted during data acquisition and analysis of the spectroscopical studies. M. D. K. conceived and directed the project. P. D. and M. D. K. supervised the project. All authors discussed the results and approved the final version of the manuscript.

## Conflicts of interest

There are no conflicts to declare.

## Supplementary Material

SC-012-D1SC00658D-s001
