# Supplementary material for: Stereoselective synthesis of unnatural α-amino acid derivatives through photoredox catalysis
Source: Chem Sci. 2021 Mar 3;12(15):5430–7. doi: 10.1039/d1sc00658d (PMC8179686; doi:10.1039/d1sc00658d)
Supplement: SC-012-D1SC00658D-s001 [file SC-012-D1SC00658D-s001.pdf]

Electronic Supplementary Material (ESI) for Chemical Science.
This journal is © The Royal Society of Chemistry 2021

Supplementary Information

Stereoselective Synthesis of Unnatural α-Amino Acid Derivatives through Photoredox Catalysis

Andrey Shatskiy,[a] *Anton Axelsson,*[a] *Elena V. Stepanova,*[b,c] *Jian-Quan Liu,*[a] *Azamat Z. Temerdashev,*[d] *Bhushan P. Kore,*[e] *Björn Blomkvist,*[a] *James M. Gardner,*[e] *Peter Dinér*[a] *and Markus D. Kärkäs***[a]

[a] Department of Chemistry, Division of Organic Chemistry, KTH Royal Institute of Technology, SE-100 44 Stockholm, Sweden

[b] Tomsk Polytechnic University, Lenin Avenue 30, 634050 Tomsk, Russia

[c] Zelinsky Institute of Organic Chemistry of the Russian Academy of Sciences, Leninsky Prospect 47, 119991 Moscow, Russia

[d] Department of Analytical Chemistry, Kuban State University, Stavropolskaya St. 149, 350040 Krasnodar, Russia

[e] Department of Chemistry, Division of Applied Physical Chemistry, KTH Royal Institute of Technology, SE-100 44 Stockholm, Sweden

E-mail: karkas@kth.se

Table of Contents

1. *General methods* S7

2. *General procedure for the synthesis of N-sulfinyl imines* S8

3. *Optimization of the reaction conditions* S8

4. *General procedure for the decarboxylative radical addition to N-sulfinyl imine **1*** S11

5. *Procedure for the 1 mmol scale synthesis of compound **3a*** S12

6. *N-sulfinyl amide deprotection and determination of the absolute configuration for product **3a*** S13

7. *Electrochemical and fluorescence quenching studies* S14

8. *Computational studies* S17

8.1 Conformation analysis for *N*-sulfinyl imine **1** and the corresponding α-amino radical S17

8.2 Addition of the tert-butyl radical to *N*-sulfinyl imine **1** S19

8.3 Evaluation of transition states from ***s-trans*-1** and ***s-trans*-2** conformers S20

8.4 Evaluation of different functionals S21

8.5 Evaluation of the solvent effect S21

9. *Analytical data* S22

Compound **1** S22

Compound **4** S22

Compound **5** S22

Compound **3a** S23

Compound **6a** S23

Compound **3b** S24

Compound **3c** S24

Compound **3d** S25

Compound **3e** S25

Compound **3f** S26

Compound **3g** S26

Compound **3h** S27

Compound **3i** S27

Compound **3j** S28

Compound **3k** S28

Compound **3l** S29

Compound **3m** S29

Compound **3n** S31

Compound **3o** ... S31
Compound **3p** ... S32
Compound **3q** ... S32
Compound **6q** ... S33
Compound **3r** ... S33
Compound **3s** ... S34
Compound **3t** ... S35
Compound **3u** ... S35
Compound **3v** ... S36
Compound **3w** ... S37
Compound **3x** ... S38
Compound **6x** ... S39
Compound **3y** ... S39
Compound **3z** ... S40
Compound **6z** ... S40
10. *NMR spectra* ... S42
^{1}H NMR (500 MHz, $CDCl_3$) of compound **1** ... S42
^{13}C NMR (126 MHz, $CDCl_3$) of compound **1** ... S43
^{1}H NMR (400 MHz, $CDCl_3$) of compound **4** ... S44
^{13}C NMR (101 MHz, $CDCl_3$) of compound **4** ... S45
^{1}H NMR (400 MHz, $CDCl_3$) of compound **5** ... S46
^{13}C NMR (101 MHz, $CDCl_3$) of compound **5** ... S47
^{1}H NMR (500 MHz, $CDCl_3$) of compound **3a** ... S48
^{13}C NMR (126 MHz, $CDCl_3$) of compound **3a** ... S49
^{1}H NMR (500 MHz, MeOH-d_4) of compound **6a** ... S50
^{13}C NMR (126 MHz, MeOH-d_4) of compound **6a** ... S51
^{19}F NMR (377 MHz, MeOH-d_4) of compound **6a** ... S52
^{1}H NMR (500 MHz, $CDCl_3$) of compound **3b** ... S53
^{13}C NMR (126 MHz, $CDCl_3$) of compound **3b** ... S54
^{1}H NMR (500 MHz, $CDCl_3$) of compound **3c** ... S55
^{13}C NMR (126 MHz, $CDCl_3$) of compound **3c** ... S56
^{1}H NMR (500 MHz, $CDCl_3$) of compound **3d** ... S57
^{13}C NMR (126 MHz, $CDCl_3$) of compound **3d** ... S58

^{1}H NMR (500 MHz, $CDCl_3$) of compound **3e**........ S59
^{13}C NMR (126 MHz, $CDCl_3$) of compound **3e**........ S60
^{1}H NMR (500 MHz, $CDCl_3$) of compound **3f**........ S61
^{1}H NMR (126 MHz, $CDCl_3$) of compound **3f**........ S62
^{1}H NMR (500 MHz, $CDCl_3$) of compound **3g**........ S63
^{13}C NMR (126 MHz, $CDCl_3$) of compound **3g**........ S64
^{1}H NMR (500 MHz, $CDCl_3$) of compound **3h**........ S65
^{13}C NMR (126 MHz, $CDCl_3$) of compound **3h**........ S66
^{1}H NMR (500 MHz, $CDCl_3$) of compound **3i**........ S67
^{13}C NMR (126 MHz, $CDCl_3$) of compound **3i**........ S68
^{1}H NMR (500 MHz, $CDCl_3$) of compound **3j**........ S69
^{13}C NMR (126 MHz, $CDCl_3$) of compound **3j**........ S70
^{1}H NMR (500 MHz, $CDCl_3$) of compound **3k**........ S71
^{13}C NMR (126 MHz, $CDCl_3$) of compound **3k**........ S72
^{1}H NMR (500 MHz, $CDCl_3$) of compound **3l**........ S73
^{13}C NMR (126 MHz, $CDCl_3$) of compound **3l**........ S74
^{19}F NMR (377 MHz, $CDCl_3$) of compound **3l**........ S75
^{1}H NMR (500 MHz, $CDCl_3$) of compound **3m** (*cis/trans*-diastereomer **3m-1**)........ S76
^{13}C NMR (126 MHz, $CDCl_3$) of compound **3m** (*cis/trans*-diastereomer **3m-1**)........ S77
^{19}F NMR (377 MHz, $CDCl_3$) of compound **3m** (*cis/trans*-diastereomer **3m-1**)........ S78
^{1}H NMR (500 MHz, $CDCl_3$) of compound **3m** (*cis/trans*-diastereomers **3m-1**/**3m-2** 3:5)........ S79
^{13}C NMR (126 MHz, $CDCl_3$) of compound **3m** (*cis/trans*-diastereomers **3m-1**/**3m-2** 3:5)........ S80
^{19}F NMR (377 MHz, $CDCl_3$) of compound **3m** (*cis/trans*-diastereomers **3m-1**/**3m-2** 3:5)........ S81
^{1}H NMR (500 MHz, $CDCl_3$) of compound **3n**........ S82
^{13}C NMR (126 MHz, $CDCl_3$) of compound **3n**........ S83
^{1}H NMR (500 MHz, $CDCl_3$) of compound **3o**........ S84
^{13}C NMR (126 MHz, $CDCl_3$) of compound **3o**........ S85
^{1}H NMR (500 MHz, $CDCl_3$) of compound **3p**........ S86
^{13}C NMR (126 MHz, $CDCl_3$) of compound **3p**........ S87
^{1}H NMR (500 MHz, $CDCl_3$) of compound **3q**........ S88
^{13}C NMR (126 MHz, $CDCl_3$) of compound **3q**........ S89
^{1}H NMR (500 MHz, $CDCl_3$) of compound **6q**........ S90
^{13}C NMR (126 MHz, $CDCl_3$) of compound **6q**........ S91

^{1}H NMR (500 MHz, $CDCl_3$) of compound **3r** (β-diastereomer **3r-1**) S92
^{13}C NMR (126 MHz, $CDCl_3$) of compound **3r** (β-diastereomer **3r-1**) S93
^{1}H NMR (500 MHz, $CDCl_3$) of compound **3r** (β-diastereomer **3r-2**) S94
^{13}C NMR (126 MHz, $CDCl_3$) of compound **3r** (β-diastereomer **3r-2**) S95
^{1}H NMR (500 MHz, $CDCl_3$) of compound **3s** S96
^{13}C NMR (126 MHz, $CDCl_3$) of compound **3s** S97
^{1}H NMR (500 MHz, $CDCl_3$) of compound **3t** S98
^{13}C NMR (126 MHz, $CDCl_3$) of compound **3t** S99
^{1}H NMR (500 MHz, $CDCl_3$) of compound **3u** (β-diastereomer **3u-1**) S100
^{13}C NMR (126 MHz, $CDCl_3$) of compound **3u** (β-diastereomer **3u-1**) S101
^{1}H NMR (500 MHz, $CDCl_3$) of compound **3u** (β-diastereomers **3u-1**/**3u-2** 1:1) S102
^{13}C NMR (126 MHz, $CDCl_3$) of compound **3u** (β-diastereomers **3u-1**/**3u-2** 1:1) S103
^{1}H NMR (500 MHz, $CDCl_3$) of compound **3v** S104
^{13}C NMR (126 MHz, $CDCl_3$) of compound **3v** S105
^{1}H NMR (500 MHz, $CDCl_3$) of compound **3w** S106
^{13}C NMR (126 MHz, $CDCl_3$) of compound **3w** S107
^{1}H NMR (500 MHz, $CDCl_3$) of compound **3x** (β-diastereomer **3x-1**) S108
^{13}C NMR (126 MHz, $CDCl_3$) of compound **3x** (β-diastereomer **3x-1**) S109
^{19}F NMR (377 MHz, $CDCl_3$) of compound **3x** (β-diastereomer **3x-1**) S110
^{1}H NMR (500 MHz, $CDCl_3$) of compound **3x** (β-diastereomer **3x-2**) S111
^{13}C NMR (126 MHz, $CDCl_3$) of compound **3x** (β-diastereomer **3x-2**) S112
^{19}F NMR (377 MHz, $CDCl_3$) of compound **3x** (β-diastereomer **3x-2**) S113
^{1}H NMR (500 MHz, $CDCl_3$) of compound **6x** S114
^{13}C NMR (126 MHz, $CDCl_3$) of compound **6x** S115
^{19}F NMR (377 MHz, $CDCl_3$) of compound **6x** S116
^{1}H NMR (500 MHz, $CDCl_3$) of compound **3y** S117
^{13}C NMR (126 MHz, $CDCl_3$) of compound **3y** S118
^{1}H NMR (500 MHz, $CDCl_3$) of compound **3z** S119
^{13}C NMR (126 MHz, $CDCl_3$) of compound **3z** S120
^{1}H NMR (500 MHz, $CDCl_3$) of compound **6z** S121
^{13}C NMR (126 MHz, $CDCl_3$) of compound **3z** S122
11. *Cartesian coordinates and energies* S123
tert-butyl radical S123

s-cis conformer of *N*-sulfinyl imine **1** S124
***s-trans*-1** conformer of *N*-sulfinyl imine **1** S125
***s-trans*-2** conformer of N-sulfinyl imine **1** S126
s-cis conformer of *N*-sulfinyl imidoyl fluoride S128
s-trans conformer of *N*-sulfinyl imidoyl fluoride S129
re-precomplex for ***s-cis*** conformer of *N*-sulfinyl imine **1** S131
si-precomplex for ***s-cis*** conformer of *N*-sulfinyl imine **1** S133
re*-TS** for ***s-cis conformer of *N*-sulfinyl imine **1** S134
si*-TS** for ***s-cis conformer of *N*-sulfinyl imine **1** S136
***si*-TS** for ***s-trans*-1** conformer of *N*-sulfinyl imine **1** S138
***re*-TS** for ***s-trans*-1** conformer of *N*-sulfinyl imine **1** S140
***si*-TS** for ***s-trans*-2** conformer of *N*-sulfinyl imine **1** S142
***re*-TS** for ***s-trans*-2** conformer of *N*-sulfinyl imine **1** S144
R,R-radical adduct from *tert*-butyl radical and *N*-sulfinyl imine **1** S145
R,S-radical adduct from *tert*-butyl radical and *N*-sulfinyl imine **1** S147
tert-butyl radical, SMD(CH_2Cl_2) S149
s-cis conformer of *N*-sulfinyl imine **1**, SMD(CH_2Cl_2) S150
re*-TS** for ***s-cis conformer of *N*-sulfinyl imine **1**, SMD(CH_2Cl_2) S151
si*-TS** for ***s-cis conformer of *N*-sulfinyl imine **1**, SMD(CH_2Cl_2) S153
12. *References* S155

1. *General methods*

Commercial anhydrous α,α,α-trifluorotoluene ($PhCF_3$, Sigma-Aldrich, Art. No. 547948-100ML) was used without further purification. When needed, CH_2Cl_2, MeCN, and Et_3N were dried by refluxing over CaH_2 for several hours followed by distillation, and the dried solvents were used immediately after preparation. 2,2,2-Trifluoroethanol (TFE) and PhF were dried over activated 3 Å molecular sieves. The 4CzIPN photocatalyst[1] and tetrabutylammonium dibutylphosphate ($Bu_4N(BuO)_2PO_2$)[2] were synthesized according to the previously published procedures.

NMR spectra were recorded in $CDCl_3$ or MeOH-*d*$_4$ on Bruker Avance DMX 500 MHz or Bruker Ascend 400 MHz NMR spectrometers and internally calibrated against the residual undeuterated solvent peaks ($CHCl_3$: δ 7.26 for ^1H NMR and δ 77.16 for ^{13}C NMR; CHD_2OD: δ 3.31 for ^1H NMR and δ 49.00 for ^{13}C NMR). The chemical shifts are reported in ppm and the peak multiplicities are designated as: s (singlet), d (doublet), t (triplet), q (quadruplet), dd (doublet of doublets), dt (doublet of triplets), ddd (doublet of doublets of doublets), td (triplet of doublets), tt (triplet of triplets), qd (quadruplet of doublets), m (multiplet). HRMS measurements were performed on methanolic solutions of the synthesized compounds with Bruker micrOTOF spectrometer (direct injection, electrospray ionization, ESI).

The radical addition reactions were carried out in 4 mL vials (VWR, Screw neck vials N13, Art. No. MANA702962) equipped with a stirring bar (VWR, Art. No. 442-0401) and a septum (VWR, Art. No. 217-0183), unless otherwise noted. The reaction vials were placed in a holder (distance between the reaction vial and the lamp is ca. 2 cm, Figure S1) and illuminated with 440 nm LED (40 W, Kessil PR160, set to maximum intensity) with continuous stirring at 1000 rpm. The isolated radical addition products were purified by column chromatography (15–25 cm height, 2 cm inner diameter) with silica gel (high-purity grade, 60 Å, 130–270 mesh, Sigma-Aldrich, Art. No. 288608-1KG) or by preparative TLC (PLC Silica gel 60 F_{254}, 1 mm, 20 x 20 cm, Merck, Art. No. 1.13895).

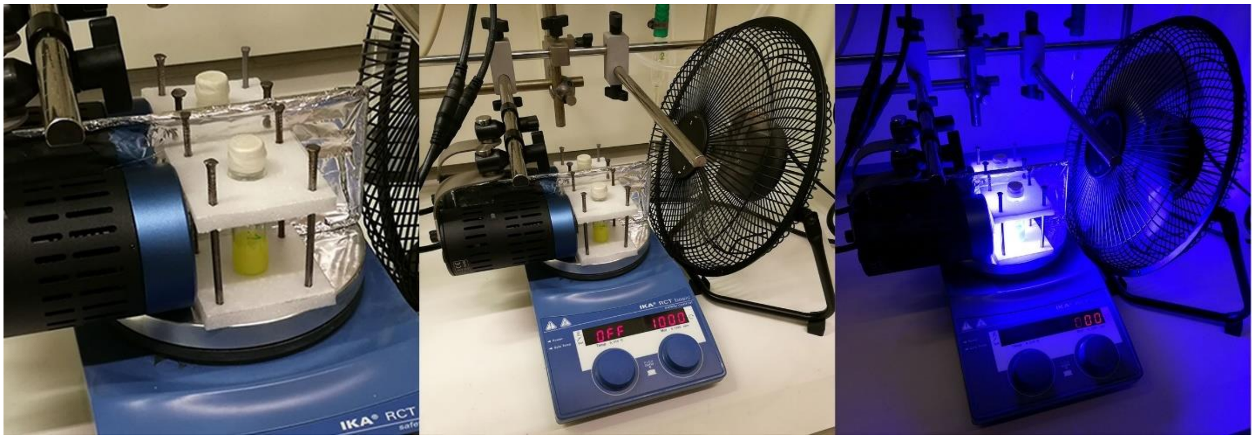

Figure S1. The reaction setup for optimization of the reaction conditions and substrate scope evaluation.

2. *General procedure for the synthesis of N-sulfinyl imines*

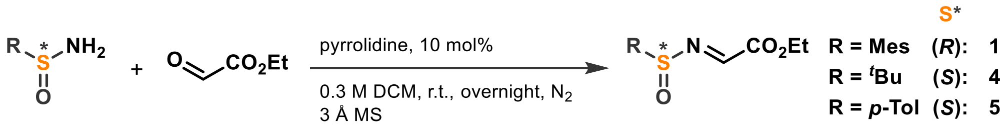

N-sulfinyl imines **1**, **4**, and **5** were synthesized according to a procedure adapted from reference 3. *N*-sulfinyl amide (1 equiv.) and 4 Å activated molecular sieves (2 g per 1 mmol of *N*-sulfinyl amide) were placed in a dry 25 mL round-bottom flask equipped with a stirring bar and a septum and evacuated and back-filled with N_2 three times. Anhydrous DCM (0.03 M), ethyl glyoxalate (1 equiv., 50% solution in toluene), and pyrrolidine (10 mol%) were added and the reaction mixture was stirred at room temperature under N_2 balloon. After 19 h the reaction mixture was filtered through celite washing with DCM, and concentrated on rotary evaporator resulting in a crude product as a yellow oil. The crude product was purified by column chromatography (silica gel, *n*-hexane/ethyl acetate 4:1) and the fractions containing the desired product were combined, concentrated on rotary evaporator, and dried under vacuo overnight, resulting in the desired product.

3. *Optimization of the reaction conditions*

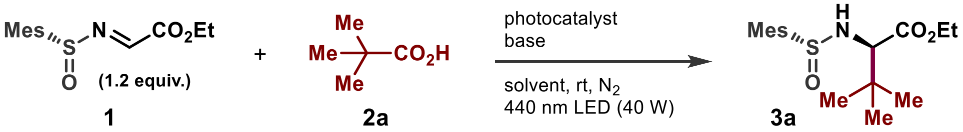

The reactions were typically performed on 0.1 mmol scale (relative to the limiting starting material). The *N*-sulfinyl imine **1**, photocatalyst, and the base were placed in the reaction vial equipped with a stirring bar and a septum. The solids were evacuated and back-filled with N_2 three times, followed by addition of a stock solution of pivalic acid **2a** in deaerated anhydrous $PhCF_3$ and short sonication. Thereafter, the reaction mixture was placed ca. 2 cm from the light source (440 nm LED, see Figure S1) and stirred with a fan cooling. The reactions were monitored by ^1H NMR with 1,3,5-trimethoxybenzene as an internal standard. Typically, 0.5 mL of the reaction mixture was taken at a time, filtered through a silica plug eluting with *n*-hexane/ethyl acetate 1:1, concentrated on rotary evaporator, dissolved in a stock solution of the internal standard in $CDCl_3$, and analyzed by ^1H NMR (500 MHz). When performing the reactions with small amounts of the photocatalysts (Table S1, entries 1–8), both photocatalyst and pivalic acid were added as stock solutions.

Table S1. Optimization of the reaction conditions with pivalic acid **2a** as the radical precursor.

entry	*photocatalyst*	*base*	*solvent*	*time*	*yield*[a]	*dr*[a]
**1**	[Ir(dF(CF_3)ppy)$_2$(dtbbpy)](PF_6), 1 mol%	Cs_2CO_3, 0.2 equiv.	DMSO, 0.1 M	20 min	—	–
**2**	[Ir(dF(CF_3)ppy)$_2$(dtbbpy)](PF_6), 1 mol%	Cs_2CO_3, 0.2 equiv.	$PhCF_3$, 0.05 M	20 min	65%	4 : 1
**3**	[Ir(dF(CF_3)ppy)$_2$(dtbbpy)](PF_6), 1 mol%	2,6-lutidine, 0.2 equiv.	$PhCF_3$, 0.05 M	20 min	—	—
**4**	[Ir(dF(CF_3)ppy)$_2$(dtbbpy)](PF_6), 1 mol%	DIPEA, 0.2 equiv.	$PhCF_3$, 0.05 M	20 min	< 5%	—
**5**	[Ir(dF(CF_3)ppy)$_2$(dtbbpy)](PF_6), 1 mol%	K_3PO_4, 0.2 equiv.	$PhCF_3$, 0.05 M	20 min	—	—
**6**	[Ir(dF(CF_3)ppy)$_2$(dtbbpy)](PF_6), 1 mol%	$Bu_4NH_2PO_4$, 0.2 equiv.	$PhCF_3$, 0.05 M	20 min	—	—
**7**	4CzIPN, 1 mol%	Cs_2CO_3, 0.2 equiv.	$PhCF_3$, 0.05 M	20 min	—	—
**8**	[Mes-Acr-Me](BF_4), 1 mol%	Cs_2CO_3, 0.2 equiv.	$PhCF_3$, 0.05 M	20 min	27%	> 95 : 5
**9**	[Mes-Acr-Me](BF_4), 5 mol%	Cs_2CO_3, 0.2 equiv.	$PhCF_3$, 0.05 M	20 min<br>60 min	48%<br>66%	> 95 : 5<br>> 95 : 5
**10**	[Mes-Acr-Me](BF_4), 5 mol%	Cs_2CO_3, 0.2 equiv.	MeCN, 0.05 M	20 min	15%	> 95 : 5
**11**	[Mes-Acr-Me](BF_4), 5 mol%	Cs_2CO_3, 0.2 equiv.	CH_2Cl_2, 0.05 M	20 min	24%	> 95 : 5
**12**	[Mes-Acr-Ph](BF_4), 5 mol%	Cs_2CO_3, 0.2 equiv.	$PhCF_3$, 0.05 M	60 min	73%	> 95 : 5
**13**	[Mes-2,7-Me_2Acr-Ph](BF_4), 5 mol%	Cs_2CO_3, 0.2 equiv.	$PhCF_3$, 0.05 M	60 min	78%	> 95 : 5
**14**	[Mes-2,7-Me_2Acr-Ph](BF_4), 5 mol%	Cs_2CO_3, 0.2 equiv.	PhF, 0.05 M	20 min	10%	> 95 : 5
**15**	[Mes-2,7-Me_2Acr-Ph](BF_4), 5 mol%	Cs_2CO_3, 0.2 equiv.	TFE, 0.05 M	20 min	< 5%	—
**16**	[Mes-2,7-Me_2Acr-Ph](BF_4), 5 mol%	2,6-lutidine, 0.2 equiv.	$PhCF_3$, 0.05 M	20 min	< 5%	—
**17**	[Mes-2,7-Me_2Acr-Ph](BF_4), 5 mol%	$Bu_4N(BuO)_2PO_2$,<br>0.5 equiv.	$PhCF_3$, 0.05 M	60 min<br>120 min	30%<br>45%	> 95 : 5<br>> 95 : 5
**18**	[Mes-2,7-Me_2Acr-Ph](BF_4), 5 mol%	$Bu_4NH_2PO_2$, 0.5 equiv.	$PhCF_3$, 0.05 M	60 min	< 5%	—
**19**	[Mes-2,7-Me_2Acr-Ph](BF_4), 5 mol%	TBD, 0.5 equiv.	$PhCF_3$, 0.05 M	60 min	51%	> 95 : 5
**20**	[Mes-2,7-Me_2Acr-Ph](BF_4), 5 mol%	Li_2CO_3, 0.2 equiv.	$PhCF_3$, 0.05 M	60 min	—	—
**21**	[Mes-2,7-Me_2Acr-Ph](BF_4), 5 mol%	K_3PO_4, 0.2 equiv.	$PhCF_3$, 0.05 M	60 min	80%	> 95 : 5
**22**	[Mes-2,7-Me_2Acr-Ph](BF_4), 5 mol%	K_2CO_3, 0.2 equiv.	$PhCF_3$, 0.05 M	60 min	84%	> 95 : 5
**23**	[Mes-2,7-Me_2Acr-Ph](BF_4), 5 mol%	K_2CO_3, 0.05 equiv.	$PhCF_3$, 0.05 M	20 min	< 5%	—
**24**	[Mes-2,7-Me_2Acr-Ph](BF_4), 5 mol%	K_2CO_3, 0.5 equiv.	$PhCF_3$, 0.05 M	60 min	85%	> 95 : 5

entry	photocatalyst	base	solvent	time	yield[a]	dr[a]
**25**	[Mes-2,7-Me_2Acr-Ph](BF_4), 5 mol%	K_2CO_3, 0.5 equiv.	$PhCF_3$, 0.1 M	60 min	77%	> 95 : 5
**26**[b]	[Mes-2,7-Me_2Acr-Ph](BF_4), 5 mol%	K_2CO_3, 0.5 equiv.	$PhCF_3$, 0.05 M	60 min	91%	> 95 : 5
**27**[c]	[Mes-2,7-Me_2Acr-Ph](BF_4), 5 mol%	K_2CO_3, 0.5 equiv.	$PhCF_3$, 0.05 M	60 min	95%	> 95 : 5

[a] Determined by ^{1}H NMR of the crude reaction mixture with 1,3,5-trimethoxybenzene as an internal standard. [b] 1.0 equiv. of sulfinyl imine **1** and 1.2 equiv. of pivalic acid **2a**. [c] 1.0 equiv. of sulfinyl imine **1** and 1.5 equiv. of pivalic acid **2a**.

Table S2. Optimization of the reaction conditions with hydrocinnamic acid **2o** as the radical precursor.

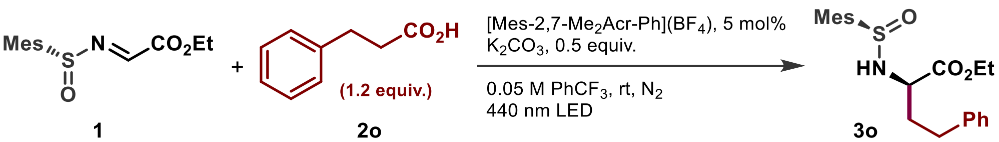

1 **2o** **3o**

entry	deviations from standard conditions	time	remaining imine[a]	yield[a]
1	***none***	60 min	0.67 equiv.	19%
		150 min	0.45 equiv.	22%
2	***solvent:*** $PhCF_3$/TFE 19:1, 0.05 M	60 min	0.47 equiv.	≈10%[b]
3	***solvent:*** $PhCF_3$/HFIP 19:1, 0.05 M	60 min	0.46 equiv.	12%
4	***solvent:*** $PhCF_3$/toluene 1:1, 0.05 M	20 min	0.82 equiv.	7%
5	***base:*** Bu_4NOAc, 0.5 equiv.	20 min	0.74 equiv.	11%
		60 min	0.54 equiv.	20%
6	***base:*** Bu_4NOBz, 0.5 equiv.	20 min	0.70 equiv.	9%
		60 min	0.49 equiv.	16%
7	***base:*** $Bu_4NOP(O)(OBu)_2$, 0.5 equiv.	60 min	0.75 equiv.	9%
		270 min	0.35 equiv.	15%
8	***base:*** CsF, 0.5 equiv.	60 min	0.65 equiv.	15%
9	***light:*** two LEDs (double intensity)	60 min	0.55 equiv.	20%
10	***additive:*** Bu_4NPF_6, 0.5 equiv.	60 min	0.62 equiv.	13%
11	***additive:*** Bu_4NOTf, 0.5 equiv.	60 min	0.66 equiv.	11%

[a] Determined by ^{1}H NMR of the crude reaction mixture with 1,3,5-trimethoxybenzene as an internal standard. [b] Complex mixture.

Table S3. Optimization of the reaction conditions with valeric acid **2p** as the radical precursor.

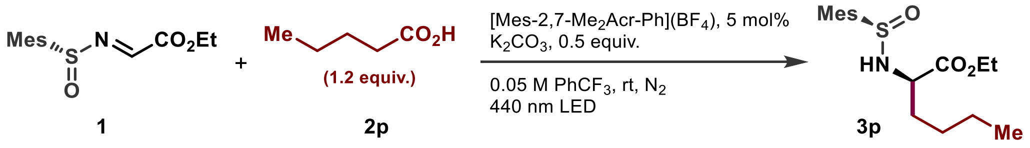

entry	deviations from standard conditions	time	remaining imine[a]	yield[a]
1	*none*	60 min	0.49 equiv.	32%
		180 min	0.34 equiv.	41%
2	***solvent:*** $PhCF_3$, 0.025 M	60 min	0.62 equiv.	20%
		180 min	0.52 equiv.	21%
3	***acid:*** 2.0 equiv.	60 min	0.18 equiv.	38%
		180 min	0 equiv.	38%
4	***light:*** half intensity	180 min	0.37 equiv.	39%
		360 min	0.22 equiv.	45%
5	***photocatalyst:*** 1 mol%	60 min	0. 56 equiv.	21%
6	***temperature:*** −20 °C	180 min	0.80 equiv.	11%
7	***temperature:*** 70 °C	60 min	0.41 equiv.	21%

[a] Determined by ^{1}H NMR of a crude reaction mixture with 1,3,5-trimethoxybenzene as an internal standard.

4. *General procedure for the decarboxylative radical addition to N-sulfinyl imine 1*

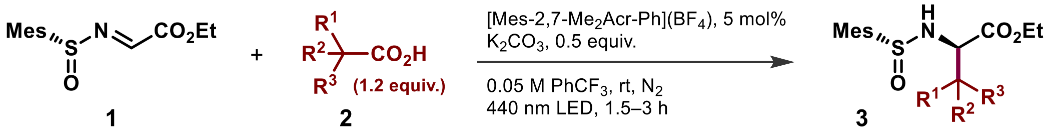

The *N*-sulfinyl imine **1** (1 equiv.), [Mes-2,7-Me_2Acr-Ph](BF_4) photocatalyst (5 mol%), K_2CO_3 (0.5 equiv.), and carboxylic acid **2** (1.2 equiv.) were placed in the reaction vial equipped with a stirring bar and a septum. The solids were evacuated and back-filled with N_2 three times, followed by addition of deaerated anhydrous $PhCF_3$ (0.05 M relative to *N*-sulfinyl imine **1**) and short sonication to obtain a fine suspension (acids that are liquid at room temperature were added as solutions in $PhCF_3$). Thereafter, the reaction mixture was placed ca. 2 cm from the light source (440 nm LED) and stirred under illumination with a fan cooling (see Figure S1). After the reaction was complete the mixture was filtered through a silica plug eluting with *n*-hexane/ethyl acetate 1:1, concentrated on rotary evaporator (40 °C), and the crude product **3** was purified by column chromatography or preparative TLC.

Note! If the stirring during the reaction is uneven and a solid residue is formed on the walls of the reaction vial above the liquid — the vial should be manually agitated in order to move the residue into the active stirring zone. Allowing the solid residue on the walls of the reaction vial to be irradiated for prolonged times can lead to formation of side-products.

Important! Products 3 tend to decompose in unstabilized acidic $CDCl_3$. In order to suppress the decomposition, $CDCl_3$ can be filtered through neutral Al_2O_3 dried in vacuo at 400–500 °C for ca. 20 min.

5. *Procedure for the 1 mmol scale synthesis of compound 3a*

The *N*-sulfinyl imine **1** (267.4 mg, 1 mmol, 1 equiv.), [Mes-2,7-Me_2Acr-Ph](BF_4) photocatalyst (24.5 mg, 0.05 mmol, 5 mol%), K_2CO_3 (69.1 mg, 0.5 mmol, 0.5 equiv.), and pivalic acid (122.6 mg, 1.2 mmol, 1.2 equiv.) were placed in the reaction vial (VWR, 24.0 mL sample vial, Art. No. 218-2226) equipped with a stirring bar and a septum. The solids were evacuated and back-filled with N_2 three times, followed by addition of deaerated anhydrous $PhCF_3$ (20 mL, 0.05 M) and short sonication to obtain a fine suspension. Thereafter, the reaction mixture was placed between two 440 nm LED lamps and stirred under illumination with a fan cooling (Figure S2). After 2.5 h the reaction mixture was filtered through silica eluting with *n*-hexane/ethyl acetate 1:1, concentrated on rotary evaporator (40 °C), and the crude product was purified by column chromatography with chloroform/ethyl acetate 35:1 as eluent. The purified product was dried in vacuo overnight, resulting in **3a** as white solid (247.2 mg, 76% yield, >95:5 α dr).

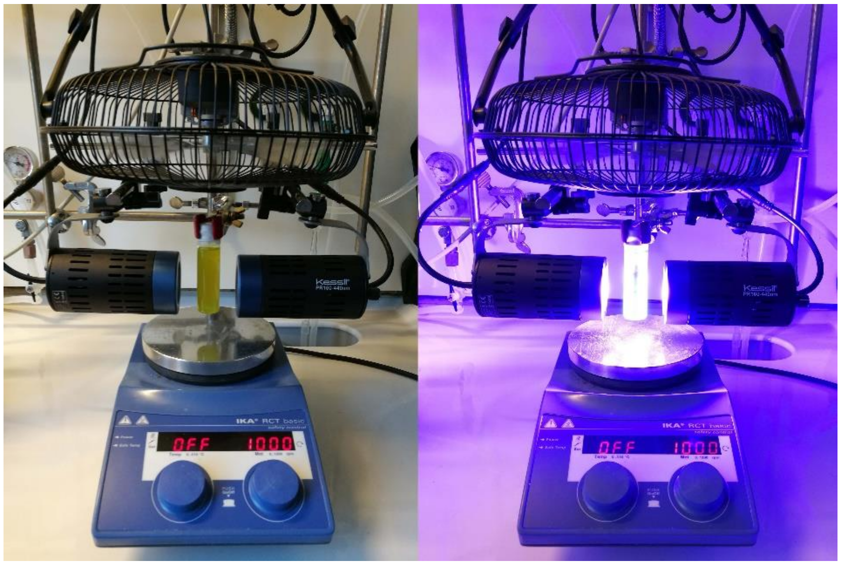

Figure S2. The reaction setup for 1 mmol scale synthesis.

6. *N-sulfinyl amide deprotection and determination of the absolute configuration for product 3a*

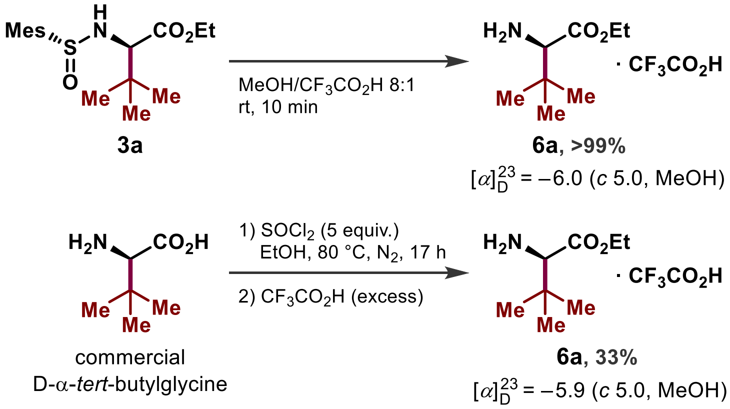

The proposed (*R*) absolute configuration of the *α*-stereocenter in the model product **3a** was confirmed by comparing specific optical rotation of the corresponding deprotected *α*-amino acid ethyl ester **6a** and specific optical rotation of a reference compound synthesized from commercial D-*α*-*tert*-butylglycine (98%, Sigma-Aldrich, Art. No. 269115): $[\alpha]^{23}_{D}$ = −6.0 (*c* 5.0, MeOH) for **6a** obtained from **3a**, and $[\alpha]^{23}_{D}$ = −5.9 (*c* 5.0, MeOH) for **6a** obtained from commercial D-*α*-*tert*-butylglycine. The absolute configuration of the α-stereocenter in **3a** was therefore determined as (*R*), which is in agreement with previous reports on radical addition reactions to chiral (*R*)-sulfinyl imines and the results of our computational studies (*vide infra*).

Synthesis of 6a from 3a: *N*-sulfinyl amide **3a** (32.1 mg, 0.1 mmol) was placed in a 5 mL round-bottom flask, dissolved in 1 mL MeOH and a solution of CF_3CO_2H (0.25 mL) in 1 mL of MeOH was added. The reaction mixture was stirred at room temperature under nitrogen for 10 min and the solvent was removed on rotary evaporator (40 °C). The solid residue was purified by column chromatography with a gradient CH_2Cl_2/MeOH 1:0 to 4:1 as eluent and dried in vacuo overnight, resulting in the product **6a** as a colorless solid (27.5 mg, >99% yield).

Synthesis of 6a from D-α-tert-butylglycine: D-*α*-*tert*-butylglycine (100 mg, 0.76 mmol) was suspended in 3 mL of absolute ethanol in a 10 mL round-bottom flask. Thionyl chloride (277 µL, 3.8 mmol, 5 equiv.) was added dropwise to the reaction mixture, the reaction flask was equipped with a reflux condenser, and heated to 80 °C under nitrogen in an oil bath. After 17 h the reaction mixture was concentrated in nitrogen flow and the obtained residue was suspended in *n*-hexane (3 mL) and extracted with water (3 x 3 mL). To the combined water extracts CF_3CO_2H (2 mL) was carefully added, and the resulting solution was extracted with ethyl acetate (3 x 8 mL). The combined organic phases were dried over Na_2SO_4 and concentrated. The crude product was purified by column chromatography with a gradient CH_2Cl_2/MeOH 20:1 to 10:1 as eluent and dried in vacuo overnight, resulting in the product **6a** as a colorless solid (68 mg, 33% yield). The ^{1}H NMR, ^{13}C NMR and ^{19}F NMR spectra for this product were identical to those of product **6a** obtained from **3a**.

7. *Electrochemical and fluorescence quenching studies*

Electrochemical measurements were performed under Ar in a one-compartment electrochemical cell with carbon fiber microelectrode (⌀ 33 µm, ALS, Art. No. 002002) as the working electrode, Pt coil as the auxiliary electrode, and saturated calomel electrode (SCE) as the reference electrode, using CHI750E bipotentiostat (CH Instruments). The cyclic voltammetry (CV) measurements were performed with 1 V s^{-1} scan rate on $PhCF_3$ solutions (4 mL) of *N*-sulfinyl imine **1** (4 mM), [Mes-Me_2Acr-Ph](BF_4) (1.5 mM) and [Ir(dF(CF_3)ppy)$_2$(dtbbpy)](PF_6) (1.5 mM), with 0.1 M Bu_4NBF_4 as the supporting electrolyte (≥99.0%, for electrochemical analysis, Fluka Analytical). The working electrode was thoroughly polished with 0.05 µm alumina prior each measurement. The relatively high scan rate was employed in order to suppress more complex coupled electrochemical processes that precede/follow the electron transfer steps and the small area working electrode was used in order to minimize the *iR*-drop. No *iR* compensation was applied.

The fluorescence quenching studies were performed under Ar using 10 x 10 mm quartz cuvettes. The steady-state measurements were performed on Varian Cary Eclipse fluorescence spectrophotometer and the time-resolved measurements were performed with a custom-build laser setup.[4] For the time-resolved measurements, a 450 nm laser (Thorlabs, NPL45C) was used as an excitation source with a Siglent SDG 1032X waveform generator (square wave, 10 ns laser pulses).

For steady-state fluorescence quenching measurements, a series of solutions of [Mes-Me_2Acr-Ph](BF_4) (15 µM) with pivalic acid (0 to 0.05 M) or tetrabutylammonium pivalate (0 to 0.1 M, prepared *in situ* by mixing equimolar amounts of pivalic acid and $Bu_4N(BuO)_2PO_2$ base) were prepared and the emission spectra were recorded at 440–750 nm with excitation at 420 nm (Figure S3). The decrease in emission intensity at 491 nm for the pivalate quencher was then used for calculation of the Stern-Volmer quenching constant (K_{SV} = 237.5 M^{-1}) as the slope of the linear region of the I_0/I vs. [quencher] plot (0–0.05 M, Figure S3, *top* and Figure 3B). Deviation from the linearity was observed at higher concentrations of the pivalate quencher (>0.05 M) and is likely due to contribution from the static quenching. No quenching was observed for pivalic acid (Figure S3, *bottom* and Figure 3B), while the quenching measurements for the base used to prepare tetrabutylammonium pivalate ($Bu_4N(BuO)_2PO_2$) were prohibited due to fast decomposition of the photocatalyst under basic conditions.

For time-resolved fluorescence quenching measurements, a series of solutions of [Mes-Me_2Acr-Ph](BF_4) (15 µM) with tetrabutylammonium pivalate (0 to 0.01 M, prepared *in situ* by mixing equimolar amounts of pivalic acid and $Bu_4N(BuO)_2PO_2$ base) were prepared and the emission decay traces were collected at 491 nm with excitation at 450 nm (Figures S4, *top*). The emission decay traces were fitted to a monoexponential decay function and the obtained fluorescence lifetimes (τ) were normalized to lifetime of the photocatalyst in the absence of the quencher (τ_0 = 14.9 ns) and the slope of the τ_0/τ vs. [quencher] plot ($k_q \cdot \tau_0$ = 102.2 M^{-1}) was used to calculate the bimolecular quenching constant (k_q = $6.8 \cdot 10^9$ M^{-1} s^{-1}, see Figure S4, *bottom*).

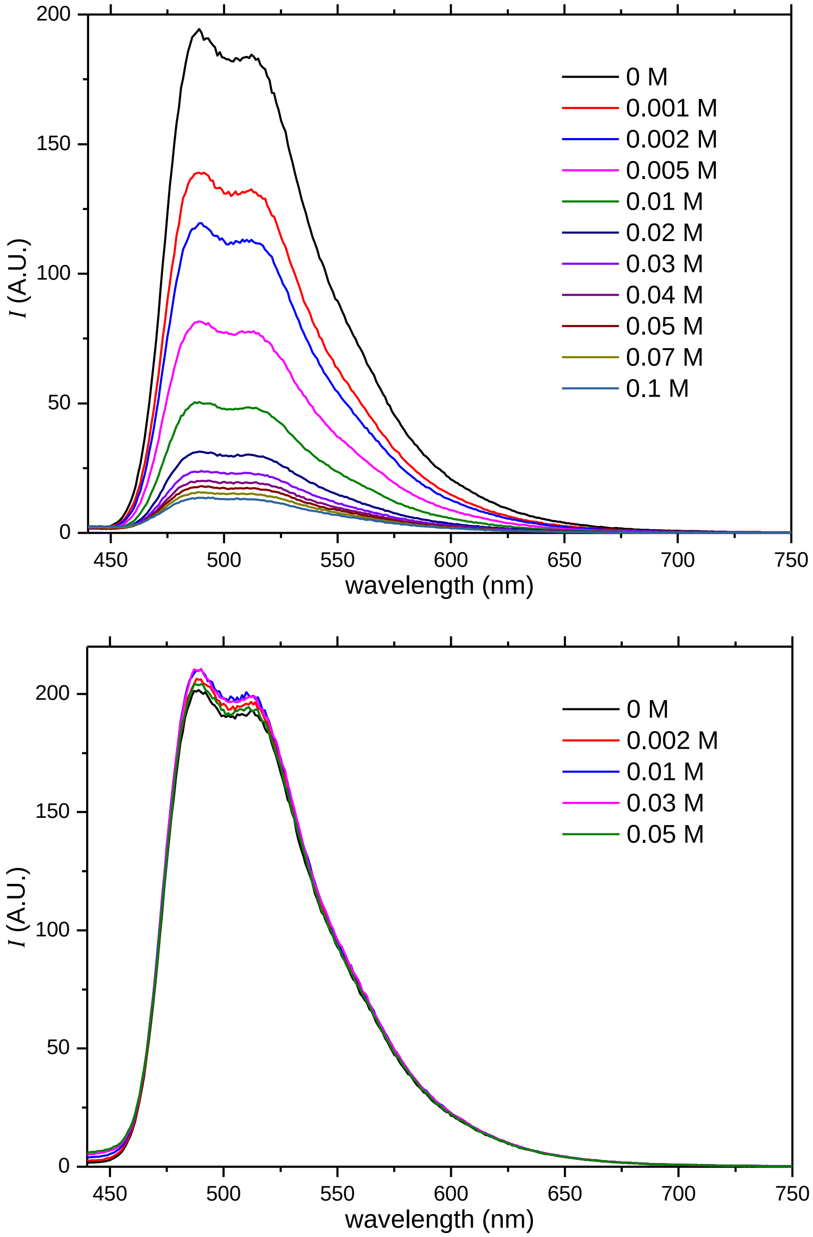

Figure S3. Steady-state fluorescence quenching measurements with [Mes-Me_2Acr-Ph](BF_4) (15 µM) and tetrabutylammonium pivalate (0–0.1 M, *top*) and pivalic acid (0–0.05 M, *bottom*).

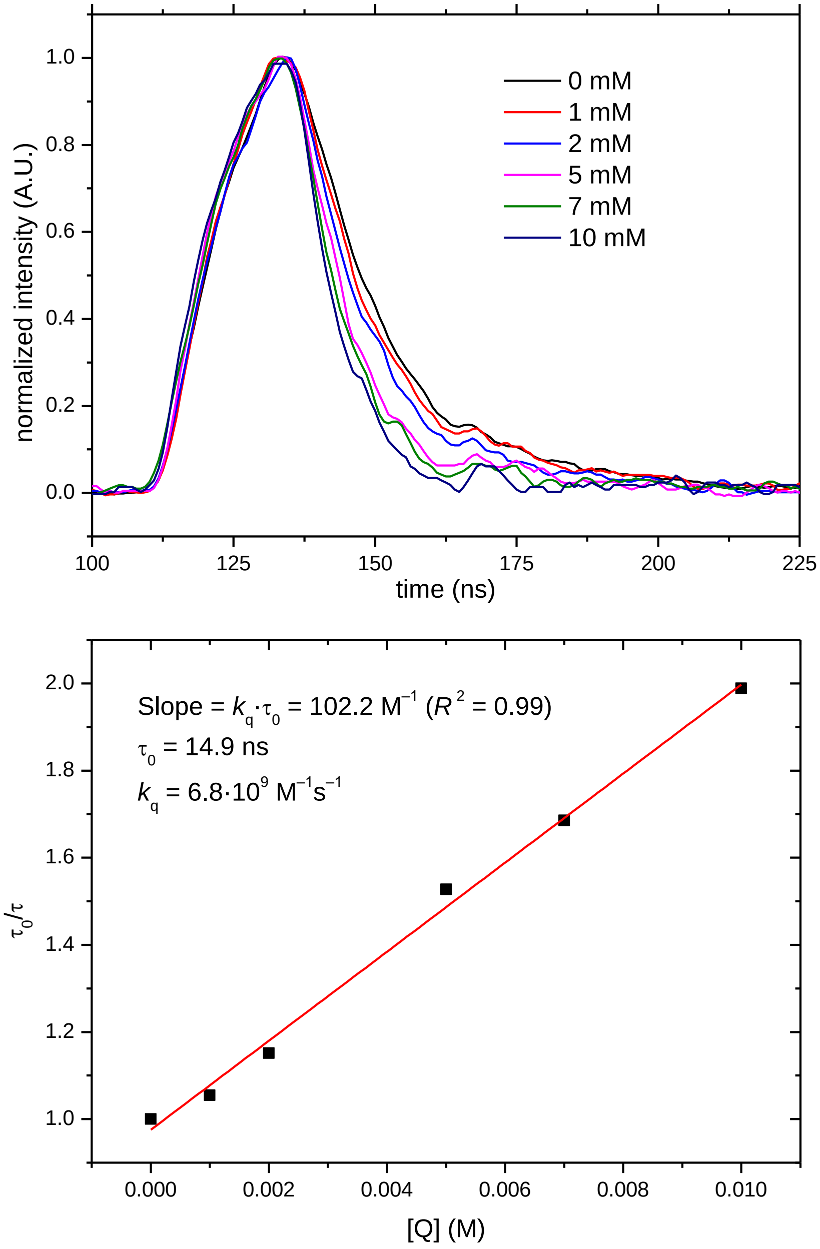

Figure S4. Time-resolved fluorescence quenching measurements with [Mes-Me_2Acr-Ph](BF_4) (15 µM) and tetrabutylammonium pivalate (0–0.01 M): normalized fluorescence decay traces (*top*) and determination of the bimolecular quenching constant k_q (*bottom*).

8. *Computational studies*

Formation of product **3a** from the *N*-sulfinyl imine **1** and pivalic acid **2a** was selected as the model reaction for the computational studies. All calculations were performed on the uM062X-D3/6-311+G(d,p)[5] level of theory using Gaussian 16 package, revision B.01.[6] The effect of the solvent (chlorobenzene) was modeled implicitly using the Solvation Model based on Density (SMD) method.[7] Vibrational analysis confirmed that all reported structures either represents true minima (no imaginary frequencies), or the true first order saddle points (for transition states, one imaginary frequency) on the potential energy surface. Free energies were obtained by adding the thermal correction term from the frequency analysis to the electronic energy. To account for the change in the standard state (1 atm → 1 M) an entropic correction term of 1.89 kcal/mol (−RTlnQ) was added to all considered species. Non-covalent interaction plots (NCI)[8] were generated using Maestro Version 11.9.011[9] and Jaguar,[10] as implemented in the Schrödinger suite.

8.1 *Conformation analysis for N-sulfinyl imine 1 and the corresponding α-amino radical*

Previously, Alemán and co-workers suggested that the *s-cis* conformer of *N*-sulfinyl imine **1** should be more stable compared to the *s-trans* conformer due to the hydrogen bonding between the imine hydrogen and sulfoxide oxygen.[11] To confirm this, we performed a relaxed scan for the O–S–N–C dihedral angle with 30° increments on the M062X-3D/631+G(d)/SMD(chlorobenzene) level of theory (Figure S5). The identified minimum (D = 0°) indeed corresponded to the *s-cis* conformation, which was then reoptimized on the M062X-D3/6-311+G(d,p)/SMD(chlorobenzene) level of theory. For comparison, two *s-trans* conformers (***s-trans*-1** (D = 150°) and ***s-trans*-2** (D = −150°)) were also recalculated at the same level of theory. The structures of ***s-cis*** and ***s-trans*-1** conformers, their NCI plots, and the relative free energies are presented in Figures 3 and S3. The ***s-cis*** conformer was found to be favored by 3.8 kcal/mol compared to the ***s-trans*-1** conformer, an energy difference representing a >99.8:0.2 ratio considering a 1:1 Boltzmann distribution at room temperature (298.15 K). This result is in good agreement with the previous report investigating *S*-*t*Bu-*N*-sulfinyl imines, where the *s-cis* conformer was calculated to be 4.0 kcal/mol more stable than the *s-trans* conformer.[12] However, sulfinyl imine from the previous report did not include an additional potential hydrogen bond acceptor, *i.e.* the carbonyl oxygen present in *N*-sulfinyl imine **1**. NCI plots were generated with Jaguar at the M062X-D3/6-311+G(d,p)/CPCM(chlorobenzene) level of theory to corroborate these interactions (Figure S5, *bottom*). A favorable interaction was found between the imine hydrogen and the sulfinyl oxygen in the ***s-cis*** conformer, as proposed. However, no bond critical point could be located between the imine hydrogen and the carbonyl oxygen in neither ***s-cis*** nor ***s-trans*-1** conformer, suggesting that this interaction is negligible.

The conformation analysis was also repeated for *N*-sulfinyl α-amino radical derived from imine **1**, as the key intermediate for formation of **3** *via* an alternative radical-radical coupling mechanism. A relaxed scan for the O–S–N–C dihedral angle with 30° increments was performed on the same level of theory as for **1**, demonstrating significantly smaller barriers for transition between different conformations of the α-amino radical derived from **1** (ca. 2.5 kcal/mol) compared to the imine **1** (ca. 8.0 kcal/mol, Figure S5). The latter suggests that the alternative radical-radical coupling pathway should proceed with a lower level of diastereoselectivity compared to the radical addition to *N*-sulfinyl imine.

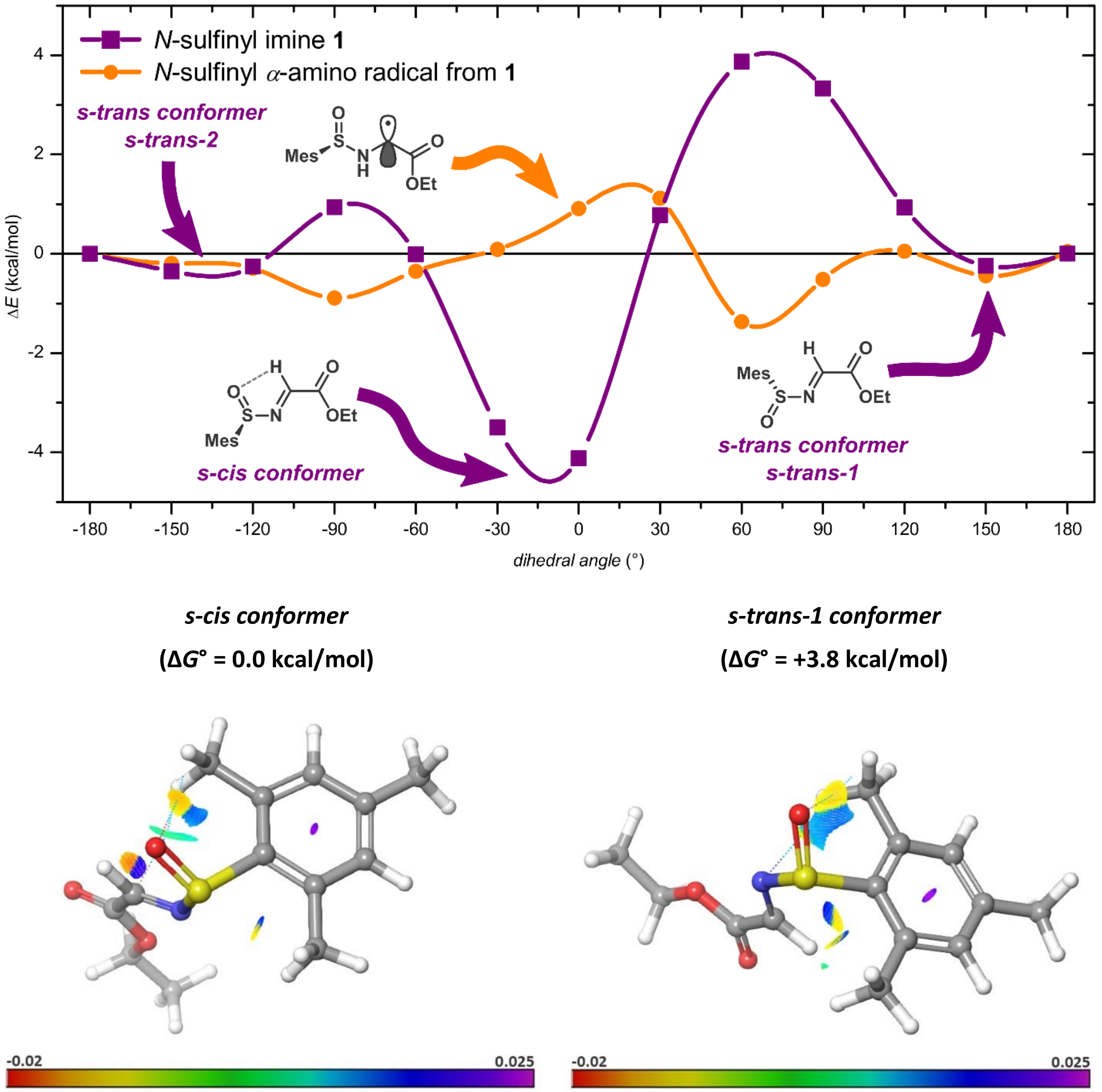

Figure S5. *Top:* Potential energy curves for *N*-sulfinyl imine **1** (***violet***) and *N*-sulfinyl α-amino radical from imine **1** (***orange***) as derived from a relaxed scan of the O-S-N-C dihedral angle. Calculations performed on the M062X-3D/631+G(d)/SMD(chlorobenzene) level of theory. *Bottom:* NCI plots and the relative free energies (kcal/mol) of the ***s-cis*** and ***s-trans*-1** conformations of the *N*-sulfinyl imine **1**.

To estimate the strength of the intramolecular hydrogen bonding between the imine hydrogen and sulfoxide oxygen in *N*-sulfinyl imine **1**, the relative stabilities of the corresponding conformers of *N*-sulfinyl imidoylfluoride were evaluated at the M062X-D3/6-311+G(d,p)/SMD(chlorobenzene) level of theory (Figure S6). The comparison demonstrated that the *s-trans* conformer is 0.6 kcal/mol more stable compared to the *s-cis* conformer, indicating that the intramolecular hydrogen bond in the sulfinyl imine **1** is worth approximately 4.4 kcal/mol.

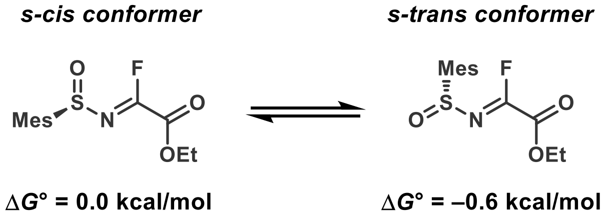

Figure S6. The relative free energies of the *s-cis* and *s-trans* conformers of the *N*-sulfinyl imidoyl fluoride.

8.2 *Addition of the tert-butyl radical to N-sulfinyl imine 1*

Next, the stereodetermining radical addition of the *tert*-butyl radical derived from pivalic acid **2a** to the *N*-sulfinyl imine **1** was investigated. The computed free energy surface of the reaction is presented in Figure 3. Formation of the precomplex is slightly endergonic for both *re* and *si* precomplexes, by 1.3 kcal/mol and 3.3 kcal/mol, respectively, and the activation barriers for the *re*- and *si*-additions were found to be 6.4 and 10.2 kcal/mol. The observed barriers originate primarily from the entropic cost of combining the two reacting species, as seen by the negative activation enthalpy ($\Delta H^{\ddagger}$) for both transition states compared to the free species. However, the difference in activation energy is almost exclusively due to enthalpic terms ($\Delta\Delta G^{\ddagger}$ = 3.8 kcal/mol, $\Delta\Delta H^{\ddagger}$ = 3.4 kcal/mol). In both cases, addition of the *tert*-butyl radical to the *N*-sulfinyl imine is exergonic, presumably due to the formation of a more stable radical, while formation of the experimentally observed *R,R*-diastereomer is 2.5 kcal/mol more favored compared to the *R,S*-diastereomer. Hence, the formation of the *R,R*-diastereomer is favored both kinetically and thermodynamically.

The structure, selected distances and relative free energies of both ***re*-TS** and ***si*-TS** can be seen in Figure 3. The C–C distance in the forming bond and the incoming angle is similar for both transition states, albeit ***re*-TS** having a slightly longer C–C distance (2.27 Å vs. 2.22 Å) and a slightly wider incoming angle (∠NCC, 109.7° vs. 108.6°). The geometries agree qualitatively with a transition state for the addition of an isopropyl radical to glyoxylate oxime methyl ether reported previously by Jørgensen and coworkers.[13] In Jørgensen's work, a lower energy barrier of 1.6 kcal/mol was predicted, presumably as a result of smaller stability of the isopropyl radical as compared to the *tert*-butyl radical.

Two reasons for the energetic ordering of the located transition states can be deduced. Firstly, the distance between the imine hydrogen and the sulfoxide oxygen is significantly longer in the ***si*-TS** compared to the ***re*-TS** (2.53 Å vs. 2.35 Å), indicating a weaker hydrogen bond. Secondly, there is a significant steric crowding between one of the methyl groups of the mesityl substituent in the ***si*-TS**, which is not present in the ***re*-TS**. This is an effect of a rotation of the mesityl group in ***si*-TS**, which makes room for the incoming *tert*-butyl radical. The O–S–C–C dihedral angle then becomes −50° in the ***re*-TS** and −2° in the ***si*-TS**, making the methyl group and the sulfoxide oxygen in ***si*-TS** almost completely coplanar. The NCI plots were generated for both of the transition states using Jaguar on the M062X-D3/6-311+G(d,p)/CPCM(chlorobenzene) level of theory to further visualize the described interactions (Figure S7). The NCI plots confirm the stronger hydrogen bond in the ***re*-TS** as compared to the ***si*-TS**, as can be seen by a redder bond critical point in the former. The increased steric encumberment between the sulfinyl oxygen, incoming *tert*-butyl radical, and the methyl group on the mesityl substituent in the ***si*-TS** compared to the ***re*-TS** can also be seen in the NCI plots.

re-TS

(Δ*G*° = +6.4 kcal/mol)

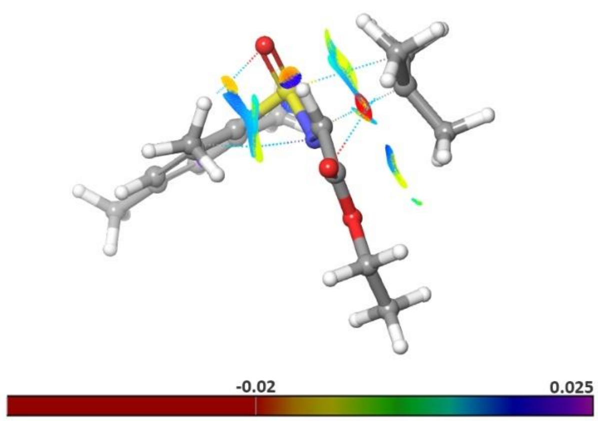

***si*-TS**

(Δ*G*° = +10.2 kcal/mol)

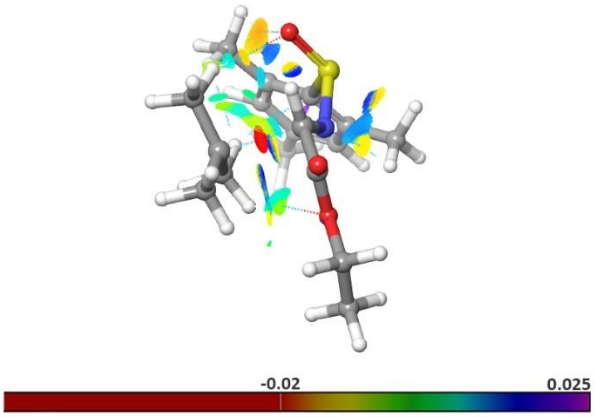

Figure S7. NCI plots and the relative free energies (kcal/mol) for ***re*-TS** and ***si*-TS**.

8.3 *Evaluation of transition states from s-trans-1 and s-trans-2 conformers*

To exclude the existence of other kinetically competent reaction paths, transition states resulting from attack of the *tert*-butyl radical to either of the *s-trans* conformers of the *N*-sulfinyl imine **1** (***s-trans*-1** (*D* = 150°) and ***s-trans*-2** (*D* = −150°)) were evaluated. The stability of both conformers and the Gibbs free energy of activation (Δ$G^‡$) for both *re*- and *si*-face addition of the *tert*-butyl radical in relation to the paths discussed in Section 8.2 can be seen in Table S4. All of the identified transition states are higher in Gibbs free energy than the located ***re*-TS** starting from the ***s-cis*** conformer, which is predicted to be the dominant one. Interestingly, addition from the *si*-face is favored for both of the *s-trans* conformers, showcasing once again the importance of the hydrogen bond between the imine hydrogen and the sulfinyl oxygen.

Table S4. Gibbs free energies for ***s-trans*-1** and ***s-trans*-2** conformers of *N*-sulfinyl imine **1** and the corresponding transition state energies for the tert-butyl radical addition to ***s-trans*-1** and ***s-trans*-2**.

	***s-trans*-1**	***s-trans*-2**
N-sulfinyl imine, Δ*G*°	3.8 kcal/mol	2.8 kcal/mol
***re*-TS**, Δ$G^‡$	14.6 kcal/mol	10.7 kcal/mol
***si*-TS**, Δ$G^‡$	9.7 kcal/mol	8.9 kcal/mol

8.4 *Evaluation of different functionals*

To evaluate the accuracy of the M062X-D3 functional, the single point energies of the ***re*-TS** and ***si*-TS** were recalculated using B3LYP-D3[14] and wB97XD functionals (Table S5).[15] Notably, the wB97XD functional is using the D2 version of Grimme's empirical dispersion[16] instead of the D3 version that is used for the other two functionals. As can be seen in Table S5, the three functionals are in excellent agreement with each other. The largest difference is between the M062X-D3 and the wB97XD functionals (0.4 kcal/mol), which is to be expected since they are using different models for the empirical dispersion. Nonetheless, the tested functionals all predict that the ***re*-TS** is favored by 3.4–3.8 kcal/mol compared to the ***si*-TS**.

Table S5. Relative free energy of activation for the ***re*-TS** and ***si*-TS** computed with different functionals for the single point electronic energy.

	**M062X-D3**	**B3LYP-D3**	**wB97XD**
***re*-TS**, $\Delta\Delta G^{\ddagger}$	0.0 kcal/mol	0.0 kcal/mol	0.0 kcal/mol
***si*-TS**, $\Delta\Delta G^{\ddagger}$	3.8 kcal/mol	3.7 kcal/mol	3.4 kcal/mol

8.5 *Evaluation of the solvent effect*

Chlorobenzene was chosen for SMD method in the above calculations as the solvent due to its structural similarity to $PhCF_3$. Meanwhile, CH_2Cl_2 is more similar to $PhCF_3$ in terms of polarity, as the dielectric constants for PhCl, $PhCF_3$ and CH_2Cl_2 are 5.71, 9.04 and 9.18, respectively. To evaluate the solvent effect, we reoptimized the *tert*-butyl radical, ***s-cis***, ***re*-TS** and ***si*-TS**, using SMD(CH_2Cl_2). As the result of the re-optimization on the same level of theory, only minor changes were observed for the geometries (**Section 11**) and negligible differences were observed for the activation barriers (Table S6).

Table S6. The effect of solvent on the free energy of activation for ***re*-TS** and ***si*-TS**

	**SMD (PhCl)**	**SMD (CH_2Cl_2)**
***re*-TS**, $\Delta G^{\ddagger}$	6.4 kcal/mol	5.8 kcal/mol
***si*-TS**, $\Delta G^{\ddagger}$	10.2 kcal/mol	10.2 kcal/mol

9. *Analytical data*

Compound **1** (ethyl (*R,E*)-2-((mesitylsulfinyl)imino)acetate)

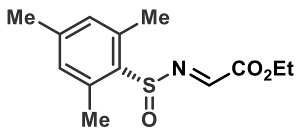

Synthesized according to the general procedure described in **Section 2** on 2.73 mmol scale. The product is a white solid (0.621 g, **85% yield**). The NMR spectra agree with the previously reported data (75% reported yield).[3]

^{1}H NMR (500 MHz, $CDCl_3$) δ 8.24 (s, 1H), 6.86 (s, 3H), 4.45–4.30 (m, 2H), 2.45 (s, 9H), 2.28 (s, 4H), 1.37 (t, *J* = 7.1 Hz, 3H).

^{13}C NMR (126 MHz, $CDCl_3$) δ 161.53, 154.05, 142.63, 138.93, 133.17, 131.19, 62.76, 21.26, 19.02, 14.21.

***R*f** = 0.53 (*n*-hexane/ethyl acetate 4:1).

HRMS (ESI): calcd for $C_{13}H_{17}NO_3SNa$ [M + Na]$^+$: 290.0827, found: 290.0821.

Compound **4** (ethyl (*S,E*)-2-((*tert*-butylsulfinyl)imino)acetate)

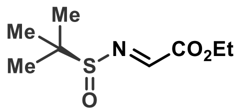

Synthesized according to the general procedure described in **Section 2** on 4.13 mmol scale. The product is a yellow oil (0.629 g, **74% yield**). The NMR spectra agree with the previously reported data.[17]

^{1}H NMR (400 MHz, $CDCl_3$) δ 8.00 (s, 1H), 4.38 (q, *J* = 6.6 Hz, 2H), 1.38 (t, *J* = 6.6 Hz, 3H), 1.27 (s, 9H).

^{13}C NMR (101 MHz, $CDCl_3$) δ 161.26, 155.74, 62.59, 59.05, 22.87, 14.20.

***R*f** = 0.43 (*n*-hexane/ethyl acetate 4:1).

HRMS (ESI): calcd for $C_8H_{15}NO_3S$ [M + H]$^+$: 206.0851, found: 206.0845.

Compound **5** (ethyl (*R,E*)-2-((*p*-tolylsulfinyl)imino)acetate)

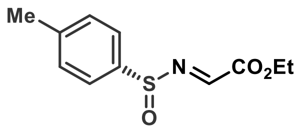

Synthesized according to the general procedure described in **Section 2** on 3.22 mmol scale. The product is a colorless oil (0.327 g, **42% yield**). The NMR spectra agree with the previously reported data.[18]

^{1}H NMR (400 MHz, $CDCl_3$) δ 8.14 (s, 1H), 7.60 (d, *J* = 7.1 Hz, 2H), 7.33 (d, *J* = 7.6 Hz, 2H), 4.43–4.26 (m, 2H), 2.41 (s, 3H), 1.35 (t, *J* = 6.9 Hz, 3H).

^{13}C NMR (101 MHz, $CDCl_3$) δ 161.39, 153.22, 142.69, 139.33, 62.75, 21.61, 14.17.

***R*f** = 0.39 (*n*-hexane/ethyl acetate 4:1).

HRMS (ESI): calcd for $C_{11}H_{13}NO_3SNa$ [M + Na]$^+$: 262.0514, found: 262.0512.

Compound **3a** (ethyl (*R*)-2-(((*R*)-mesitylsulfinyl)amino)-3,3-dimethylbutanoate)

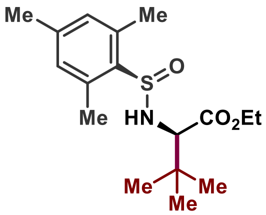

Synthesized from pivalic acid according to the general procedure described in **Section 4** on 0.3 mmol scale and 1.5 h reaction time (1 h reaction time for NMR yield). The crude product was purified by column chromatography using *n*-hexane/ethyl acetate 9:1 as eluent. The product is a white solid (79.1 mg, **81% yield**, >95:5 α dr). The NMR spectra agree with the previously reported data.[18]

^{1}H NMR (500 MHz, $CDCl_3$) δ 6.87 (s, 2H), 5.05 (d, *J* = 10.0 Hz, 1H), 4.34–4.14 (m, 2H), 3.60 (d, *J* = 10.1 Hz, 1H), 2.57 (s, 6H), 2.29 (s, 3H), 1.31 (t, *J* = 7.1 Hz, 3H), 0.97 (s, 9H).

^{13}C NMR (126 MHz, $CDCl_3$) δ 172.84, 140.99, 138.24, 136.90, 130.92, 66.63, 61.44, 35.16, 26.62, 21.19, 19.45, 14.32.

R_f = 0.33 (*n*-hexane/ethyl acetate 4:1)

HRMS (ESI): calcd for $C_{17}H_{27}NO_3SNa$ $[M + Na]^+$: 348.1609, found: 348.1608.

Compound **6a** (ethyl (*R*)-2-amino-3,3-dimethylbutanoate, trifluoroacetate salt)

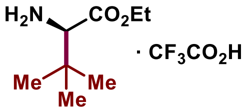

Synthesized from sulfinyl amide **3a** or D-*α*-*tert*-butylglycine according to the procedures described in **Section 6**.

^{1}H NMR (500 MHz, MeOH-*d*$_4$) δ 4.37–4.25 (m, 2H), 3.77 (s, 1H), 1.33 (t, *J* = 7.1 Hz, 3H), 1.11 (s, 9H).

^{13}C NMR (126 MHz, MeOH-*d*$_4$) δ 169.65, 63.38, 62.86, 34.26, 26.74, 14.41.

^{19}F NMR (377 MHz, MeOH-*d*$_4$) δ −77.37.

R_f = 0.74 (CH_2Cl_2/MeOH 10:1)

HRMS (ESI): calcd for $C_8H_{18}NO_2$ $[M + H]^+$: 160.1338, found: 160.1331.

$[\alpha]^{23}_D$ = −6.0 (*c* 5.0, MeOH), for **6a** obtained from **3a**.

$[\alpha]^{23}_D$ = −5.9 (*c* 5.0, MeOH), for **6a** obtained from commercial D-*α*-*tert*-butylglycine.

Compound **3b** (ethyl (*R*)-6-(2,5-dimethylphenoxy)-2-(((*R*)-mesitylsulfinyl)amino)-3,3-dimethylhexanoate)

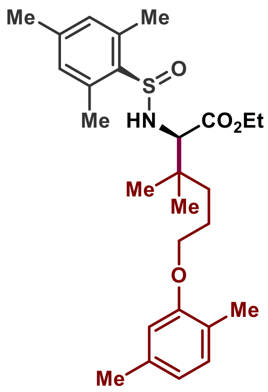

Synthesized from gemfibrozil according to the general procedure described in **Section 4** on 0.15 mmol scale and 2 h reaction time (2 h reaction time for NMR yield). The crude product (after filtration through silica plug and removal of the solvent) was dissolved in 20 mL of ethyl acetate and washed with 0.1 M aq. NaOH (5 x 10 mL) and water (10 mL), dried over anhydrous Na_2SO_4, concentrated, and purified by column chromatography using petroleum ether/CH_2Cl_2/ethyl acetate 8:1:1 as eluent. The product is a pale-yellow oil (31.3 mg, **44% yield**, >95:5 α dr).

^{1}H NMR (500 MHz, $CDCl_3$) δ 7.00 (d, *J* = 7.4 Hz, 1H), 6.87 (s, 2H), 6.66 (d, *J* = 7.4 Hz, 1H), 6.61 (s, 1H), 5.07 (d, *J* = 10.1 Hz, 1H), 4.31–4.18 (m, 2H), 3.90 (t, *J* = 6.4 Hz, 2H), 3.74 (d, *J* = 10.1 Hz, 1H), 2.57 (s, 6H), 2.30 (d, *J* = 8.4 Hz, 6H), 2.14 (s, 3H), 1.91–1.70 (m, 2H), 1.57–1.41 (m, 2H), 1.31 (t, *J* = 7.2 Hz, 3H), 0.97 (s, 6H).

^{13}C NMR (126 MHz, $CDCl_3$) δ 172.79, 157.09, 141.07, 138.17, 136.92, 136.61, 130.95, 130.45, 123.73, 120.88, 112.17, 68.41, 65.20, 61.54, 37.60, 35.80, 24.15, 23.87, 23.70, 21.55, 21.19, 19.43, 15.88, 14.32.

R_f = 0.24 (petroleum ether/CH_2Cl_2/ethyl acetate 8:1:1)

HRMS (ESI): calcd for $C_{27}H_{39}NO_4SNa$ $[M + Na]^+$: 496.2497, found: 496.2505.

Compound **3c** ((*R*)-*N*-((*R*)-2-(ethylperoxy)-1-(1-methylcyclohexyl)-2λ^2-ethyl)-2,4,6-trimethylbenzenesulfinamide)

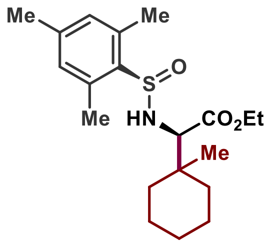

Synthesized from 1-methyl-1-cyclohexanecarboxylic acid according to the general procedure described in **Section 4** on 0.2 mmol scale and 1.5 h reaction time (1 h reaction time for NMR yield). The crude product was purified by column chromatography using chloroform/ethyl acetate 30:1 as eluent. The product is a pale-yellow oil (63.7 mg, **87% yield**, >95:5 α dr). The NMR spectra agree with the previously reported data.[18]

^{1}H NMR (500 MHz, $CDCl_3$) δ 6.86 (s, 2H), 5.01 (d, *J* = 10.1 Hz, 1H), 4.32–4.14 (m, 2H), 3.83 (d, *J* = 10.1 Hz, 1H), 2.57 (s, 6H), 2.29 (s, 3H), 1.68–1.38 (m, 7H), 1.38–1.22 (m, 3H), 1.30 (t, *J* = 7.2 Hz, 3H), 0.84 (s, 3H).

^{13}C NMR (126 MHz, $CDCl_3$) δ 172.87, 140.94, 138.29, 136.86, 130.90, 64.85, 61.36, 37.73, 34.96, 34.89, 26.08, 21.70, 21.66, 21.17, 20.73, 19.43, 14.32.

R_f = 0.20 (chloroform/ethyl acetate 30 :1)

HRMS (ESI): calcd for $C_{20}H_{31}NO_3SNa$ $[M + Na]^+$: 388.1922, found: 388.1908.

Compound **3d** (*tert*-butyl 4-((*R*)-2-(ethylperoxy)-1-(((*R*)-mesitylsulfinyl)amino)-2λ^2-ethyl)-4-methylpiperidine-1-carboxylate)

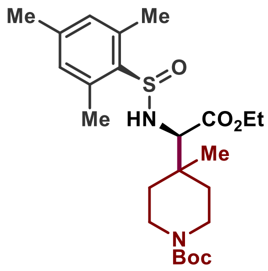

Synthesized from 1-*tert*-butoxycarbonyl-4-methyl-piperidine-4-carboxylic acid according to the general procedure described in **Section 4** on 0.2 mmol scale and 2 h reaction time (1 h reaction time for NMR yield). The crude product was purified by column chromatography using *n*-hexane/CH_2Cl_2/ethyl acetate 6:1:1 as eluent. The product is a pale-yellow oil (30.6 mg, **33% yield**, >95:5 α dr).

^{1}H NMR (500 MHz, $CDCl_3$) δ 6.87 (s, 2H), 5.04 (*d*, *J* = 10.0 Hz, 1H), 4.31–4.15 (m, 2H), 3.77 (d, *J* = 9.9 Hz, 1H), 3.80–3.60 (m, 2H), 3.20–3.06 (m, 2H), 2.56 (s, 6H), 2.29 (s, 3H), 1.72–1.61 (m, 1H), 1.60–1.51 (m, 1H), 1.45 (s, 9H), 1.43–1.37 (m, 1H), 1.30 (t, *J* = 7.1 Hz, 3H), 1.33–1.23 (m, 1H), 0.91 (s, 3H).

^{13}C NMR (126 MHz, $CDCl_3$) δ 172.20, 154.98, 141.20, 137.95, 136.88, 130.99, 79.67, 64.89, 61.72, 36.47, 34.07, 33.96, 31.73, 28.58, 22.79, 21.18, 19.45, 19.02, 14.31, 14.26.

***R*$_f$** = 0.16 (*n*-hexane/CH_2Cl_2/ethyl acetate 4:1:1)

HRMS (ESI): calcd for $C_{24}H_{38}N_2O_5SNa$ [M + Na]$^+$: 489.2399, found: 489.2408.

Compound **3e** (ethyl (*R*)-2-(((*R*)-mesitylsulfinyl)amino)-2-(3-methyloxetan-3-yl)acetate)

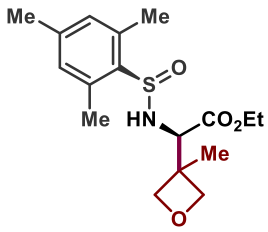

Synthesized from 3-methyloxetane-3-carboxylic acid according to the general procedure described in **Section 4** on 0.2 mmol scale and 3 h reaction time (2 h reaction time for NMR yield). The crude product was purified by column chromatography using petroleum ether/CH_2Cl_2/ethyl acetate 2:1:1 as eluent. The product is a pale-orange oil (36.7 mg, **54% yield**, >95:5 α dr). The NMR spectra agree with the previously reported data.[3]

^{1}H NMR (500 MHz, $CDCl_3$) δ 6.88 (s, 2H), 5.21 (d, *J* = 8.2 Hz, 1H), 4.84 (d, *J* = 6.3 Hz, 1H), 4.74 (d, *J* = 6.1 Hz, 1H), 4.44 (d, *J* = 8.1 Hz, 1H), 4.31 (d, *J* = 6.3 Hz, 1H), 4.28–4.23 (m, 2H), 4.23–4.15 (m, 1H), 2.58 (s, 6H), 2.29 (s, 3H), 1.27 (t, *J* = 7.1 Hz, 3H), 1.22 (s, 3H).

^{13}C NMR (126 MHz, $CDCl_3$) δ 171.48, 141.33, 137.73, 136.88, 131.03, 80.37, 80.32, 62.21, 62.18, 42.90, 21.17, 19.45, 19.31, 14.28.

***R*$_f$** = 0.28 (petroleum ether/CH_2Cl_2/ethyl acetate 2:1:1)

HRMS (ESI): calcd for $C_{17}H_{25}NO_4SNa$ [M + Na]$^+$: 362.1402, found: 162.1400.

Compound **3f** (ethyl (*R*)-2-(((*R*)-mesitylsulfinyl)amino)-2-(1-phenylcyclopropyl)acetate)

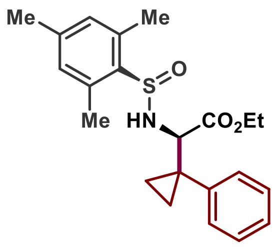

Synthesized from 1-phenyl-1-cyclopropanecarboxylic acid according to the general procedure described in **Section 4** on 0.2 mmol scale and 3 h reaction time (3 h reaction time for NMR yield). The crude product was purified by column chromatography with gradient *n*-hexane/CH_2Cl_2/ethyl acetate 10:1:1 to 6:1:1 as eluent. The product is a pale-red oil (47.1 mg, **61% yield**, >95:5 α dr). The NMR spectra agree with the previously reported data.[3]

^{1}H NMR (500 MHz, $CDCl_3$) δ 7.26–7.16 (m, 5H), 6.86 (s, 2H), 5.10 (d, *J* = 7.8 Hz, 1H), 4.21–4.09 (m, 2H), 3.77 (d, *J* = 7.8 Hz, 1H), 2.56 (s, 6H), 2.29 (s, 3H), 1.28–1.17 (m, 1H), 1.24 (t, *J* = 7.2 Hz, 3H), 1.07–1.01 (m, 1H), 1.01–0.94 (m, 1H), 0.93–0.87 (m, 1H).

^{13}C NMR (126 MHz, $CDCl_3$) δ 171.60, 140.92, 140.74, 137.91, 136.81, 130.91, 130.58, 128.16, 127.31, 64.24, 61.70, 29.60, 21.14, 19.40, 14.20, 11.64, 11.48.

***R*$_f$** = 0.33 (*n*-hexane/CH_2Cl_2/ethyl acetate 4:1:1)

HRMS (ESI): calcd for $C_{22}H_{27}NO_3SNa$ $[M + Na]^+$: 408.1609, found: 408.1600.

Compound **3g** (ethyl (*R*)-3-(4-chlorophenoxy)-2-(((*R*)-mesitylsulfinyl)amino)-3-methylbutanoate)

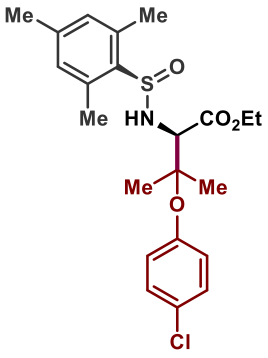

Synthesized from clofibric acid according to the general procedure described in **Section 4** on 0.15 mmol scale and 1.5 h reaction time (1 h reaction time for NMR yield). The crude product was purified by column chromatography with neutralized silica (prior to loading of the crude product the column was washed with the eluent containing 2% Et_3N, followed by pristine eluent) using petroleum ether/CH_2Cl_2/ethyl acetate 8:1:1 as eluent. The product is a pale-yellow oil (59.3 mg, **90% yield**, >95:5 α dr). The NMR spectra agree with the previously reported data.[3]

^{1}H NMR (500 MHz, $CDCl_3$) δ 7.23–7.17 (m, 2H), 6.90–6.84 (m, 4H), 5.33 (d, *J* = 9.9 Hz, 1H), 4.37–4.19 (m, 2H), 4.01 (d, *J* = 9.8 Hz, 1H), 2.58 (s, 6H), 2.29 (s, 3H), 1.36–1.25 (m, 9H).

^{13}C NMR (126 MHz, $CDCl_3$) δ 171.43, 152.97, 141.18, 137.89, 136.98, 131.00, 129.43, 129.19, 125.52, 81.87, 65.58, 61.89, 24.29, 23.85, 21.18, 19.46, 14.29.

***R*$_f$** = 0.16 (petroleum ether/CH_2Cl_2/ethyl acetate 8:1:1)

HRMS (ESI): calcd for $C_{22}H_{28}ClNO_4SNa$ $[M + Na]^+$: 460.1325, found: 460.1354.

Compound **3h** (ethyl (2*R*)-3-(4-(2,2-dichlorocyclopropyl)phenoxy)-2-(((*R*)-mesitylsulfinyl)amino)-3-methylbutanoate)

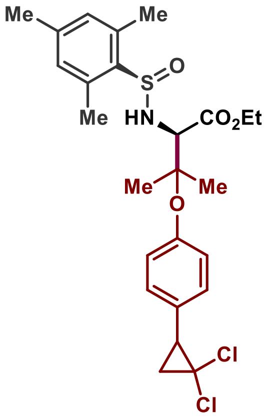

Synthesized from ciprofibrate according to the general procedure described in **Section 4** on 0.2 mmol scale and 1 h reaction time (1 h reaction time for NMR yield). The crude product was purified by column chromatography using *n*-hexane/CH_2Cl_2/ethyl acetate 8:1:1 as eluent. The product is a pale-violet oil (93.1 mg, **91% yield**, >95:5 α dr).

^{1}H NMR (500 MHz, $CDCl_3$) δ 7.14–7.09 (m, 2H), 6.94–6.89 (m, 2H), 6.88 (s, 2H), 5.34 (d, J = 10.0 Hz, 1H), 4.35–4.20 (m, 2H), 4.03 (d, J = 10.0 Hz, 1H), 2.84 (dd, J = 10.7, 8.3 Hz, 1H), 2.59 (s, 6H), 2.29 (s, 3H), 1.95 (dd, J = 10.6, 7.4 Hz, 1H), 1.79 (dd, J = 8.3, 7.4 Hz, 1H), 1.37–1.27 (m, 7H).

^{13}C NMR (126 MHz, $CDCl_3$) δ 171.51, 153.86, 141.12, 137.95, 136.97, 130.98, 130.29, 129.61, 123.90, 81.60, 65.69, 61.83, 60.89, 35.04, 26.02, 24.41, 23.87, 21.18, 19.47, 14.29.

R_f = 0.19 (*n*-hexane/CH_2Cl_2/ethyl acetate 6:1:1)

HRMS (ESI): calcd for $C_{25}H_{31}Cl_2NO_4SNa$ $[M + Na]^+$: 534.1249, found: 534.1230.

Compound **3i** (ethyl (2*R*)-3-(4-(4-chlorobenzoyl)phenoxy)-2-(((*R*)-mesitylsulfinyl)amino)-3-methylbutanoate)

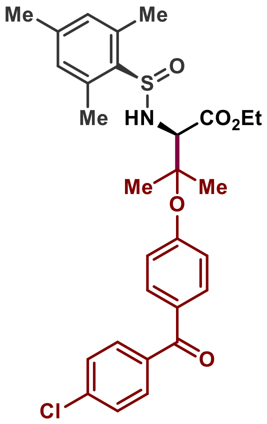

Synthesized from fenofibric acid according to the general procedure described in **Section 4** on 0.2 mmol scale and 1.5 h reaction time (1 h reaction time for NMR yield). The crude product was purified by column chromatography using *n*-hexane/CH_2Cl_2/ethyl acetate 10:1:1 as eluent. The product is a pale-yellow oil (94.0 mg, **87% yield**, >95:5 α dr).

^{1}H NMR (500 MHz, $CDCl_3$) δ 7.74–7.68 (m, 4H), 7.47–7.43 (m, 2H), 7.05–7.00 (m, 2H), 6.88 (s, 2H), 5.37 (d, J = 9.9 Hz, 1H), 4.37–4.20 (m, 2H), 4.09 (d, J = 9.9 Hz, 1H), 2.59 (s, 6H), 2.29 (s, 3H), 1.44 (s, 3H), 1.42 (s, 3H), 1.31 (t, J = 7.2 Hz, 3H).

^{13}C NMR (126 MHz, $CDCl_3$) δ 194.44, 171.19, 158.84, 141.23, 138.69, 137.67, 136.91, 136.17, 132.73, 132.40, 131.66, 131.31, 131.16, 130.98, 128.67, 128.49, 122.81, 115.45, 82.46, 65.55, 61.93, 24.35, 23.88, 21.13, 19.41, 14.23.

R_f = 0.29 (*n*-hexane/CH_2Cl_2/ethyl acetate 4:1:1)

HRMS (ESI): calcd for $C_{29}H_{33}ClNO_5S$ $[M + H]^+$: 542.1768, found: 542.1759.

Compound **3j** (ethyl (*R*)-2-(((*R*)-mesitylsulfinyl)amino)-2-((3a*S*,3b*R*,7a*S*,8a*S*)-2,2,5,5-tetramethyltetrahydro-8a*H*-[1,3]dioxolo[4',5':4,5]furo[3,2-d][1,3]dioxin-8a-yl)acetate)

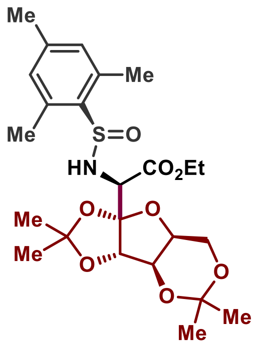

Synthesized from diprogulic acid according to the general procedure described in **Section 4** on 0.2 mmol scale and 3 h reaction time (2 h reaction time for NMR yield). In order to intensify the mixing, this reaction was performed in a 24 mL vial (23 x 88 mm, DWK Life Sciences, E-C Sample Vials, VWR Art. No. 66011-535) equipped with an X-shaped stirring bar (⌀ 16 mm). The crude product was purified by column chromatography with gradient *n*-hexane/CH_2Cl_2/ethyl acetate 8:1:1 to 1:1:2 as eluent. The product is a pale-yellow oil (34.7 mg, **37% yield**, >95:5 α dr, >95:5 β dr).

^{1}H NMR (500 MHz, $CDCl_3$) δ 6.80 (s, 2H), 5.46 (d, *J* = 8.8 Hz, 1H), 4.72 (s, 1H), 4.41 (d, *J* = 8.8 Hz, 1H), 4.26 (d, *J* = 1.9 Hz, 1H), 4.19–4.08 (m, 2H), 4.07–4.04 (m, 1H), 4.02 (d, *J* = 1.9 Hz, 2H), 2.60 (s, 6H), 2.24 (s, 3H), 1.45 (s, 3H), 1.39 (s, 3H), 1.35 (s, 3H), 1.22 (t, *J* = 7.1 Hz, 3H), 1.18 (s, 3H).

^{13}C NMR (126 MHz, $CDCl_3$) δ 169.24, 140.68, 137.33, 137.20, 130.86, 113.37, 112.79, 97.58, 86.82, 73.36, 72.92, 61.81, 61.11, 60.10, 28.58, 27.47, 26.63, 21.05, 19.49, 18.79, 14.08.

***R*$_f$** = 0.24 (*n*-hexane/CH_2Cl_2/ethyl acetate 2:1:1)

HRMS (ESI): calcd for $C_{24}H_{36}NO_8S$ $[M + H]^+$: 498.2162, found: 498.2152.

Compound **3k** (ethyl (*R*)-2-cyclohexyl-2-(((*R*)-mesitylsulfinyl)amino)acetate)

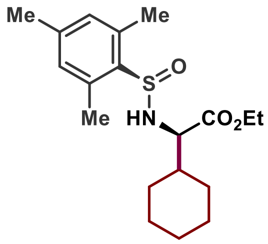

Synthesized from cyclohexanecarboxylic acid according to the general procedure described in **Section 4** on 0.15 mmol scale and 3 h reaction time (2 h reaction time for NMR yield). The crude product was purified by preparative TLC using chloroform/ethyl acetate 12:1 as eluent. The product is a pale-yellow oil (38.7 mg, **73% yield**, >95:5 α dr).

^{1}H NMR (500 MHz, $CDCl_3$) δ 6.87 (s, 2H), 5.01 (d, *J* = 9.3 Hz, 1H), 4.31–4.15 (m, 2H), 3.76 (dd, *J* = 9.3, 5.4 Hz, 1H), 2.59 (s, 6H), 2.29 (s, 3H), 1.79–1.55 (m, 5H), 1.30 (t, *J* = 7.1 Hz, 3H), 1.26–0.95 (m, 6H).

^{13}C NMR (126 MHz, $CDCl_3$) δ 173.26, 140.94, 138.32, 136.82, 130.91, 62.74, 61.61, 41.83, 29.73, 27.99, 26.13, 25.99, 21.17, 19.51, 14.32.

***R*$_f$** = 0.39 (chloroform/ethyl acetate 10:1)

HRMS (ESI): calcd for $C_{19}H_{29}NO_3SNa$ $[M + Na]^+$: 374.1766, found: 374.1758.

Compound **3l** (ethyl (*R*)-2-(4,4-difluorocyclohexyl)-2-(((*R*)-mesitylsulfinyl)amino)acetate)

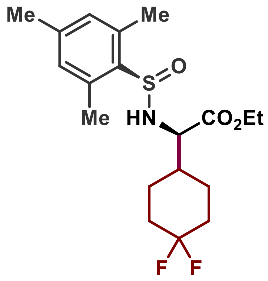

Synthesized from 4,4-difluorocyclohexanecarboxylic acid according to the general procedure described in **Section 4** on 0.2 mmol scale and 4 h reaction time (2 h reaction time for NMR yield). The crude product was purified by column chromatography using chloroform/ethyl acetate 35:1 as eluent. The product is a yellow oil (39.9 mg, **51% yield**, >95:5 α dr).

^{1}H NMR (500 MHz, $CDCl_3$) δ 6.88 (s, 2H), 5.06 (d, *J* = 8.9 Hz, 1H), 4.31–4.18 (m, 2H), 3.85 (dd, *J* = 8.9, 5.4 Hz, 1H), 2.58 (s, 6H), 2.29 (s, 3H), 2.19–2.01 (m, 2H), 1.87–1.52 (m, 6H), 1.49–1.36 (m, 1H), 1.31 (t, *J* = 7.1 Hz, 3H).

^{13}C NMR (126 MHz, $CDCl_3$) δ 172.73, 141.24, 137.90, 136.87, 131.02, 62.03, 61.10 (d, *J* = 2.4 Hz), 39.86 (d, *J* = 1.2 Hz), 33.26 (ddd, *J* = 25.6, 23.2, 18.3 Hz), 24.93 (dd, *J* = 203.1, 9.9 Hz), 21.17, 19.45, 14.28.

^{19}F NMR (377 MHz, $CDCl_3$) δ −92.08 (d, *J* = 236.6 Hz, 1F), −102.45 (d, *J* = 236.5 Hz, 1F).

R_f = 0.32 (chloroform/ethyl acetate 35:1)

HRMS (ESI): calcd for $C_{19}H_{27}F_2NO_3SNa$ $[M + Na]^+$: 410.1577, found: 410.1570.

Compound **3m** (ethyl (*R*)-2-(((*R*)-mesitylsulfinyl)amino)-2-(4-(trifluoromethyl)cyclohexyl)acetate)

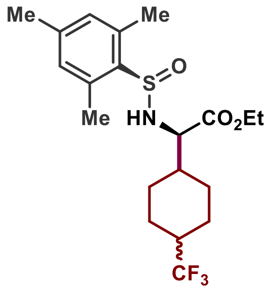

Synthesized from 4-(trifluoromethyl)cyclohexanecarboxylic acid (mixture of *cis* and *trans* isomers) according to the general procedure described in **Section 4** on 0.2 mmol scale and 4 h reaction time (2 h reaction time for NMR yield). The crude product was purified by column chromatography using *n*-hexane/CH_2Cl_2/ethyl acetate 4:1:1 as eluent. Two sets of fractions were combined and concentrated, resulting in pure *cis*/*trans*-diastereomer **3m-1** (23.0 mg, >95:5 α dr, >95:5 *cis*/*trans* dr) and a mixture of *cis*/*trans*-diastereomers **3m-1** and **3m-2** (28.0 mg, >95:5 α dr, 3:5 *cis*/*trans* dr). The products are yellow oils (in total 51.0 mg, **68% yield**, >95:5 α dr, 2:1 *cis*/*trans* dr).

^{1}H NMR for *cis/trans*-diastereomer **3m-1** (500 MHz, $CDCl_3$) δ 6.88 (s, 2H), 5.05 (d, *J* = 9.0 Hz, 1H), 4.31–4.17 (m, 2H), 3.81 (dd, *J* = 9.0, 4.9 Hz, 1H), 2.58 (s, 6H), 2.29 (s, 3H), 2.04–1.84 (m, 3H), 1.83–1.68 (m, 3H), 1.38–1.20 (m, 3H), 1.30 (t, *J* = 7.2 Hz, 3H), 1.08 (qd, *J* = 13.1, 3.3 Hz, 1H).

^{13}C NMR for *cis/trans*-diastereomer **3m-1** (126 MHz, $CDCl_3$) δ 172.79, 141.18, 138.04, 136.81, 130.99, 127.66 (q, *J* = 276.0 Hz), 61.96, 61.92, 41.60 (q, *J* = 26.7 Hz), 40.84, 27.98, 26.19, 24.49 (q, *J* = 2.6, Hz), 24.36 (q, *J* = 2.4, Hz), 21.17, 19.50, 14.30.

^{19}F NMR for *cis/trans*-diastereomer **3m-1** (377 MHz, $CDCl_3$) δ −73.82.

^{1}H NMR for a mixture of *cis/trans*-diastereomers **3m-1** and **3m-2** (500 MHz, CDCl3) δ 6.88 (s, 2H), 6.87 (s, 2H, diastereomer **3m-2**), 5.05 (d, *J* = 9.0 Hz, 1H), 4.93 (d, *J* = 10.0 Hz, 1H, diastereomer **3m-2**), 4.31–4.16 (m, 4H), 3.91 (dd, *J* = 9.9, 8.3 Hz, 1H, diastereomer **3m-2**), 3.81 (dd, *J* = 9.0, 4.9 Hz, 1H), 2.58 (s, 6H), 2.56 (s, 6H, diastereomer **3m-2**), 2.29 (s, 3H), 2.28 (s, 3H, diastereomer **3m-2**), 2.22–2.08 (m, 1H, diastereomer **3m-2**), 2.04–1.86 (m, 3H), 1.86–1.57 (m, 10H), 1.57–1.49 (m, 1H, diastereomer **3m-2**), 1.49–1.38 (m, 1H, diastereomer **3m-2**), 1.35–1.21 (m, 3H), 1.30 (t, *J* = 7.1 Hz, 6H), 1.08 (qd, *J* = 13.1, 3.4 Hz, 1H). *Mixture of two cis/trans-diastereomers is reported.*

^{13}C NMR for a mixture of *cis/trans*-diastereomers **3m-1** and **3m-2** (126 MHz, $CDCl_3$) δ 173.52 (*cis/trans*-diastereomer **3m-2**), 172.78, 141.17, 141.12 (*cis/trans*-diastereomer **3m-2**), 138.03, 137.95 (*cis/trans*-diastereomer **3m-2**), 136.87 (*cis/trans*-diastereomer **3m-2**), 136.81, 130.99, 130.97 (*cis/trans*-diastereomer **3m-2**), 128.10 (q, *J* = 279.7 Hz, *cis/trans*-diastereomer **3m-2**), 127.65 (q, *J* = 278.4 Hz), 61.95, 61.92, 61.73, *cis/trans*-diastereomer **3m-2**), 59.66 (*cis/trans*-diastereomer **3m-2**), 41.60 (q, *J* = 26.6 Hz), 40.83, 39.44 (q, *J* = 26.2 Hz, *cis/trans*-diastereomer **3m-2**), 38.49 (*cis/trans*-diastereomer **3m-2**), 27.97, 26.19, 25.49 (*cis/trans*-diastereomer **3m-2**), 24.64 (q, *J* = 2.4 Hz), 24.52 (q, *J* = 2.5 Hz), 24.29 (*cis/trans*-diastereomer **3m-2**), 21.56 (q, *J* = 2.2 Hz, *cis/trans*-diastereomer **3m-2**), 21.24 (q, *J* = 1.7 Hz, *cis/trans*-diastereomer **3m-2**), 21.17, 21.15 (*cis/trans*-diastereomer **3m-2**), 19.49, 19.37 (*cis/trans*-diastereomer **3m-2**), 14.29, 14.24 (*cis/trans*-diastereomer **3m-2**). *Mixture of two cis/trans-diastereomers is reported.*

^{19}F NMR for a mixture of *cis/trans*-diastereomers **3m-1** and **3m-2** (377 MHz, CDCl3) δ −70.81 (*cis/trans*-diastereomer **3m-2**), −73.82. *Mixture of two cis/trans-diastereomers is reported.*

***R*$_f$** = 0.40 & 0.33 for *cis-trans*-diastereomers **3m-1** and **3m-2**, respectively (*n*-hexane/CH_2Cl_2/ethyl acetate 4:1:1)

HRMS (ESI) for *cis/trans*-diastereomer **3m-1**: calcd for $C_{20}H_{28}F_3NO_3SNa$ $[M + Na]^+$: 442.1640, found: 442.1635.

HRMS (ESI) for a mixture of *cis/trans*-diastereomers **3m-1** and **3m-2**: calcd for $C_{20}H_{28}F_3NO_3SNa$ $[M + Na]^+$: 442.1640, found: 442.1638.

Compound **3n** (ethyl (*R*)-2-cyclobutyl-2-(((*R*)-mesitylsulfinyl)amino)acetate)

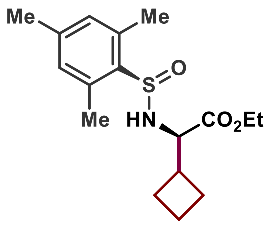

Synthesized from cyclohexanecarboxylic acid according to the general procedure described in **Section 4** on 0.2 mmol scale and 3 h reaction time (2.5 h reaction time for NMR yield). The crude product was purified by column chromatography using chloroform/ethyl acetate 35:1 as eluent. The product is a pale-pink oil (19.1 mg, **30% yield**, >95:5 α dr).

^{1}H NMR (500 MHz, $CDCl_3$) δ 6.87 (s, 2H), 5.04 (d, *J* = 8.9 Hz, 1H), 4.29–4.09 (m, 2H), 3.86 (dd, *J* = 8.9, 7.7 Hz, 1H), 2.66–2.53 (m, 1H), 2.59 (s, 6H), 2.28 (s, 3H), 2.07–1.91 (m, 3H), 1.91–1.72 (m, 3H), 1.27 (t, *J* = 7.1 Hz, 3H).

^{13}C NMR (126 MHz, $CDCl_3$) δ 172.88, 140.98, 138.18, 136.87, 130.93, 61.61, 61.31, 38.99, 24.94, 24.91, 21.16, 19.49, 18.02, 14.31.

***R*$_f$** = 0.20 (chloroform/ethyl acetate 35:1)

HRMS (ESI): calcd for $C_{17}H_{25}NO_3SNa$ $[M + Na]^+$: 346.1453, found: 346.1449.

Compound **3o** (ethyl (*R*)-2-(((*R*)-mesitylsulfinyl)amino)-4-phenylbutanoate)

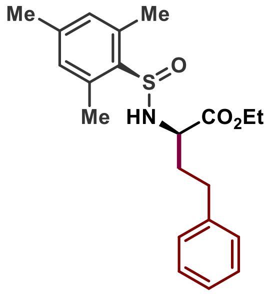

Synthesized from hydrocinnamic acid according to the general procedure described in **Section 4** on 0.2 mmol scale and 3 h reaction time (2.5 h reaction time for NMR yield). The crude product was purified by column chromatography using chloroform/ethyl acetate 35:1 as eluent. The product is a pale-orange oil (14.8 mg, **20% yield**, >95:5 α dr).

^{1}H NMR (500 MHz, $CDCl_3$) δ 7.32–7.26 (m, 2H), 7.23–7.13 (m, 3H), 6.89 (s, 2H), 5.15 (d, *J* = 8.4 Hz, 1H), 4.26–4.13 (m, 2H), 4.04–3.96 (m, 1H), 2.79–2.67 (m, 2H), 2.62 (s, 6H), 2.30 (s, 3H), 2.19–2.07 (m, 1H), 2.00–1.89 (m, 1H), 1.28 (t, *J* = 7.1 Hz, 3H).

^{13}C NMR (126 MHz, $CDCl_3$) δ 173.45, 141.14, 140.86, 138.05, 136.89, 131.01, 128.67, 128.59, 126.34, 61.90, 56.85, 35.79, 31.75, 21.19, 19.51, 14.28.

***R*$_f$** = 0.22 (chloroform/ethyl acetate 35:1)

HRMS (ESI): calcd for $C_{21}H_{27}NO_3SNa$ $[M + Na]^+$: 396.1609, found: 396.1604.

Compound **3p** (ethyl (*R*)-2-(((*R*)-mesitylsulfinyl)amino)hexanoate)

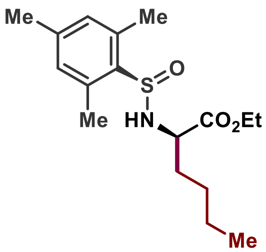

Synthesized from *n*-valeric acid according to the general procedure described in **Section 4** on 0.2 mmol scale and 3 h reaction time (3 h reaction time for NMR yield). The crude product was purified by column chromatography using chloroform/ethyl acetate 35:1 as eluent. The product is a pale-orange oil (25.2 mg, **39% yield**, >95:5 α dr).

^{1}H NMR (500 MHz, $CDCl_3$) δ 6.87 (s, 2H), 5.04 (*d*, J = 8.5 Hz, 1H), 4.29–4.13 (m, 2H), 3.95 (td, *J* = 8.3, 4.8 Hz, 1H), 2.59 (s, 6H), 2.28 (s, 3H), 1.85–1.75 (m, 1H), 1.68–1.57 (m, 1H), 1.43–1.30 (m, 4H), 1.29 (t, *J* = 7.2 Hz, 3H), 0.92–0.85 (m, 3H).

^{13}C NMR (126 MHz, $CDCl_3$) δ 173.75, 140.99, 138.10, 136.82, 131.05, 130.94, 61.72, 57.39, 33.84, 27.59, 22.31, 21.16, 19.44, 14.27, 14.01.

R_f = 0.24 (chloroform/ethyl acetate 30:1)

HRMS (ESI): calcd for $C_{17}H_{27}NO_3SNa$ $[M + Na]^+$: 348.1609, found: 348.1623.

Compound **3q** (ethyl (*R*)-3-(1-(4-chlorobenzoyl)-5-methoxy-2-methyl-1*H*-indol-3-yl)-2-(((*R*)-mesitylsulfinyl)amino)propanoate)

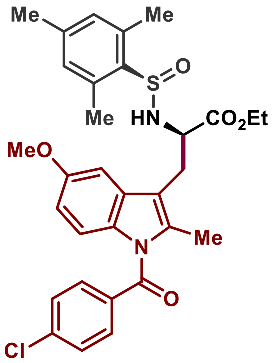

Synthesized from indomethacin according to the general procedure described in **Section 4** on 0.2 mmol scale and 3 h reaction time (2 h reaction time for NMR yield). The crude product was purified by column chromatography with gradient *n*-hexane/CH_2Cl_2/ethyl acetate 4:1:1 to 2:1:1 as eluent. The product is a pale-yellow solid (47.7 mg, **41% yield**, >95:5 α dr).

^{1}H NMR (500 MHz, $CDCl_3$) δ 7.63–7.58 (m, 2H), 7.48–7.42 (m, 2H), 6.87 (d, *J* = 9.1 Hz, 1H), 6.85 (d, *J* = 2.5 Hz, 1H), 6.81 (s, 2H), 6.65 (dd, *J* = 9.0, 2.5 Hz, 1H), 5.18 (d, *J* = 9.1 Hz, 1H), 4.29–4.14 (m, 3H), 3.68 (s, 3H), 3.17 (dd, *J* = 14.4, 5.2 Hz, 1H), 3.00 (dd, *J* = 14.5, 8.2 Hz, 1H), 2.40 (s, 6H), 2.27 (s, 3H), 2.23 (s, 3H), 1.24 (t, *J* = 7.2 Hz, 3H).

^{13}C NMR (126 MHz, $CDCl_3$) δ 173.01, 168.30, 156.08, 141.03, 139.41, 138.00, 136.62, 135.99, 134.00, 131.26, 130.96, 130.89, 130.86, 129.24, 115.06, 114.81, 111.80, 101.35, 62.13, 58.00, 55.62, 29.86, 21.15, 19.19, 14.16, 13.58.

R_f = 0.25 (*n*-hexane/CH_2Cl_2/ethyl acetate 3:1:1)

HRMS (ESI): calcd for $C_{31}H_{34}ClN_2O_5S$ $[M + H]^+$: 581.1877, found: 581.1869.

Compound **6q** (ethyl (*R*)-2-amino-3-(1-(4-chlorobenzoyl)-5-methoxy-2-methyl-1*H*-indol-3-yl)propanoate)

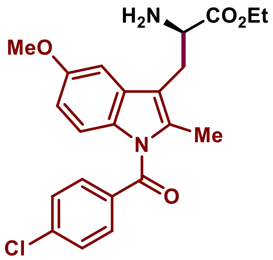

Synthesized from compound **3q** according to the *N*-sulfinyl amide deprotection procedure described for compound **3a** in **Section 6** on 0.068 mmol scale. The crude product was purified by column chromatography with gradient CH_2Cl_2/MeOH 20:1 to 10:1 as eluent. The product is a pale-yellow oil (27.9 mg, **99% yield**).

^{1}H NMR (500 MHz, $CDCl_3$) δ 7.64 (d, *J* = 8.5 Hz, 2H), 7.46 (d, *J* = 8.5 Hz, 2H), 6.98 (d, *J* = 2.5 Hz, 1H), 6.86 (d, *J* = 9.0 Hz, 1H), 6.66 (dd, *J* = 9.0, 2.6 Hz, 1H), 4.21–4.07 (m, 2H), 3.83 (s, 3H), 3.78 (t, *J* = 6.9 Hz, 1H), 3.14 (dd, *J* = 14.3, 5.6 Hz, 1H), 2.93 (dd, *J* = 14.3, 7.9 Hz, 1H), 2.36 (s, 3H), 1.22 (t, *J* = 7.1 Hz, 3H).

^{13}C NMR (126 MHz, $CDCl_3$) δ 175.33, 168.40, 156.10, 139.34, 135.92, 134.13, 131.27, 131.16, 131.09, 129.24, 115.53, 115.08, 111.48, 101.67, 61.26, 55.90, 54.97, 30.25, 14.28, 13.65.

***R*$_f$** = 0.59 (CH_2Cl_2/MeOH 10:1)

HRMS (ESI): calcd for $C_{22}H_{24}ClN_2O_4$ $[M + H]^+$: 415.1419, found: 415.1413.

$[\alpha]^{27}_D$ = −1.14 (*c* 4.0, $CHCl_3$)

Compound **3r** (*tert*-butyl (*R*)-2-((*R*)-2-ethoxy-1-(((*R*)-mesitylsulfinyl)amino)-2-oxoethyl)piperidine-1-carboxylate)

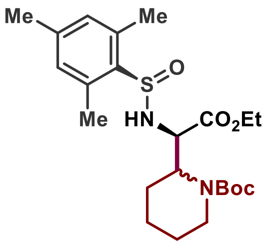

Synthesized from Boc-DL-pipecolinic acid according to the general procedure described in **Section 4** on 0.2 mmol scale and 1.5 h reaction time (1 h reaction time for NMR yield). The crude product was purified by column chromatography using *n*-hexane/CH_2Cl_2/ethyl acetate 6:1:1 as eluent. Two sets of fractions were combined and concentrated, resulting in a pure β-diastereomer **3r-1** as a mixture of two rotamers (41.5 mg, >95:5 α dr, >95:5 β dr) and a pure β-diastereomer **3r-2** (23.2 mg, >95:5 α dr, >95:5 β). The products are colorless oils (in total 64.7 mg, **71% yield**, >95:5 α dr, 2:1 β dr).

^{1}H NMR for β-diastereomer **3r-1** (500 MHz, $CDCl_3$) δ 6.80 (s, 2H), 6.79 (s, 2H), 5.19 (d, *J* = 10.7 Hz, 1H), 4.84 (d, *J* = 8.8 Hz, 1H), 4.45 (d, *J* = 10.7 Hz, 1H), 4.33–4.00 (m, 8H), 3.90 (d, *J* = 12.0 Hz, 1H), 2.78 (t, *J* = 13.5 Hz, 1H), 2.71–2.59 (m, 1H), 2.53 (s, 12H), 2.22 (s, 6H), 1.73–1.57 (m, 10H), 1.46–1.36 (m, 2H), 1.31 (t, *J* = 6.7 Hz, 3H), 1.25 (t, *J* = 7.1 Hz, 3H), 1.18 (s, 9H), 1.05 (s, 9H). *Mixture of two rotamers (ca. 1:1) is reported*.

^{13}C NMR for β-diastereomer **3r-1** (126 MHz, $CDCl_3$) δ 173.10, 172.29, 156.33, 155.17, 141.03, 140.74, 137.98, 137.68, 136.76, 130.81, 79.78, 79.72, 61.85, 61.59, 57.74, 57.50, 52.99, 51.53, 39.99, 38.65, 28.46, 28.09, 27.94, 25.62,

25.45, 25.07, 24.94, 21.87, 21.61, 21.04, 19.43, 19.24, 19.15, 14.15. *Mixture of two rotamers (ca. 1:1) is reported.*

^{1}H NMR for β-diastereomer **3r-2** (500 MHz, $CDCl_3$) δ 6.87 (s, 2H), 5.15 (d, *J* = 9.8 Hz, 1H), 4.37–4.03 (m, 5H), 3.07–2.82 (m, 1H), 2.57 (s, 6H), 2.28 (s, 3H), 1.98 (d, *J* = 13.6 Hz, 1H), 1.64 (s, 2H), 1.52–1.32 (m, 3H), 1.40 (s, 9H), 1.27 (t, *J* = 7.2 Hz, 3H).

^{13}C NMR for β-diastereomer **3r-2** (126 MHz, $CDCl_3$) δ 172.60, 171.85, 154.73, 154.27, 141.25, 137.92, 136.81, 130.99, 79.99, 61.92, 56.48, 56.09, 54.20, 52.84, 40.52, 39.11, 28.57, 28.40, 25.00, 24.84, 21.17, 19.35, 18.97, 14.05.

***R*$_f$** = 0.22 & 0.14 for β-diastereomers **3r-1** and **3r-2**, respectively (*n*-hexane/CH_2Cl_2/ethyl acetate 4:1:1)

HRMS for β-diastereomer **3r-1** (ESI): calcd for $C_{23}H_{36}N_2O_5SNa$ $[M + Na]^+$: 475.2243, found: 475.2236.

HRMS for β-diastereomer **3r-2** (ESI): calcd for $C_{23}H_{36}N_2O_5SNa$ $[M + Na]^+$: 475.2243, found: 475.2337.

Compound **3s** (*tert*-butyl (*R,S*)-2-((*R*)-2-ethoxy-1-(((*R*)-mesitylsulfinyl)amino)-2-oxoethyl)pyrrolidine-1-carboxylate)

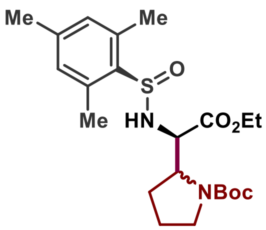

Synthesized from Boc-L-proline according to the general procedure described in **Section 4** on 0.2 mmol scale and 1.5 h reaction time (1 h reaction time for NMR yield). The crude product was purified by column chromatography using *n*-hexane/ethyl acetate 7:1 as eluent. The product is a colorless oil (0.0758 g, **86% yield**, >95:5 α dr, 1:1 β dr). Two rotamers for each β-diastereomers (1:1) were observed by NMR. The NMR spectra agree with the previously reported data.[3]

^{1}H NMR (500 MHz, $CDCl_3$) δ 6.85 (d, *J* = 14.0 Hz, 4H), 5.48 (d, *J* = 8.4 Hz, 1H), 5.39 (d, *J* = 7.6 Hz, 1H), 5.27 (d, *J* = 7.0 Hz, 1H), 5.11 (d, *J* = 8.7 Hz, 1H), 4.82 (dd, *J* = 7.6, 4.0 Hz, 1H), 4.73 (dd, *J* = 7.0, 3.8 Hz, 1H), 4.36 (s, 1H), 4.31–4.15 (m, 4H), 4.15–4.07 (m, 2H), 4.07–3.98 (m, 1H), 3.63–3.30 (m, 2H), 3.30–3.18 (m, 1H), 3.18–3.07 (m, 1H), 2.67–2.47 (m, 12H), 2.36–2.18 (m, 6H), 2.01–1.90 (m, 1H), 1.90–1.75 (m, 4H), 1.75–1.59 (m, 3H), 1.59–1.34 (m, 18H), 1.34–1.18 (m, 6H). *Mixture of two rotamers for each of two β-diastereomers is reported*.

^{13}C NMR (126 MHz, $CDCl_3$) δ 172.19, 171.99, 171.77, 155.30, 154.39, 154.06, 153.89, 140.97, 140.74, 138.62, 138.29, 137.72, 136.92, 136.64, 136.47, 130.84, 80.46, 80.22, 79.74, 62.12, 62.01, 61.66, 60.09, 59.43, 59.16, 58.75, 57.79, 57.32, 47.55, 47.37, 47.17, 46.69, 28.56, 28.49, 28.38, 27.71, 26.82, 26.07, 24.25, 23.78, 23.67, 22.76, 21.09, 21.05, 20.89, 19.45, 19.31, 14.20, 14.05. *Mixture of two rotamers for each of two β-diastereomers is reported*.

***R*f** = 0.26 (*n*-hexane/ethyl acetate 7:1)

HRMS (ESI): calcd for $C_{22}H_{34}N_2O_5SNa$ [M + Na]$^+$: 461.2086, found: 461.2083.

Compound **3t** (ethyl (2*R*,3*S*)-3-((*tert*-butoxycarbonyl)amino)-2-(((*R*)-mesitylsulfinyl)amino)-4-methylpentanoate)

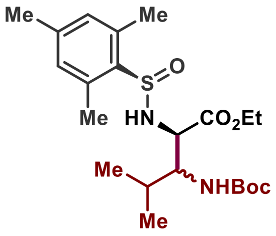

Synthesized from Boc-L-valine according to the general procedure described in **Section 4** on 0.2 mmol scale and 1 h reaction time (0.5 h reaction time for NMR yield). The crude product was purified by column chromatography using chloroform/ethyl acetate 20:1 as eluent. The product is pale-orange oil (87.4 mg, **99% yield**, >95:5 α dr, 1.5:1 β dr).

^{1}H NMR (500 MHz, $CDCl_3$) δ 6.94–6.80 (m, 4H), 5.31 (d, *J* = 7.4 Hz, 1H), 5.11 (d, *J* = 9.6 Hz, 1H, *minor β-diastereomer*), 4.69 (d, *J* = 10.3 Hz, 1H, *minor β-diastereomer*), 4.39 (d, *J* = 10.5 Hz, 1H), 4.30–4.13 (m, 5H), 4.03 (dd, *J* = 9.6, 6.6 Hz, 1H, *minor diastereomer*), 3.85–3.74 (m, 2H), 2.63–2.54 (m, 12H), 2.32–2.26 (m, 6H), 1.90–1.81 (m, 1H, *minor β-diastereomer*), 1.81–1.70 (m, 1H), 1.42–1.33 (m, 18H), 1.29 (td, *J* = 7.1, 2.9 Hz, 6H), 1.11 (d, *J* = 6.7 Hz, 4H), 0.97 (d, *J* = 6.7 Hz, 4H), 0.93 (d, *J* = 6.8 Hz, 2H), 0.88 (d, *J* = 6.8 Hz, 2H). *Mixture of two β-diastereomers is reported*.

^{13}C NMR (126 MHz, $CDCl_3$) δ 172.30, 172.02, 155.69, 155.42, 141.39, 141.17, 138.03, 137.73, 137.57, 136.95, 136.92, 131.08, 131.04, 131.01, 79.68, 79.50, 62.46, 62.00, 61.80, 59.55, 58.97, 57.84, 57.33, 30.51, 30.07, 29.37, 28.43, 28.41, 28.38, 28.35, 21.20, 21.17, 21.14, 20.17, 19.99, 19.70, 19.58, 19.55, 19.48, 19.43, 19.30, 18.74, 17.49, 14.24, 14.19, 14.16, 14.11. *Mixture of two β-diastereomers is reported*.

***R*f** = 0.26 & 0.40 for two β-diastereomers (chloroform/ethyl acetate 20:1)

HRMS (ESI): calcd for $C_{22}H_{36}N_2O_5SNa$ [M + Na]$^+$: 463.2243, found: 463.2237.

Compound **3u** (ethyl (2*R*)-3-((*tert*-butoxycarbonyl)amino)-2-(((*R*)-mesitylsulfinyl)amino)-4-phenylbutanoate)

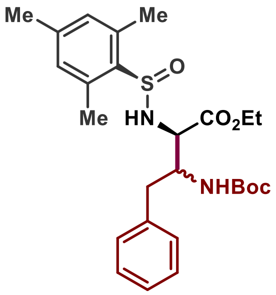

Synthesized from Boc-DL-phenylalanine according to the general procedure described in **Section 4** on 0.2 mmol scale and 3 h reaction time (1 h reaction time for NMR yield). The crude product was purified by column chromatography using *n*-hexane/CH_2Cl_2/ethyl acetate 6:1:1 as eluent. Two sets of fractions were combined and concentrated, resulting in pure β-diastereomer **3u-1** (20.5 mg, >95:5 α dr, >95:5 β dr) and a mixture of β-diastereomers **3u-1** and **3u-2** (78.6 mg, >95:5 α dr, 1:1 β dr). The products are pale-yellow oils (in total 99.1 mg, **>99% yield**, >95:5 α dr, 1.5:1 β dr).

^{1}H NMR for β-diastereomer **3u-1** (500 MHz, $CDCl_3$) δ 7.37–7.28 (m, 2H), 7.28–7.17 (m, 3H), 6.93 (s, 2H), 5.40 (d, *J* = 7.3 Hz, 1H), 4.58–4.41 (m, 2H), 4.28–4.12 (m, 2H), 4.04 (dd, *J* = 7.4, 2.3 Hz, 1H), 2.95–2.88 (m, 1H), 2.88–2.79 (m, 1H), 2.67 (s, 6H), 2.33 (s, 3H), 1.35–1.21 (m, 12H).

^{13}C NMR for β-diastereomer **3u-1** (126 MHz, $CDCl_3$) δ 171.83, 154.92, 141.50, 138.05, 137.11, 137.02, 131.13, 129.55, 129.43, 128.83, 128.64, 126.95, 79.73, 62.52, 58.00, 54.25, 39.12, 28.38, 28.29, 21.22, 19.64, 19.61, 14.10.

^{1}H NMR for a mixture of β-diastereomers **3u-1** and **3u-2** (500 MHz, $CDCl_3$) δ 7.35–7.29 (m, 2H), 7.29–7.23 (m, 4H), 7.23–7.17 (m, 2H, β-*diastereomer* **3u-2**), 7.14 (d, *J* = 7.5 Hz, 2H, β-*diastereomer* **3u-2**), 6.93 (s, 2H), 6.89 (s, 2H, β-*diastereomer* **3u-2**), 5.40 (d, *J* = 7.3 Hz, 1H), 5.28 (d, *J* = 8.7 Hz, 1H, β-*diastereomer* **3u-2**), 4.77 (d, *J* = 9.1 Hz, 1H, β-*diastereomer* **3u-2**), 4.57–4.42 (m, 2H), 4.36–4.30 (m, 1H, β-*diastereomer* **3u-2**), 4.30–4.07 (m, 7H), 4.04 (dd, *J* = 7.6, 2.2 Hz, 1H), 2.95–2.88 (m, 1H), 2.88–2.79 (m, 1H), 2.67 (s, 6H), 2.61 (s, 6H, β-*diastereomer* **3u-2**), 2.32 (s, 3H), 2.30 (s, 3H, β-*diastereomer* **3u-2**), 1.38 (s, 9H, β-*diastereomer* **3u-2**), 1.32–1.21 (m, 15H).

^{13}C NMR for a mixture of β-diastereomers **3u-1** and **3u-2** (126 MHz, $CDCl_3$) δ 171.82, 171.44, 155.06, 154.91, 141.49, 141.27, 138.04, 137.65, 137.10, 137.00, 136.90, 131.12, 131.08, 130.92, 129.54, 129.48, 129.42, 129.39, 128.82, 128.71, 128.63, 128.59, 128.52, 126.94, 126.84, 126.73, 79.89, 79.72, 62.51, 62.22, 62.06, 59.65, 57.99, 54.24, 53.68, 39.10, 36.36, 31.72, 28.42, 28.28, 23.20, 22.79, 21.20, 21.18, 19.63, 19.60, 19.58, 19.52, 14.25, 14.22, 14.09.

R_f = 0.23 & 0.19 for β-diastereomers **3u-1** and **3u-2**, respectively (*n*-hexane/CH_2Cl_2/ /ethyl acetate 6:1:1)

HRMS (ESI) for β-diastereomer **3u-1**: calcd for $C_{26}H_{36}N_2O_5SNa$ $[M + Na]^+$: 511.2243, found: 511.2237.

HRMS (ESI) for a mixture of β-diastereomers **3u-1** and **3u-2**: calcd for $C_{26}H_{36}N_2O_5SNa$ $[M + Na]^+$: 511.2243, found: 511.2238.

Compound **3v** (ethyl (*R*)-2-(((*R*)-mesitylsulfinyl)amino)-2-((*R*)-tetrahydrofuran-2-yl)acetate)

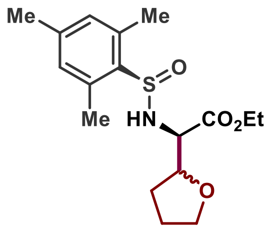

Synthesized from tetrahydro-2-furonic acid according to the general procedure described in **Section 4** on 2 mmol scale and 3 h reaction time (2 h reaction time for NMR yield). The crude product was purified by column chromatography using *n*-hexane/CH_2Cl_2/ethyl acetate 5:2:2 as eluent. The product is a pale-yellow oil (30.4 mg, **45% yield**, >95:5 α dr, 1.3:1 β dr).

^{1}H NMR (500 MHz, $CDCl_3$) δ 6.86 (s, 4H), 5.20 (d, *J* = 9.0 Hz, 1H), 5.14 (d, *J* = 8.6 Hz, 1H, *minor* β-*diastereomer*), 4.33–4.26 (m, 1H), 4.26–4.18 (m, 4H), 4.18–

4.11 (m, 1H), 4.11–4.01 (m, 1H), 4.00–3.90 (m, 1H), 3.89–3.80 (m, 1H), 3.80–3.71 (m, 2H), 3.71–3.63 (m, 1H), 2.64–2.54 (m, 12H), 2.28 (s, 6H), 2.05–1.96 (m, 1H), 1.96–1.88 (m, 2H), 1.88–1.75 (m, 5H), 1.33–1.23 (m, 6H). *Mixture of two* β-*diastereomers is reported*.

^{13}C NMR (126 MHz, $CDCl_3$) δ 171.66, 171.59, 141.03, 141.00, 140.97, 138.14, 137.89, 137.35, 137.30, 136.93, 136.86, 131.04, 130.99, 130.94, 130.93, 80.11, 79.44, 69.40, 69.04, 68.93, 62.00, 61.96, 61.82, 61.62, 60.56, 60.03, 60.01, 59.89, 28.30, 28.26, 28.04, 27.70, 26.22, 26.12, 25.75, 25.46, 21.16, 19.57, 19.53, 19.39, 14.29, 14.27, 14.25, 14.22. *Mixture of two* β-*diastereomers is reported*.

***R*$_f$** = 0.27 (*n*-hexane/CH_2Cl_2/ethyl acetate 5:2:2)

HRMS (ESI): calcd for $C_{17}H_{25}NO_4SNa$ $[M + Na]^+$: 362.1402, found: 362.1401.

Compound **3w** (ethyl (2*R*,3*S*)-2-(((*R*)-mesitylsulfinyl)amino)-3-methoxy-3-phenylpropanoate)

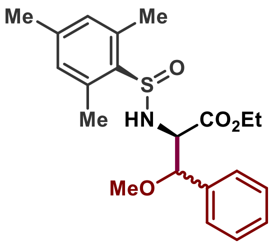

Synthesized from *O*-methyl-D-mandelic acid according to the general procedure described in **Section 4** on 0.15 mmol scale and 3 h reaction time (2 h reaction time for NMR yield). The crude product was purified by preparative TLC using chloroform/ethyl acetate 5:1 as eluent. The product is a colorless oil (35.1 mg, **60% yield**, >95:5 α dr, 1.4:1 β dr).

^{1}H NMR (500 MHz, $CDCl_3$) δ 7.39–7.24 (m, 4H), 7.24–7.18 (m, 1H), 6.80 (s, 2H), 5.26 (d, *J* = 8.8 Hz, 1H, *minor* β-*diastereomer*), 4.95 (d, *J* = 9.4 Hz, 1H), 4.72 (d, *J* = 3.4 Hz, 1H, *minor* β-*diastereomer*), 4.37 (d, *J* = 6.8 Hz, 1H), 4.34–4.26 (m, 1H, *minor* β-*diastereomer*), 4.26–4.16 (m, 2H), 4.16–4.08 (m, 1H), 3.24 (s, 3H), 3.23 (s, 3H, *minor* β-*diastereomer*), 2.36 (s, 3H), 2.33 (s, 3H), 2.26 (s, 3H), 1.28 (t, *J* = 7.1 Hz, 3H, *minor* β-*diastereomer*), 1.25 (t, *J* = 7.1 Hz, 3H). *Mixture of two* β-*diastereomers is reported*.

^{13}C NMR (126 MHz, $CDCl_3$) δ 171.62, 171.03, 140.84, 140.79, 138.19, 137.90, 137.50, 137.43, 137.34, 136.87, 136.82, 131.02, 130.97, 130.92, 130.77, 130.68, 128.48, 128.42, 128.39, 128.37, 128.20, 128.13, 127.75, 127.38, 127.25, 127.09, 84.77, 84.03, 63.40, 62.62, 62.04, 61.83, 57.74, 57.29, 21.14, 19.40, 19.19, 19.15, 14.28, 14.17. *Mixture of two* β-*diastereomers is reported*.

***R*$_f$** = 0.45 & 0.61 for two β-diastereomers (chloroform/ethyl acetate 5:1)

HRMS (ESI): calcd for $C_{21}H_{27}NO_4SNa$ $[M + Na]^+$: 412.1558, found: 412.1552.

Compound **3x** (ethyl *O*-(4-(4-cyano-2-fluorophenoxy)phenyl)-*N*-((*R*)-mesitylsulfinyl)-*D*-threoninate)

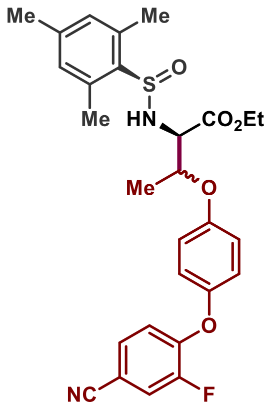

Synthesized from cyhalofop according to the general procedure described in **Section 4** on 0.2 mmol scale and 1.5 h reaction time (2 h reaction time for NMR yield). The crude product was purified by column chromatography with gradient *n*-hexane/CH_2Cl_2/ethyl acetate 8:1:1 to 2:1:1 as eluent. Two sets of fractions were combined and concentrated, resulting in pure β-diastereomer **3x-1** (29.1 mg, >95:5 α dr, >95:5 β dr) and β-diastereomer **3x-2** (78.6 mg, >95:5 α dr, ≈95:5 β dr). The products are colorless oil (**3x-1**) and white crystalline solid (**3x-2**) (in total 89.6 mg, **85% yield**, >95:5 α dr, 3:8 β dr).

^{1}H NMR for β-diastereomer **3x-1** (500 MHz, $CDCl_3$) δ 7.45 (dd, *J* = 10.2, 2.0 Hz, 1H), 7.34 (dt, *J* = 8.6, 1.6 Hz, 1H), 7.04–6.98 (m, 2H), 6.98–6.92 (m, 2H), 6.92–6.86 (m, 3H), 5.23 (d, *J* = 7.8 Hz, 1H), 4.69 (qd, *J* = 6.2, 4.1 Hz, 1H), 4.37 (dd, *J* = 7.8, 4.2 Hz, 1H), 4.34–4.22 (m, 2H), 2.61 (s, 6H), 2.30 (s, 3H), 1.32 (t, *J* = 7.1 Hz, 3H), 1.29 (d, *J* = 6.3 Hz, 3H).

^{13}C NMR for β-diastereomer **3x-1** (126 MHz, $CDCl_3$) δ 170.97, 154.51, 152.50 (d, *J* = 252.0 Hz), 150.62 (d, *J* = 10.7 Hz), 148.67, 141.18, 137.87, 136.89, 130.99, 129.49 (d, *J* = 3.8 Hz), 121.33, 120.69 (d, *J* = 21.3 Hz), 118.70 (d, *J* = 2.0 Hz), 117.79 (d, *J* = 2.6 Hz), 117.62, 106.16 (d, *J* = 8.0 Hz), 75.43, 62.43, 59.89, 21.17, 19.44, 15.44, 14.31.

^{19}F NMR for β-diastereomer **3x-1** (377 MHz, $CDCl_3$) δ −130.03.

^{1}H NMR for β-diastereomer **3x-2** (500 MHz, $CDCl_3$, contains trace amounts of diastereomer **3x-1**) δ 7.44 (dd, *J* = 10.2, 2.0 Hz, 1H), 7.33 (dt, *J* = 8.5, 1.6 Hz, 1H), 6.98–6.93 (m, 2H), 6.93–6.82 (m, 5H), 5.37 (d, *J* = 8.9 Hz, 1H), 4.83 (qd, *J* = 6.2, 3.0 Hz, 1H), 4.23–4.13 (m, 2H), 4.11 (dd, *J* = 8.9, 3.0 Hz, 1H), 2.64 (s, 6H), 2.30 (s, 3H), 1.42 (d, *J* = 6.3 Hz, 3H), 1.18 (t, *J* = 7.1 Hz, 3H).

^{13}C NMR for β-diastereomer **3x-2** (126 MHz, $CDCl_3$) δ 170.91, 154.83, 152.54 (d, *J* = 252.1 Hz), 150.51 (d, *J* = 10.7 Hz), 148.77, 141.26, 137.95, 136.95, 131.01, 129.46 (d, *J* = 4.0 Hz), 121.07, 120.73 (d, *J* = 21.2 Hz), 118.73 (d, *J* = 1.6 Hz), 117.94, 117.76 (d, *J* = 2.3 Hz), 106.26 (d, *J* = 8.0 Hz), 76.04, 62.20, 61.56, 21.20, 19.55, 16.50, 14.22.

^{19}F NMR for β-diastereomer **3x-2** (377 MHz, $CDCl_3$) δ −129.90.

R_f = 0.53 & 0.42 for β-diastereomers **3x-1** and **3x-2**, respectively (*n*-hexane/CH_2Cl_2/ethyl acetate 2:1:1)

HRMS (ESI) for β-diastereomer **3x-1**: calcd for $C_{28}H_{30}FN_2O_5S$ $[M + H]^+$: 525.1859, found: 525.1858.

HRMS (ESI) for β-diastereomer **3x-2**: calcd for $C_{28}H_{30}FN_2O_5S$ $[M + H]^+$: 525.1859, found: 525.1860.

Compound **6x** (ethyl (2*R*)-2-amino-3-(4-(4-cyano-2-fluorophenoxy)phenoxy)butanoate)

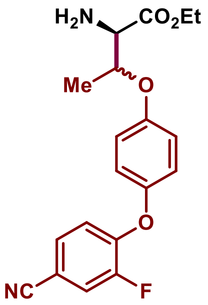

Synthesized from compound **3x-2** according to the *N*-sulfinyl amide deprotection procedure described for compound **3a** in **Section 6** on 0.082 mmol scale. The starting material was dissolved in 3 mL of MeOH upon moderate heating, allowed to reach room temperature, and 0.375 mL of CF_3CO_2H was added dropwise upon vigorous stirring. The crude product was purified by column chromatography with gradient CH_2Cl_2/MeOH 20:1 to 6:1 as eluent. The product is a pale-yellow oil (28.3 mg, **96% yield**, 10:1 β dr).

^{1}H NMR (500 MHz, $CDCl_3$) δ 7.81 (dd, *J* = 11.2, 2.0 Hz, 1H, *minor* β-*diastereomer*), 7.72 (dt, *J* = 8.6, 1.5 Hz, 1H, *minor* β-*diastereomer*), 7.44 (dd, *J* = 10.2, 2.0 Hz, 1H), 7.33 (dt, *J* = 8.7, 1.6 Hz, 1H), 7.01–6.95 (m, 2H), 6.95–6.91 (m, 2H), 6.90–6.83 (m, 1H), 4.77 (qd, *J* = 6.3, 3.6 Hz, 1H), 4.64 (qd, *J* = 6.2, 4.2 Hz, 1H, *minor* β-*diastereomer*), 4.27–4.20 (m, 2H, *minor* β-*diastereomer*), 4.20–4.10 (m, 2H), 3.84 (d, *J* = 4.4 Hz, 1H, *minor* β-*diastereomer*), 3.54 (d, *J* = 3.5 Hz, 1H), 1.78 (s, 2H), 1.41 (d, *J* = 6.3 Hz, 3H), 1.33 (d, *J* = 6.3 Hz, 3H, *minor* β-*diastereomer*), 1.29 (t, *J* = 7.1 Hz, 3H, *minor* β-*diastereomer*), 1.20 (t, *J* = 7.1 Hz, 3H). *Mixture of two* β-*diastereomers is reported*.

^{13}C NMR (126 MHz, $CDCl_3$) δ 173.62, 155.15, 152.49 (d, *J* = 252.0 Hz), 150.66 (d, *J* = 10.5 Hz), 148.52, 129.45 (d, *J* = 3.8 Hz), 121.13, 120.68 (d, *J* = 21.2 Hz), 118.61 (d, *J* = 1.9 Hz), 118.00, 106.11 (d, *J* = 8.2 Hz), 76.27, 61.36, 59.35, 16.56, 14.31. *The poorly resolved minor* β-*diastereomers is not reported.*

^{19}F NMR (377 MHz, $CDCl_3$) δ −130.07, −132.24 (*minor* β-*diastereomer*).

***R*$_f$** = 0.45 (CH_2Cl_2/MeOH 10:1)

HRMS (ESI): calcd for $C_{19}H_{20}FN_2O_4$ $[M + H]^+$: 359.1402, found: 359.1399.

Compound **3y** (ethyl *S*-(4-bromophenyl)-*N*-((*R*)-mesitylsulfinyl)-*D*-cysteinate)

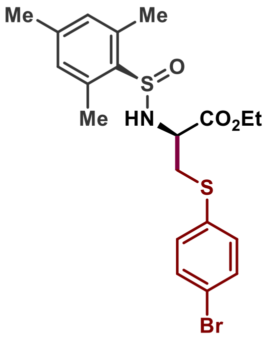

Synthesized from (4-bromo-phenylsulfanyl)acetic acid according to the general procedure described in **Section 4** on 0.2 mmol scale and 1.5 h reaction time (1 h reaction time for NMR yield). The crude product was purified by column chromatography with gradient *n*-hexane/CH_2Cl_2/ethyl acetate 8:1:1 to 1:1:1 as eluent. The product is a pale-orange oil (48.0 mg, **51% yield**, >95:5 α dr).

^{1}H NMR (500 MHz, $CDCl_3$) δ 7.38–7.33 (m, 2H), 7.25–7.21 (m, 2H), 6.86 (s, 2H), 5.20 (d, *J* = 7.9 Hz, 1H), 4.23–4.08 (m, 3H), 3.36 (dd, *J* = 13.8, 4.7 Hz, 1H), 3.18 (dd, *J* = 13.8, 6.9 Hz, 1H), 2.56 (s, 6H), 2.29 (s, 3H), 1.25 (t, *J* = 7.2 Hz, 3H).

^{13}C NMR (126 MHz, $CDCl_3$) δ 171.33, 141.20, 137.64, 136.81, 134.28, 132.41, 132.20, 130.98, 121.17, 62.36, 56.84, 39.24, 21.19, 19.46, 14.16.

***R*$_f$** = 0.23 (*n*-hexane/CH_2Cl_2/ethyl acetate 4:1:1)

HRMS (ESI): calcd for $C_{20}H_{25}BrNO_3S_2$ [M + H]$^+$: 470.0459, found: 470.0444.

Compound **3z** (ethyl (2*R*)-3-((1*r*,4*R*)-4-isopropylcyclohexane-1-carboxamido)-2-(((*R*)-mesitylsulfinyl)amino)-4-phenylbutanoate)

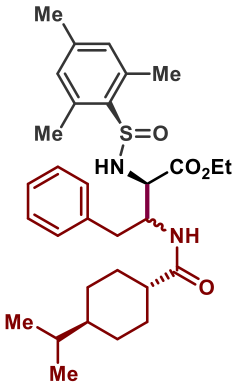

Synthesized from nateglinide according to the general procedure described in **Section 4** on 0.2 mmol scale and 1.5 h reaction time (1 h reaction time for NMR yield). The crude product was purified by column chromatography with gradient *n*-hexane/CH_2Cl_2/ethyl acetate 6:1:1 to 1:1:1 as eluent. The product is a yellow solid (75.4 mg, **70% yield**, >95:5 α dr, 9:10 β dr).

^{1}H NMR (500 MHz, $CDCl_3$) δ 7.34–7.28 (m, 2H), 7.28–7.17 (m, 6H), 7.17–7.11 (m, 2H), 6.94 (s, 2H), 6.88 (s, 2H, *minor* β-*diastereomer*), 5.92 (d, *J* = 8.9 Hz, 1H, *minor* β-*diastereomer*), 5.41 (d, *J* = 9.6 Hz, 1H), 5.37 (d, *J* = 7.3 Hz, 1H), 5.26 (d, J = 8.8 Hz, 1H, *minor* β-*diastereomer*), 4.85–4.76 (m, 1H), 4.72–4.62 (m, 1H, *minor* β-*diastereomer*), 4.16 (qd, *J* = 7.1, 1.2 Hz, 2H), 4.12–4.03 (m, 3H), 4.03–3.95 (m, 1H), 2.99–2.91 (m, 1H), 2.88–2.76 (m, 1H), 2.73 (t, *J* = 6.8 Hz, 2H), 2.66 (s, 6H), 2.58 (s, 6H, *minor* β-*diastereomer*), 2.33 (s, 3H), 2.30 (s, 3H, *minor* β-*diastereomer*), 1.91 (tt, *J* = 12.2, 3.3 Hz, 1H), 1.85–1.78 (m, 2H), 1.78–1.65 (m, 6H), 1.65–1.56 (m, 1H), 1.44–1.17 (m, 6H), 1.26 (t, *J* = 7.2 Hz, 3H), 1.21 (t, *J* = 7.1 Hz, 3H), 1.06–0.86 (m, 6H), 0.84 (d, *J* = 6.8 Hz, 6H), 0.81 (d, *J* = 6.8 Hz, 6H). *Mixture of two* β-*diastereomers is reported*.

^{13}C NMR (126 MHz, $CDCl_3$) δ 175.67, 171.71, 171.12, 141.54, 141.40, 137.88, 137.20, 137.14, 136.88, 136.75, 136.71, 131.23, 131.15, 129.50, 129.36, 128.83, 128.58, 127.03, 126.94, 62.62, 62.25, 59.53, 57.99, 51.75, 51.32, 45.94, 45.78, 43.38, 43.26, 38.49, 36.50, 32.90, 32.88, 29.93, 29.86, 29.63, 29.60, 29.14, 29.05, 29.01, 28.99, 21.24, 21.18, 19.87, 19.85, 19.83, 19.65, 19.59, 14.13. *Mixture of two* β-*diastereomers is reported*.

***R*$_f$** = 0.22 (*n*-hexane/CH_2Cl_2/ethyl acetate 3:1:1)

HRMS (ESI): calcd for $C_{31}H_{44}N_2O_4SNa$ [M + Na]$^+$: 563.2919, found: 563.2914.

Compound **6z** (ethyl (2*R*,3*S*)-2-amino-3-((1*r*,4*S*)-4-isopropylcyclohexane-1-carboxamido)-4-phenylbutanoate)

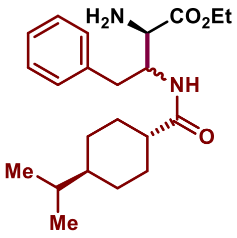

Synthesized from compound **3z** according to the *N*-sulfinyl amide deprotection procedure described for compound **3a** in **Section 6** on 0.122 mmol scale. The starting material was dissolved in 2 mL of MeOH and 0.25 mL of CF_3CO_2H was added dropwise. The crude product was purified by column chromatography with gradient CH_2Cl_2/MeOH 20:1 to 6:1 as eluent. The product is a yellow solid (45.8 mg, **99% yield**, 9:10 β dr).

^{1}H NMR (500 MHz, $CDCl_3$) δ 7.35–7.13 (m, 10H), 6.08 (dd, *J* = 9.4, 4.4 Hz, 2H), 4.71–4.56 (m, 2H), 4.21–4.03 (m, 4H), 3.70 (s, 1H, *minor* β-*diastereomer*), 3.56

(s, 1H), 2.97–2.43 (m, 10H), 2.00–1.86 (m, 2H), 1.86–1.66 (m, 8H), 1.45–1.17 (m, 13H), 1.07–0.85 (m, 7H), 0.84 (s, 3H), 0.83 (s, 3H). *Mixture of two* β-*diastereomers is reported*.

^{13}C NMR (126 MHz, $CDCl_3$) δ 176.29, 175.83, 173.16, 137.63, 137.24, 129.40, 129.28, 128.72, 128.52, 126.79, 126.77, 61.89, 61.69, 56.50, 54.61, 52.09, 51.84, 45.83, 45.76, 43.34, 38.27, 36.47, 32.88, 29.98, 29.87, 29.74, 29.68, 29.10, 29.08, 29.06, 29.02, 19.85, 14.29, 14.24. *Mixture of two* β-*diastereomers is reported*.

R_f = 0.53 (CH_2Cl_2/MeOH 10:1)

HRMS (ESI): calcd for $C_{22}H_{35}N_2O_3$ $[M + H]^+$: 375.2642, found: 375.2639.

10. *NMR spectra*

^{1}H NMR (500 MHz, $CDCl_3$) of compound **1**

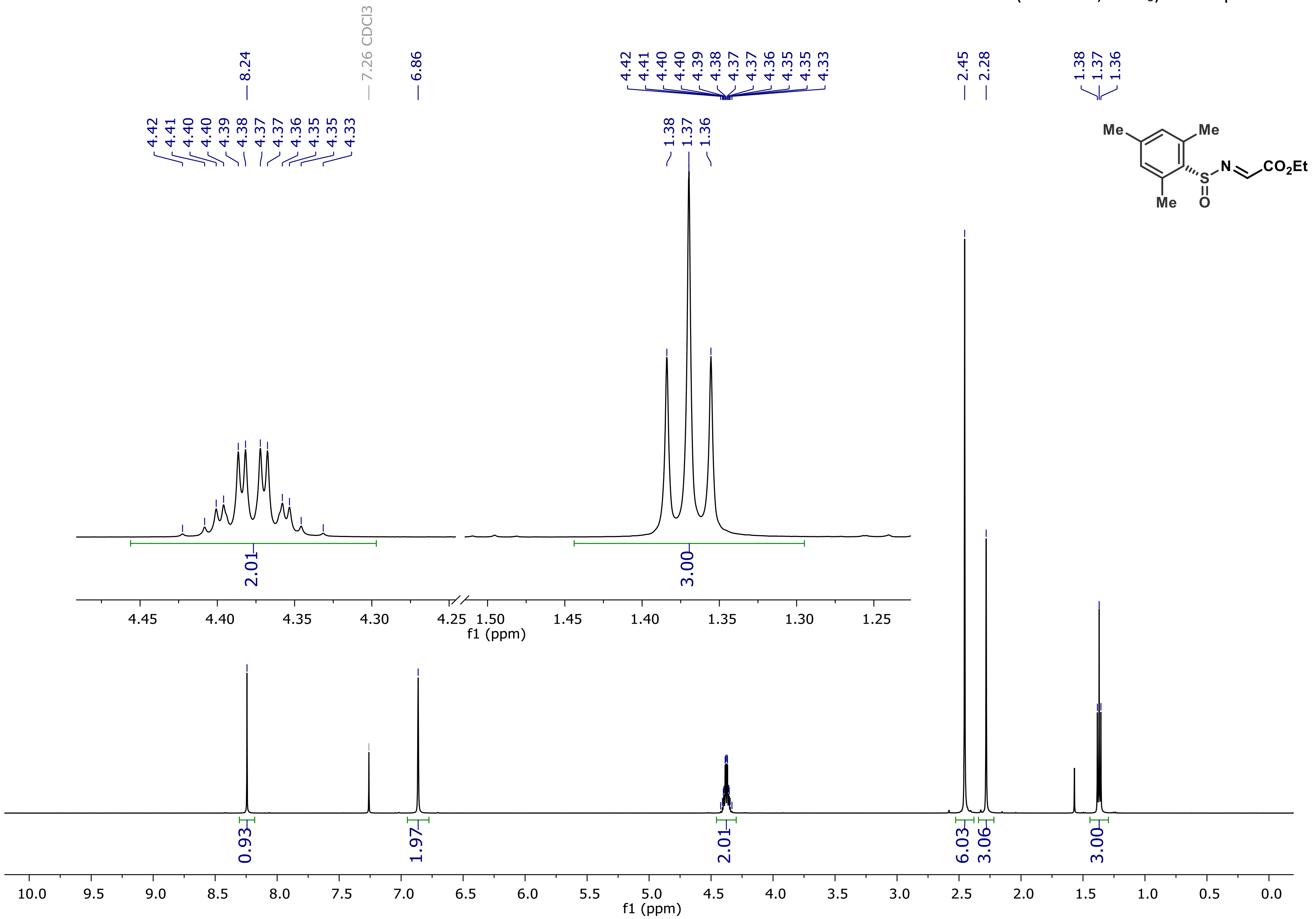

^{13}C NMR (126 MHz, $CDCl_3$) of compound **1**

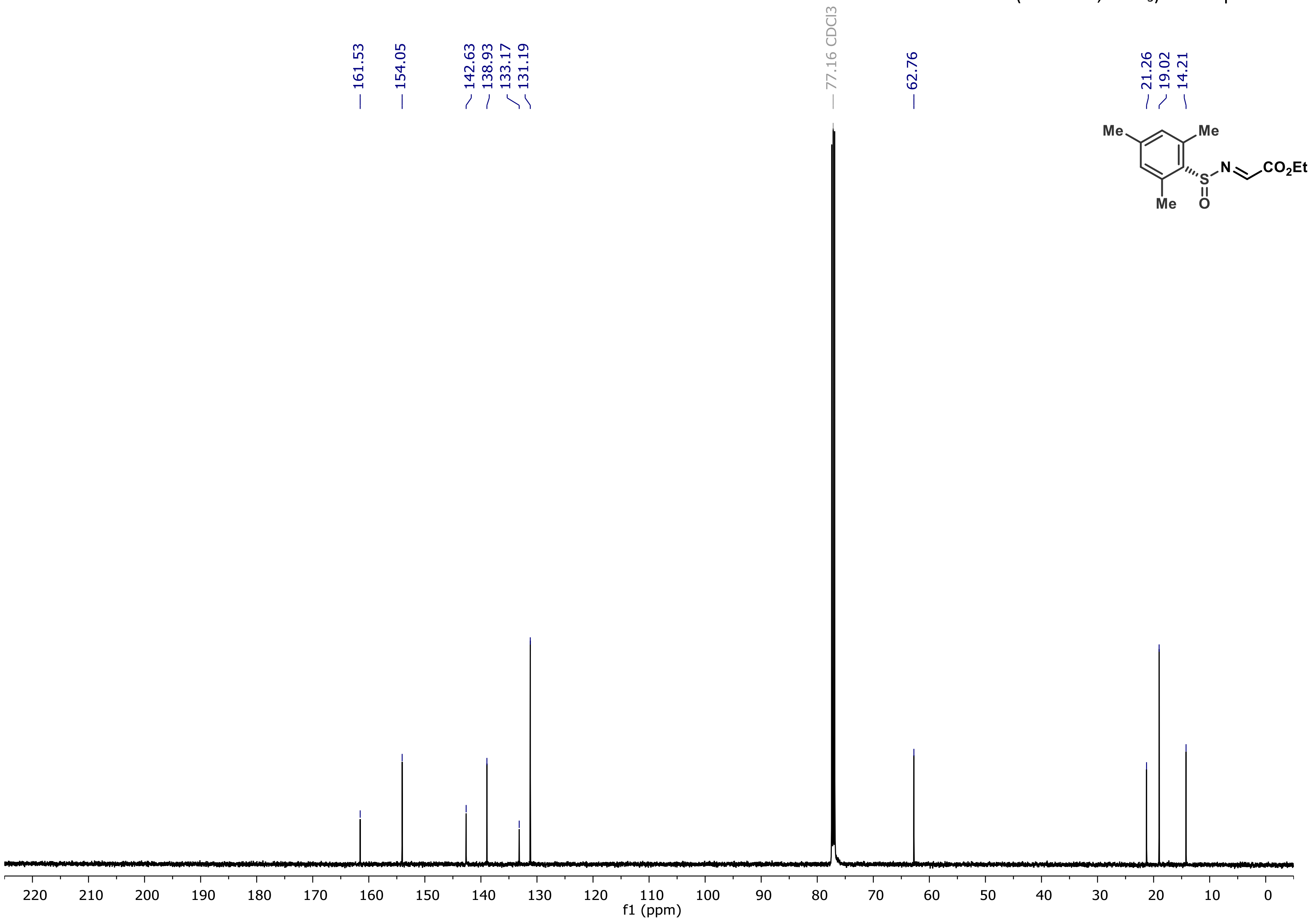

^{1}H NMR (400 MHz, $CDCl_3$) of compound **4**

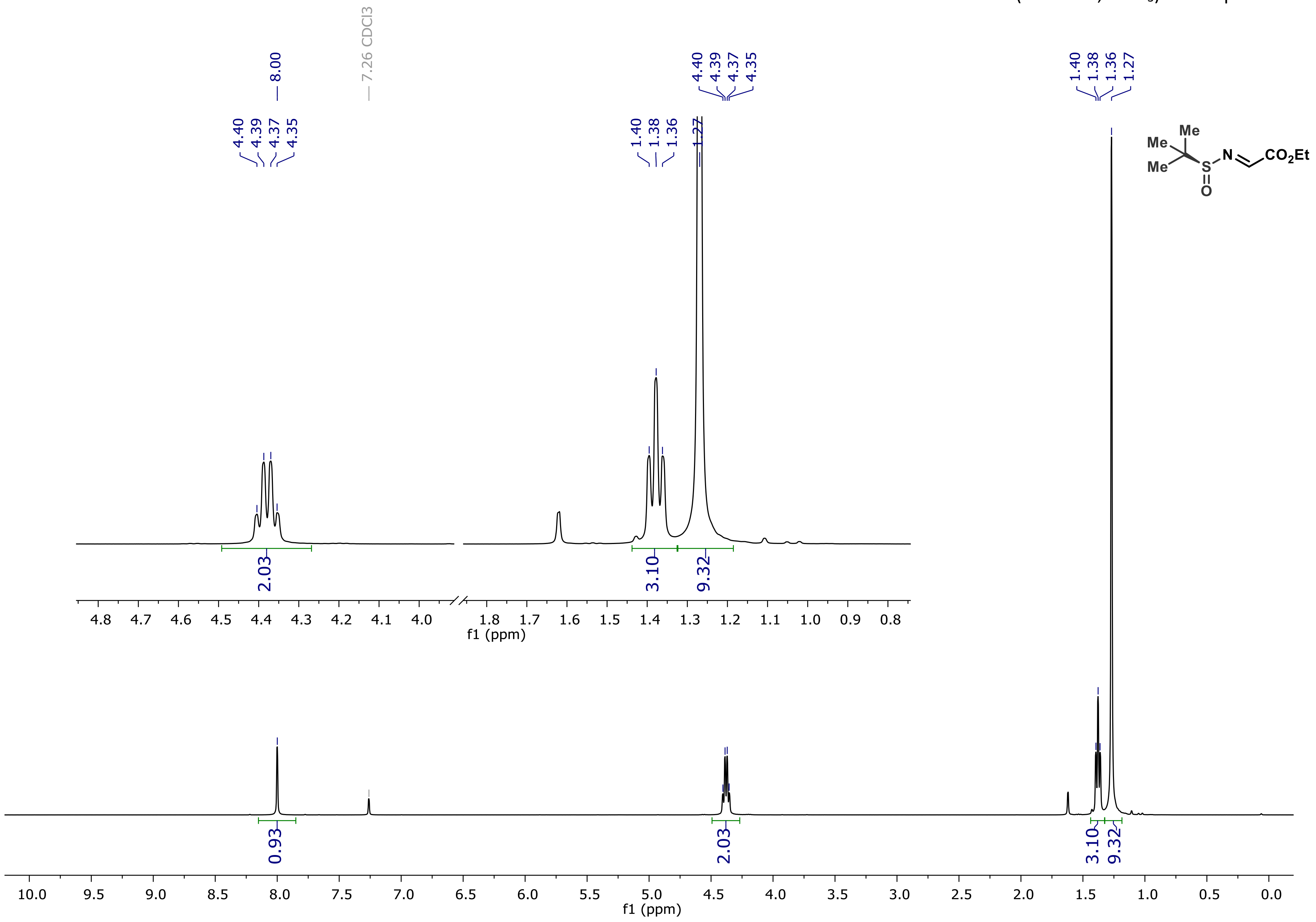

^{13}C NMR (101 MHz, $CDCl_3$) of compound **4**

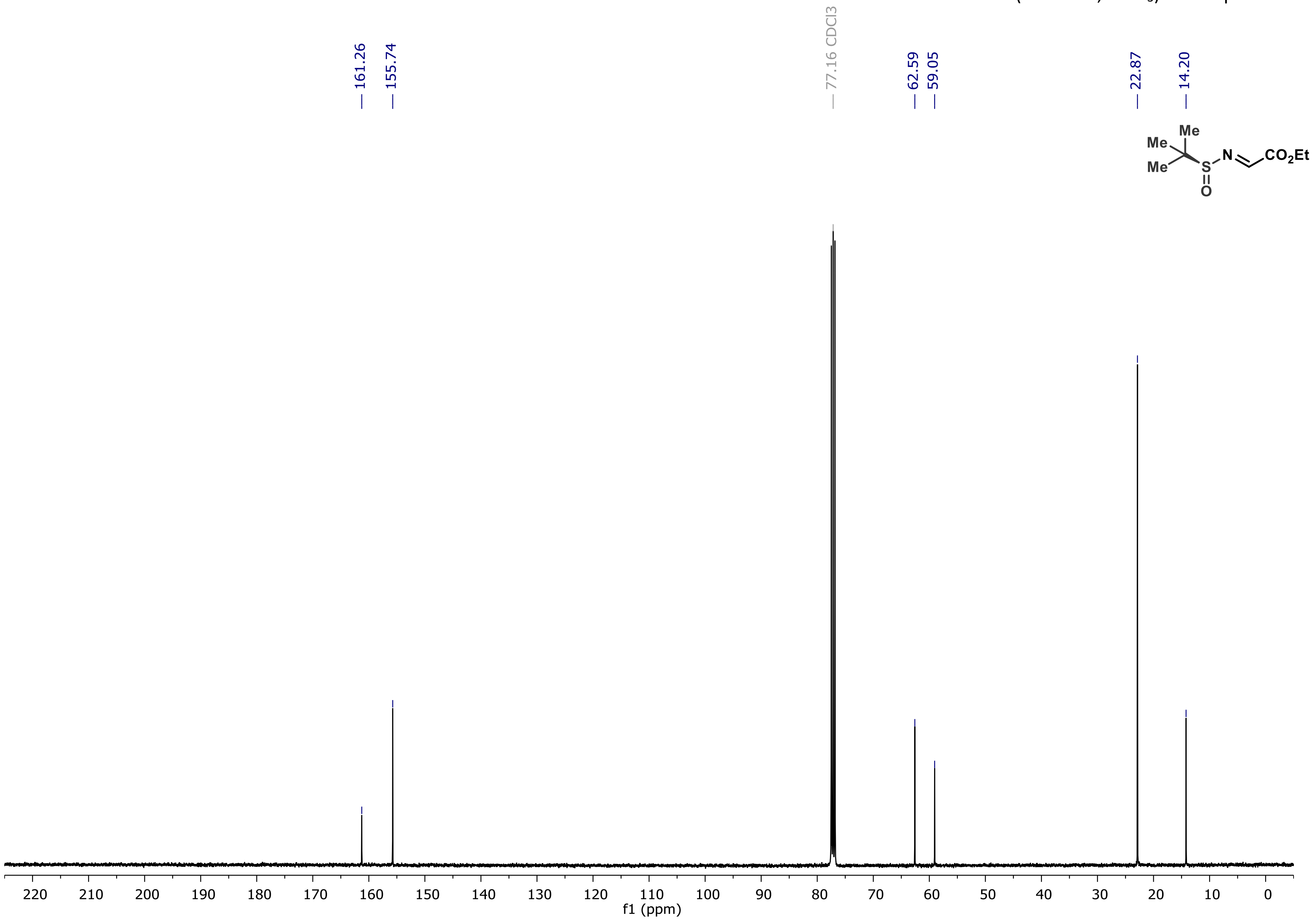

^{1}H NMR (400 MHz, $CDCl_3$) of compound **5**

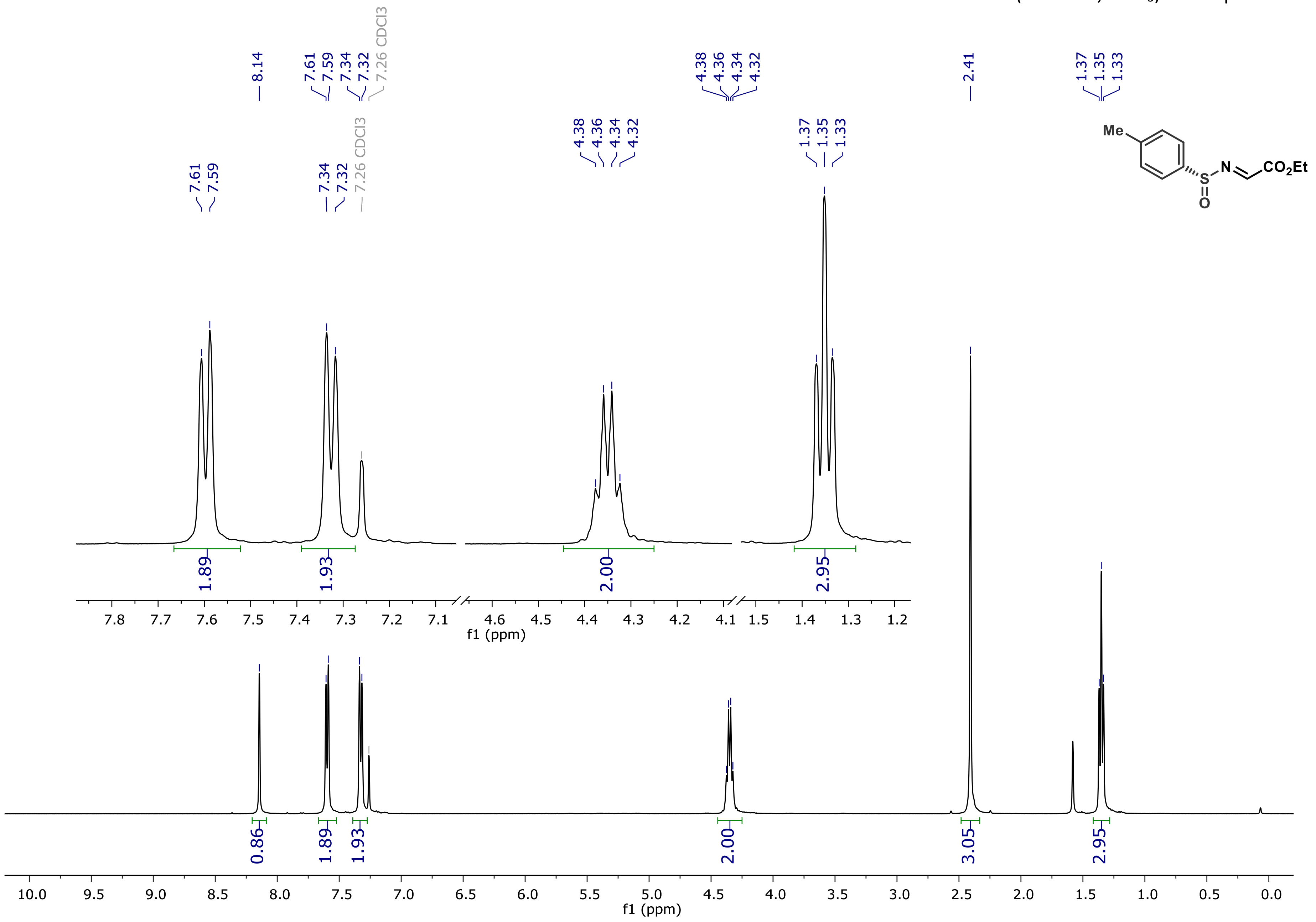

^{13}C NMR (101 MHz, $CDCl_3$) of compound **5**

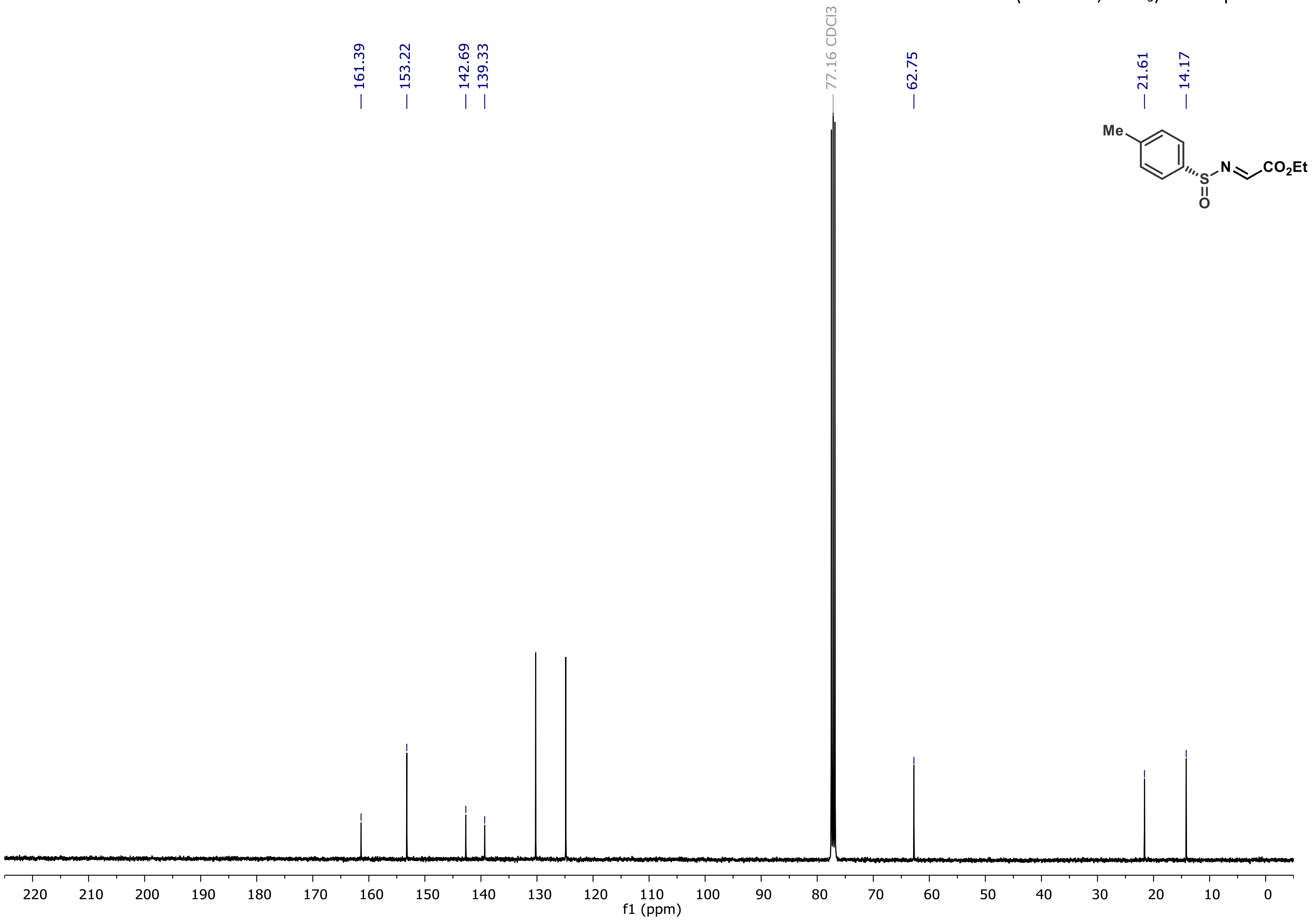

^{1}H NMR (500 MHz, $CDCl_3$) of compound **3a**

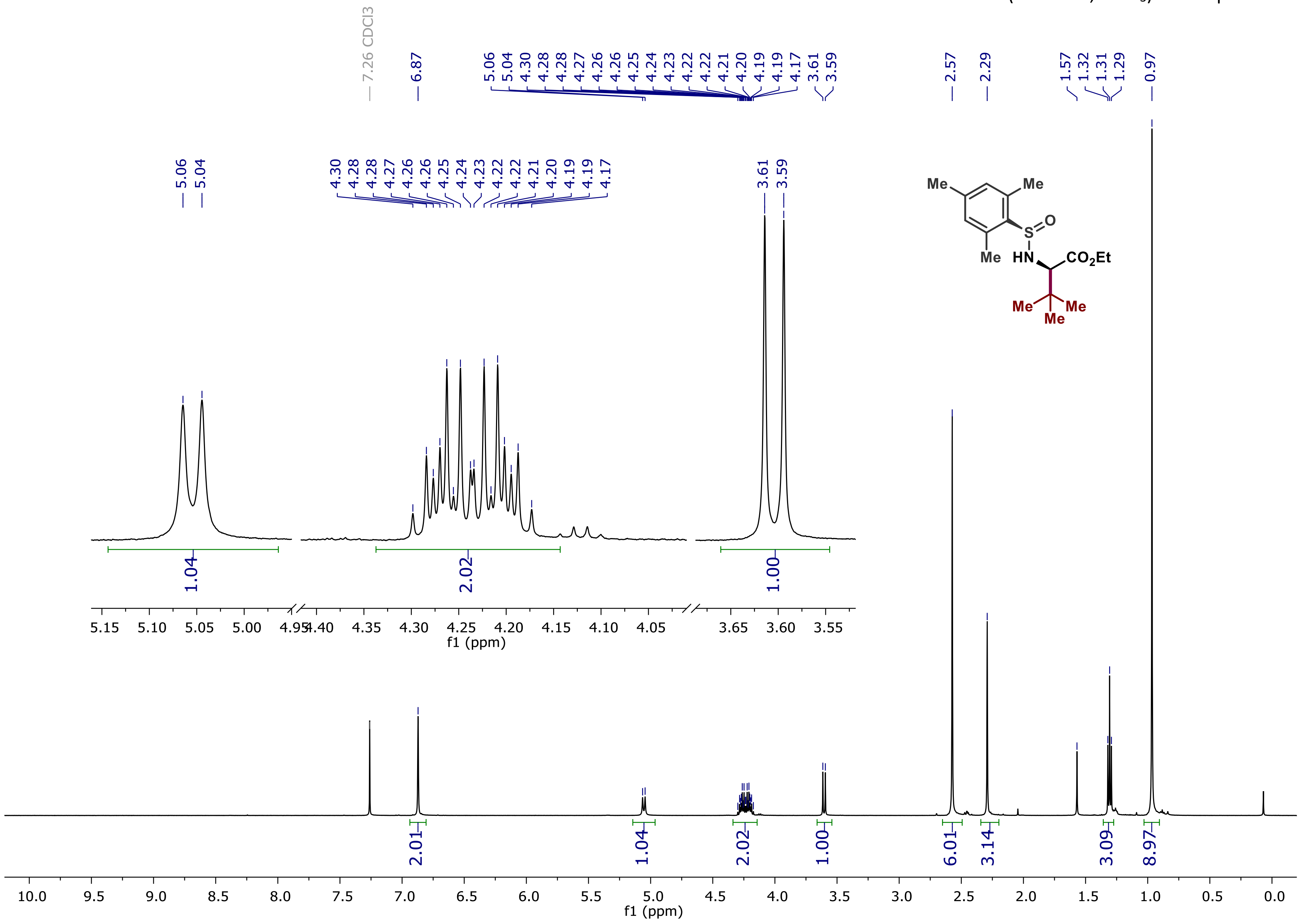

^{13}C NMR (126 MHz, $CDCl_3$) of compound **3a**

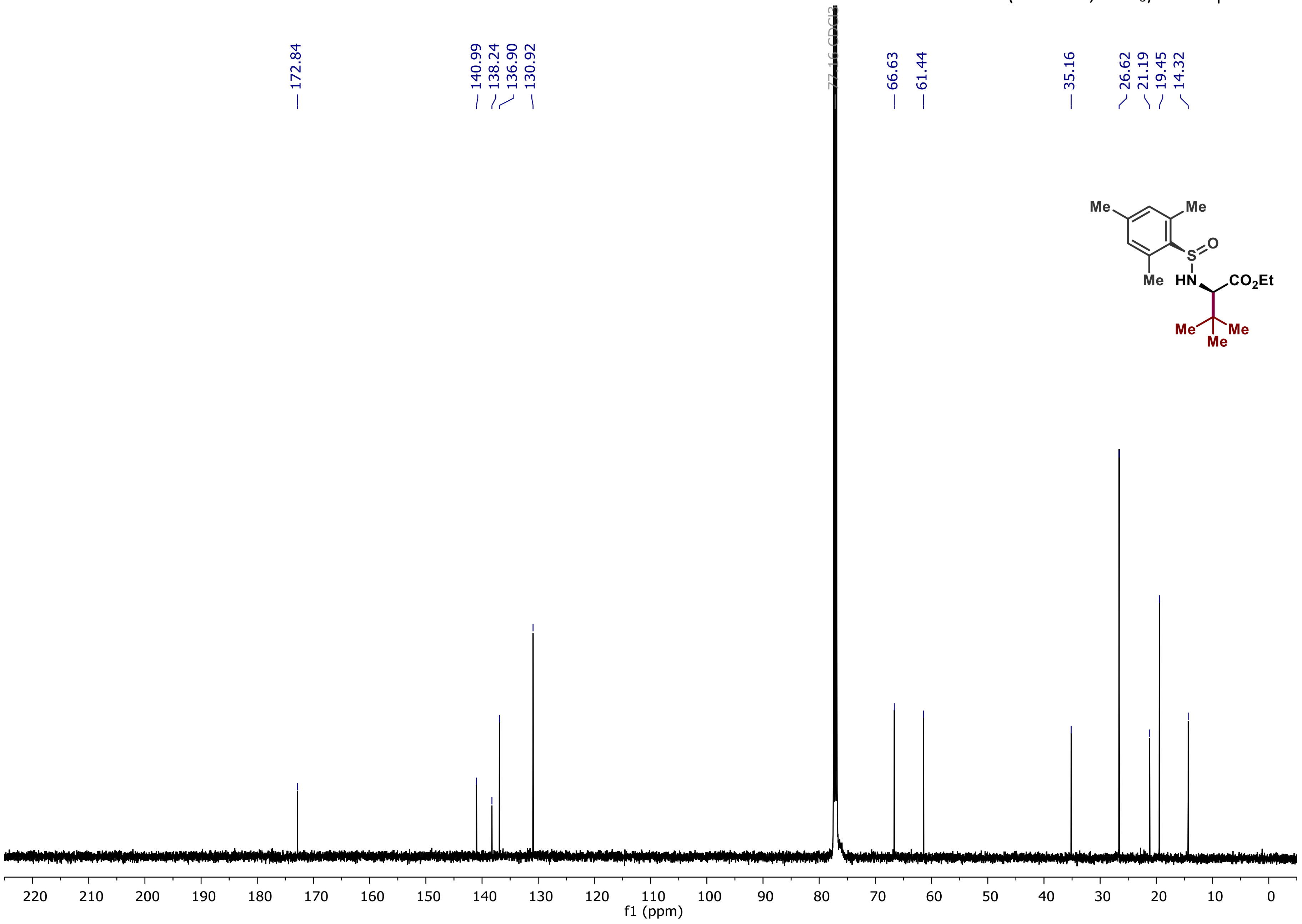

^{1}H NMR (500 MHz, MeOH-d_4) of compound **6a**

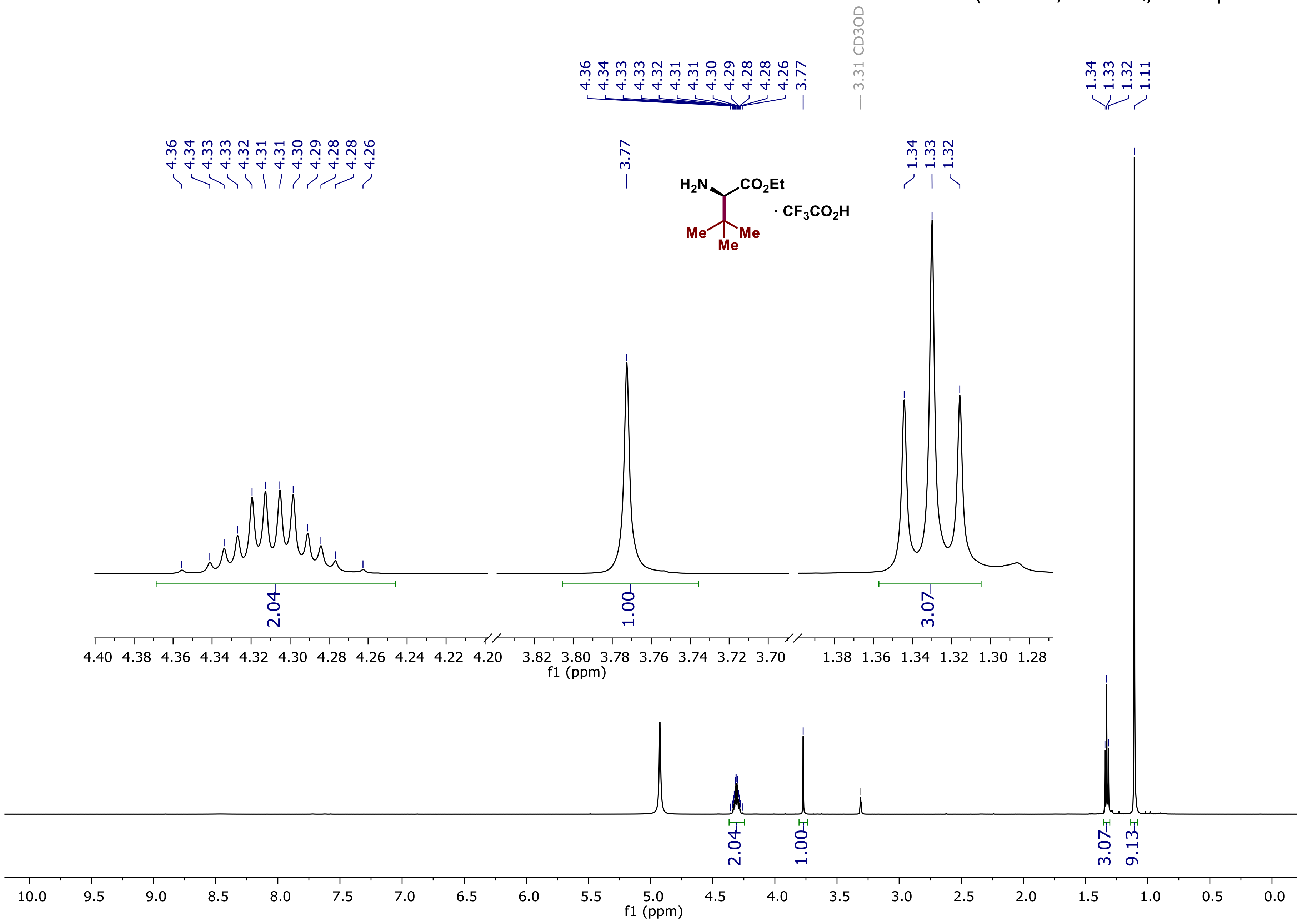

^{13}C NMR (126 MHz, MeOH-d_4) of compound **6a**

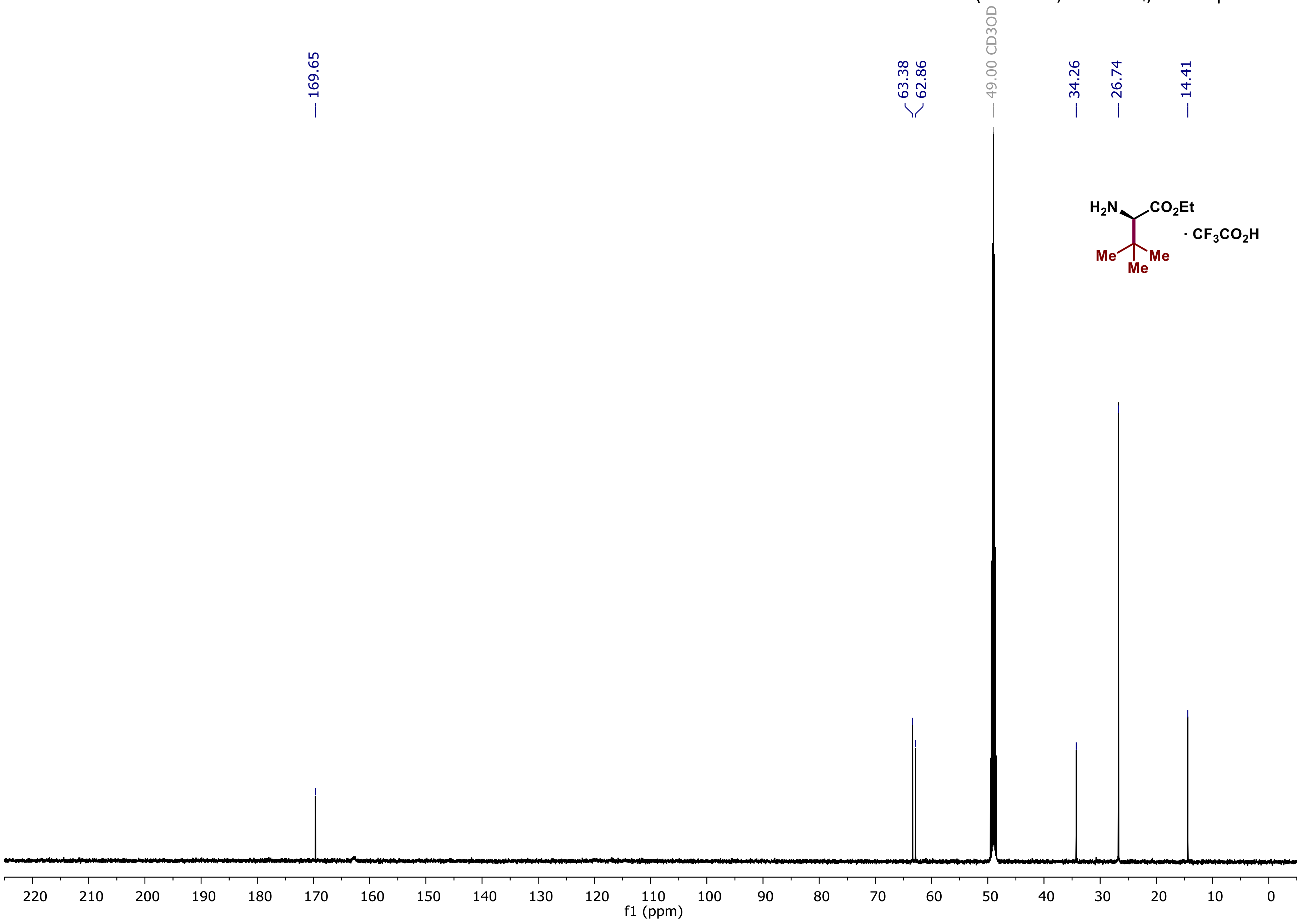

^{19}F NMR (377 MHz, MeOH-d_4) of compound **6a**

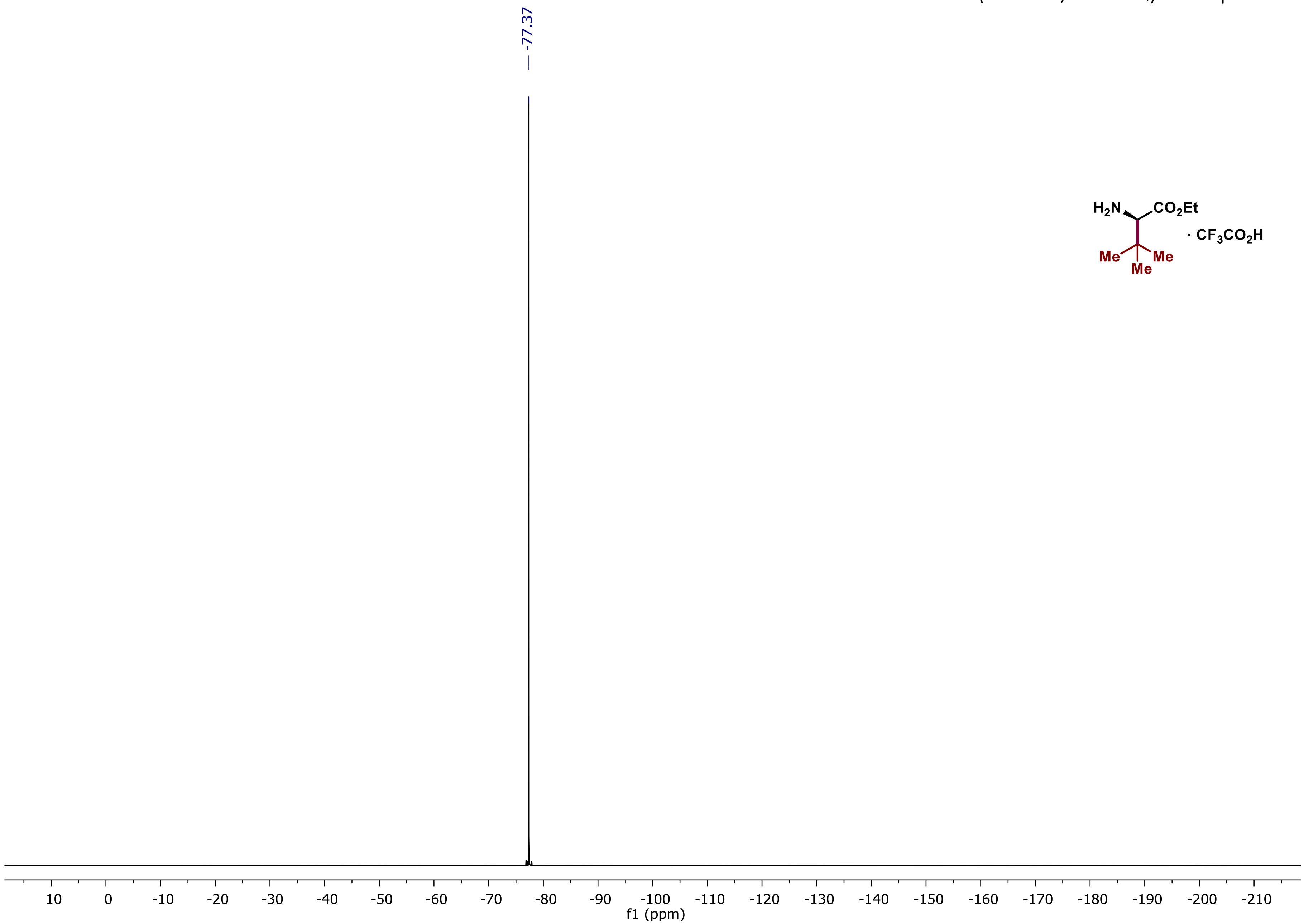

^{1}H NMR (500 MHz, $CDCl_3$) of compound **3b**

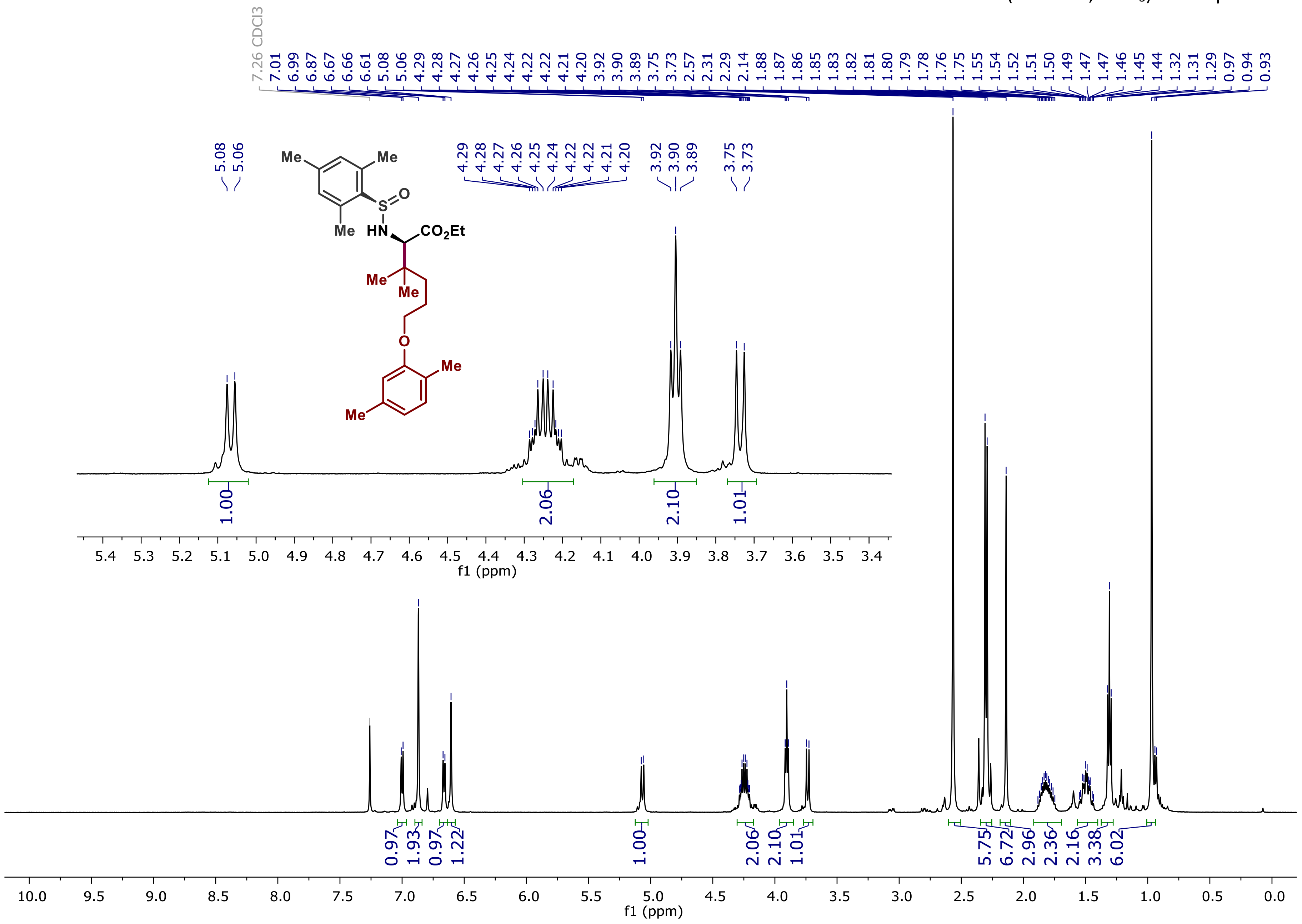

^{13}C NMR (126 MHz, $CDCl_3$) of compound **3b**

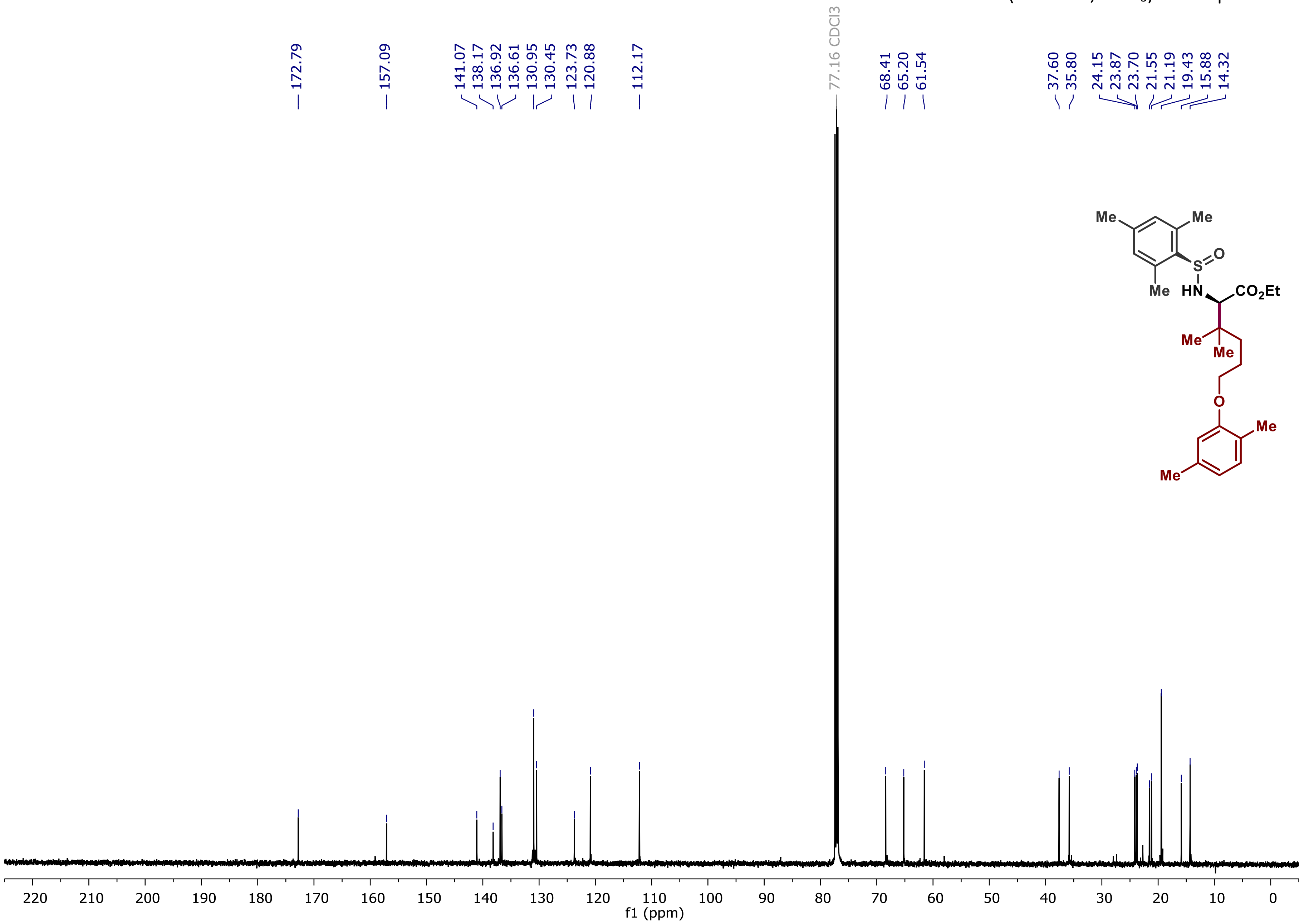

^{1}H NMR (500 MHz, $CDCl_3$) of compound **3c**

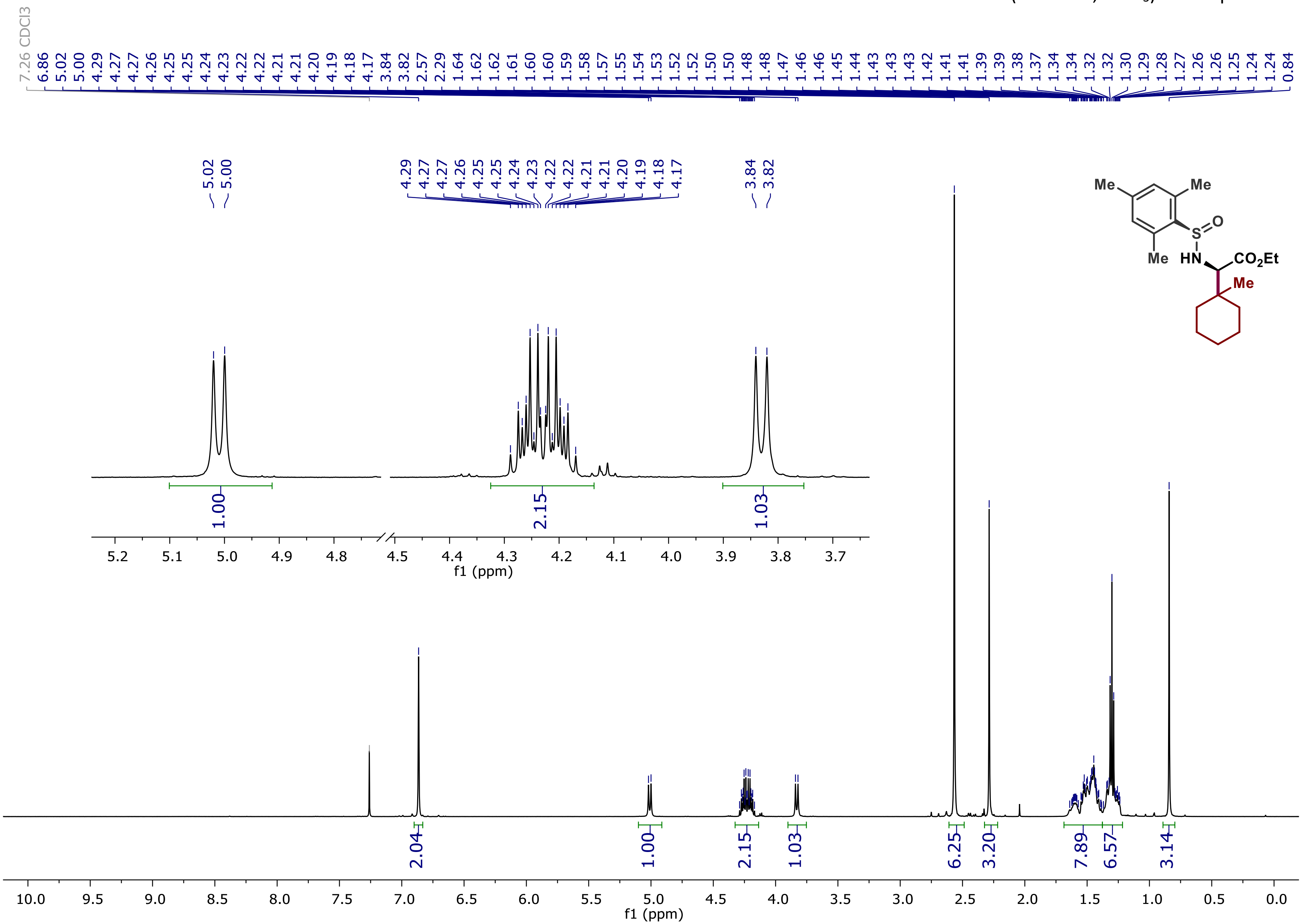

^{13}C NMR (126 MHz, $CDCl_3$) of compound **3c**

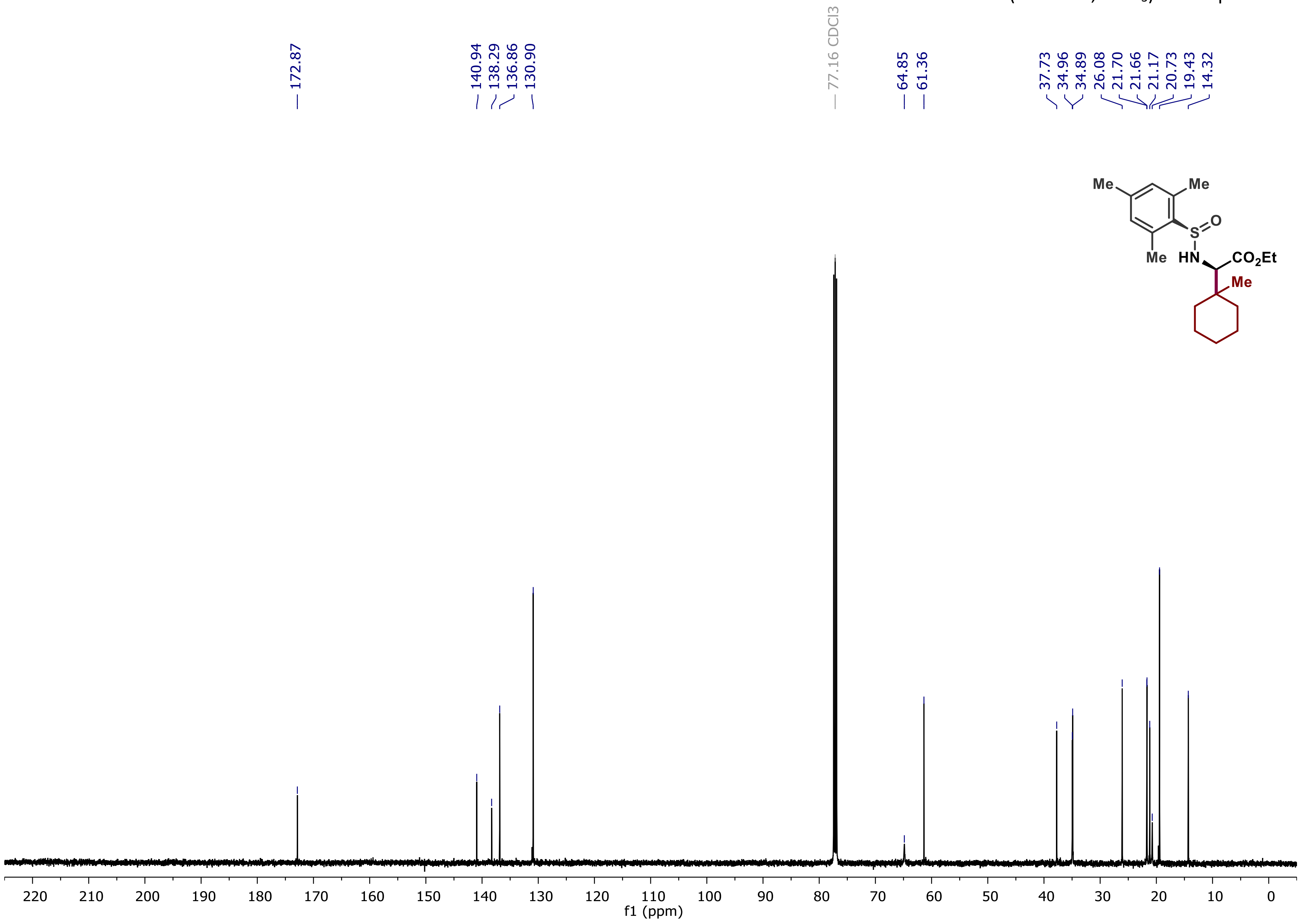

^{1}H NMR (500 MHz, CDCl$_3$) of compound **3d**

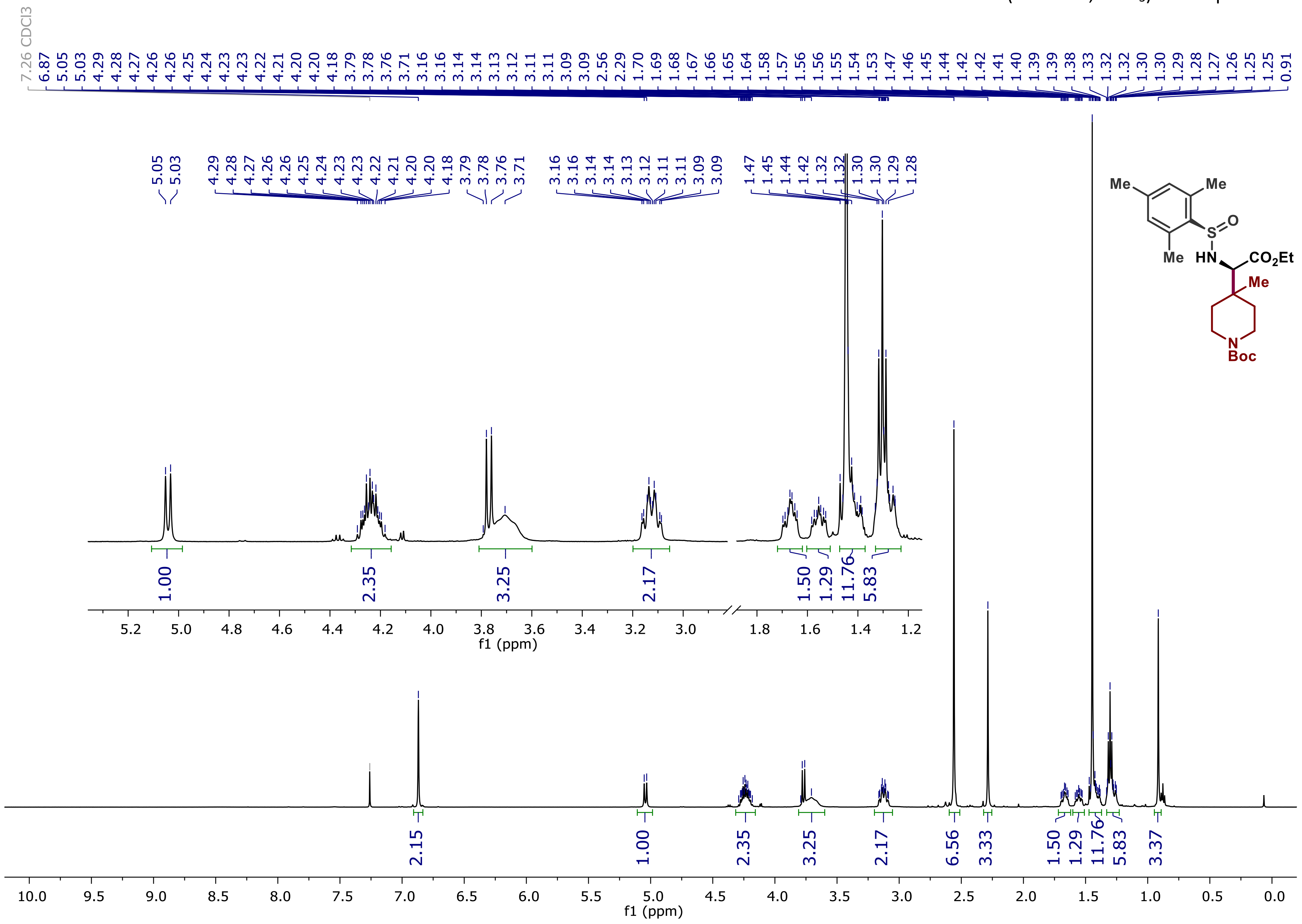

^{13}C NMR (126 MHz, $CDCl_3$) of compound **3d**

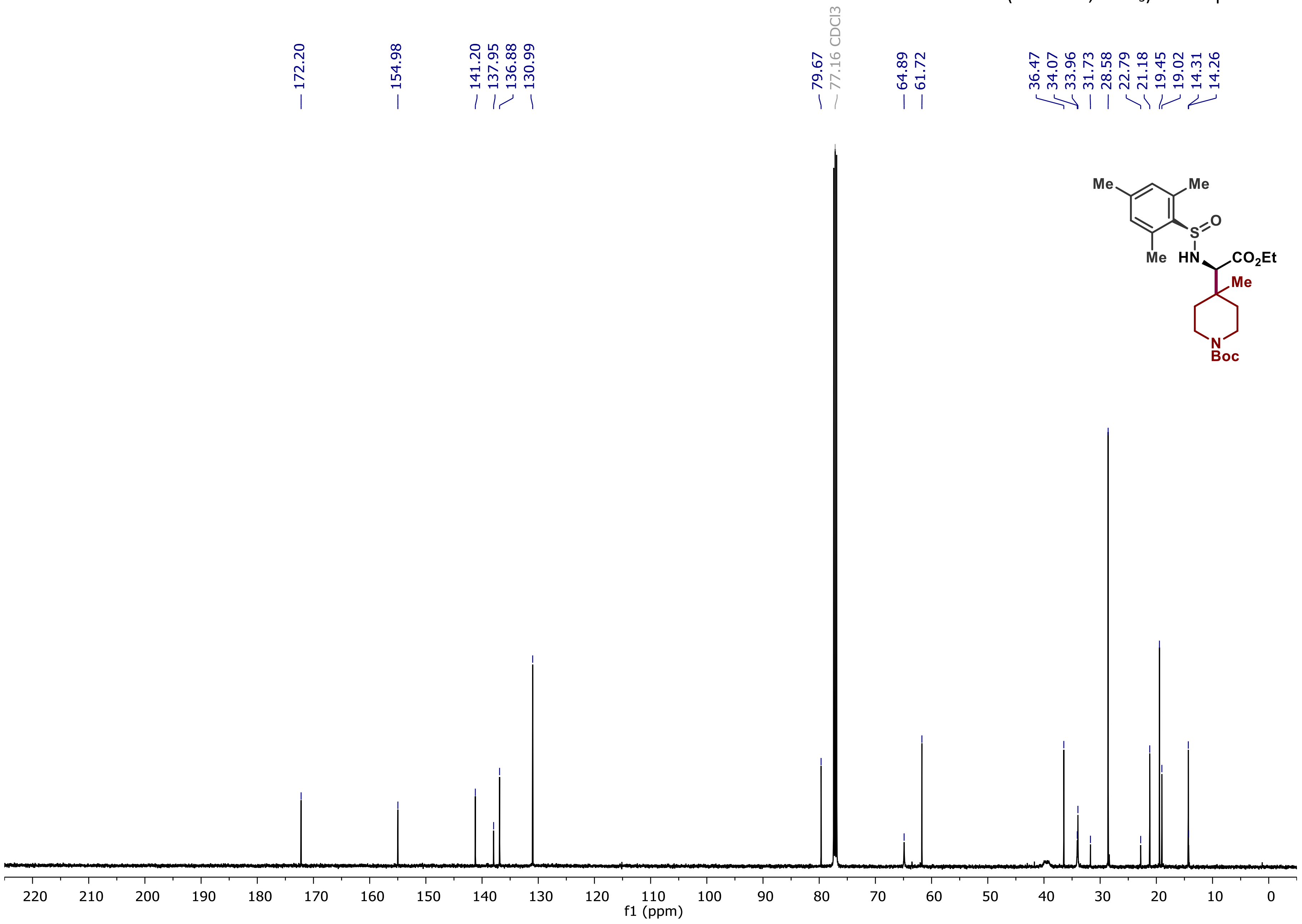

^{1}H NMR (500 MHz, $CDCl_3$) of compound **3e**

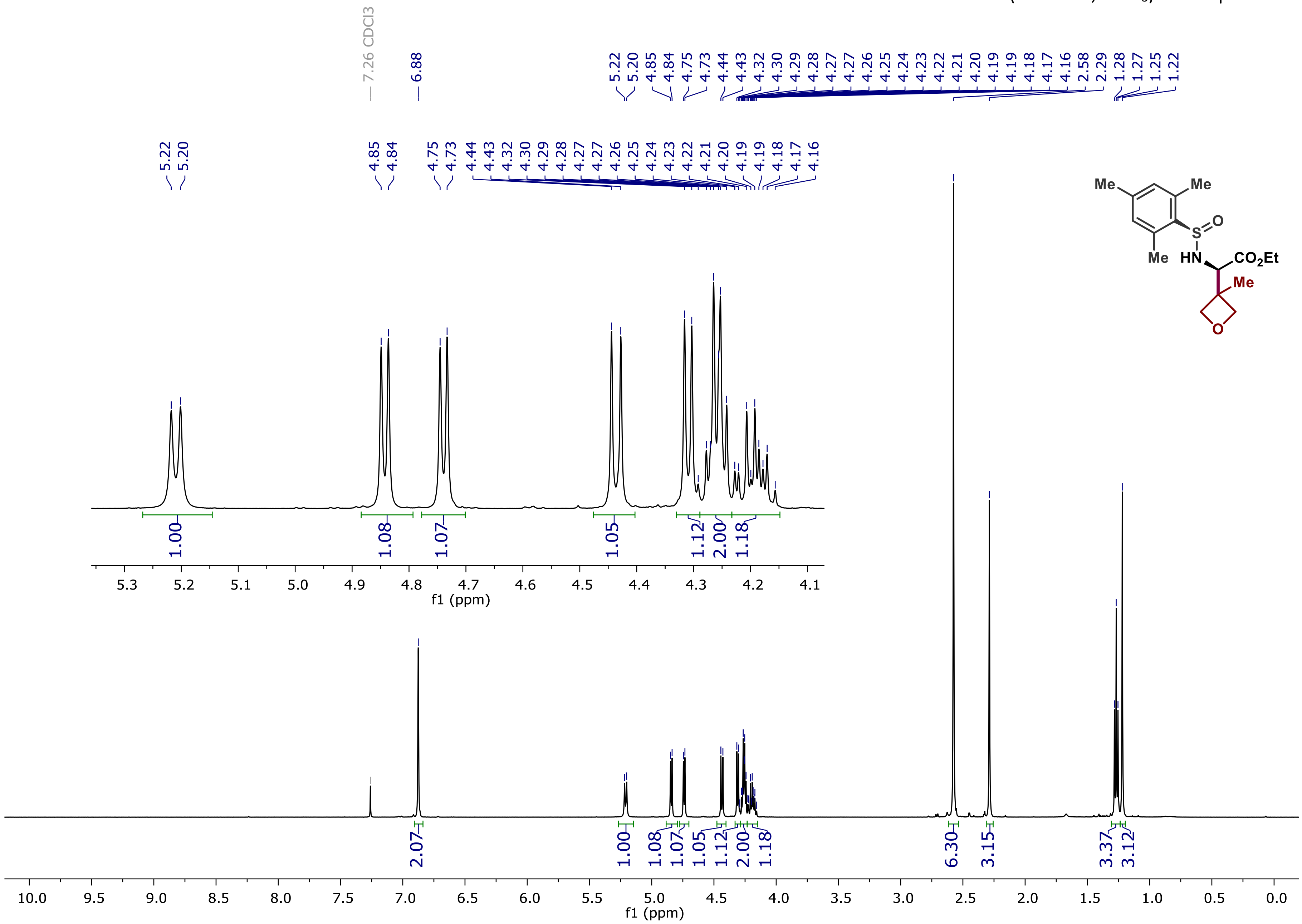

^{13}C NMR (126 MHz, $CDCl_3$) of compound **3e**

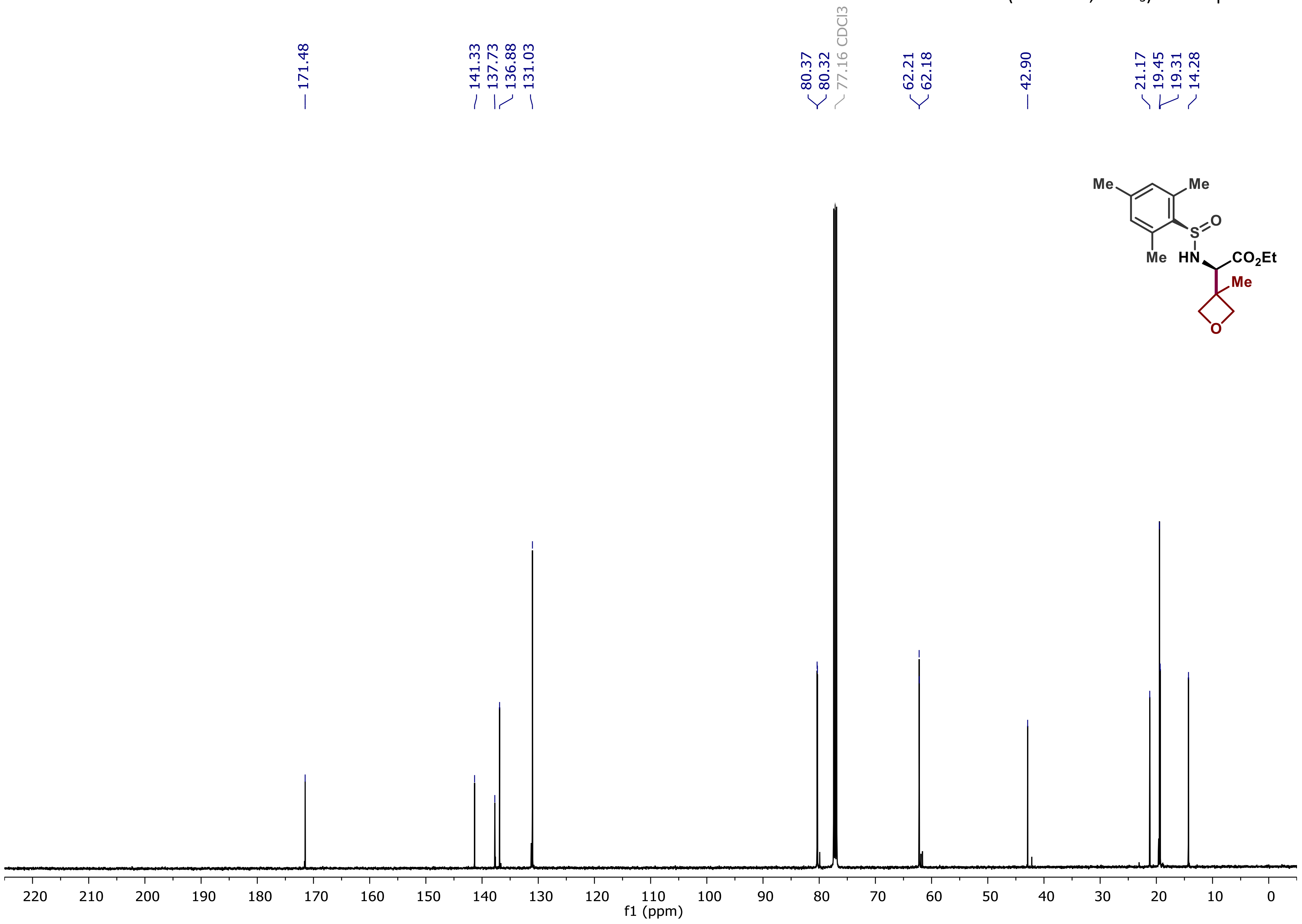

^{1}H NMR (500 MHz, $CDCl_3$) of compound **3f**

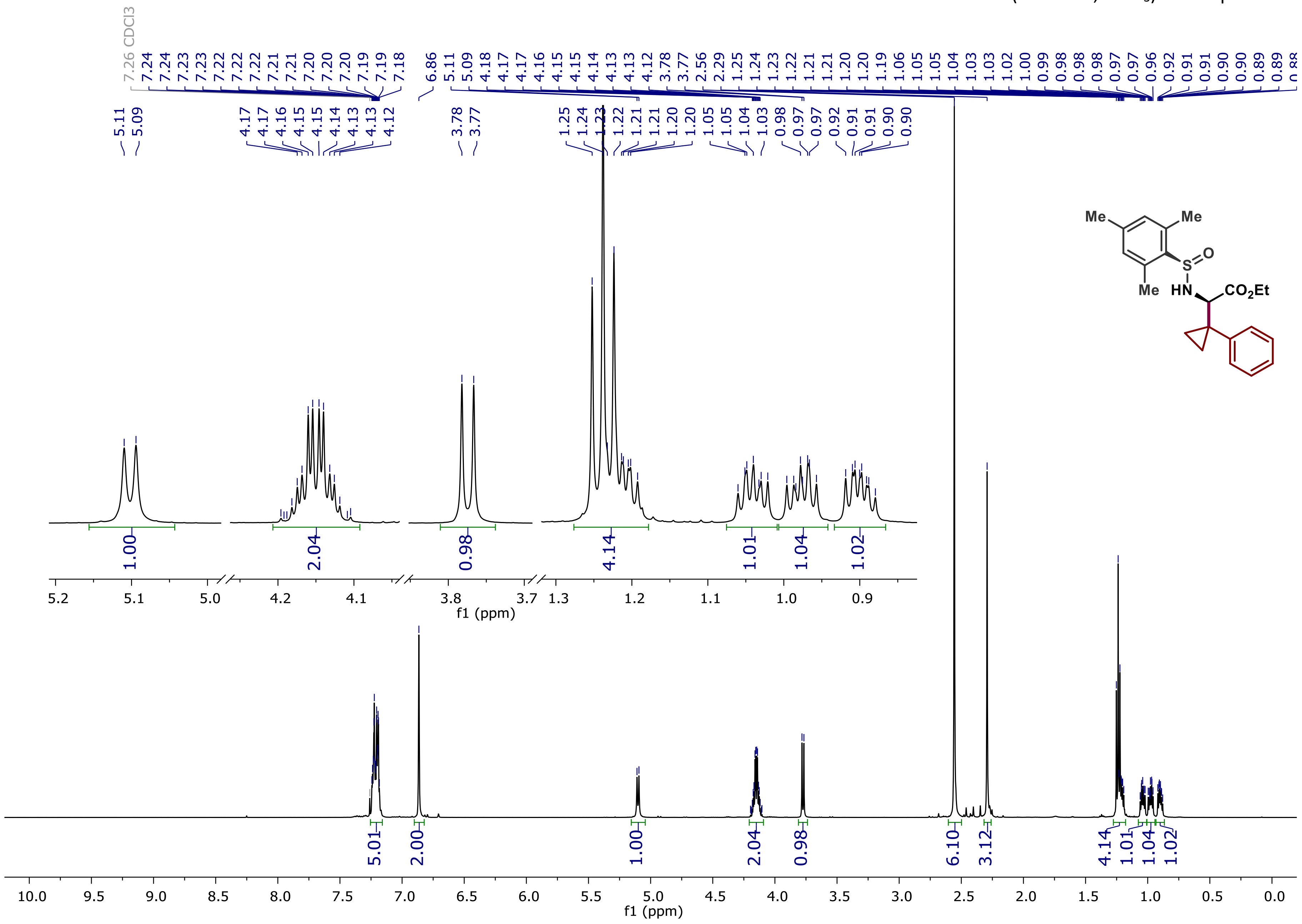

^{1}H NMR (126 MHz, $CDCl_3$) of compound **3f**

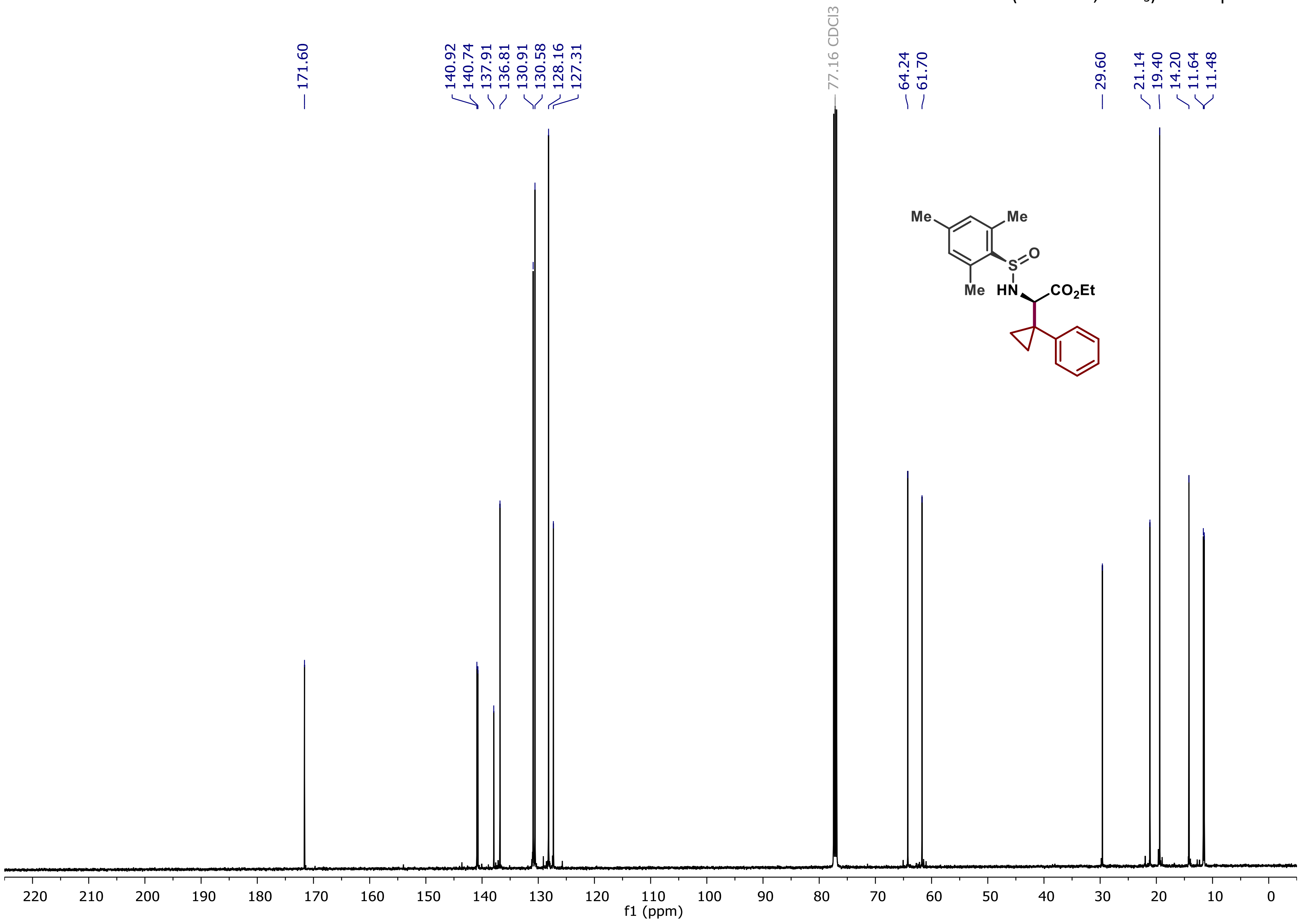

^{1}H NMR (500 MHz, $CDCl_3$) of compound **3g**

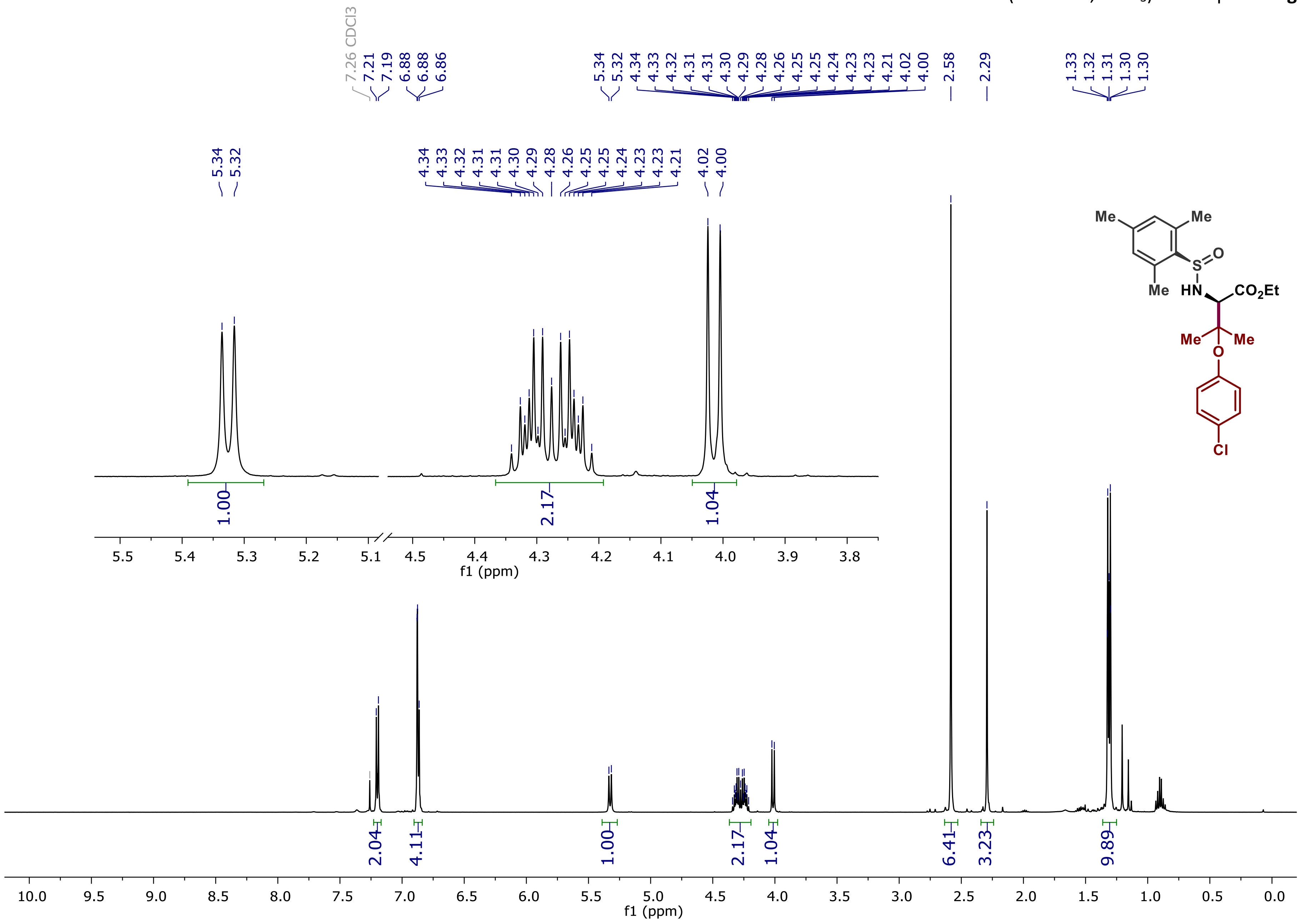

^{13}C NMR (126 MHz, $CDCl_3$) of compound **3g**

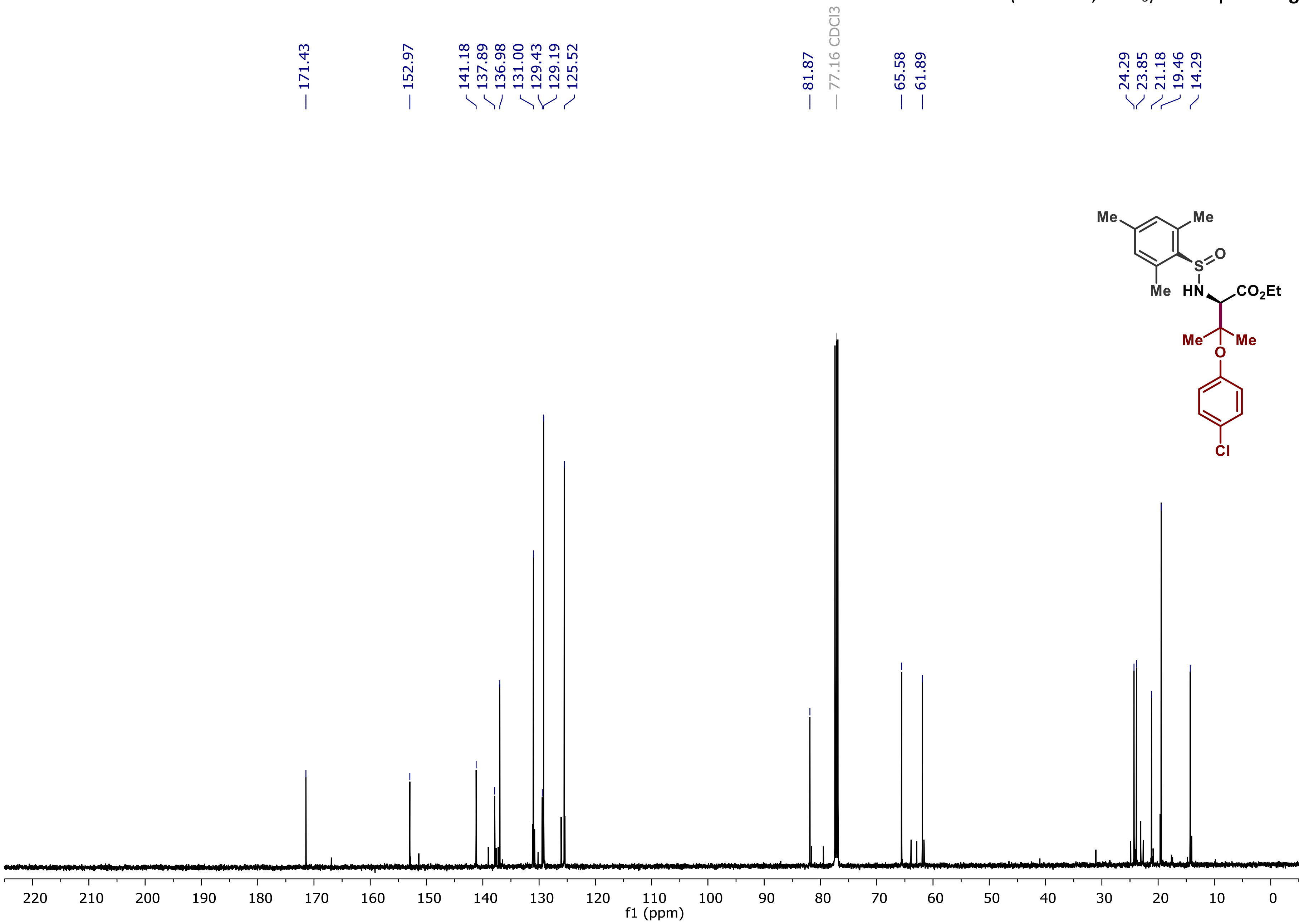

^{1}H NMR (500 MHz, $CDCl_3$) of compound **3h**

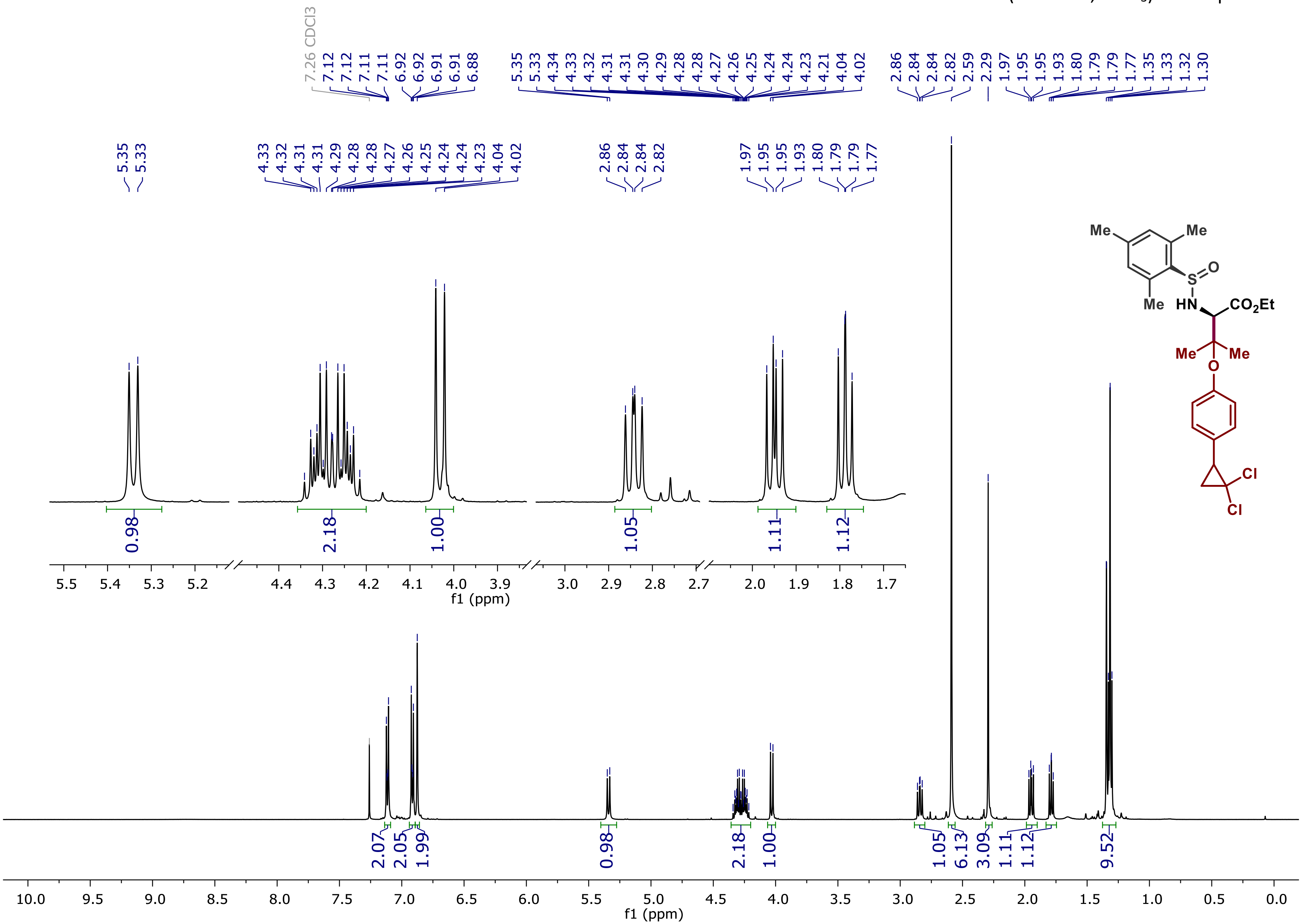

^{13}C NMR (126 MHz, $CDCl_3$) of compound **3h**

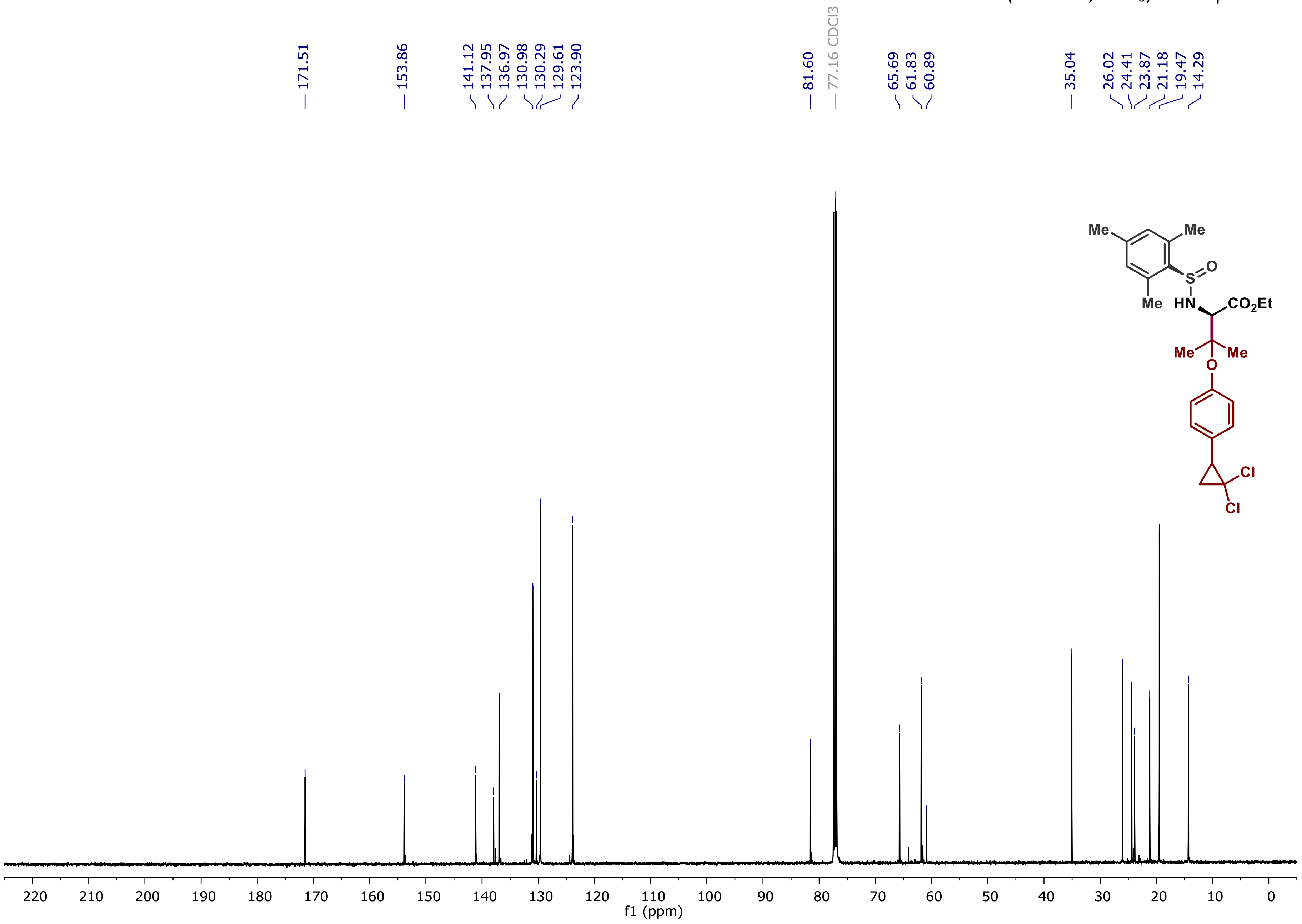

^{1}H NMR (500 MHz, $CDCl_3$) of compound **3i**

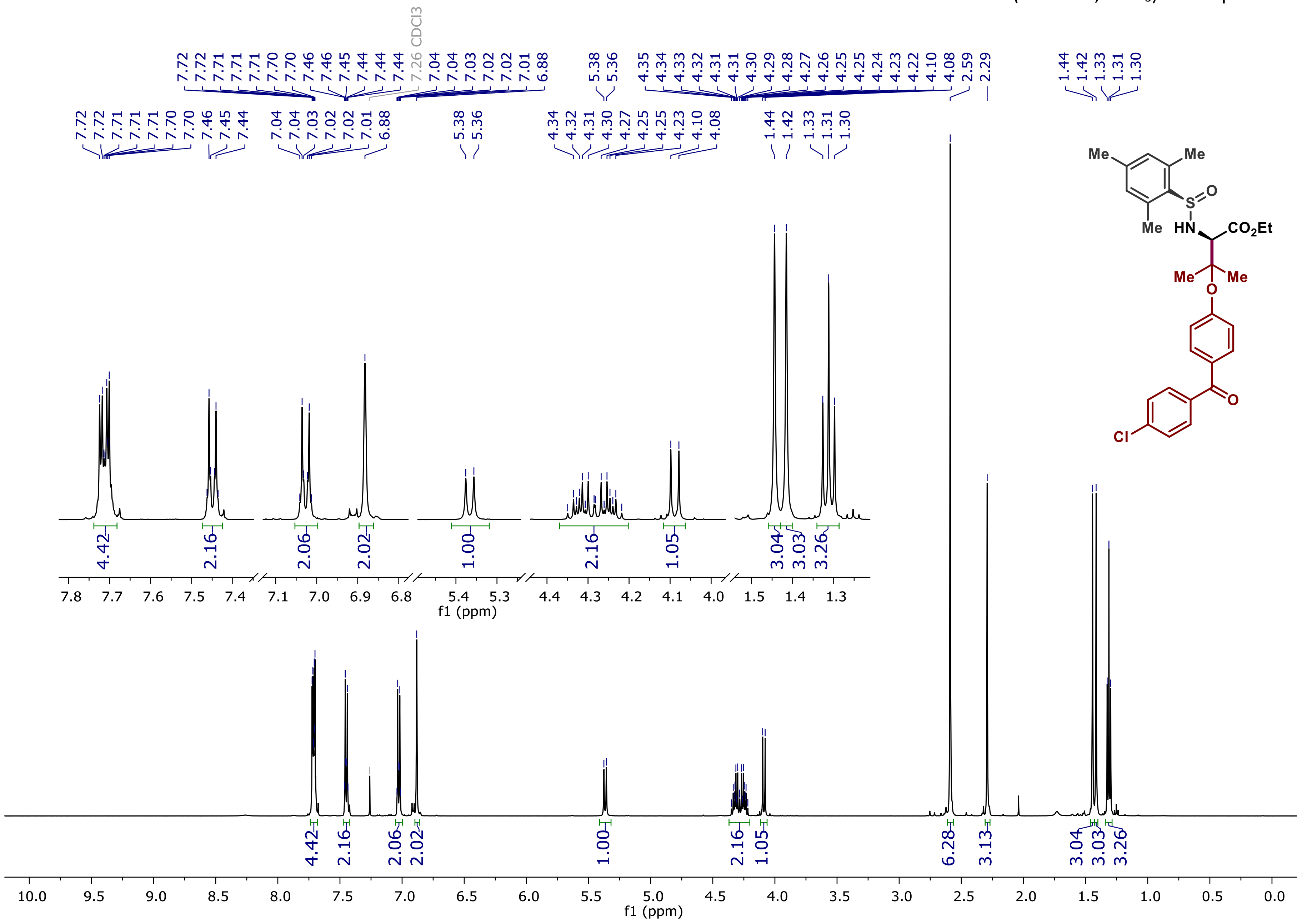

^{13}C NMR (126 MHz, $CDCl_3$) of compound **3i**

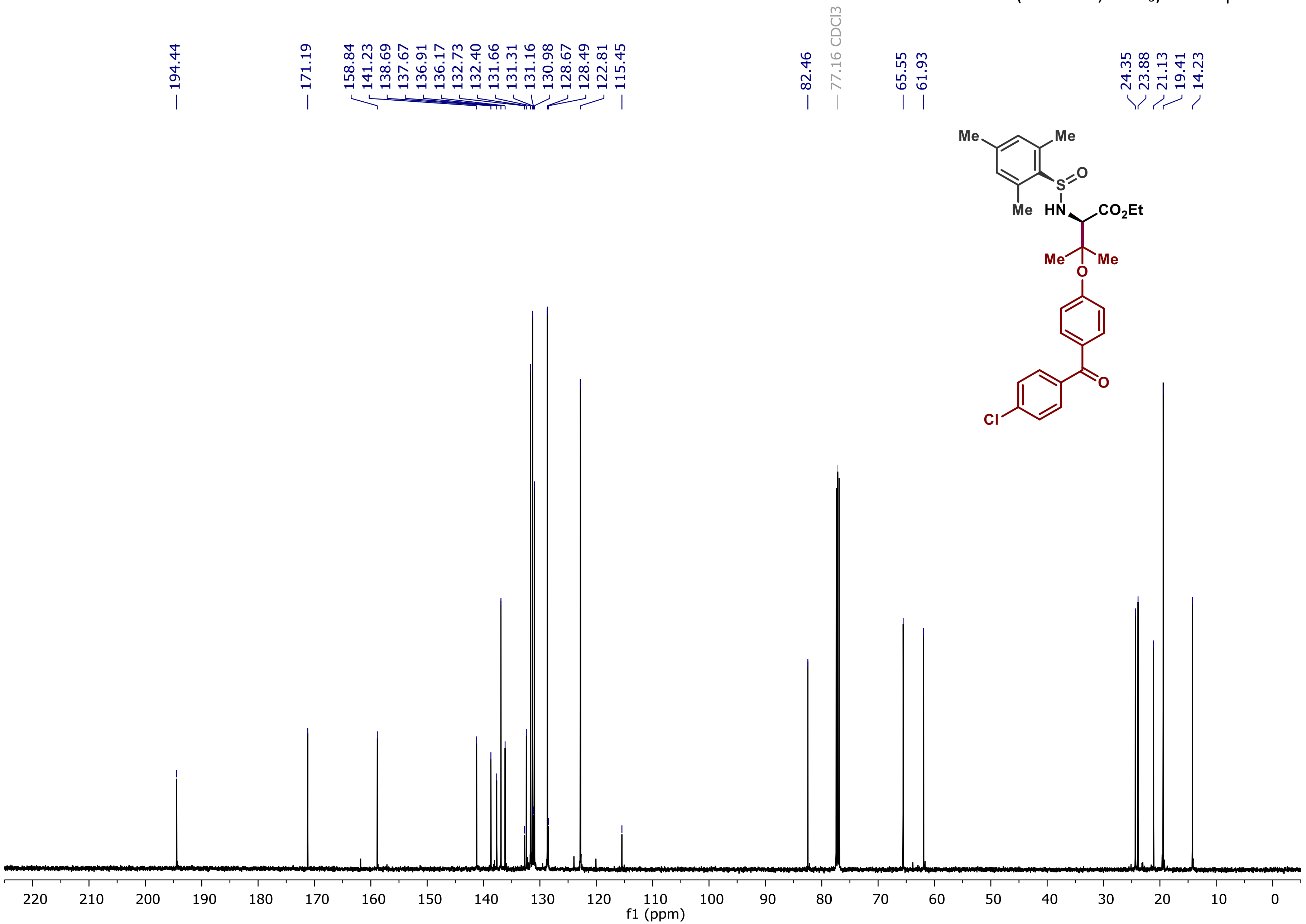

^{1}H NMR (500 MHz, $CDCl_3$) of compound **3j**

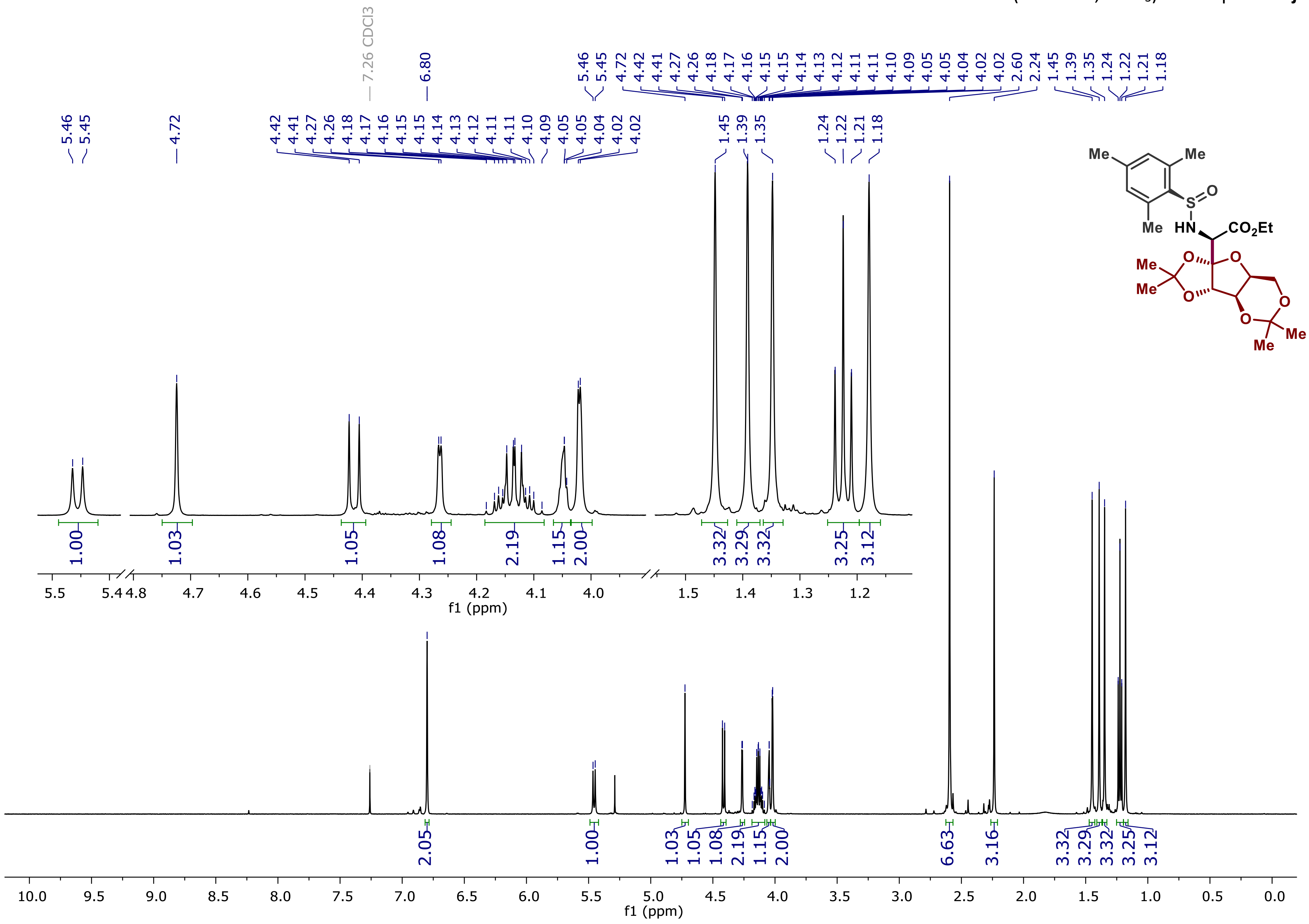

^{13}C NMR (126 MHz, $CDCl_3$) of compound **3j**

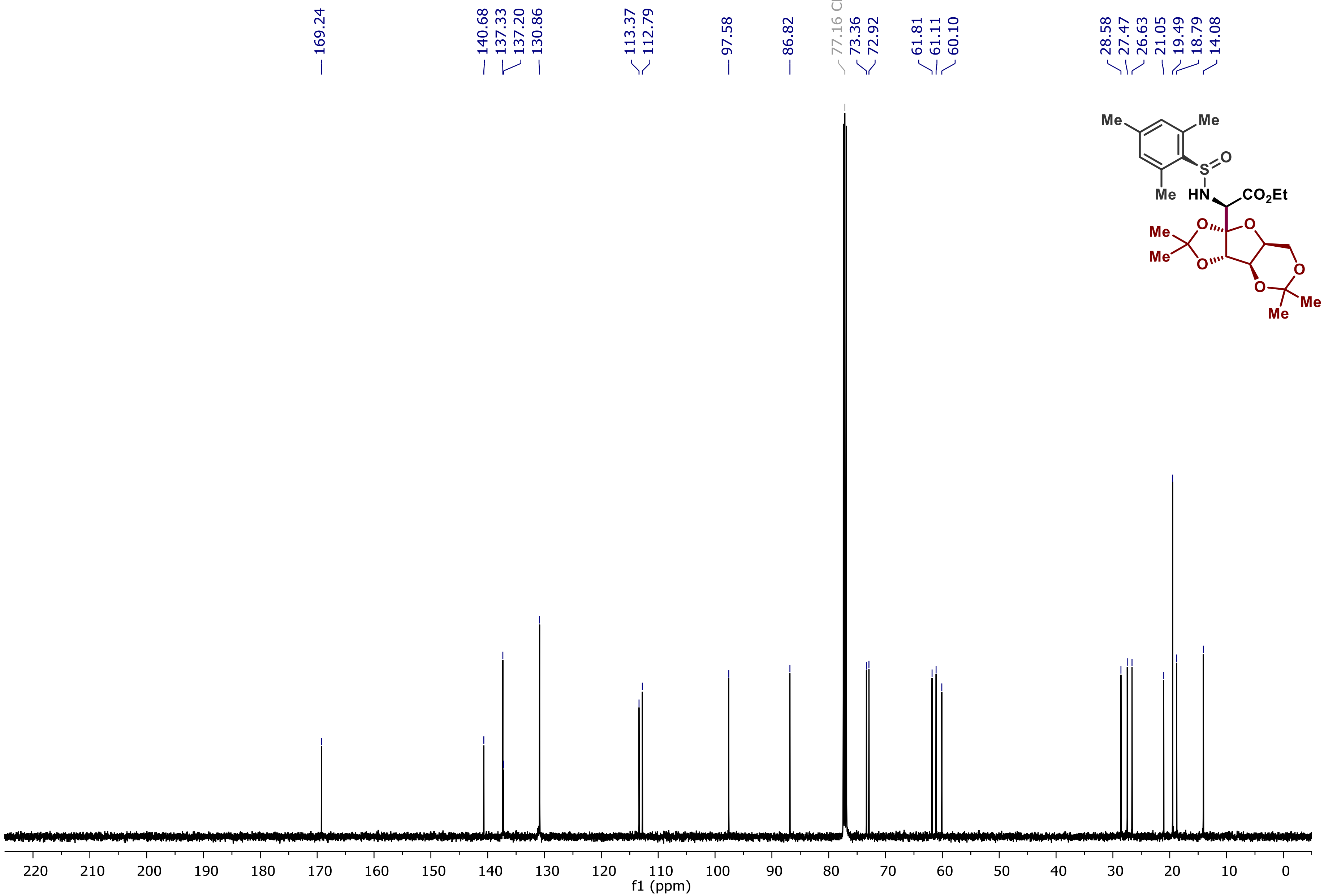

^{1}H NMR (500 MHz, $CDCl_3$) of compound **3k**

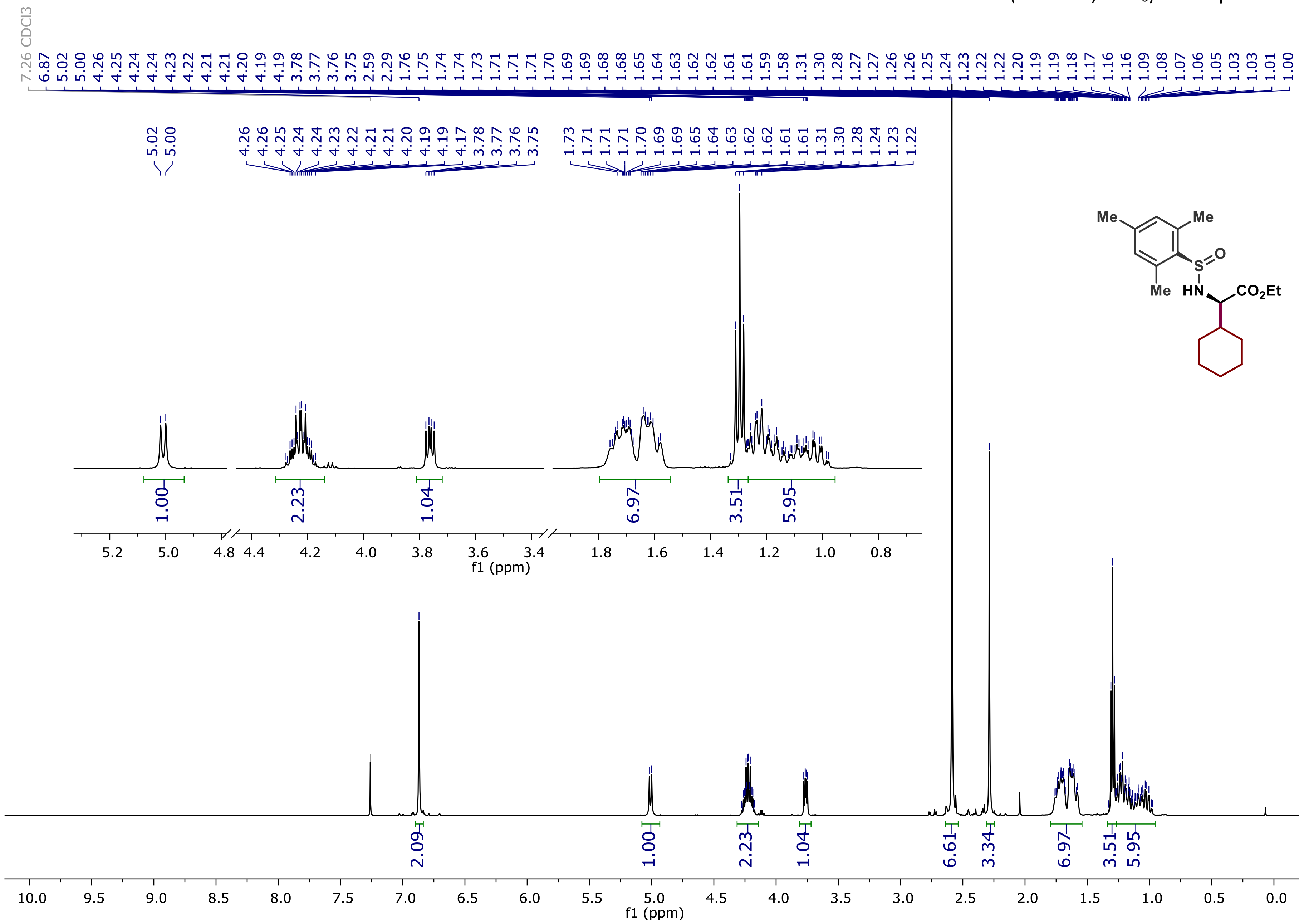

^{13}C NMR (126 MHz, $CDCl_3$) of compound **3k**

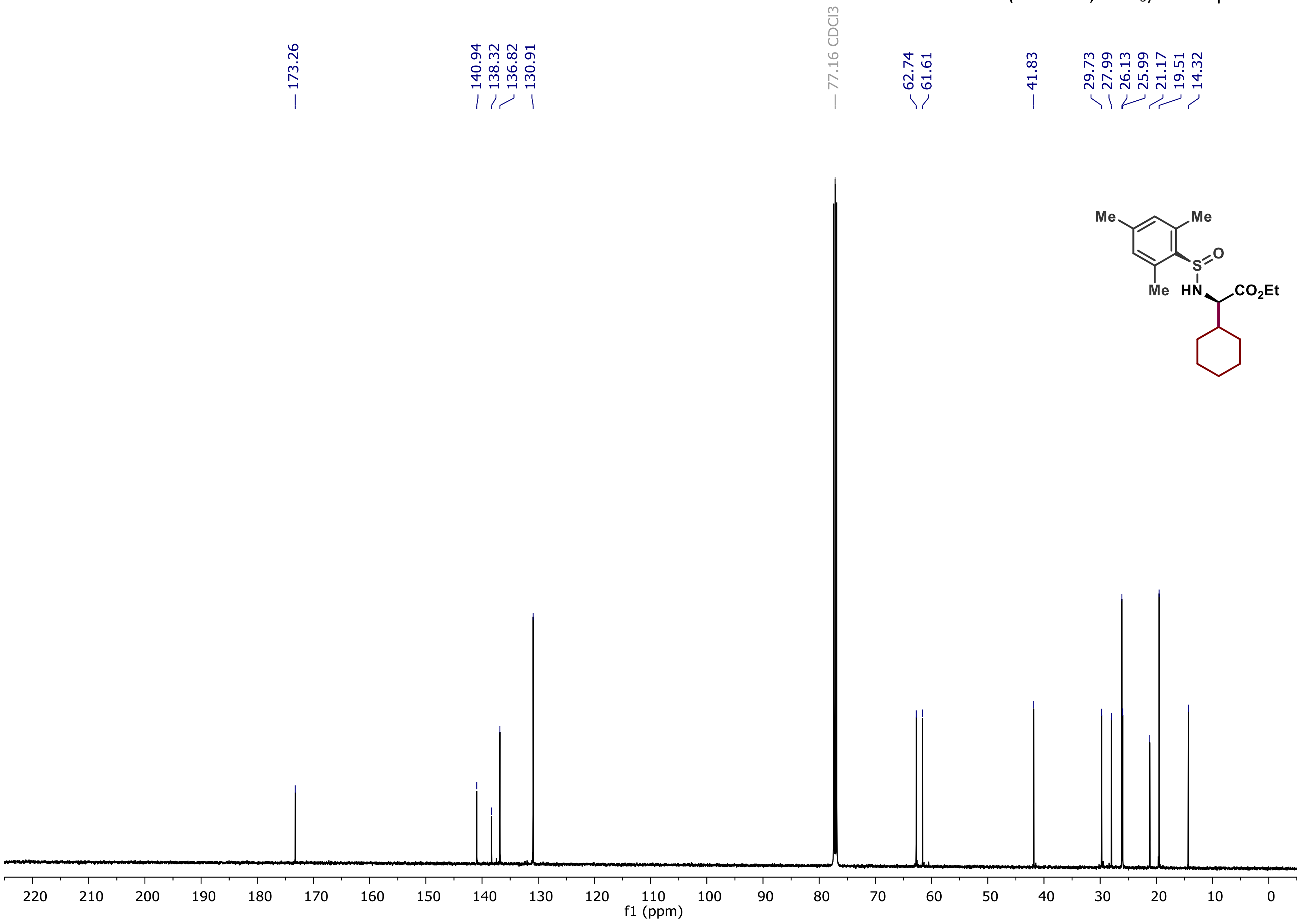

^{1}H NMR (500 MHz, $CDCl_3$) of compound **3l**

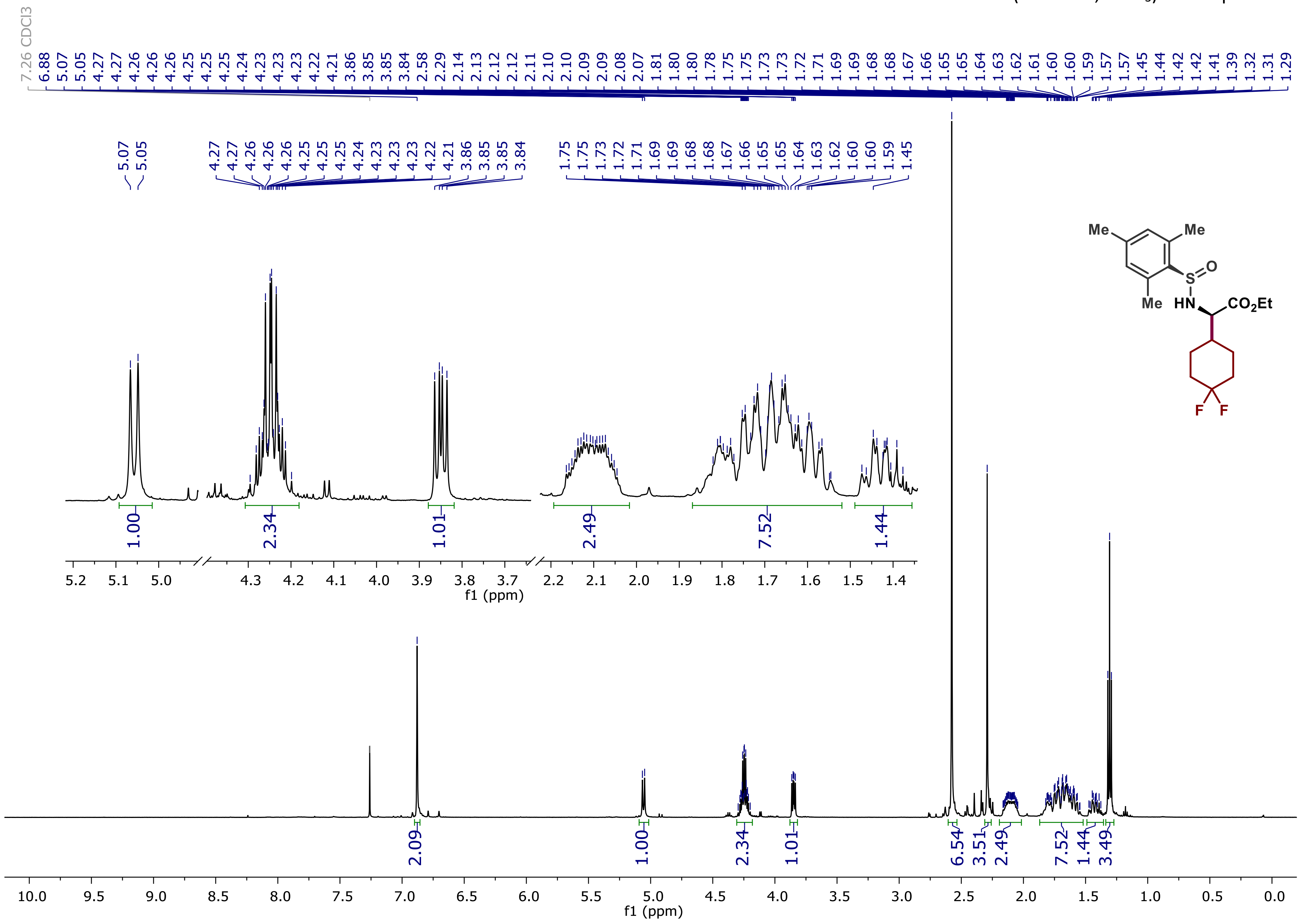

^{13}C NMR (126 MHz, $CDCl_3$) of compound **3l**

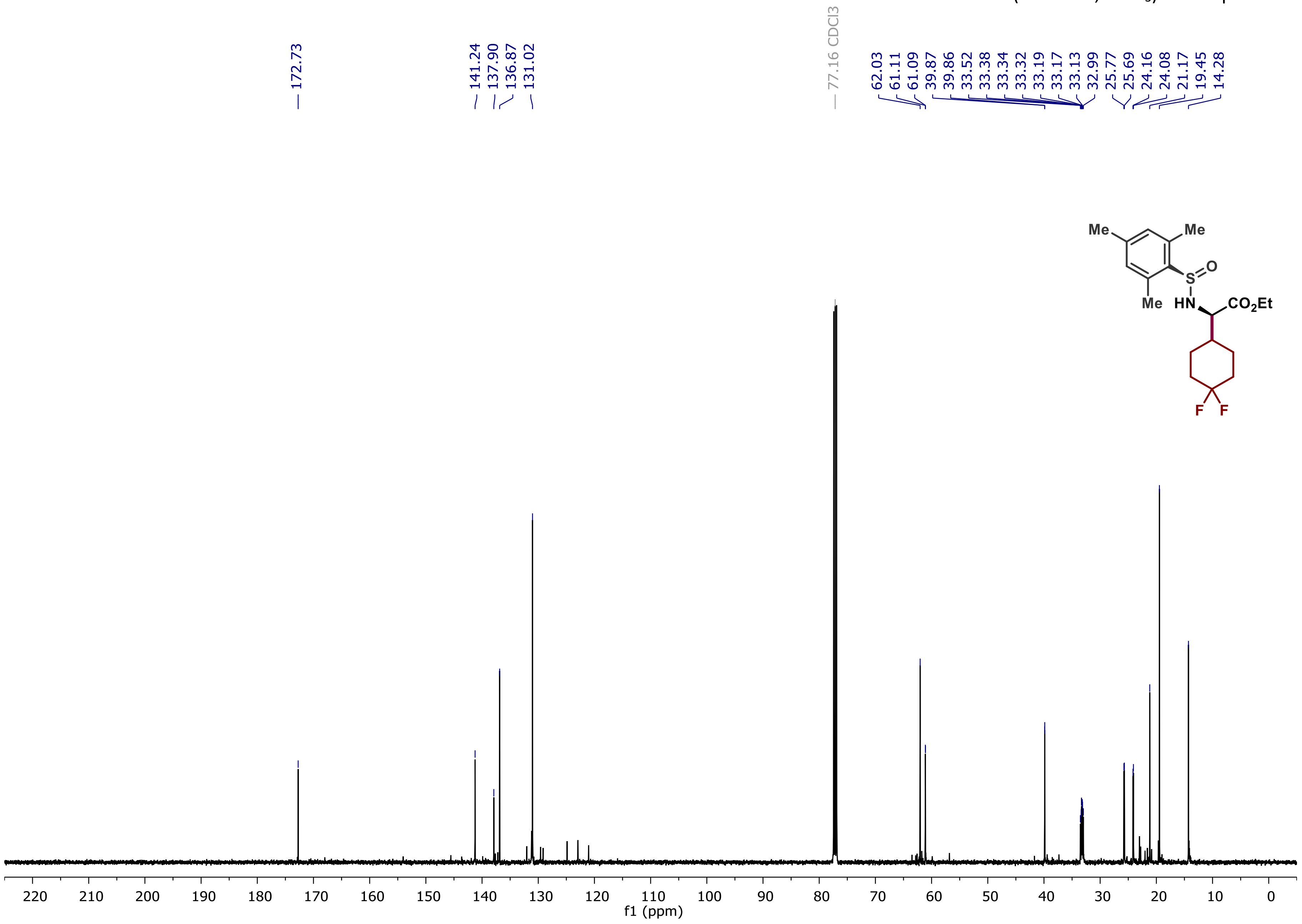

^{19}F NMR (377 MHz, $CDCl_3$) of compound **3l**

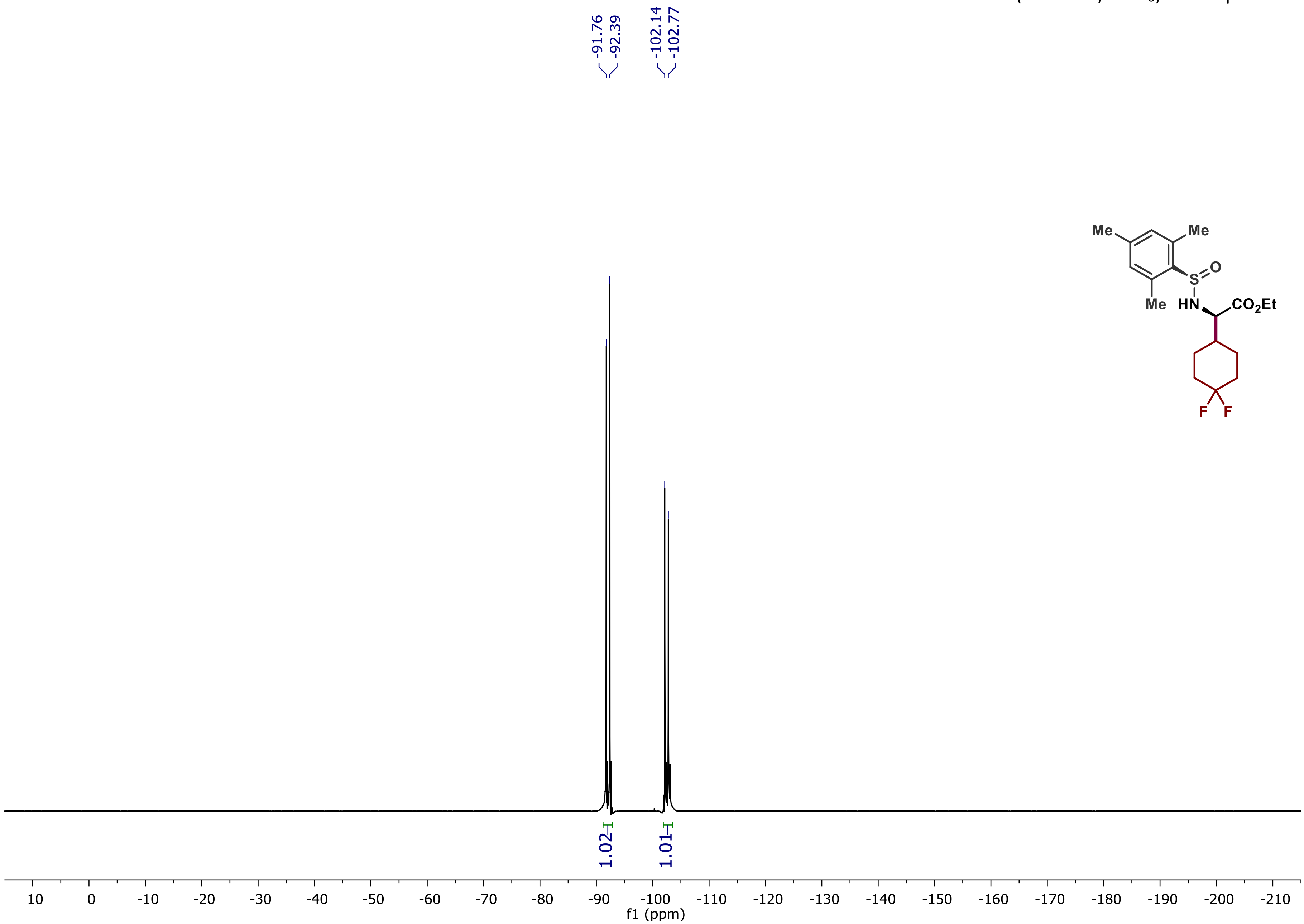

^{1}H NMR (500 MHz, $CDCl_3$) of compound **3m** (*cis/trans*-diastereomer **3m-1**)

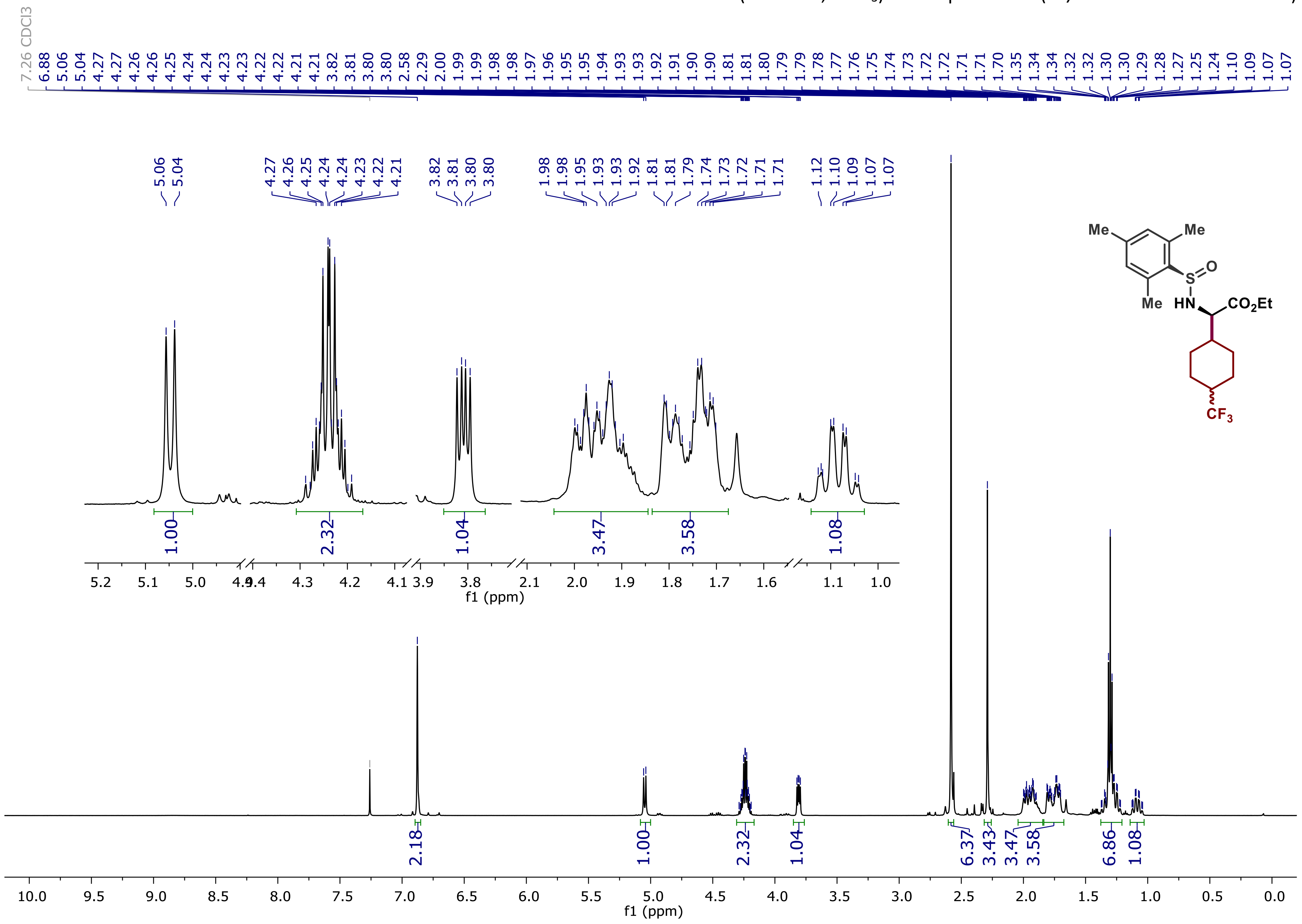

^{13}C NMR (126 MHz, $CDCl_3$) of compound **3m** (*cis/trans*-diastereomer **3m-1**)

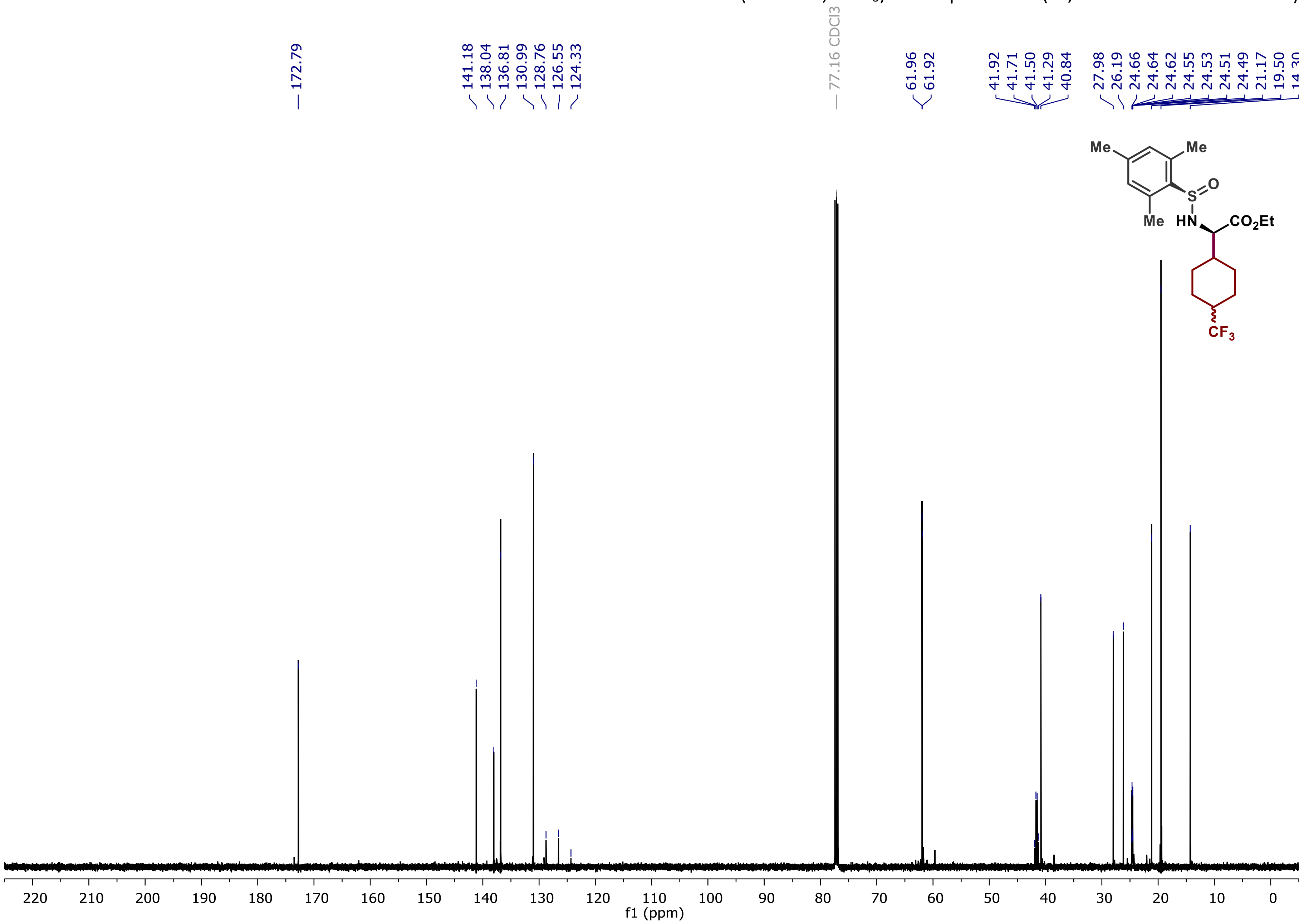

^{19}F NMR (377 MHz, $CDCl_3$) of compound **3m** (*cis/trans*-diastereomer **3m-1**)

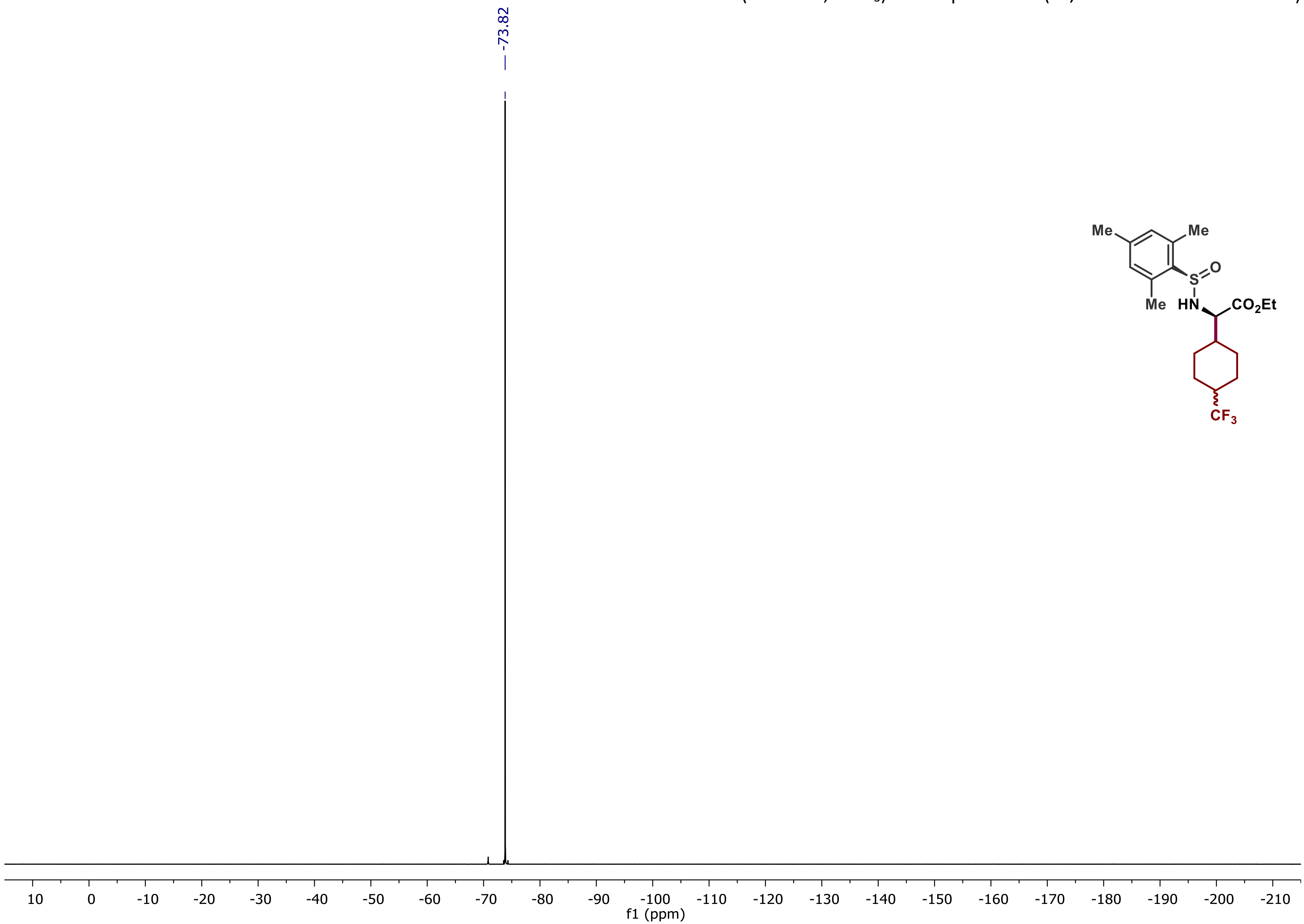

^{1}H NMR (500 MHz, $CDCl_3$) of compound **3m** (*cis/trans*-diastereomers **3m-1**/**3m-2** 3:5)

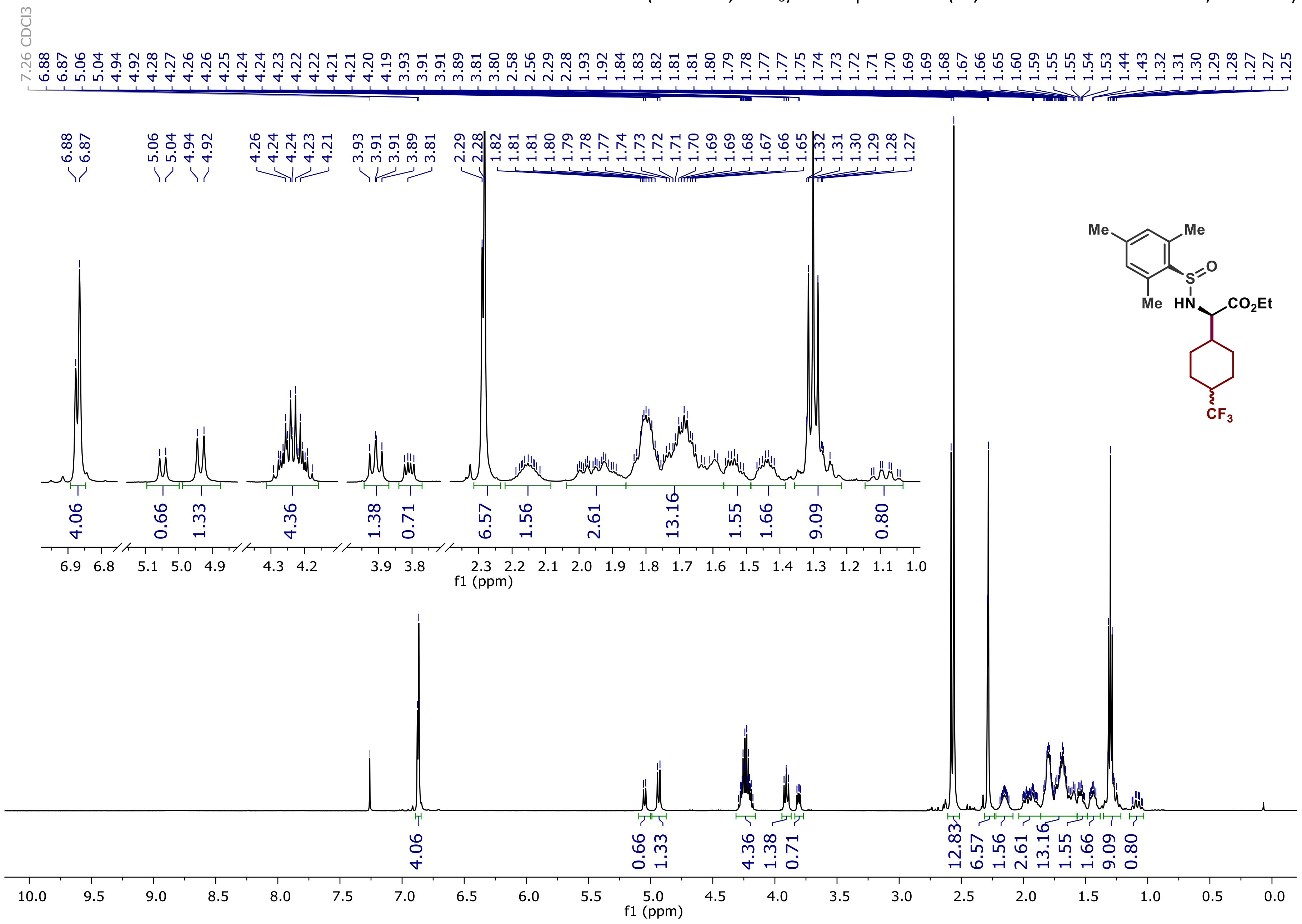

^{13}C NMR (126 MHz, $CDCl_3$) of compound **3m** (*cis/trans*-diastereomers **3m-1/3m-2** 3:5)

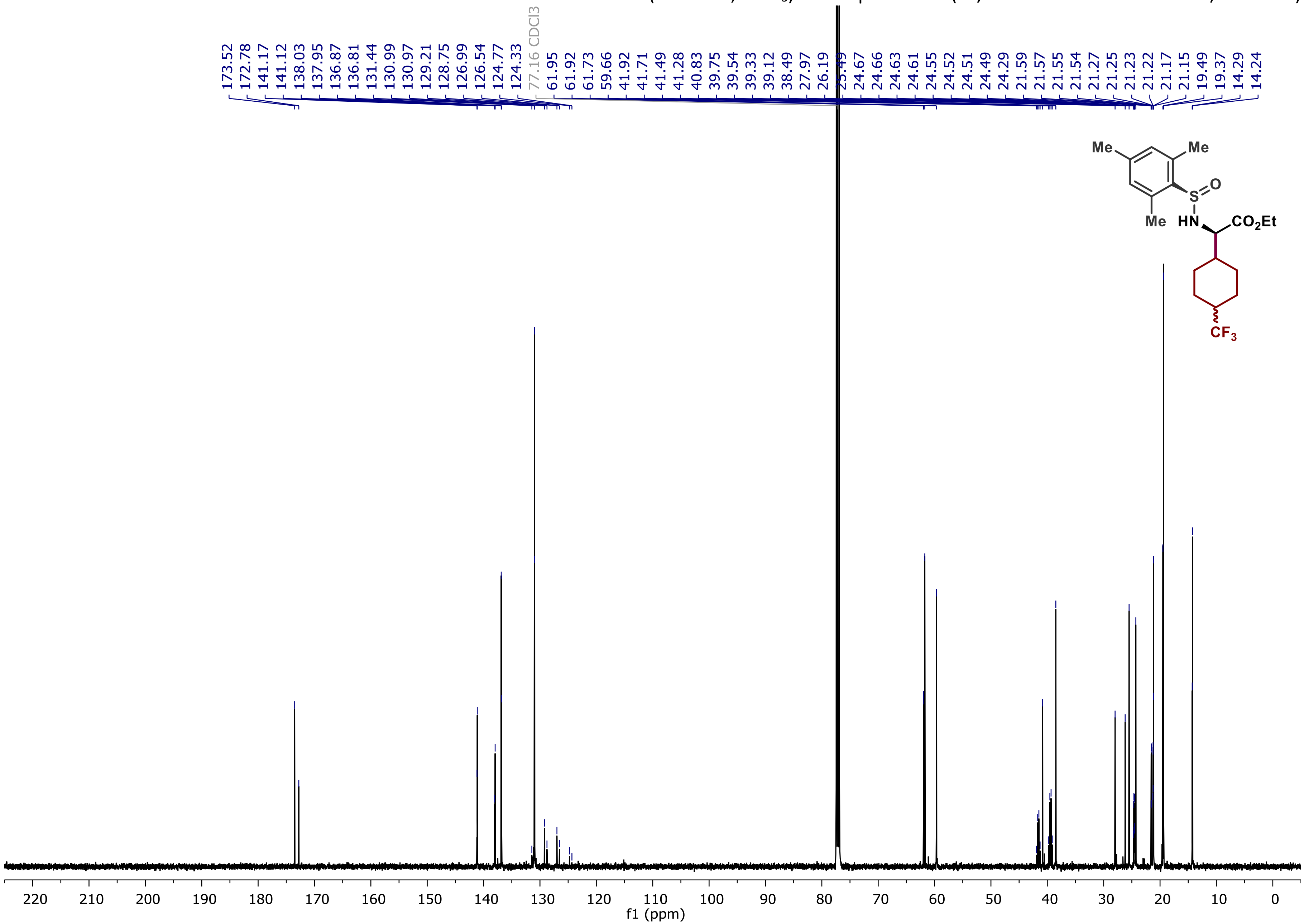

^{19}F NMR (377 MHz, $CDCl_3$) of compound **3m** (*cis/trans*-diastereomers **3m-1**/**3m-2** 3:5)

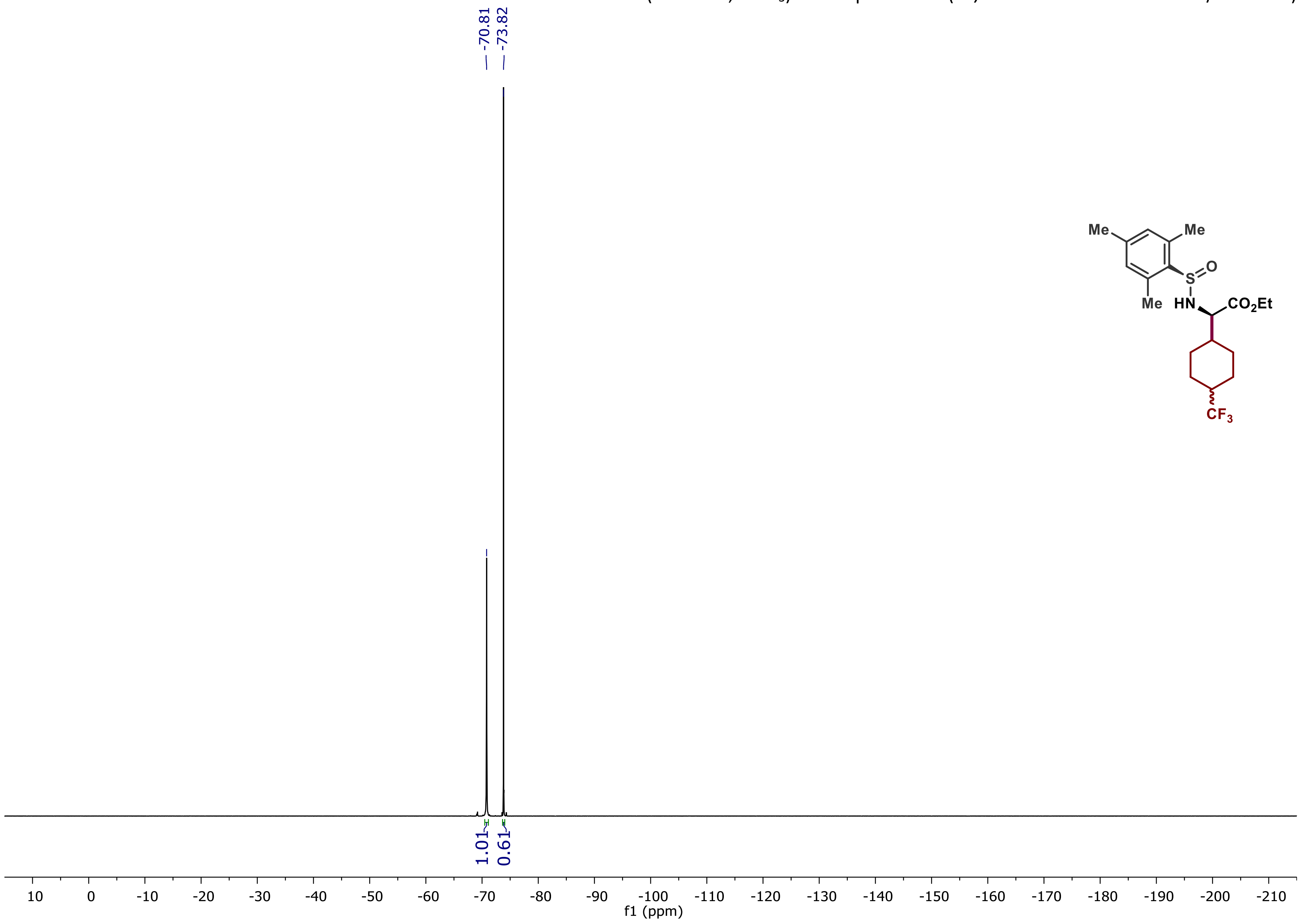

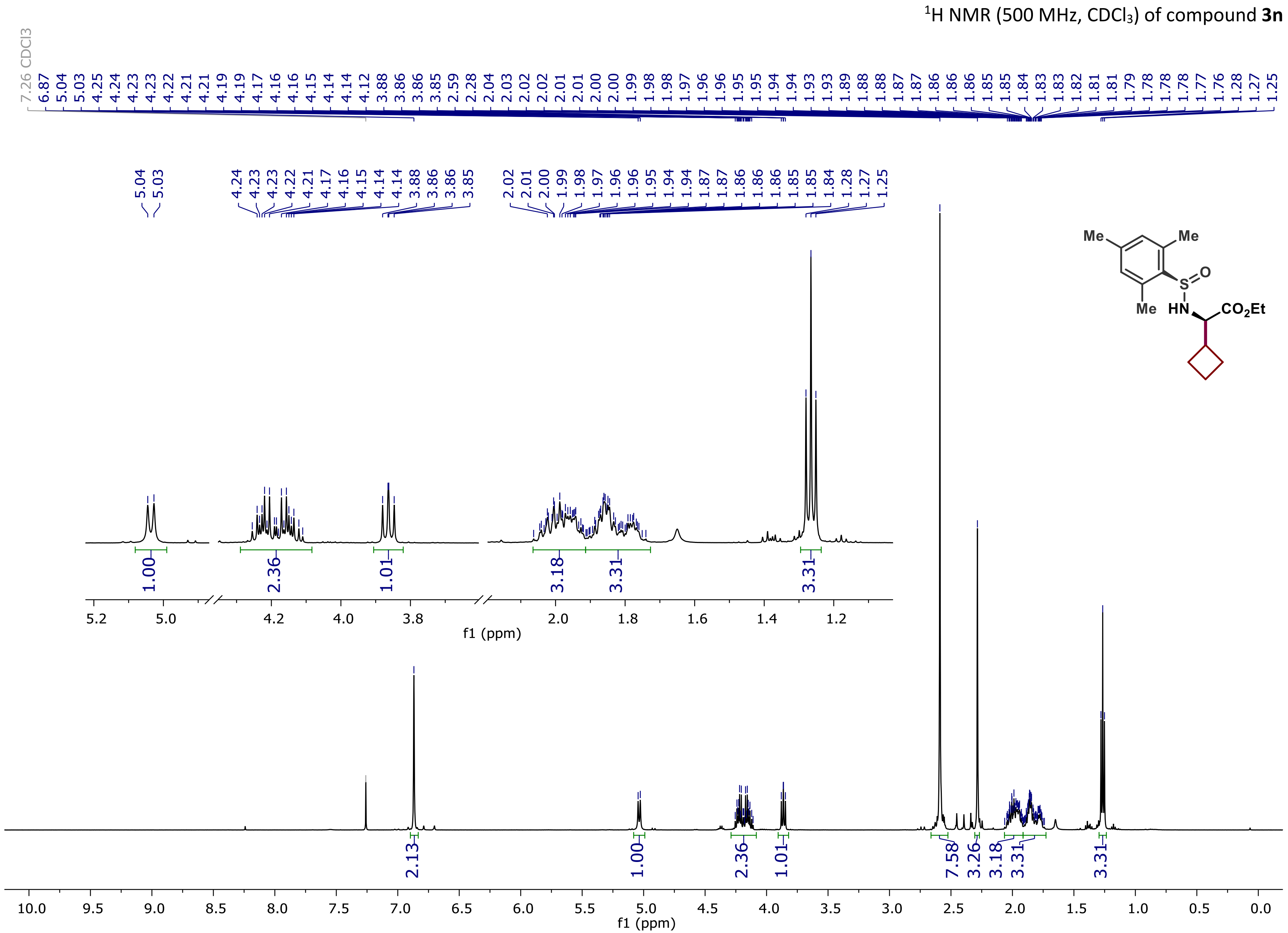

^{1}H NMR (500 MHz, CDCl3) of compound 3n
7.26 CDCl3
6.87
5.04
5.03
4.25
4.24
4.23
4.23
4.22
4.21
4.21
4.19
4.19
4.17
4.16
4.16
4.15
4.14
4.14
4.12
3.88
3.86
3.86
3.85
2.59
2.28
2.04
2.03
2.02
2.02
2.01
2.01
2.00
2.00
1.99
1.98
1.98
1.97
1.96
1.96
1.95
1.95
1.94
1.94
1.93
1.93
1.89
1.88
1.88
1.87
1.87
1.86
1.86
1.86
1.85
1.85
1.84
1.83
1.83
1.82
1.81
1.81
1.79
1.78
1.78
1.78
1.77
1.76
1.28
1.27
1.25
Me
Me
Me
S
O
HN
CO2Et
f1 (ppm)
2.13
1.00
2.36
1.01
7.58
3.26
3.18
3.31
3.31

^{13}C NMR (126 MHz, $CDCl_3$) of compound **3n**

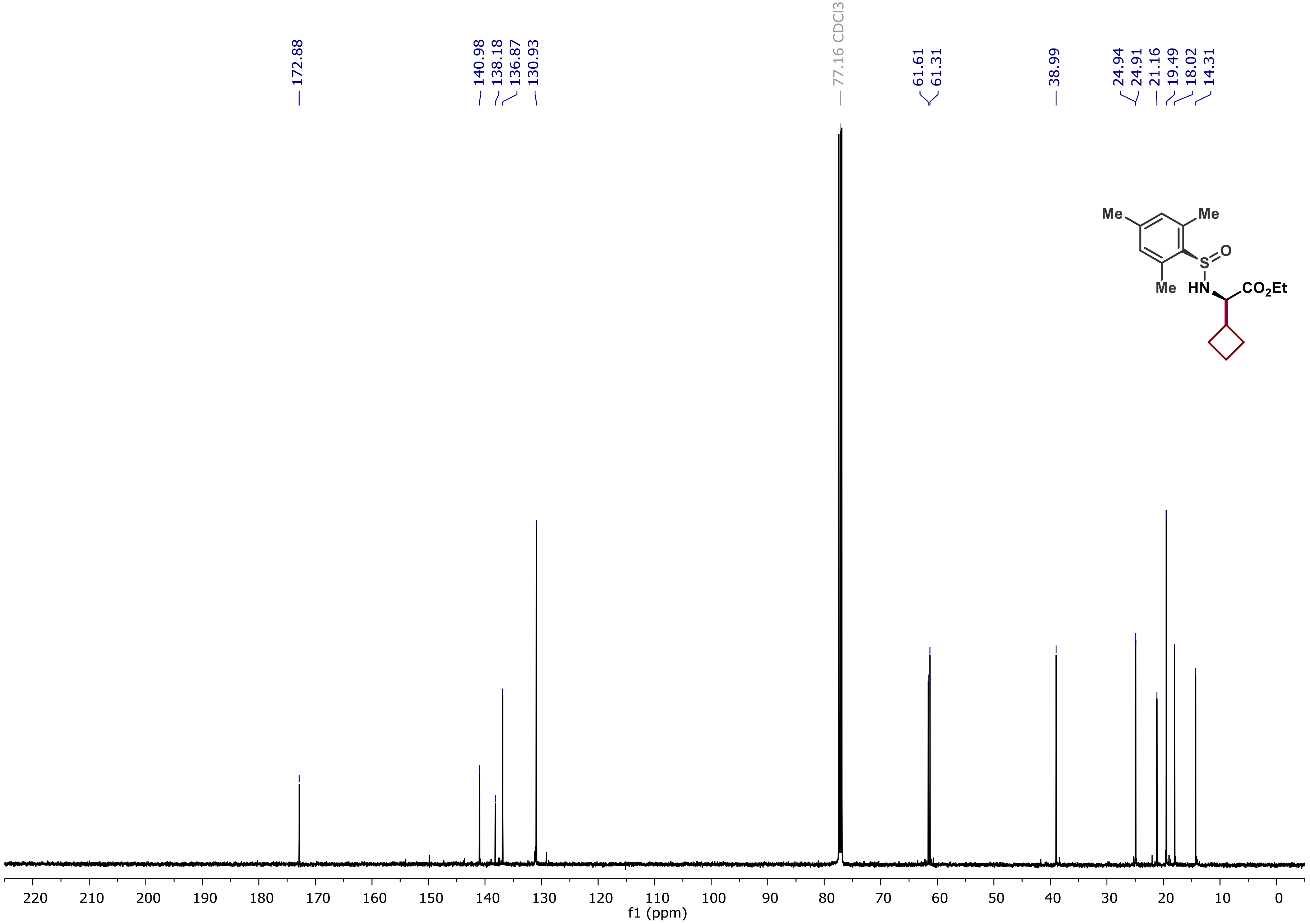

^1H NMR (500 MHz, $CDCl_3$) of compound **3o**

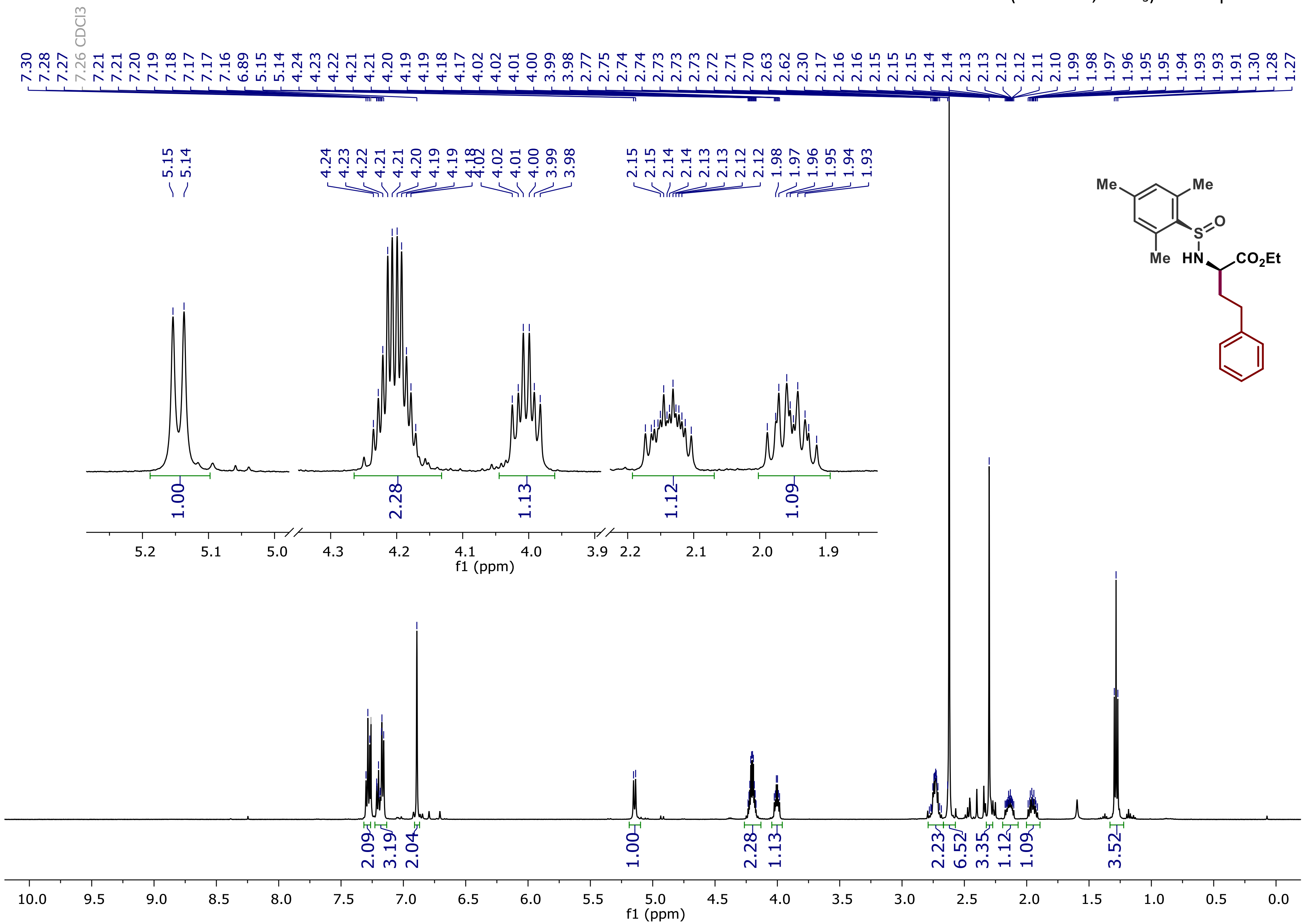

^{13}C NMR (126 MHz, $CDCl_3$) of compound **3o**

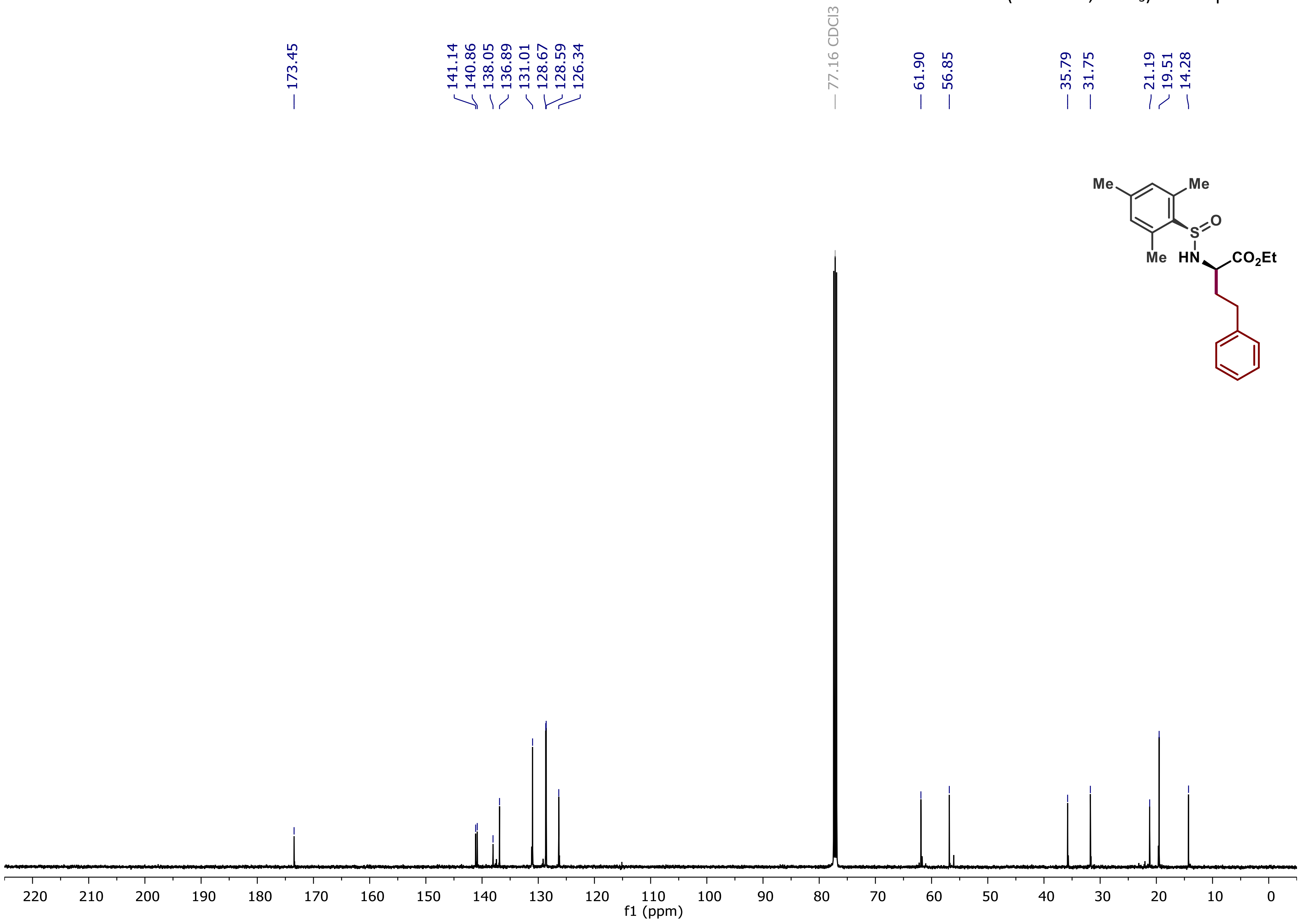

^{1}H NMR (500 MHz, $CDCl_3$) of compound **3p**

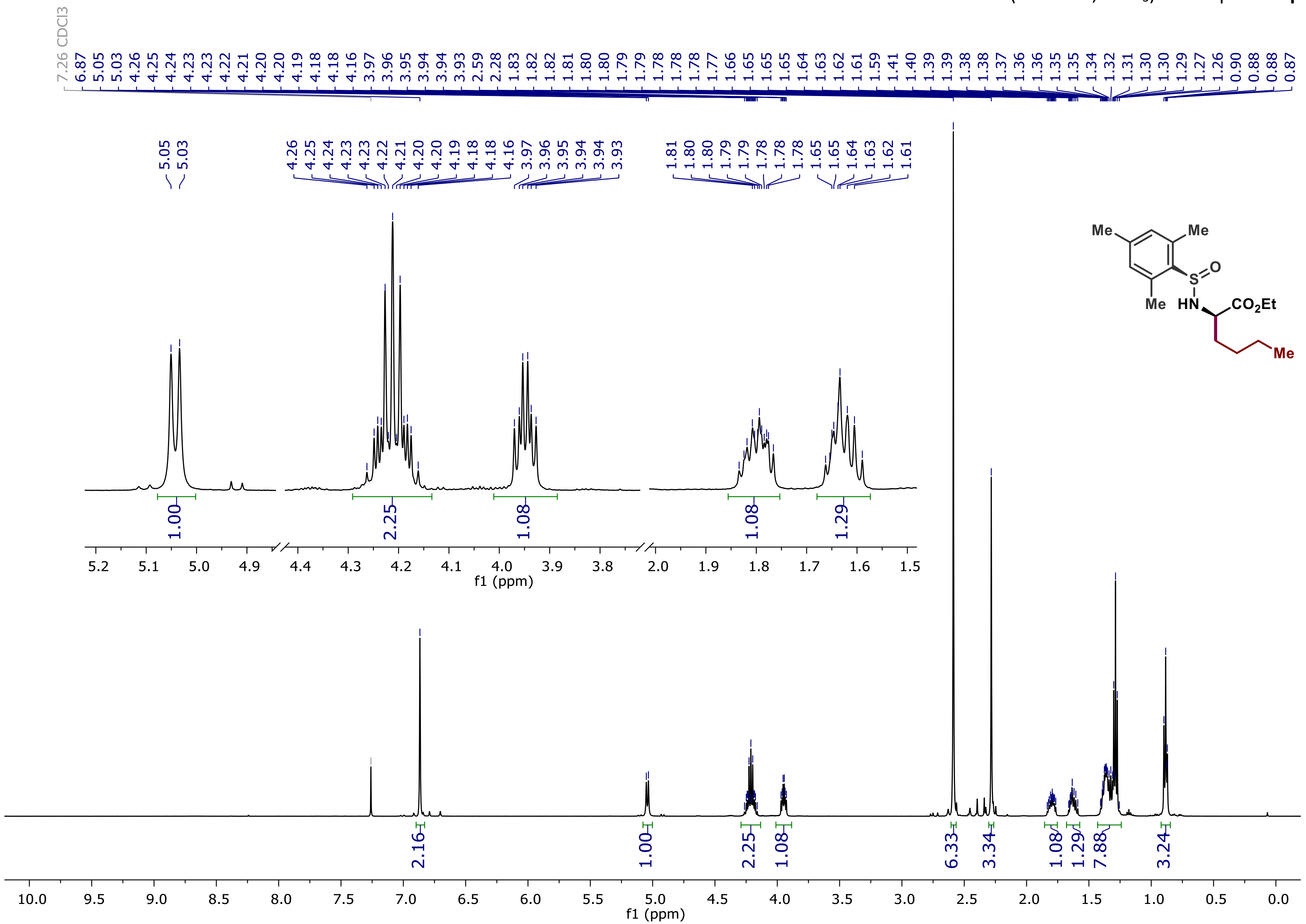

^{13}C NMR (126 MHz, $CDCl_3$) of compound **3p**

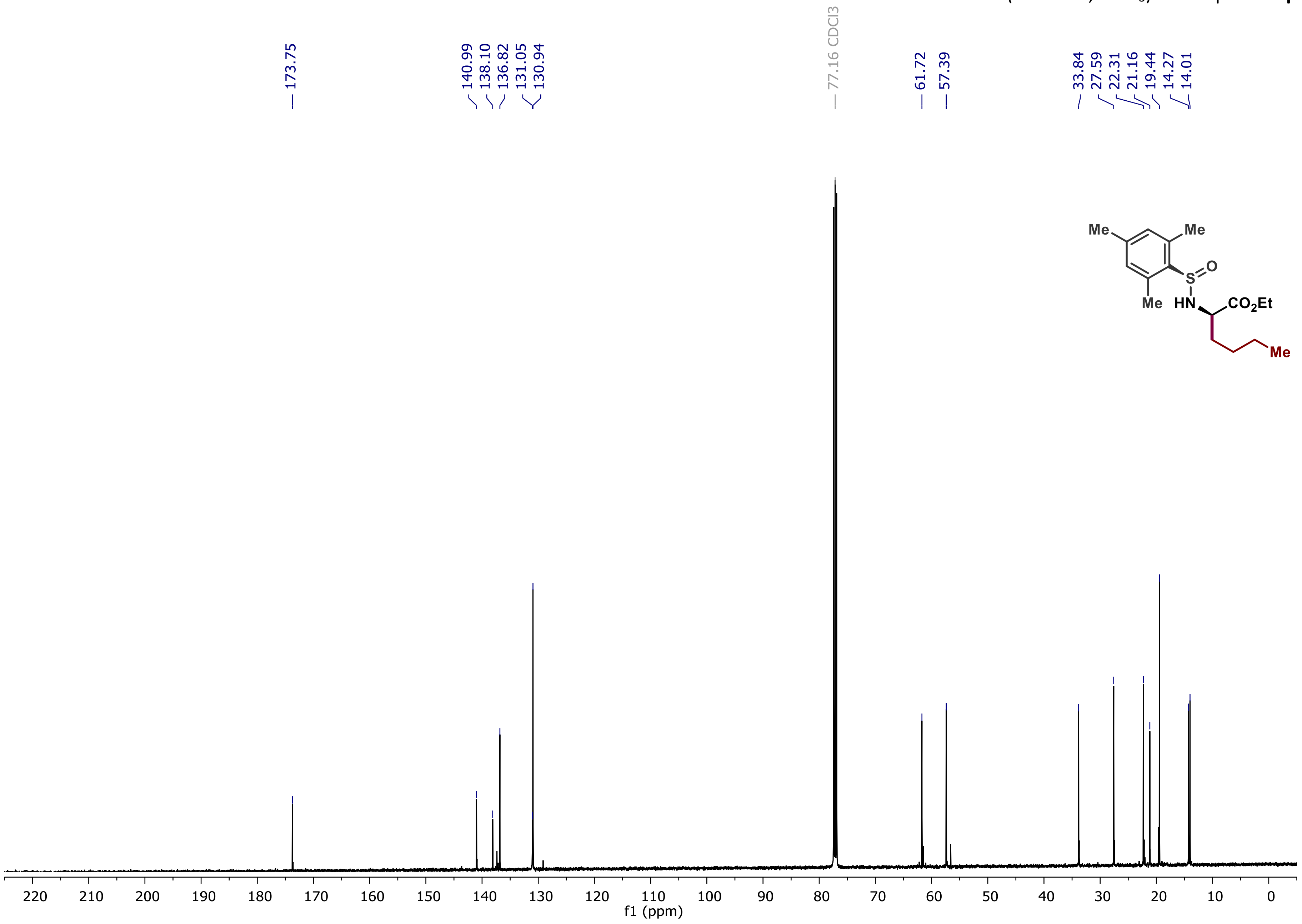

^{1}H NMR (500 MHz, $CDCl_3$) of compound **3q**

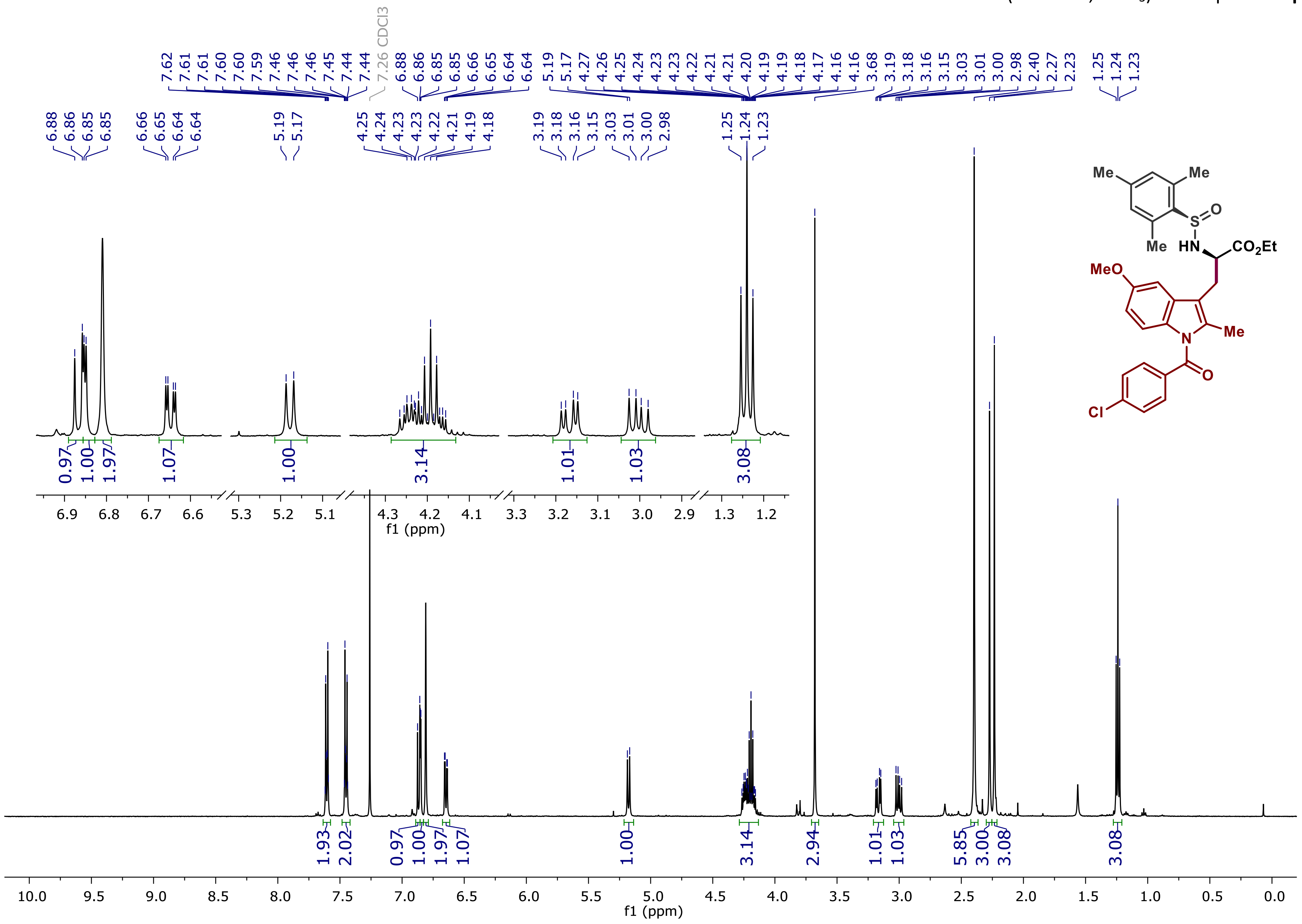

^{13}C NMR (126 MHz, CDCl$_3$) of compound **3q**

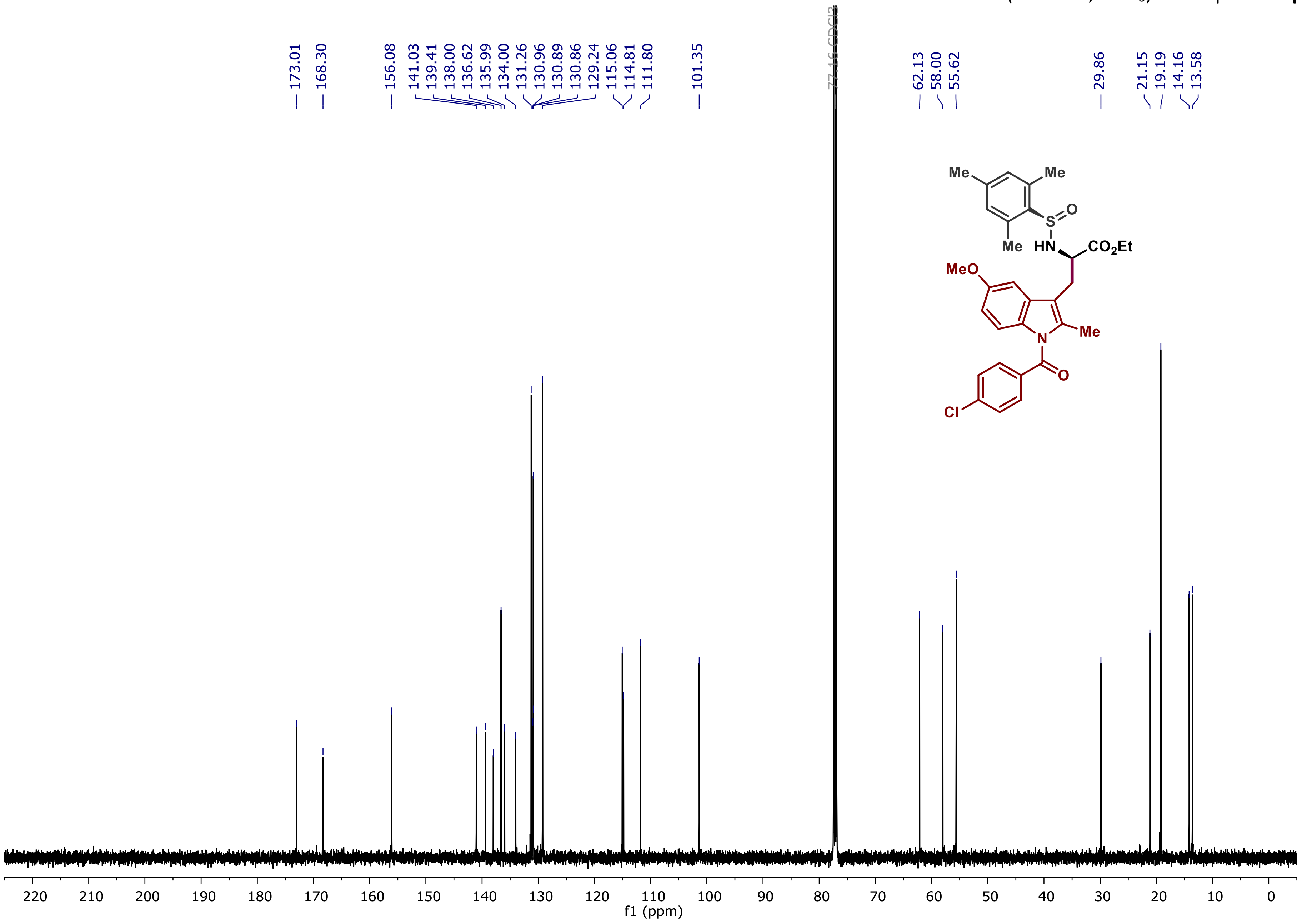

^{1}H NMR (500 MHz, $CDCl_3$) of compound **6q**

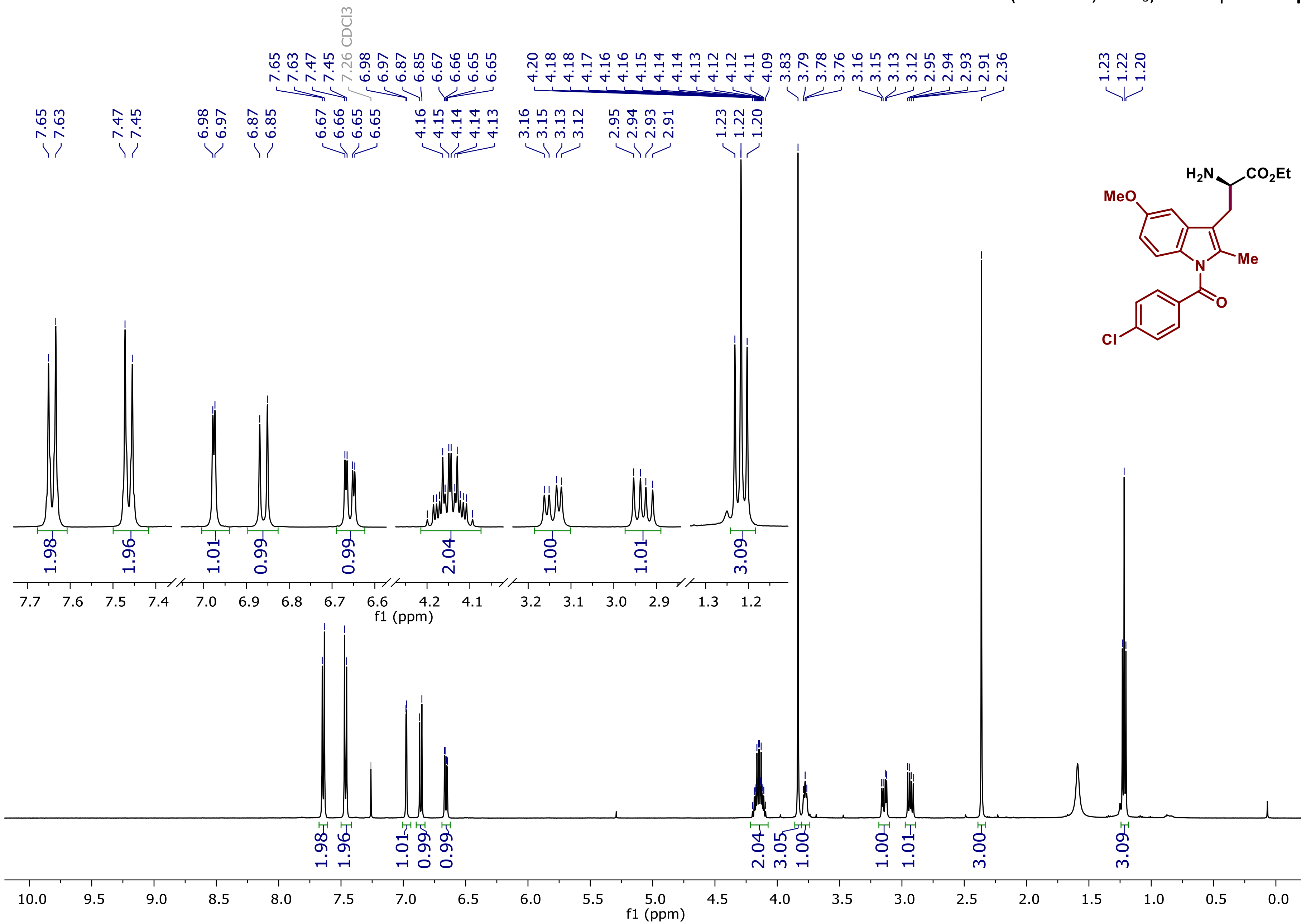

^{13}C NMR (126 MHz, $CDCl_3$) of compound **6q**

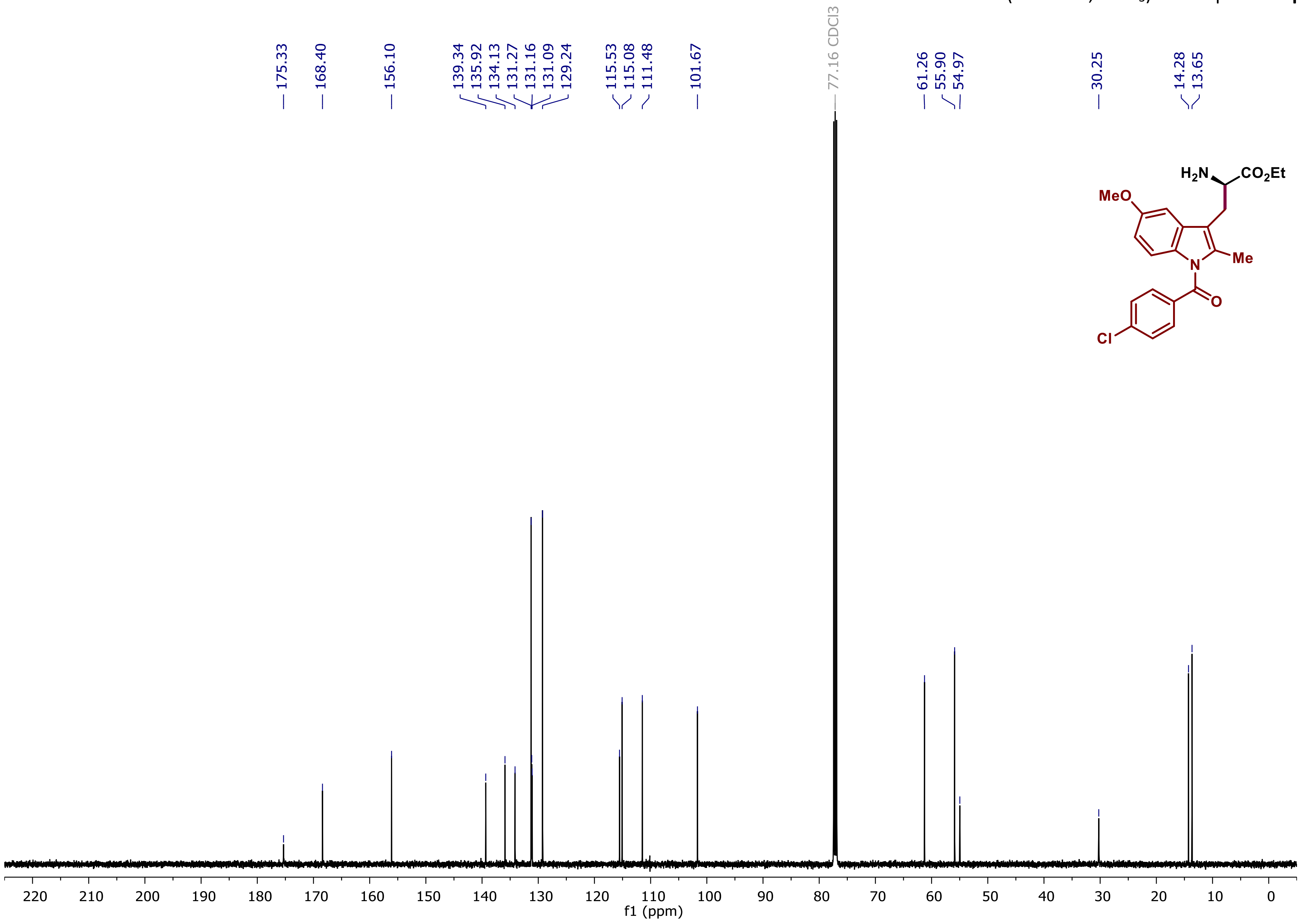

^{1}H NMR (500 MHz, $CDCl_3$) of compound **3r** (β-diastereomer **3r-1**)

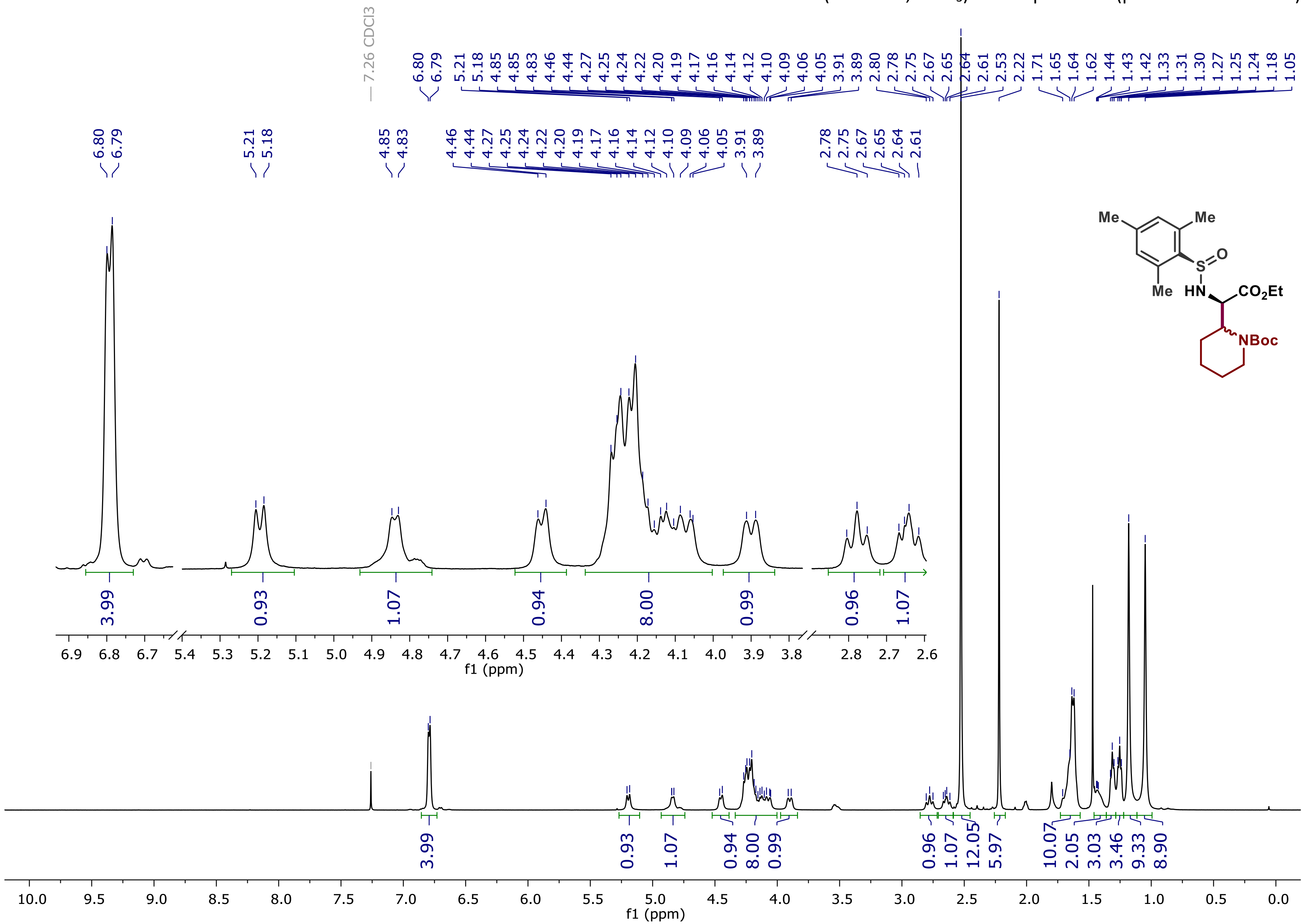

^{13}C NMR (126 MHz, $CDCl_3$) of compound **3r** (β-diastereomer **3r-1**)

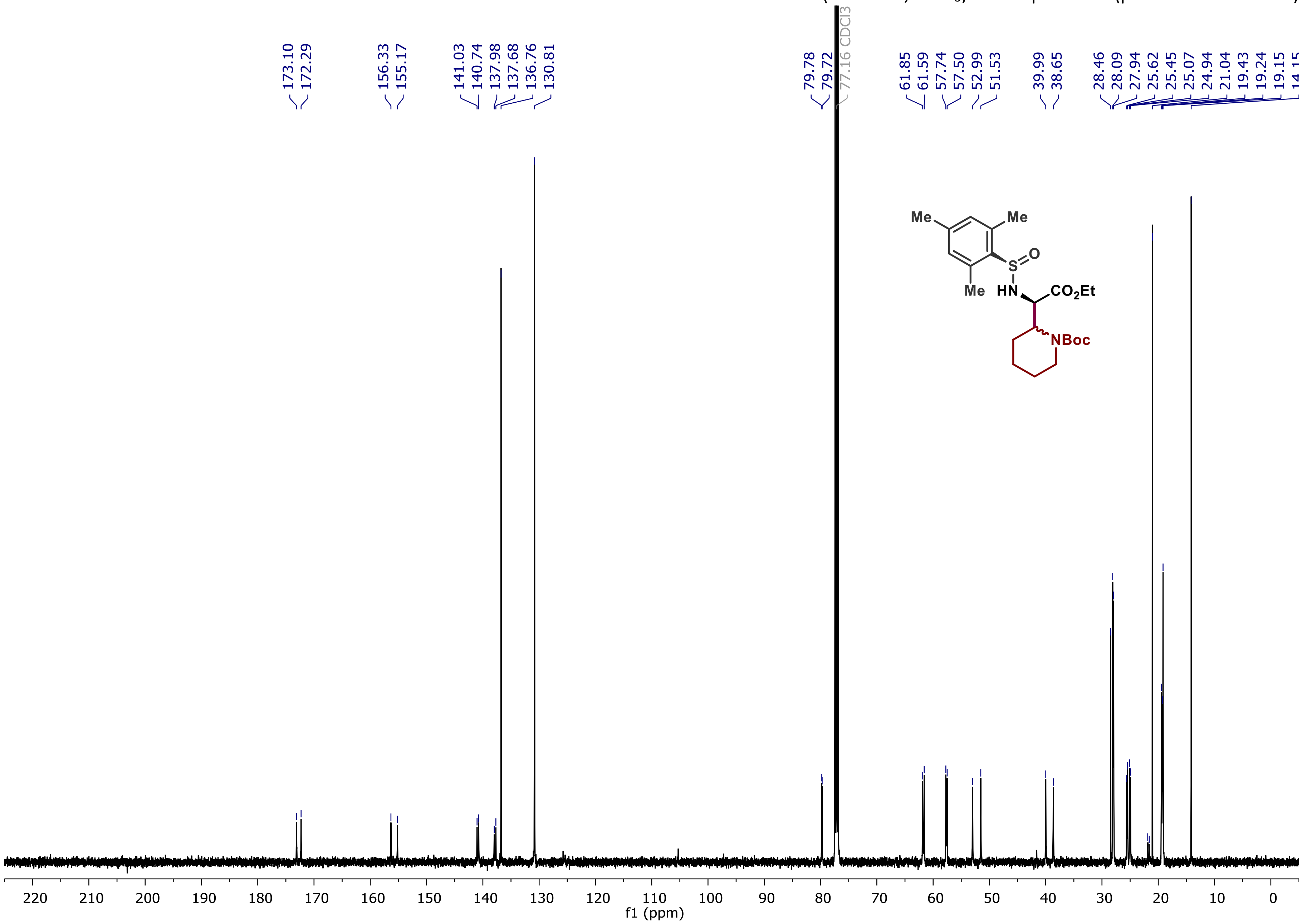

^{1}H NMR (500 MHz, $CDCl_3$) of compound **3r** (β-diastereomer **3r-2**)

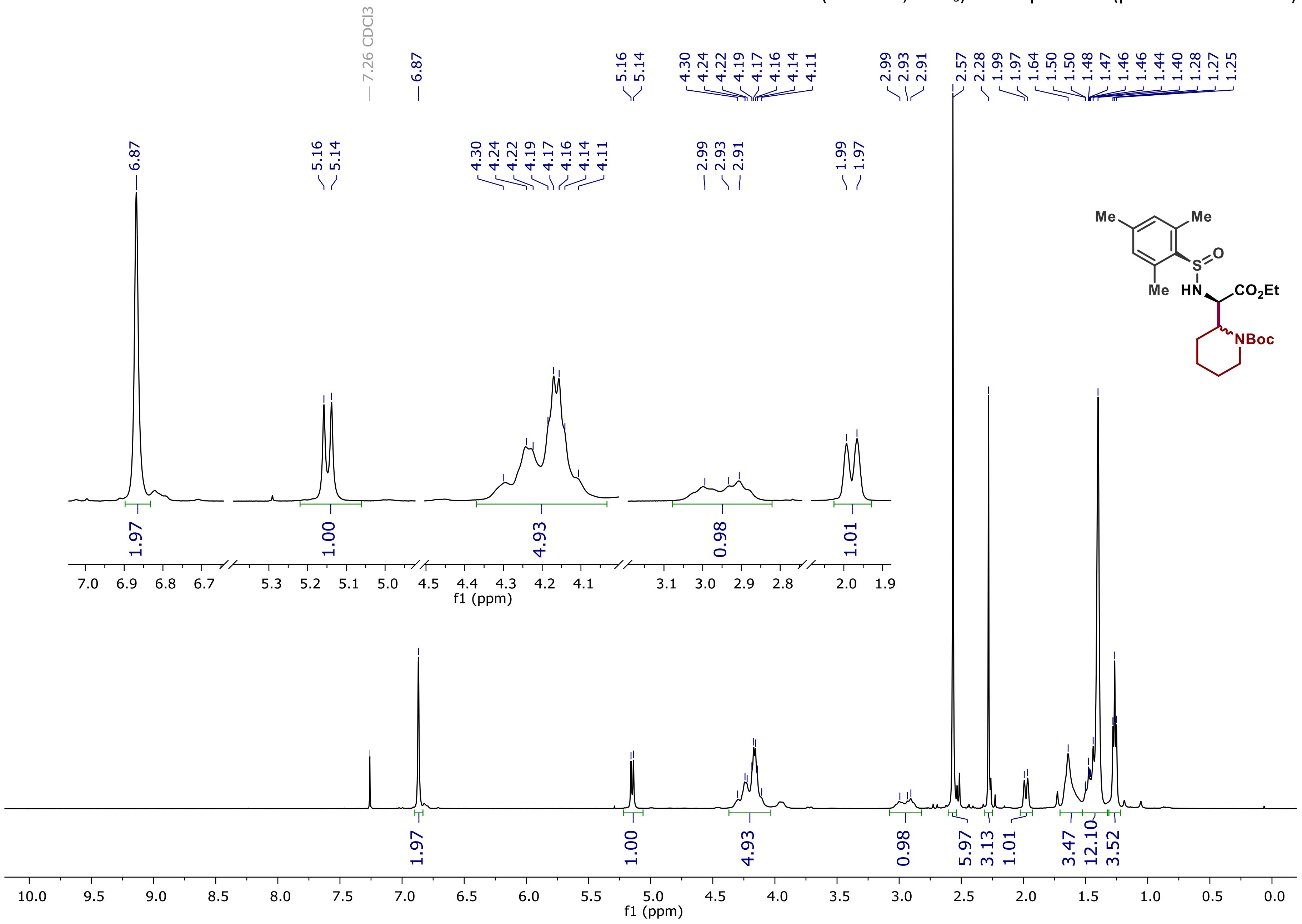

^{13}C NMR (126 MHz, $CDCl_3$) of compound **3r** (β-diastereomer **3r-2**)

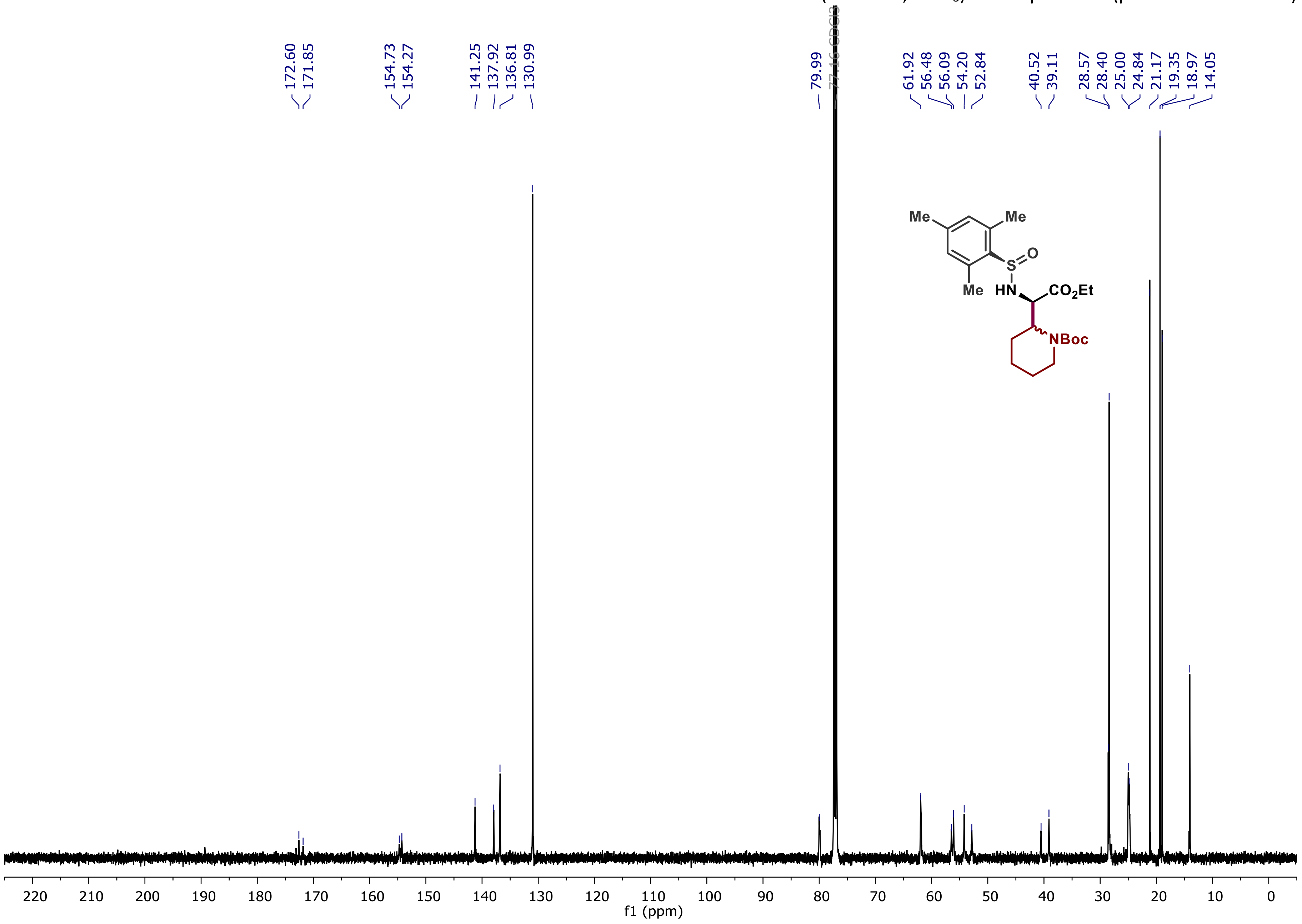

^{1}H NMR (500 MHz, $CDCl_3$) of compound **3s**

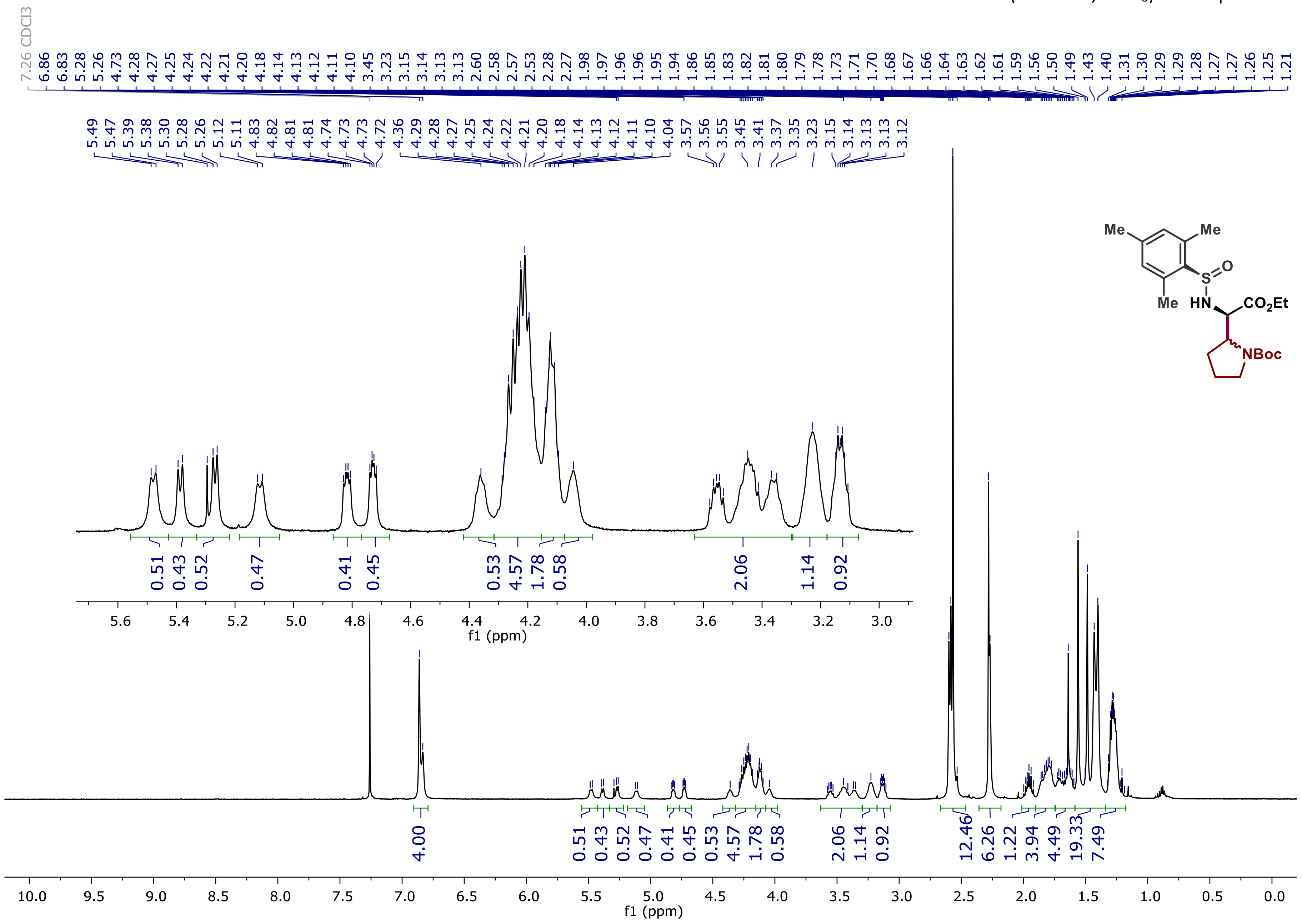

^{13}C NMR (126 MHz, $CDCl_3$) of compound **3s**

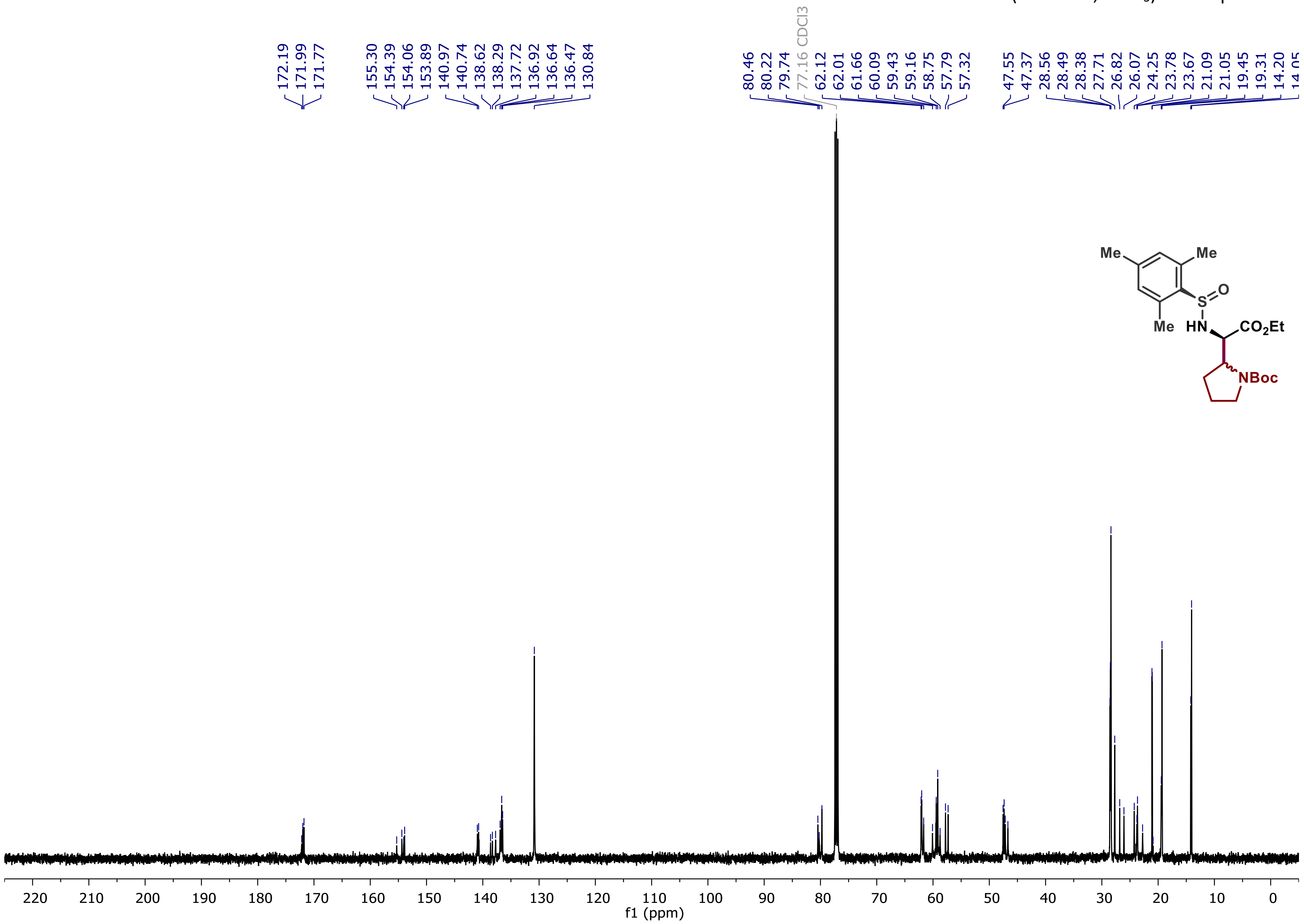

^{1}H NMR (500 MHz, $CDCl_3$) of compound **3t**

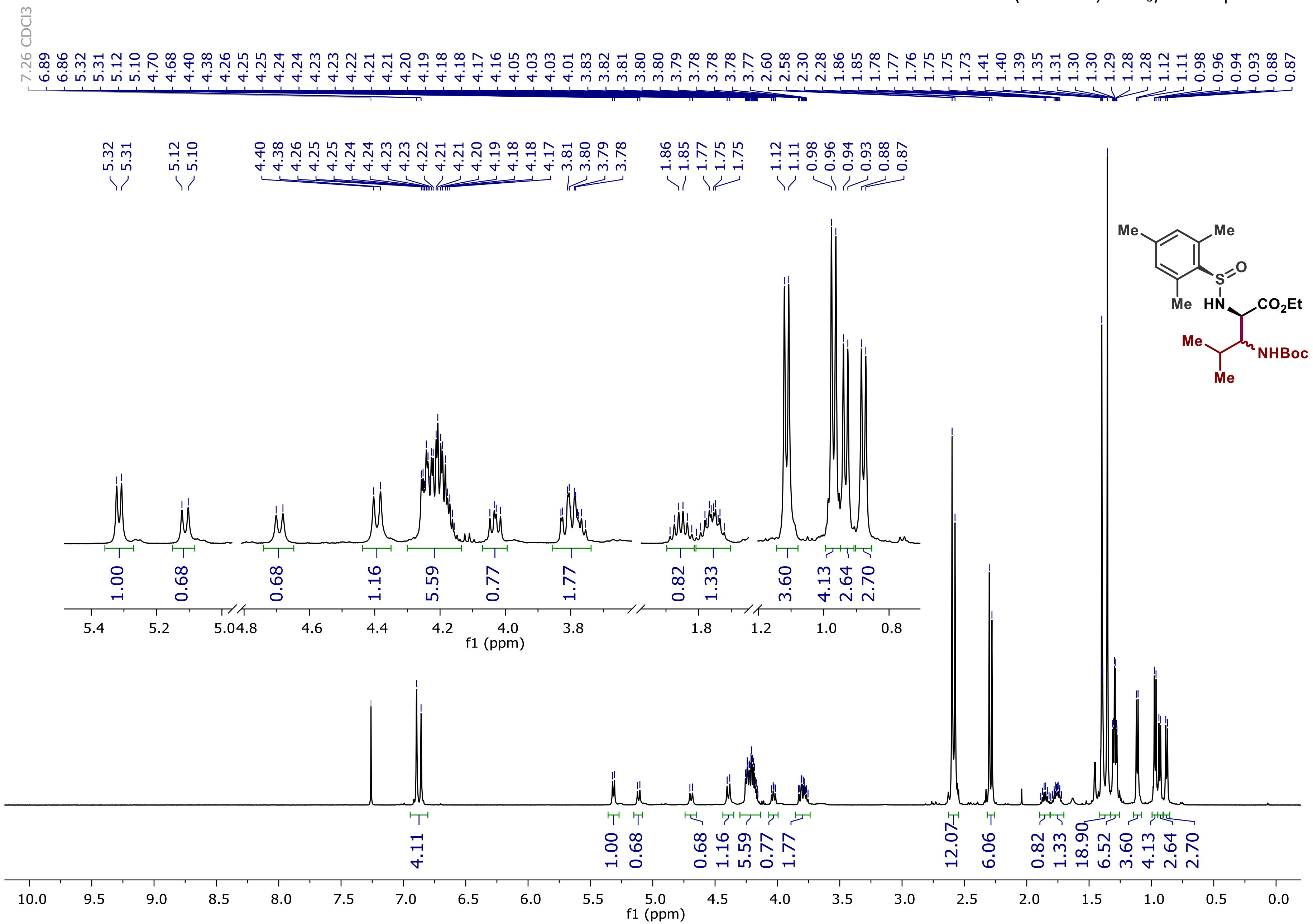

^{13}C NMR (126 MHz, $CDCl_3$) of compound **3t**

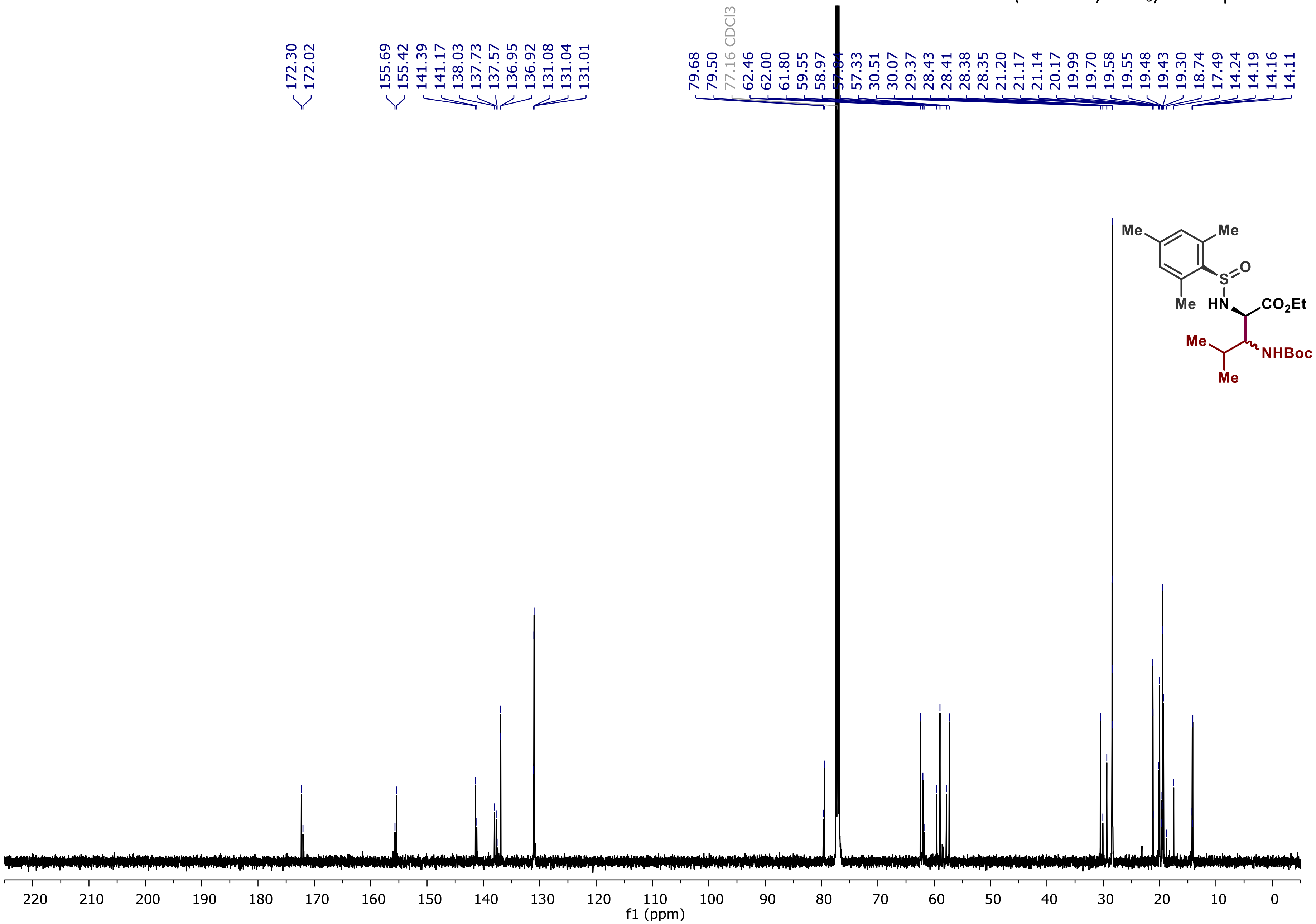

^{1}H NMR (500 MHz, $CDCl_3$) of compound **3u** (β-diastereomer **3u-1**)

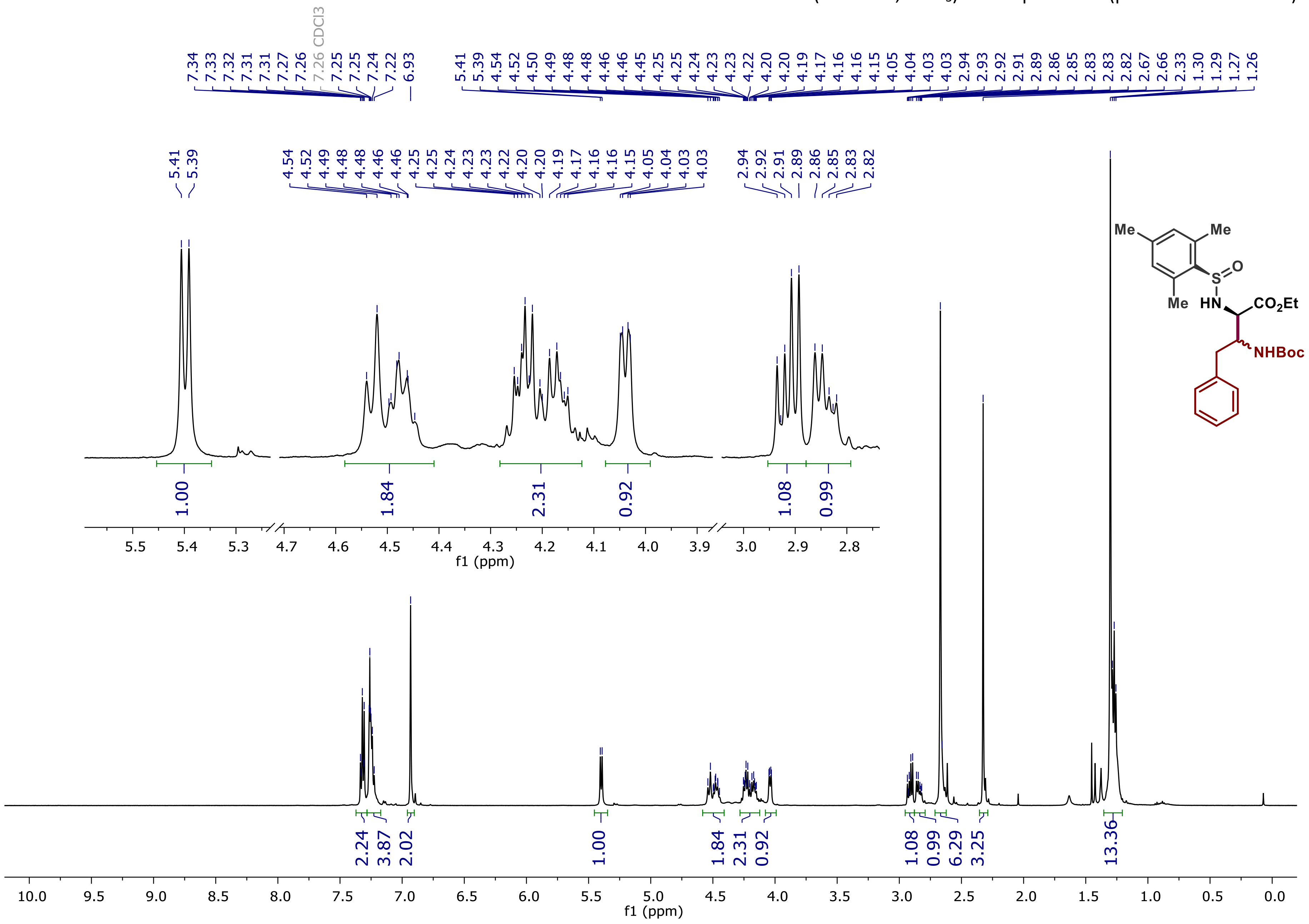

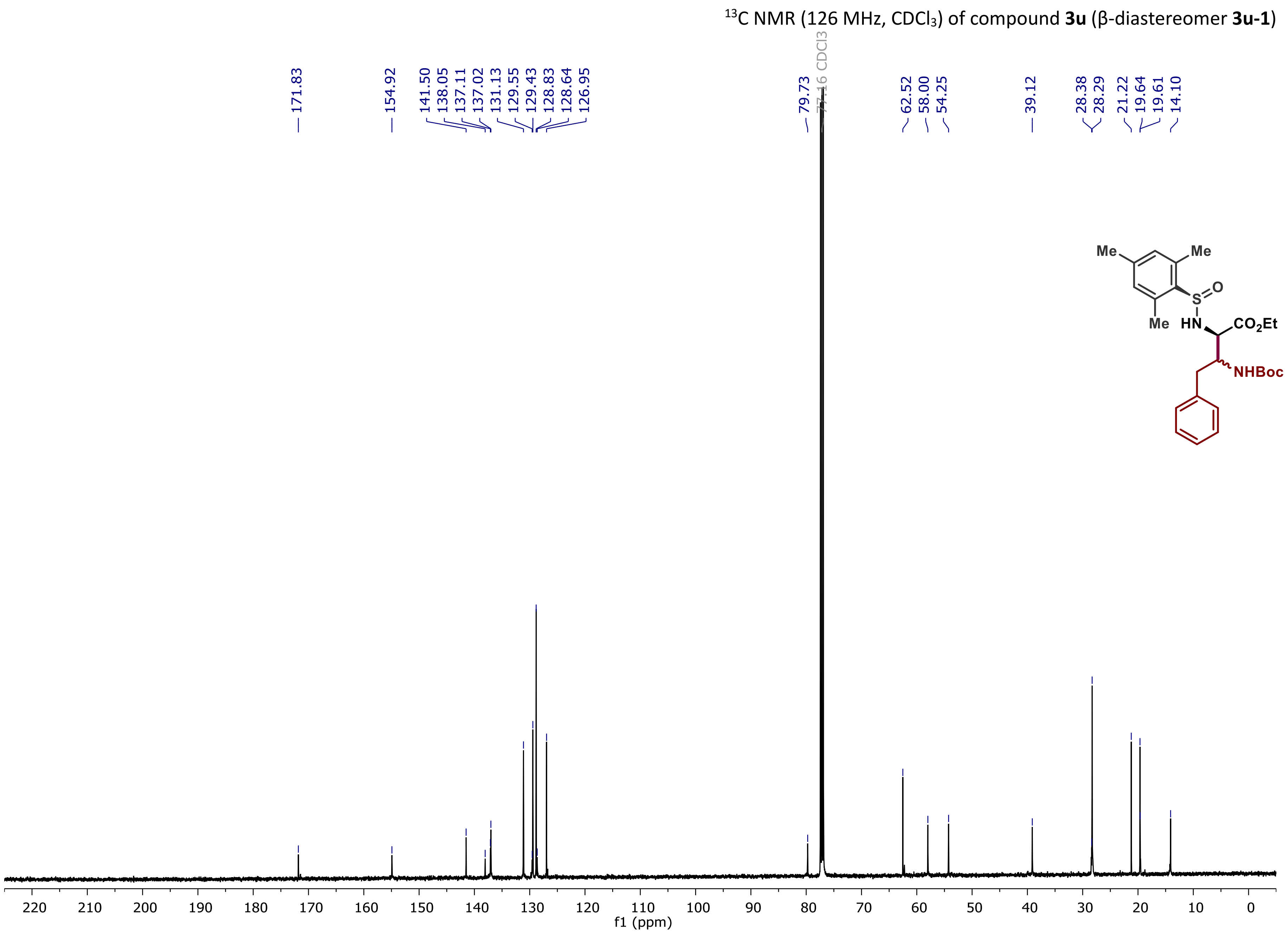

^{13}C NMR (126 MHz, $CDCl_3$) of compound **3u** (β-diastereomer **3u-1**)
171.83
154.92
141.50
138.05
137.11
137.02
131.13
129.55
129.43
128.83
128.64
126.95
79.73
77.16 CDCl3
62.52
58.00
54.25
39.12
28.38
28.29
21.22
19.64
19.61
14.10
Me
Me
Me
S
O
HN
CO_2Et
NHBoc
f1 (ppm)

^1H NMR (500 MHz, $CDCl_3$) of compound **3u** (β-diastereomers **3u-1**/**3u-2** 1:1)

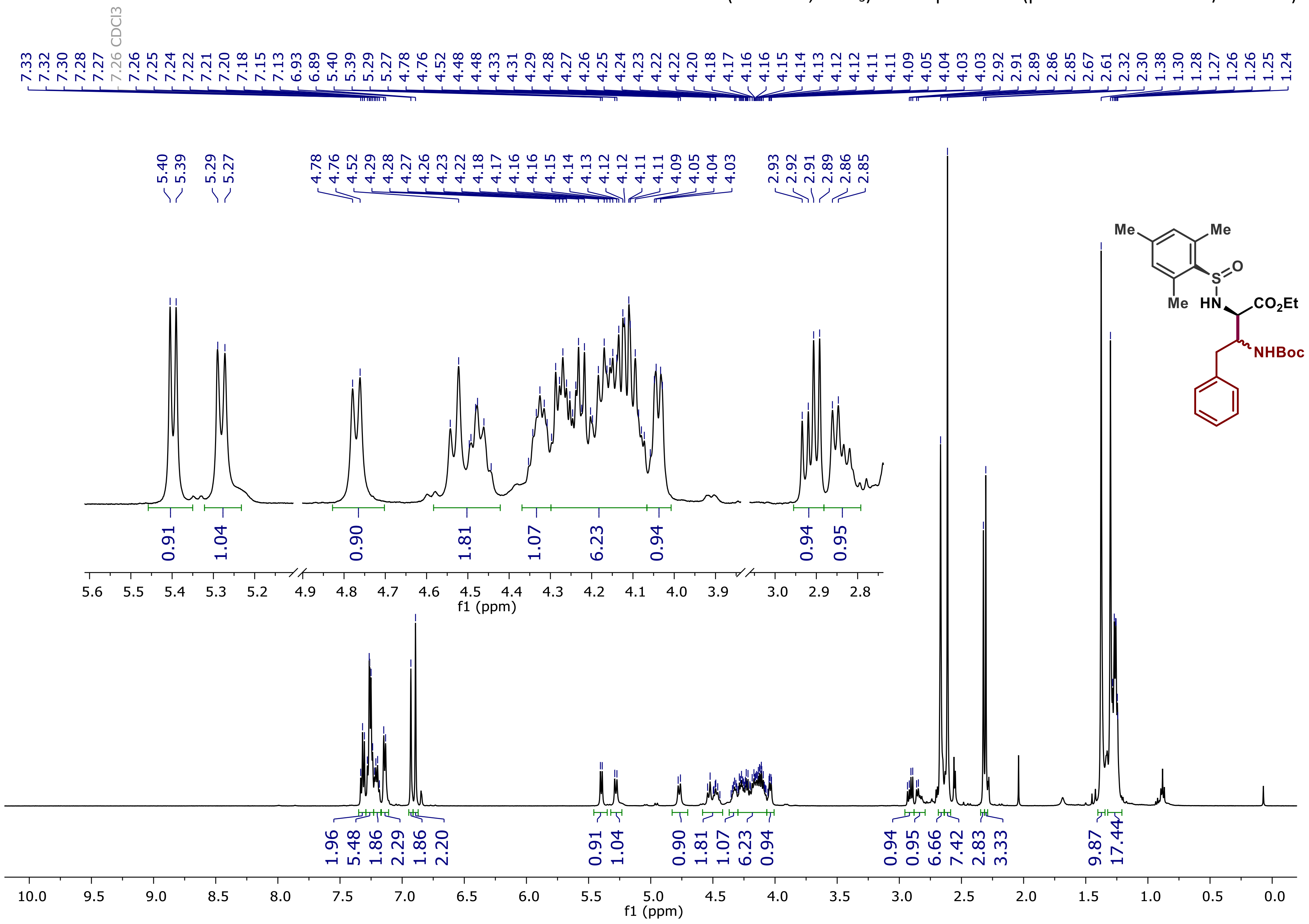

^{13}C NMR (126 MHz, $CDCl_3$) of compound **3u** (β-diastereomers **3u-1**/**3u-2** 1:1)

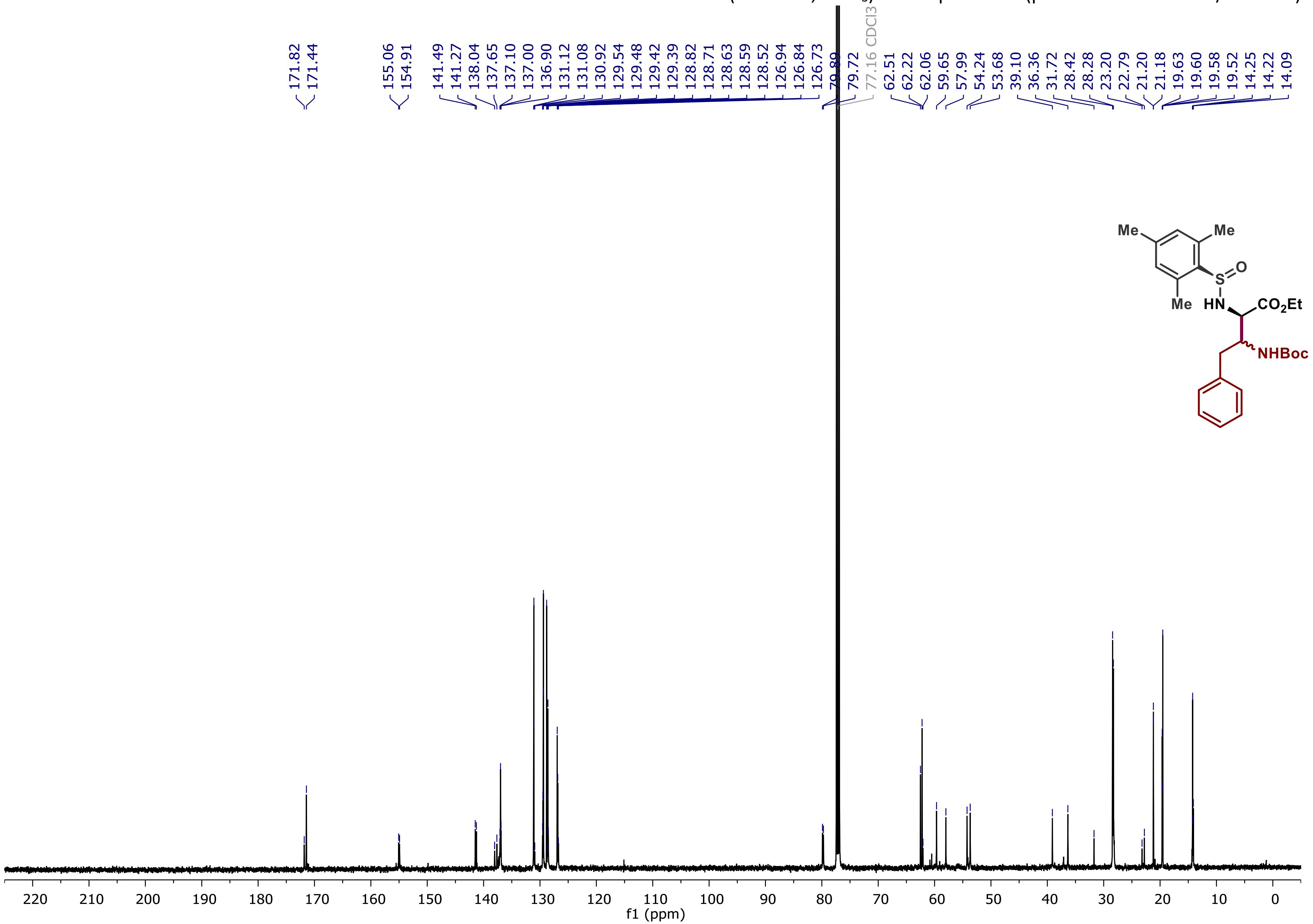

^{1}H NMR (500 MHz, $CDCl_3$) of compound **3v**

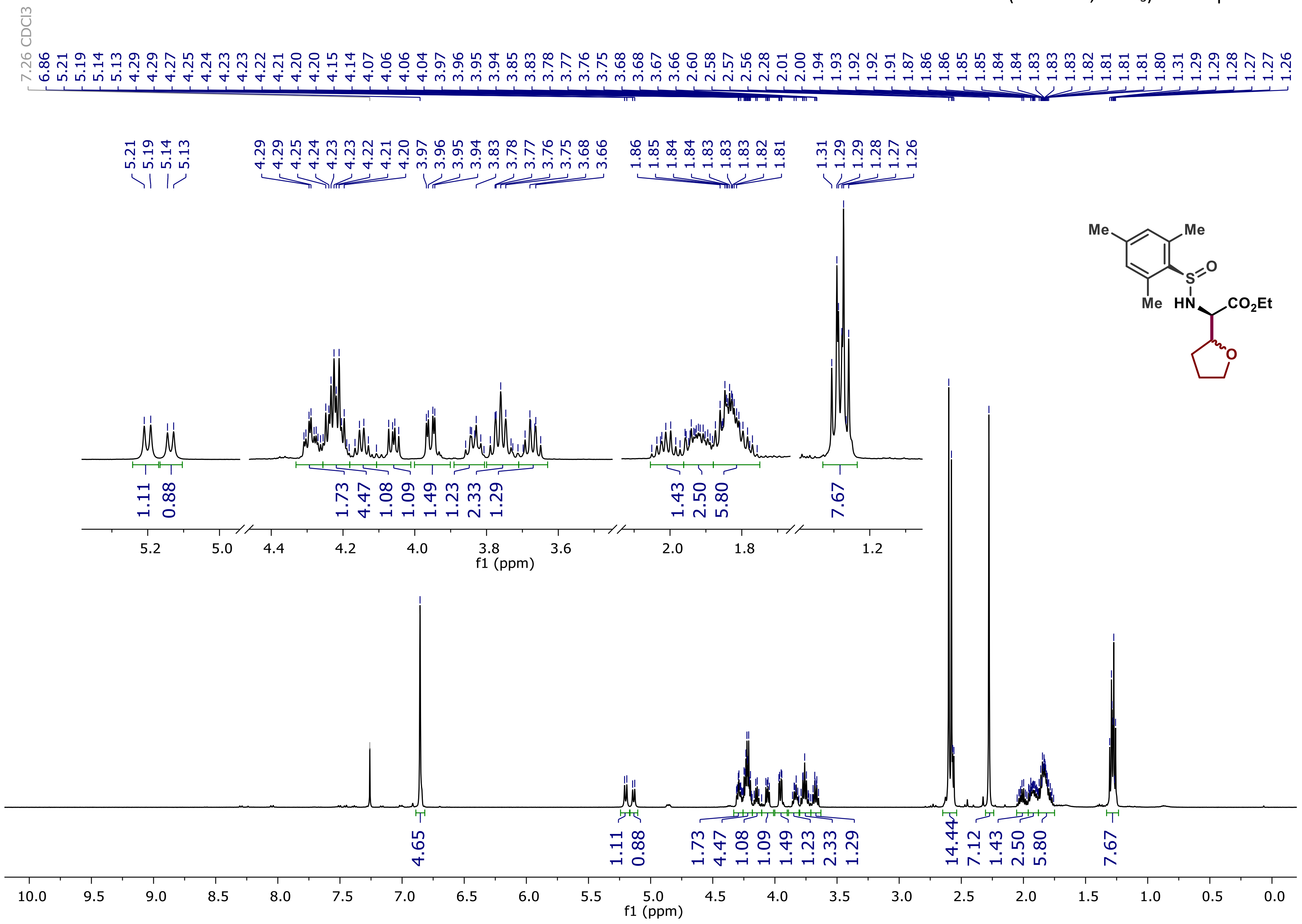

^{13}C NMR (126 MHz, $CDCl_3$) of compound **3v**

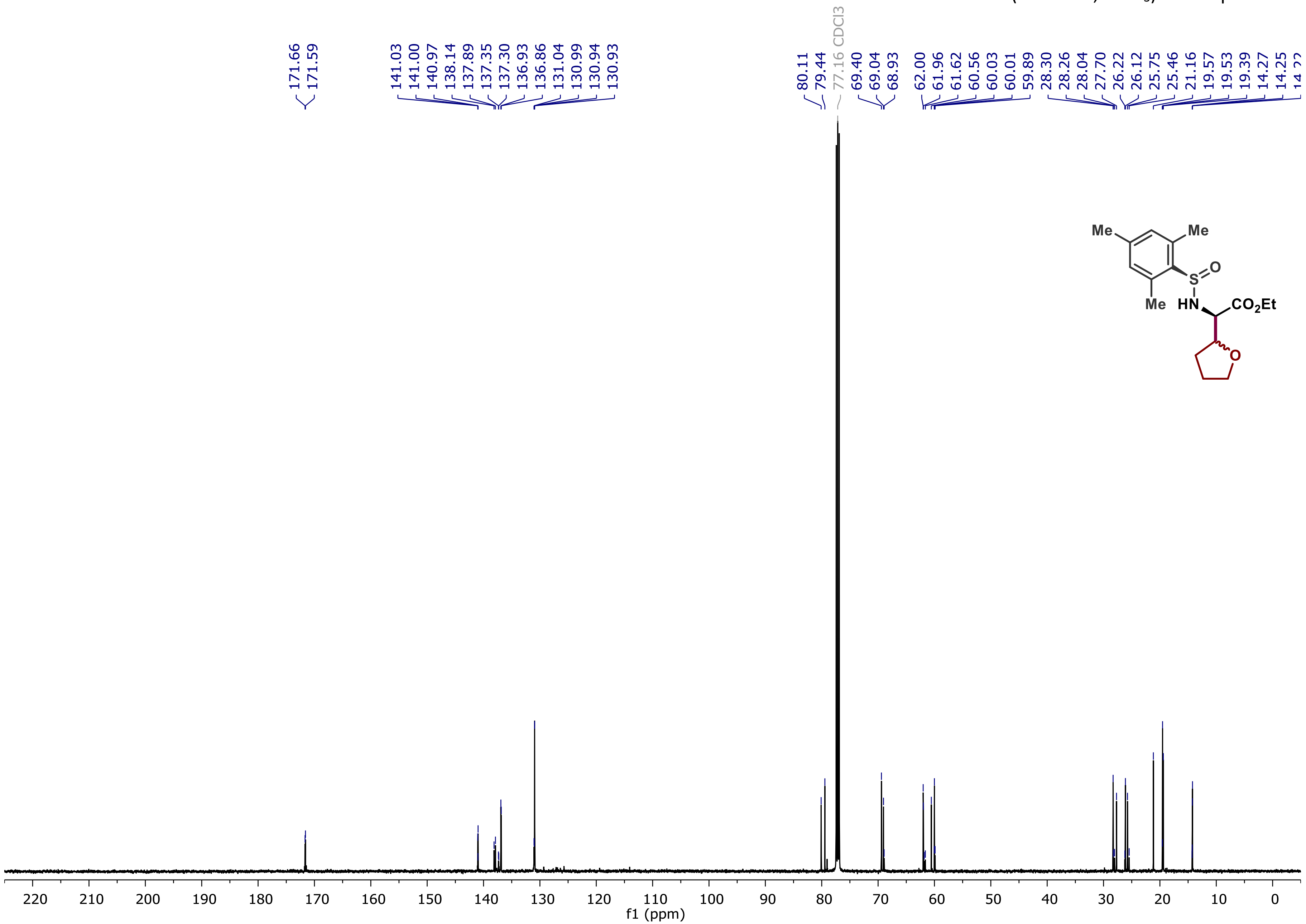

^{1}H NMR (500 MHz, $CDCl_3$) of compound **3w**

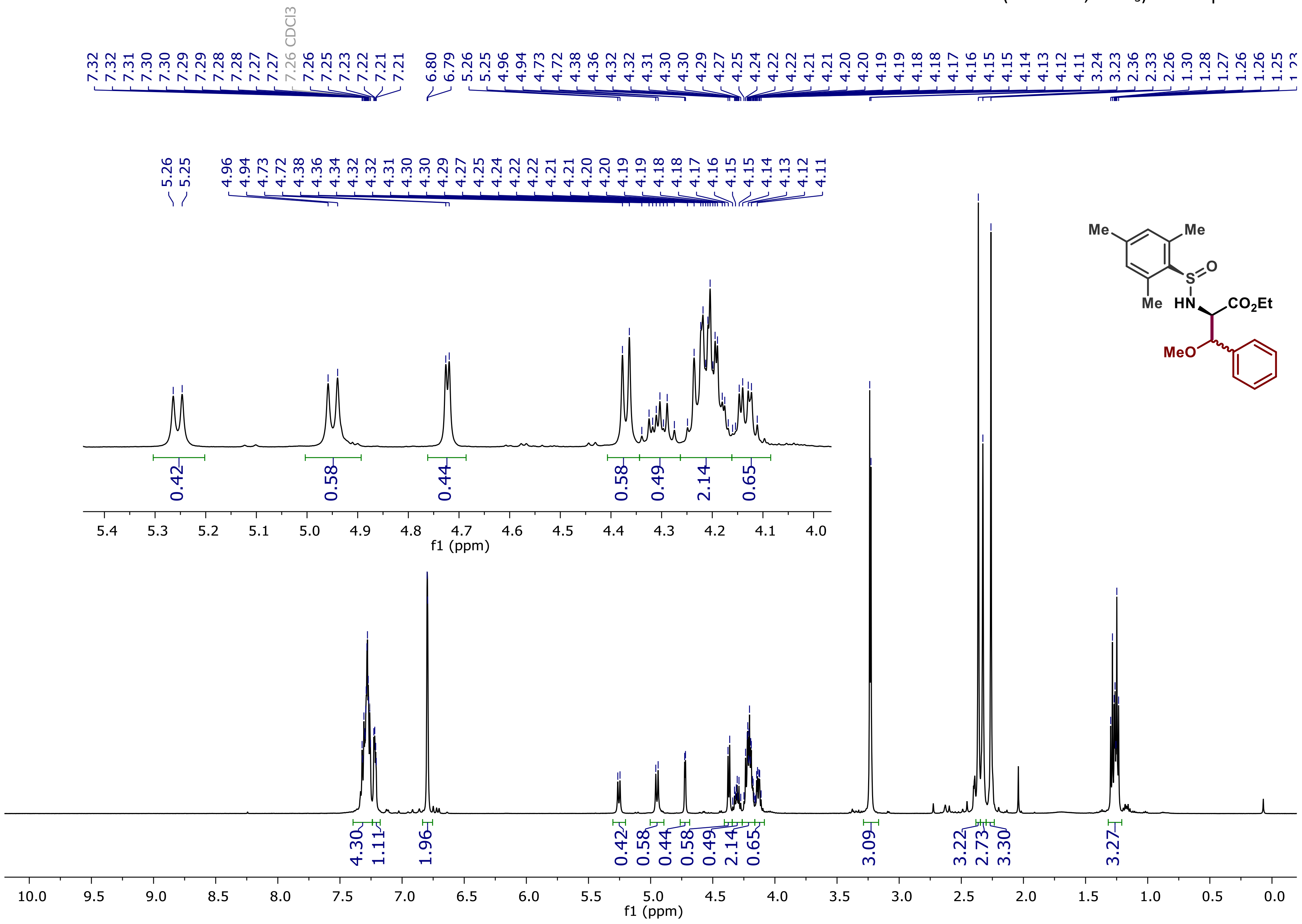

^{13}C NMR (126 MHz, $CDCl_3$) of compound **3w**

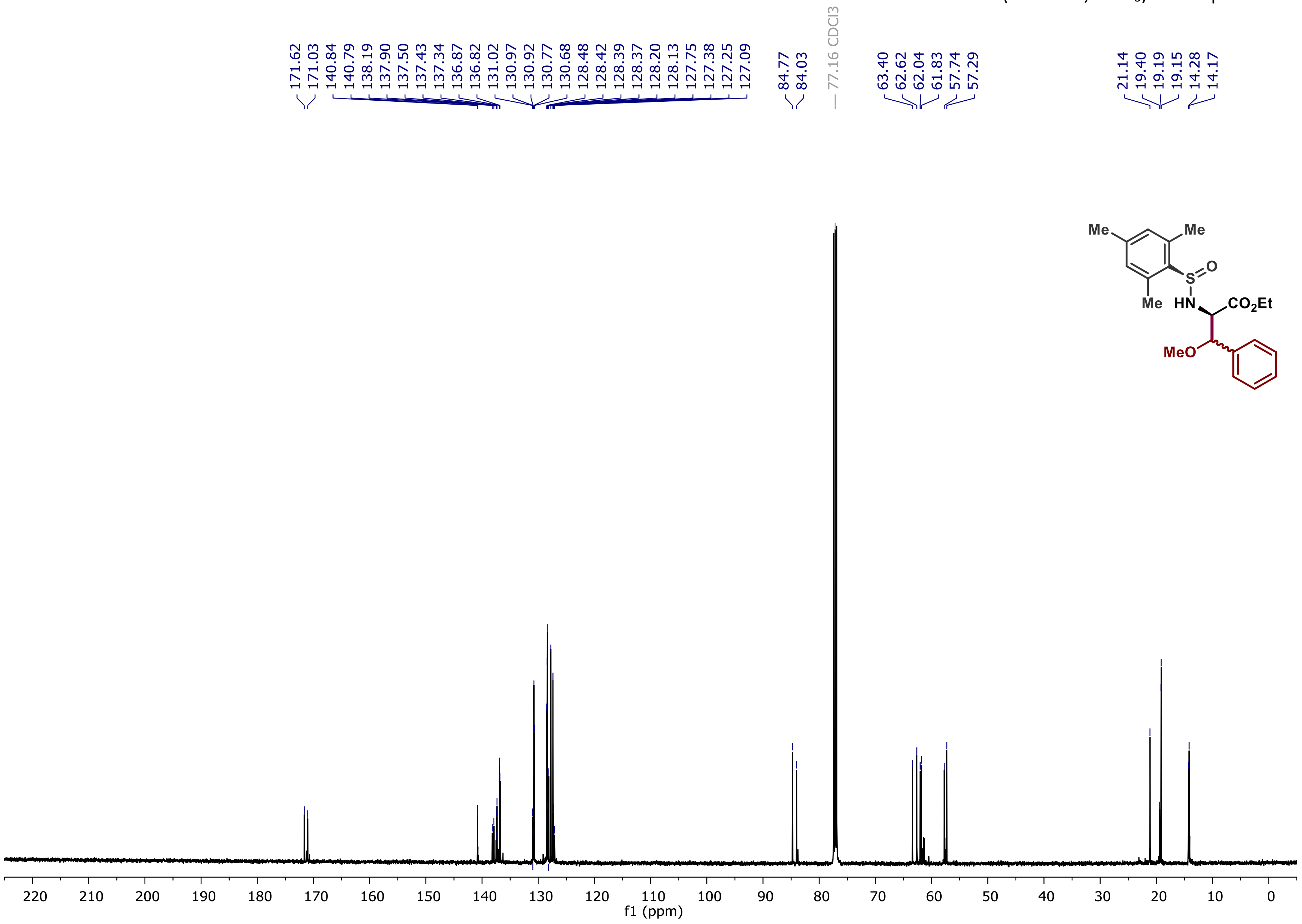

^{1}H NMR (500 MHz, $CDCl_3$) of compound **3x** (β-diastereomer **3x-1**)

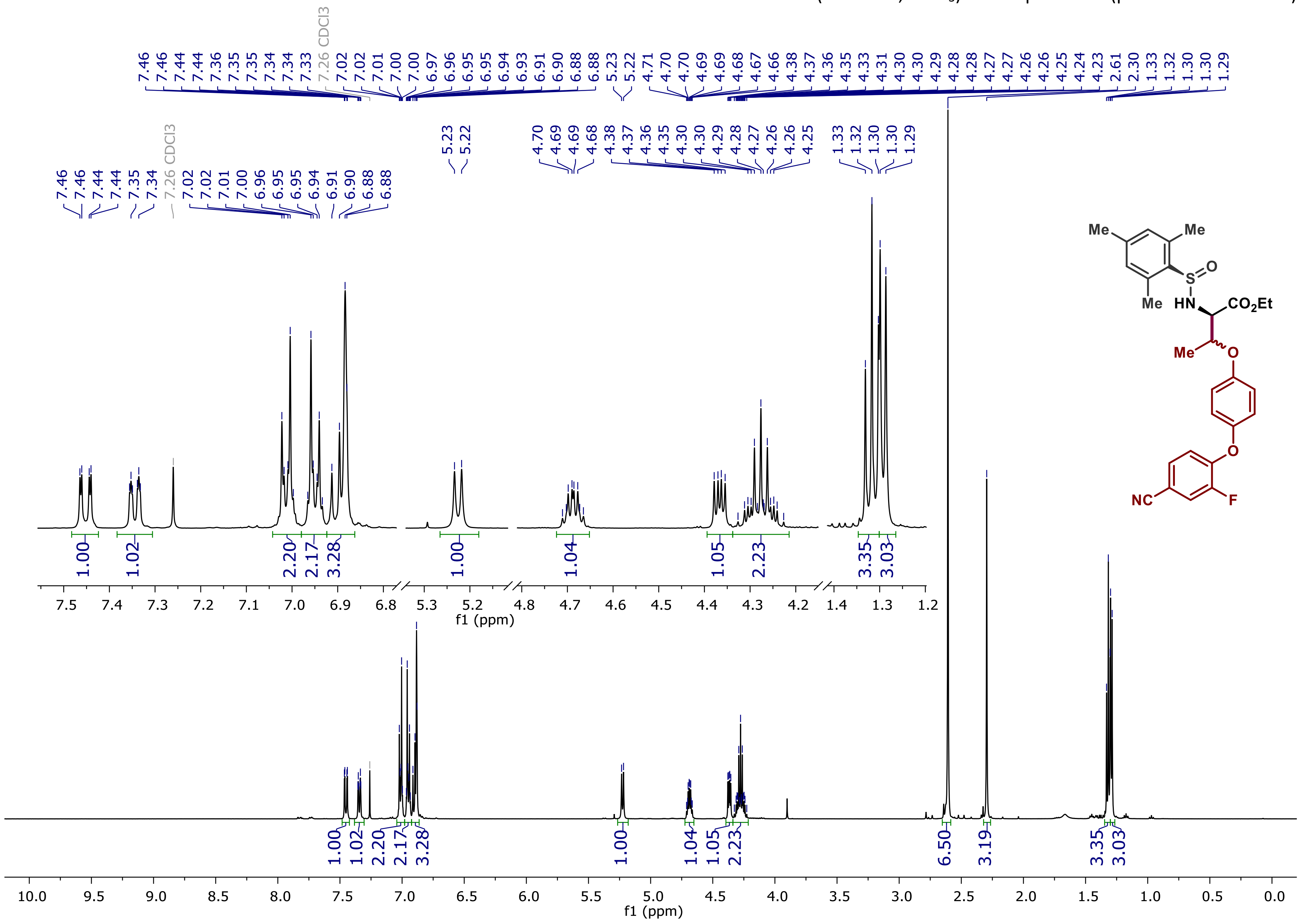

^{13}C NMR (126 MHz, $CDCl_3$) of compound **3x** (β-diastereomer **3x-1**)

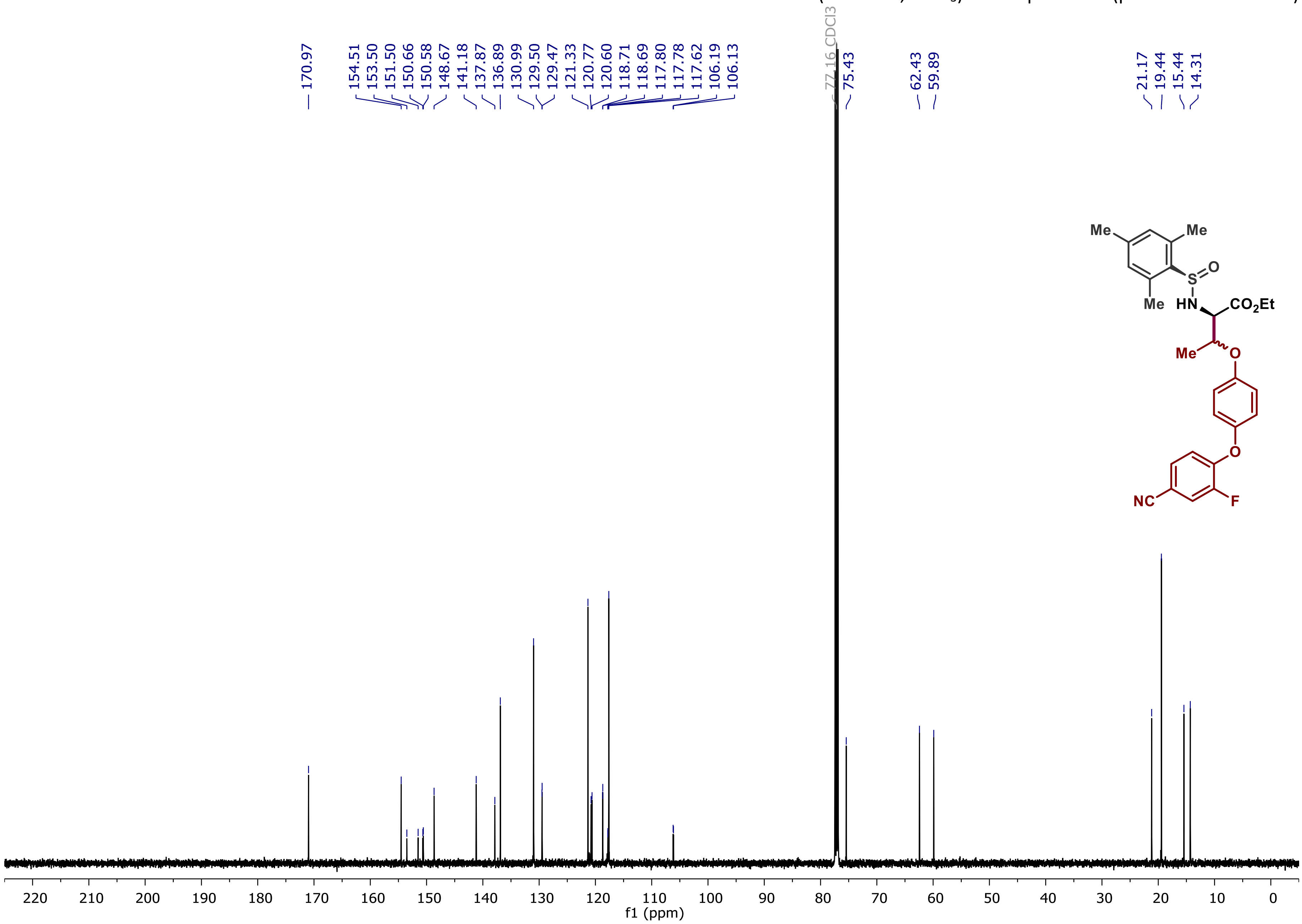

^{19}F NMR (377 MHz, $CDCl_3$) of compound **3x** (β-diastereomer **3x-1**)

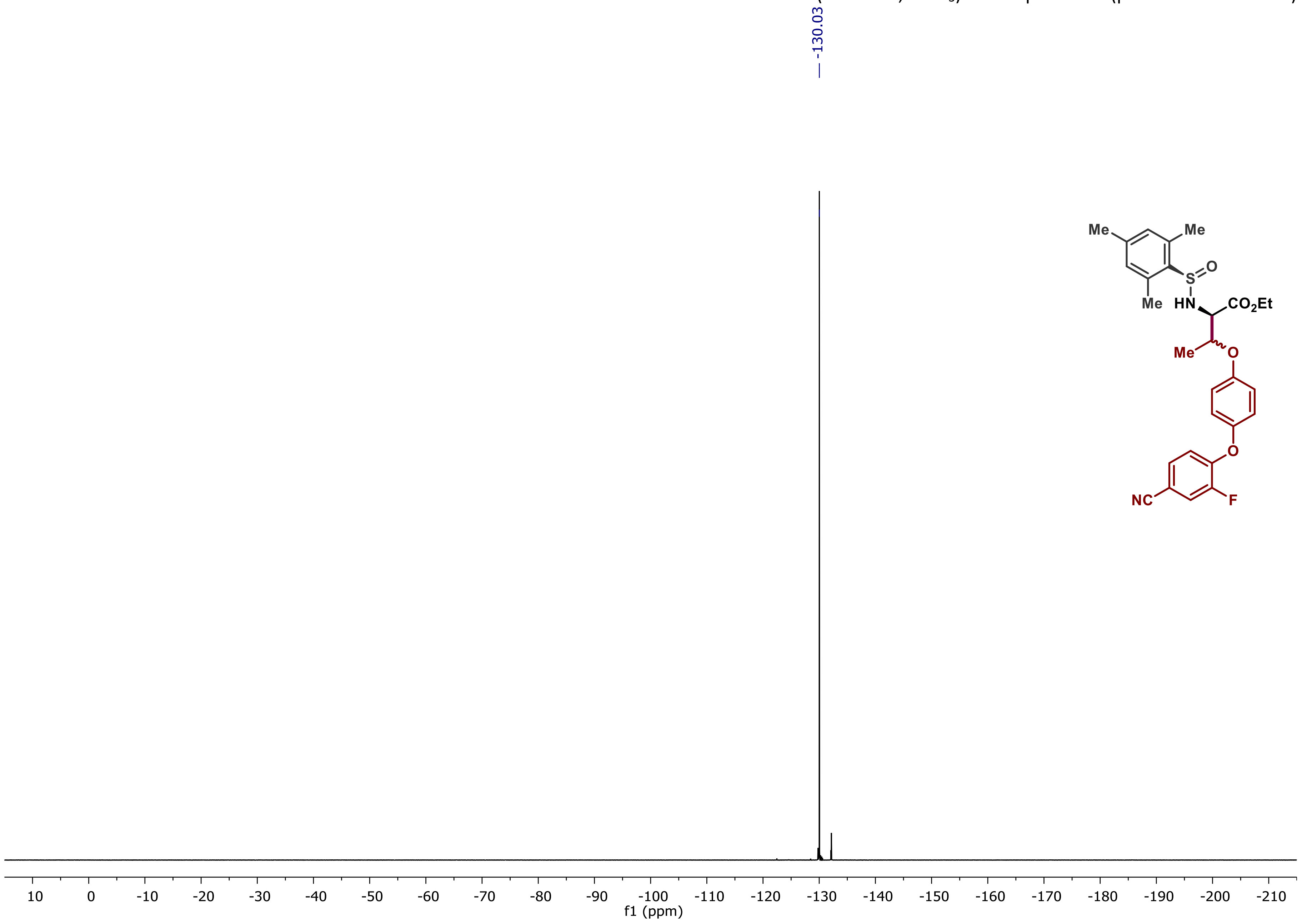

^{1}H NMR (500 MHz, $CDCl_3$) of compound **3x** (β-diastereomer **3x-2**)

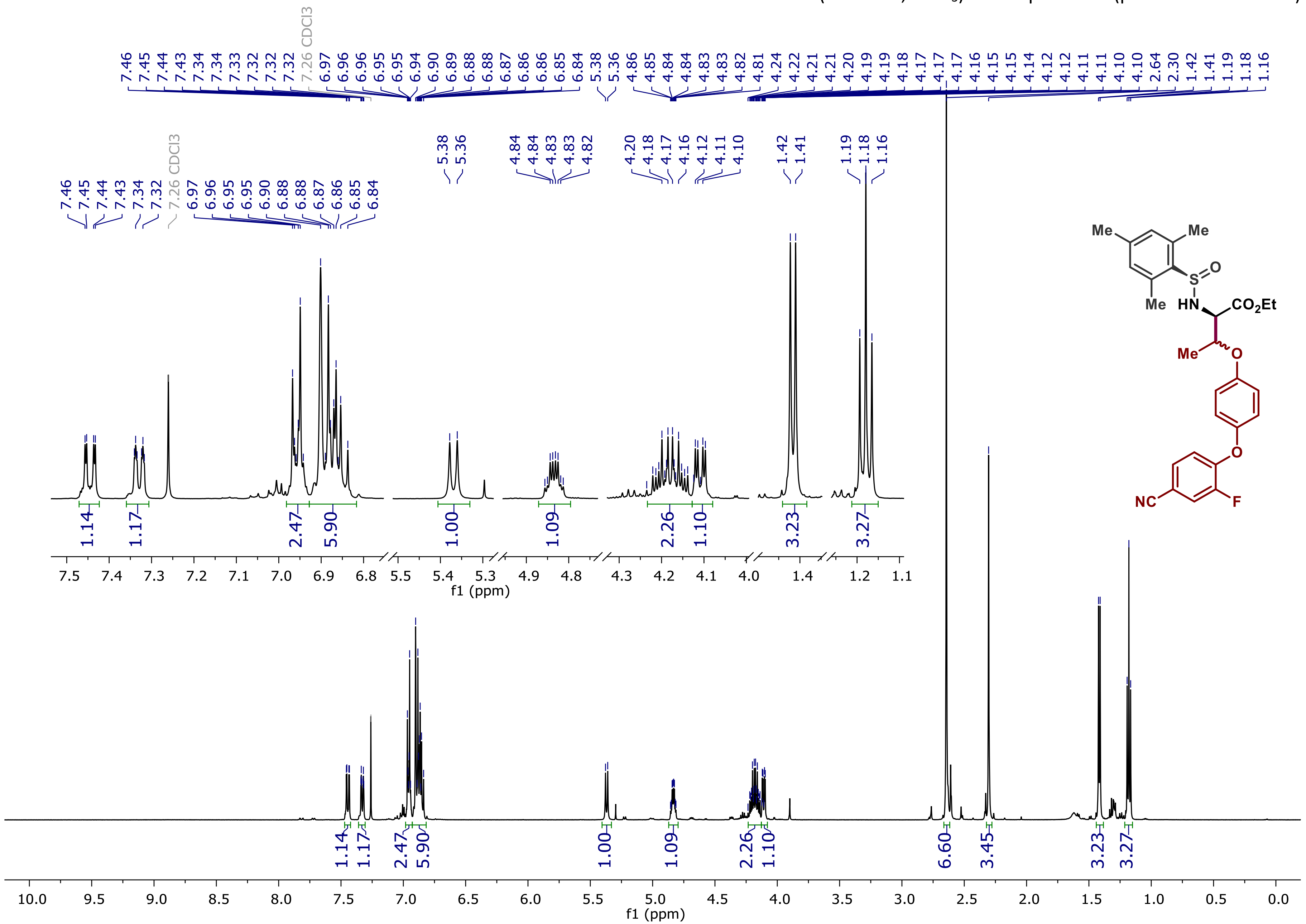

^{13}C NMR (126 MHz, $CDCl_3$) of compound **3x** (β-diastereomer **3x-2**)

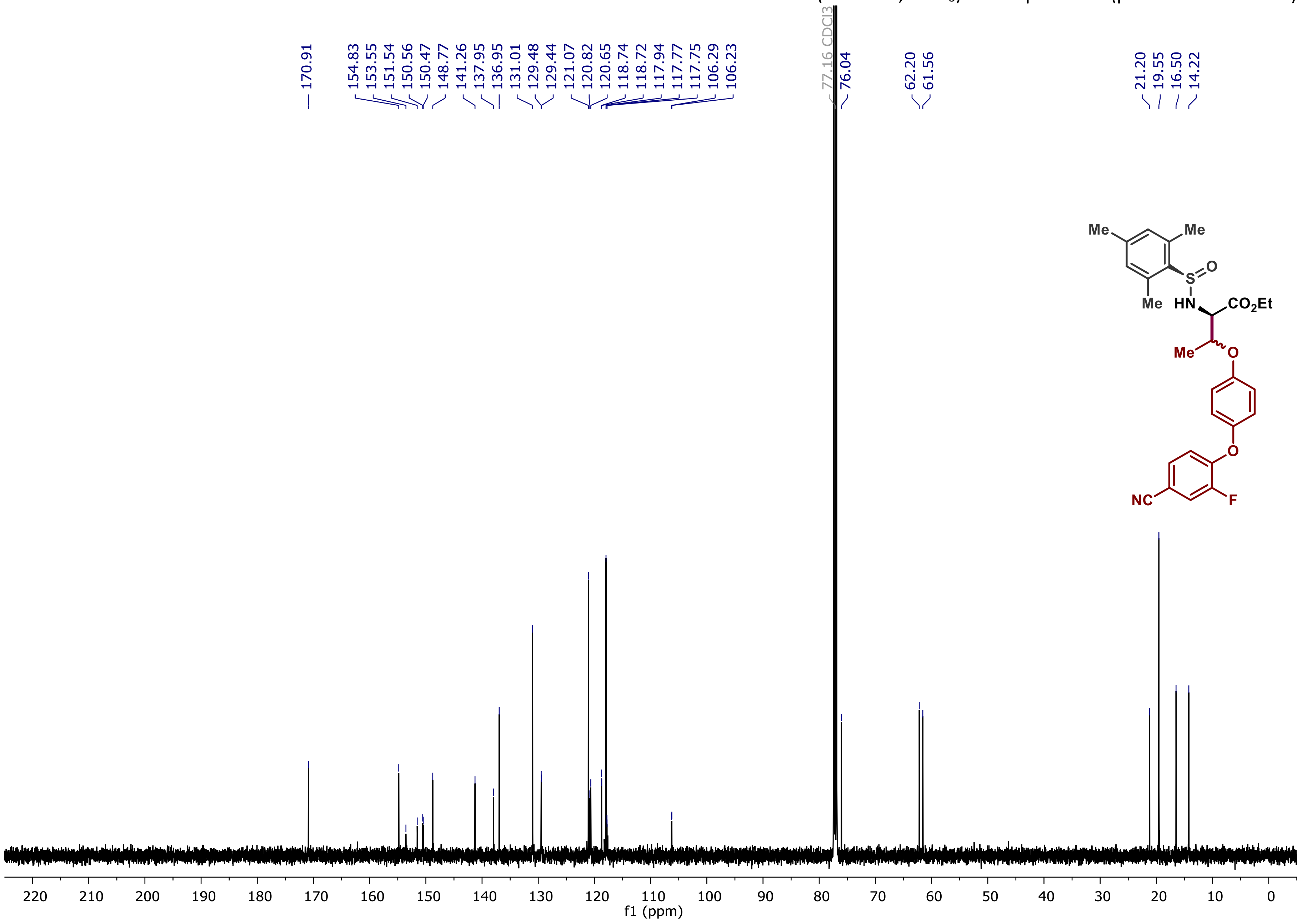

^{19}F NMR (377 MHz, $CDCl_3$) of compound **3x** (β-diastereomer **3x-2**)

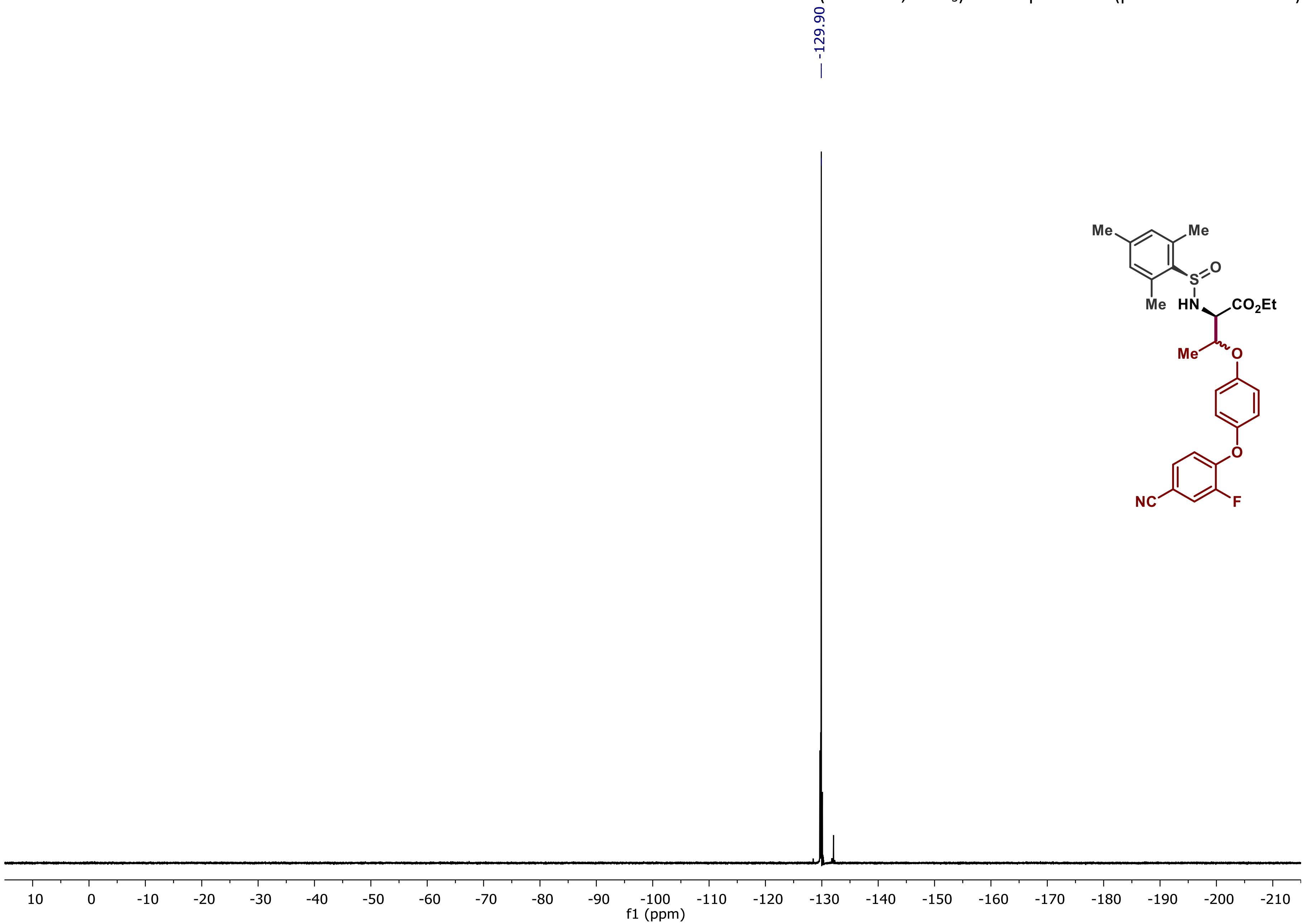

^{1}H NMR (500 MHz, $CDCl_3$) of compound **6x**

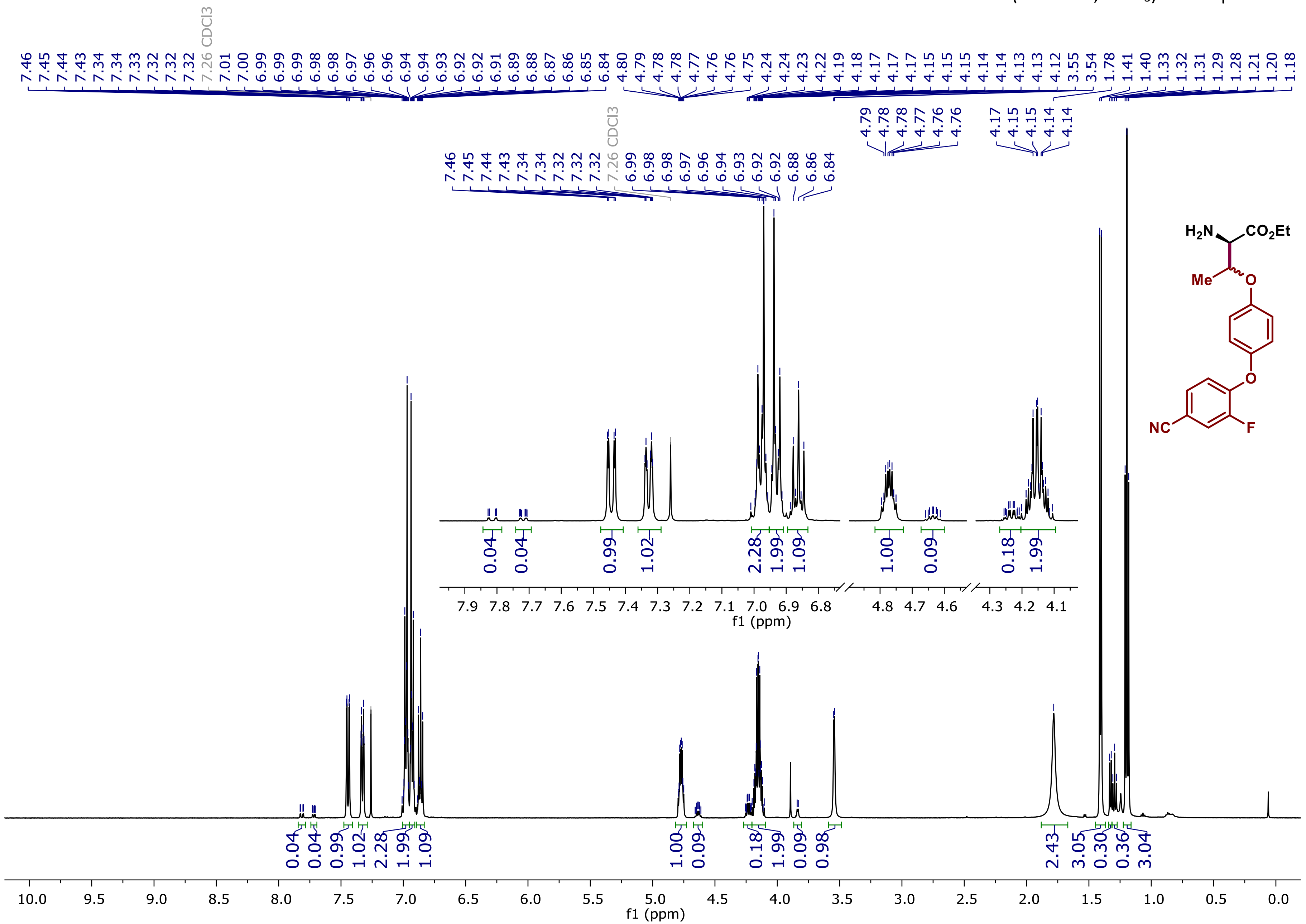

^{13}C NMR (126 MHz, $CDCl_3$) of compound **6x**

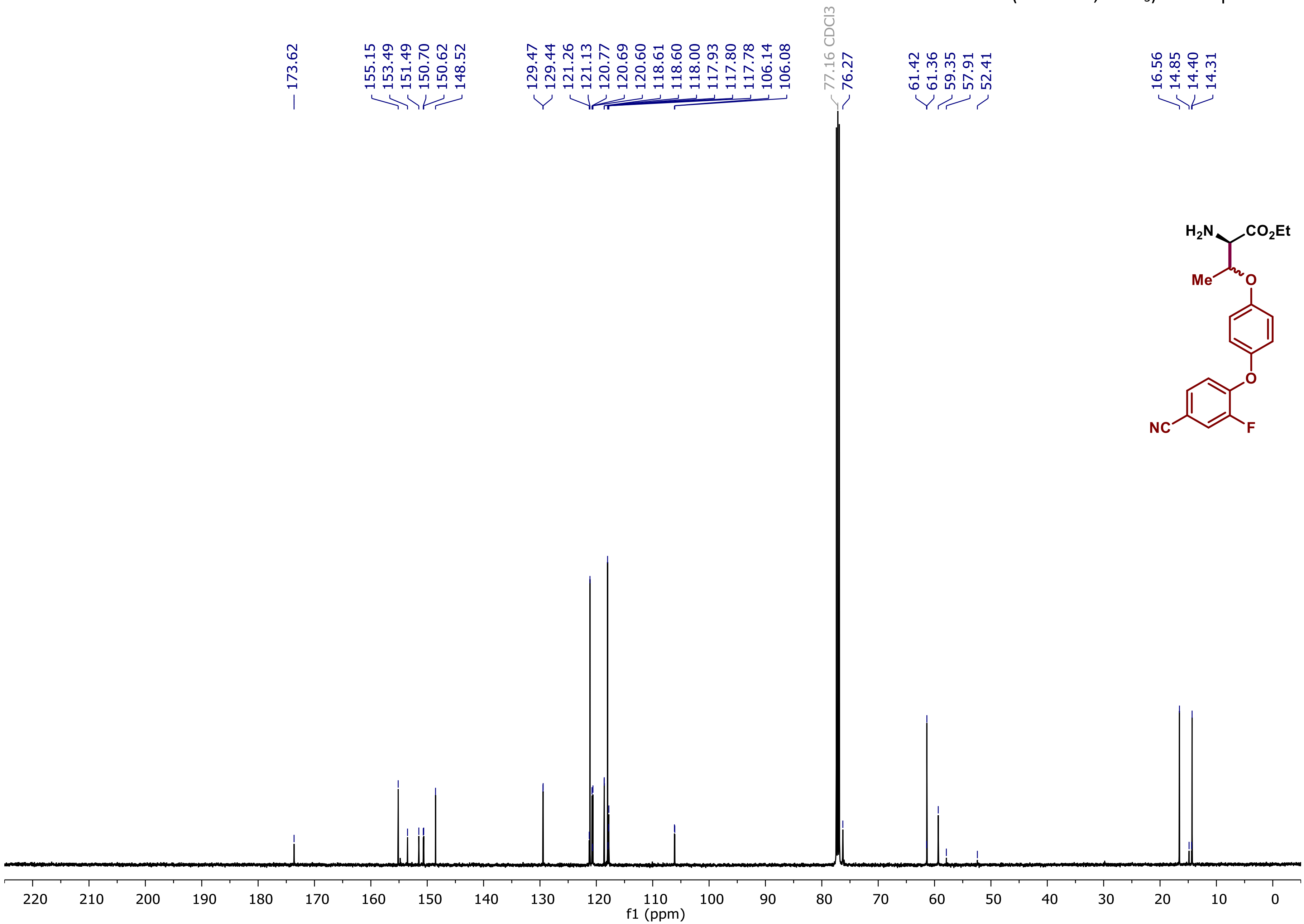

^{19}F NMR (377 MHz, $CDCl_3$) of compound **6x**

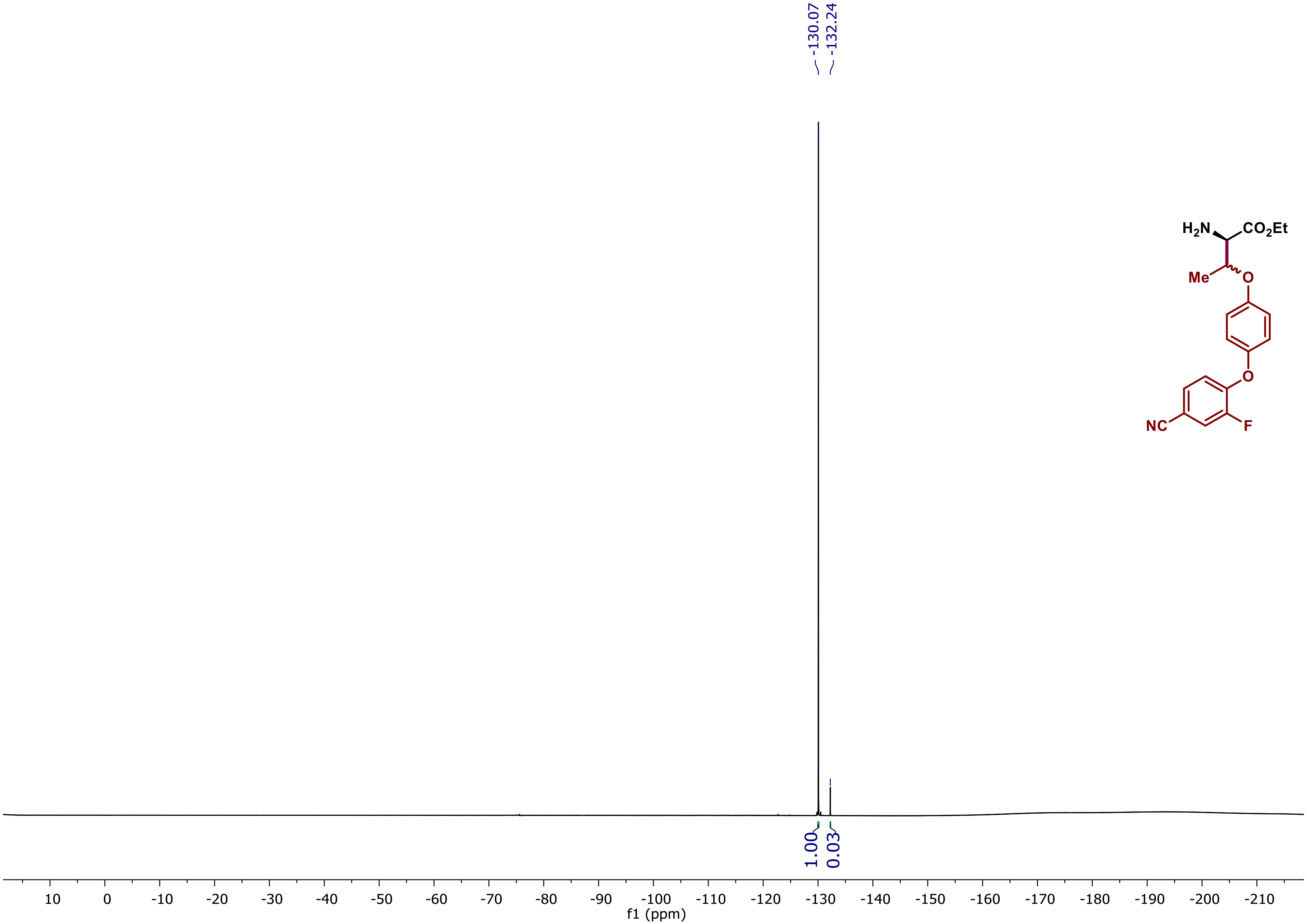

^1H NMR (500 MHz, $CDCl_3$) of compound **3y**

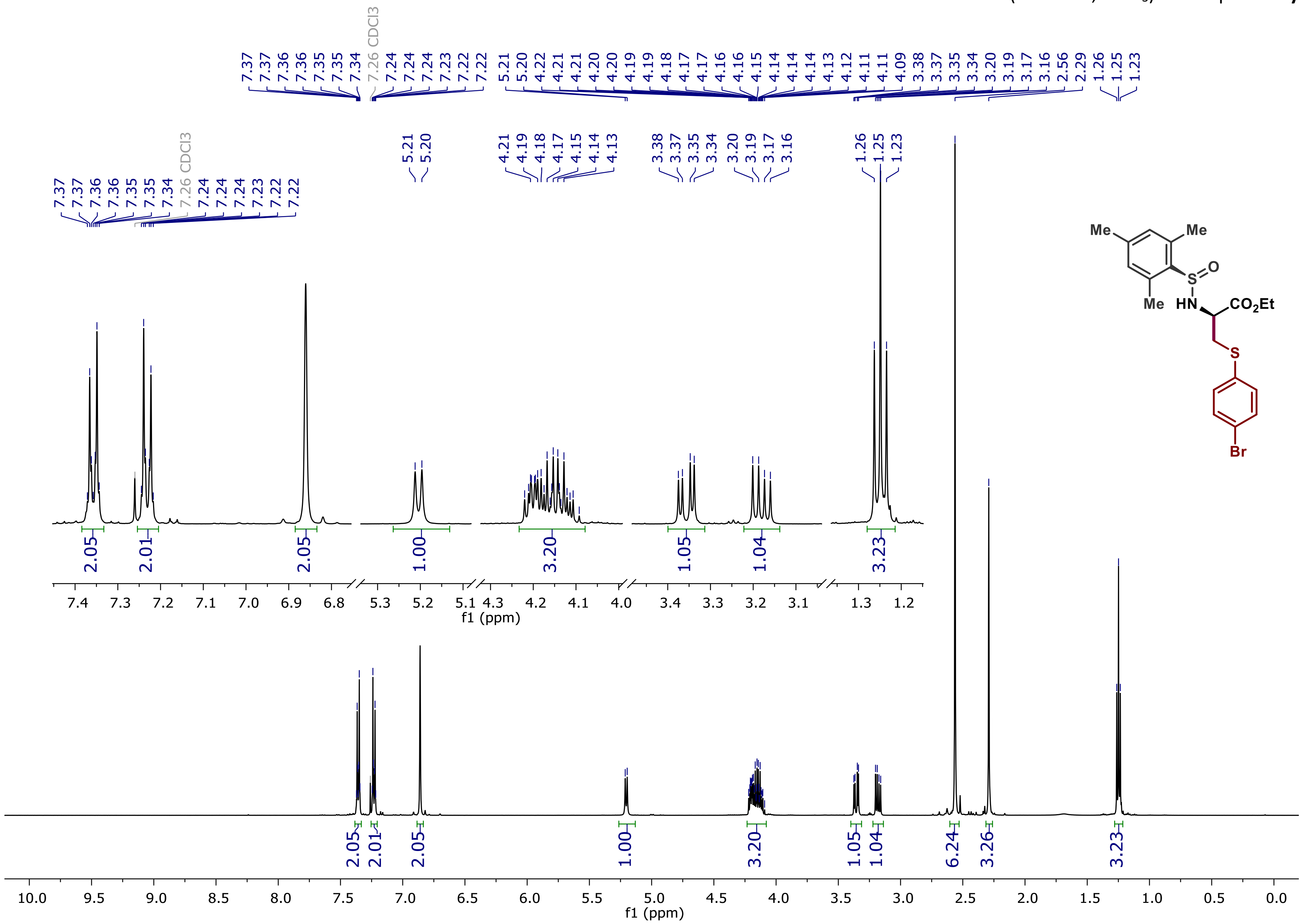

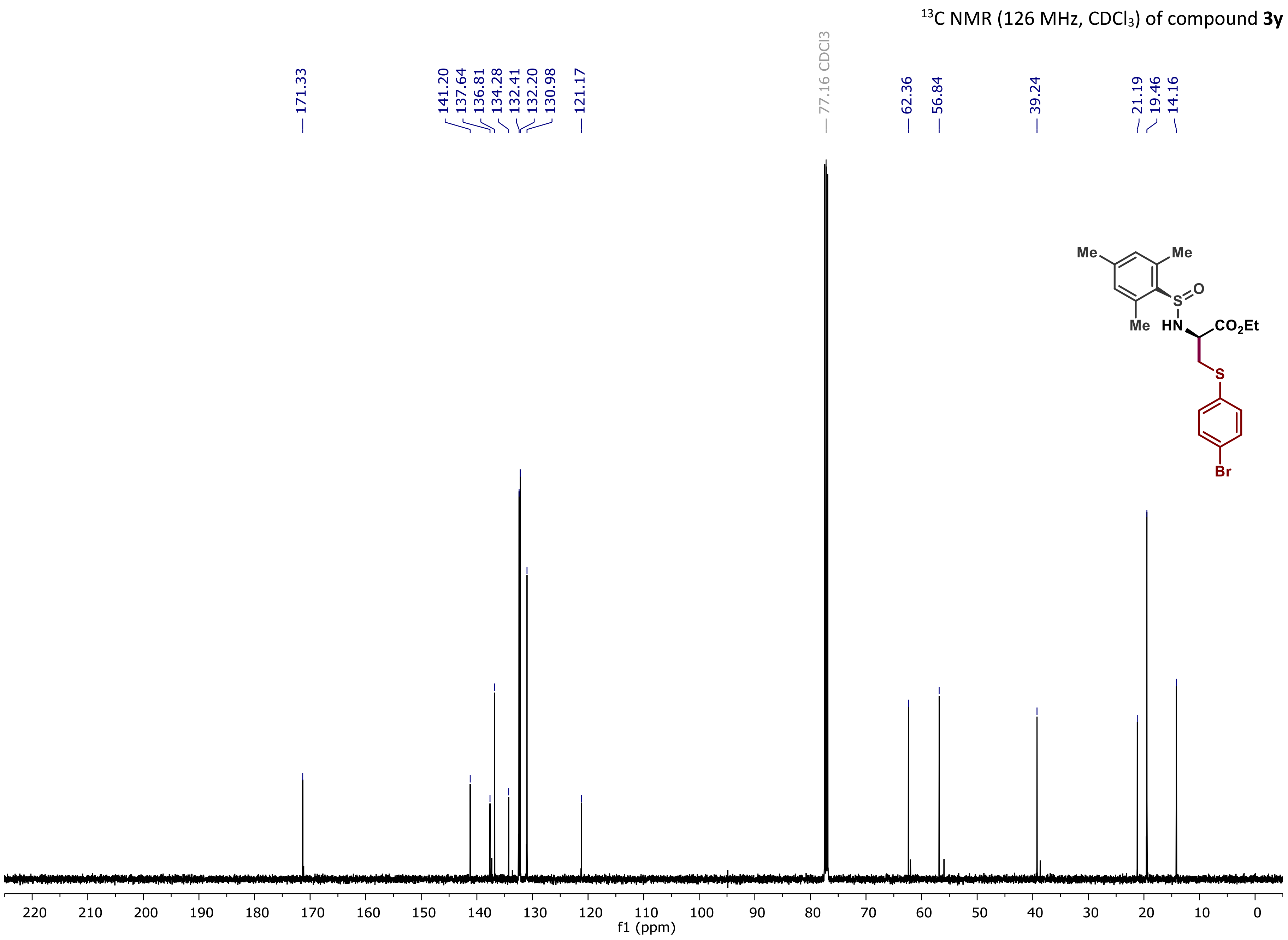

^{13}C NMR (126 MHz, CDCl$_3$) of compound **3y**
171.33
141.20
137.64
136.81
134.28
132.41
132.20
130.98
121.17
77.16 CDCl3
62.36
56.84
39.24
21.19
19.46
14.16
Me
Me
Me
S
O
HN
CO2Et
S
Br
220 210 200 190 180 170 160 150 140 130 120 110 100 90 80 70 60 50 40 30 20 10 0
f1 (ppm)

^1H NMR (500 MHz, $CDCl_3$) of compound **3z**

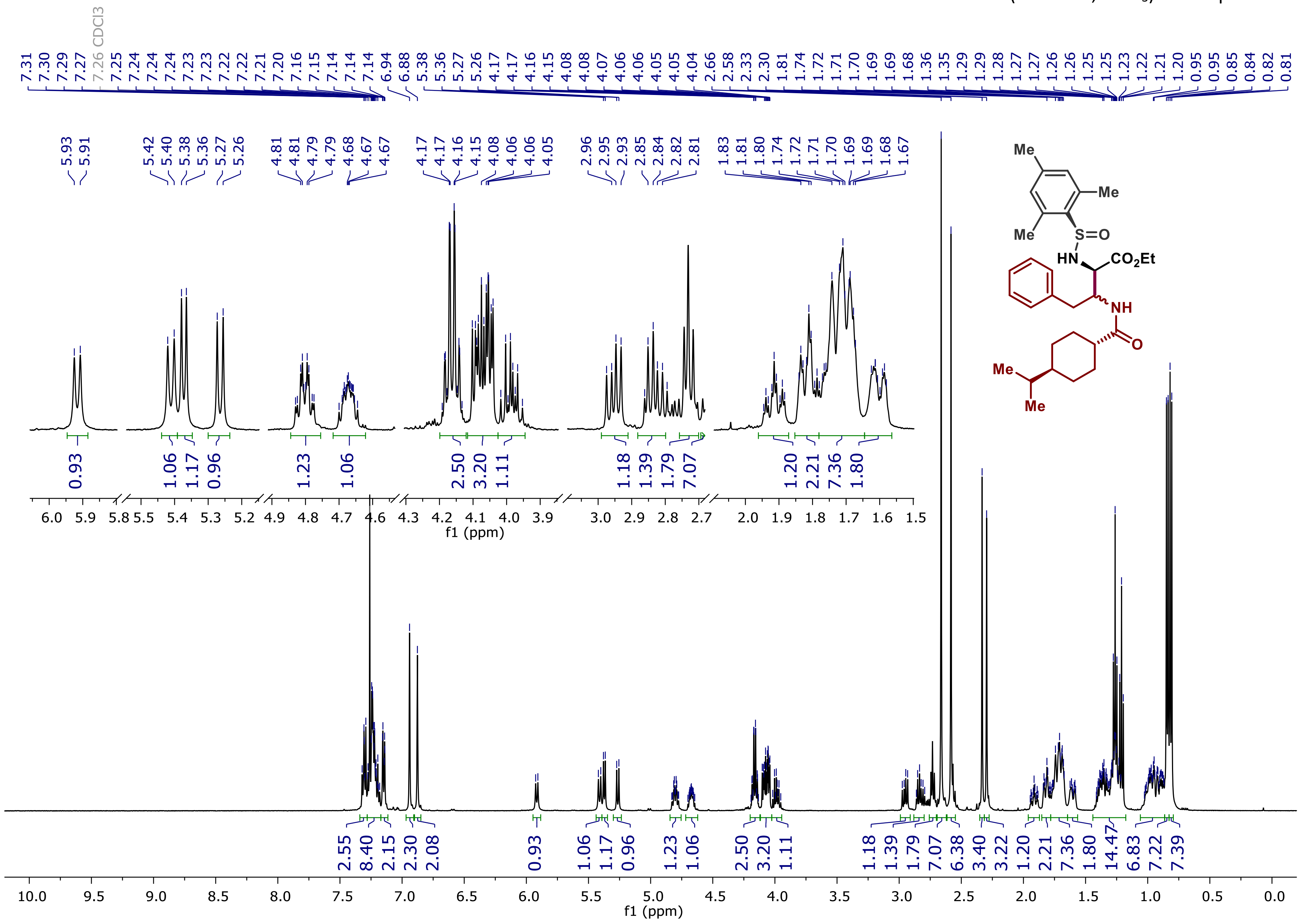

^{13}C NMR (126 MHz, $CDCl_3$) of compound **3z**

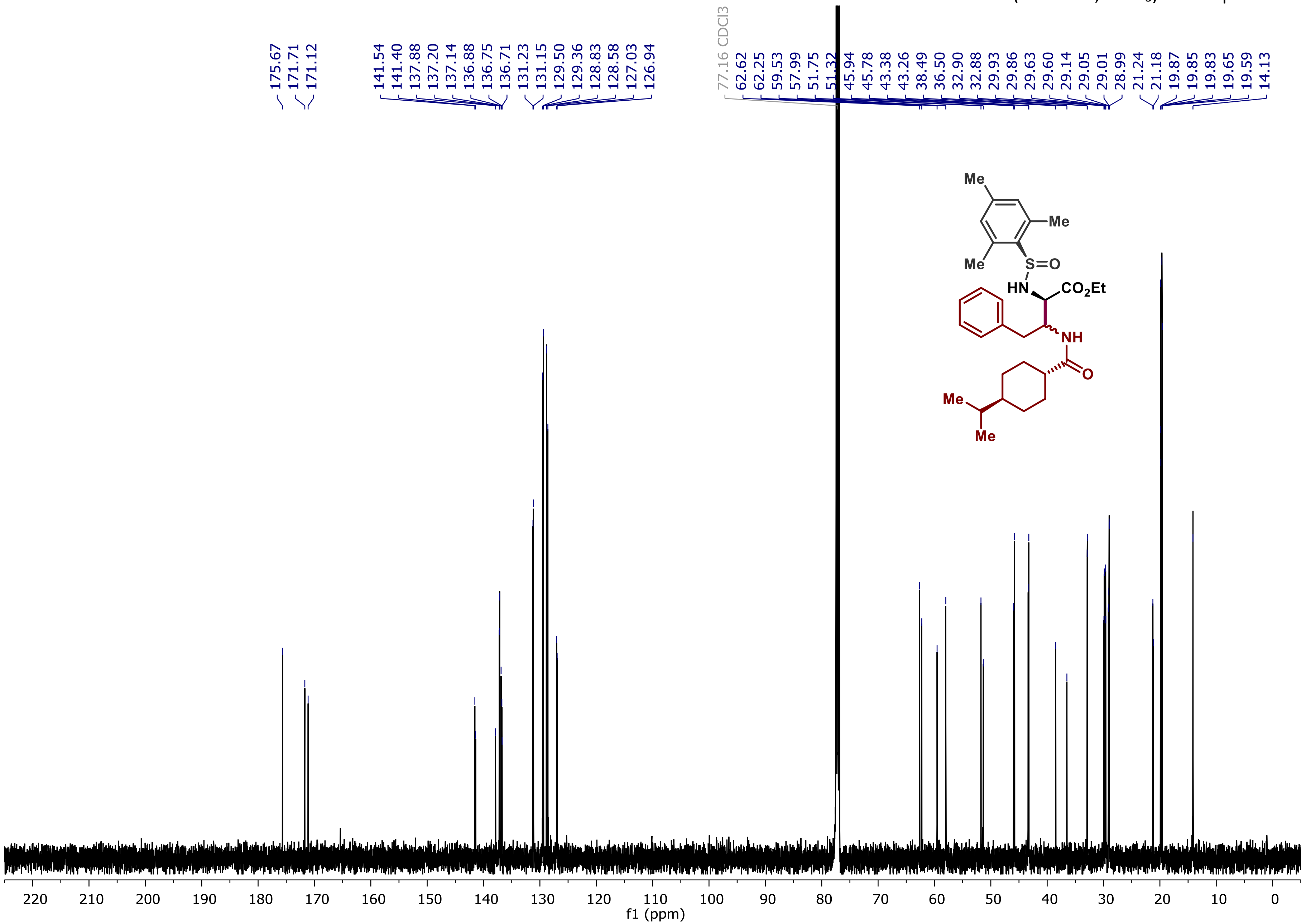

^{1}H NMR (500 MHz, $CDCl_3$) of compound **6z**

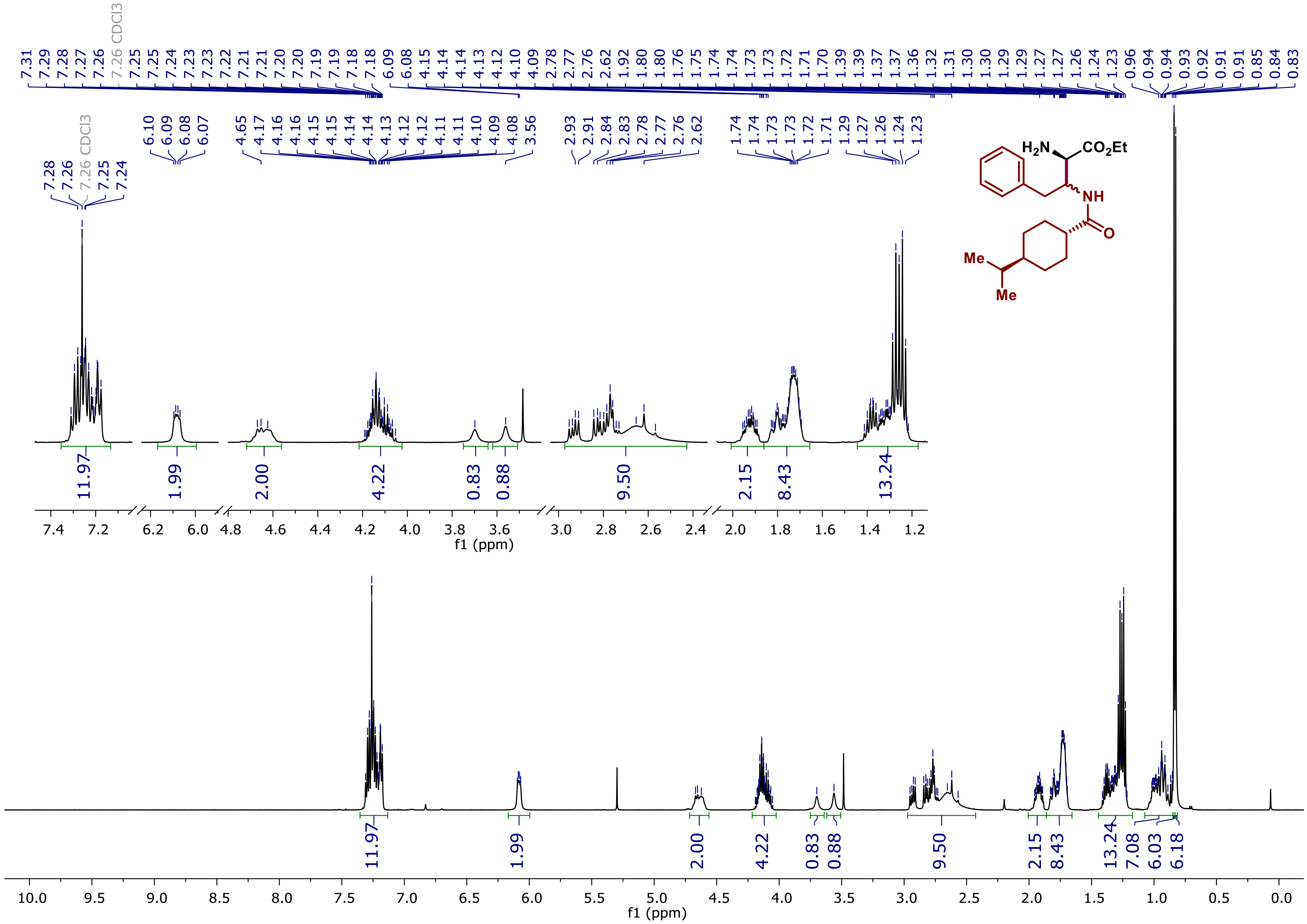

^{13}C NMR (126 MHz, $CDCl_3$) of compound **3z**

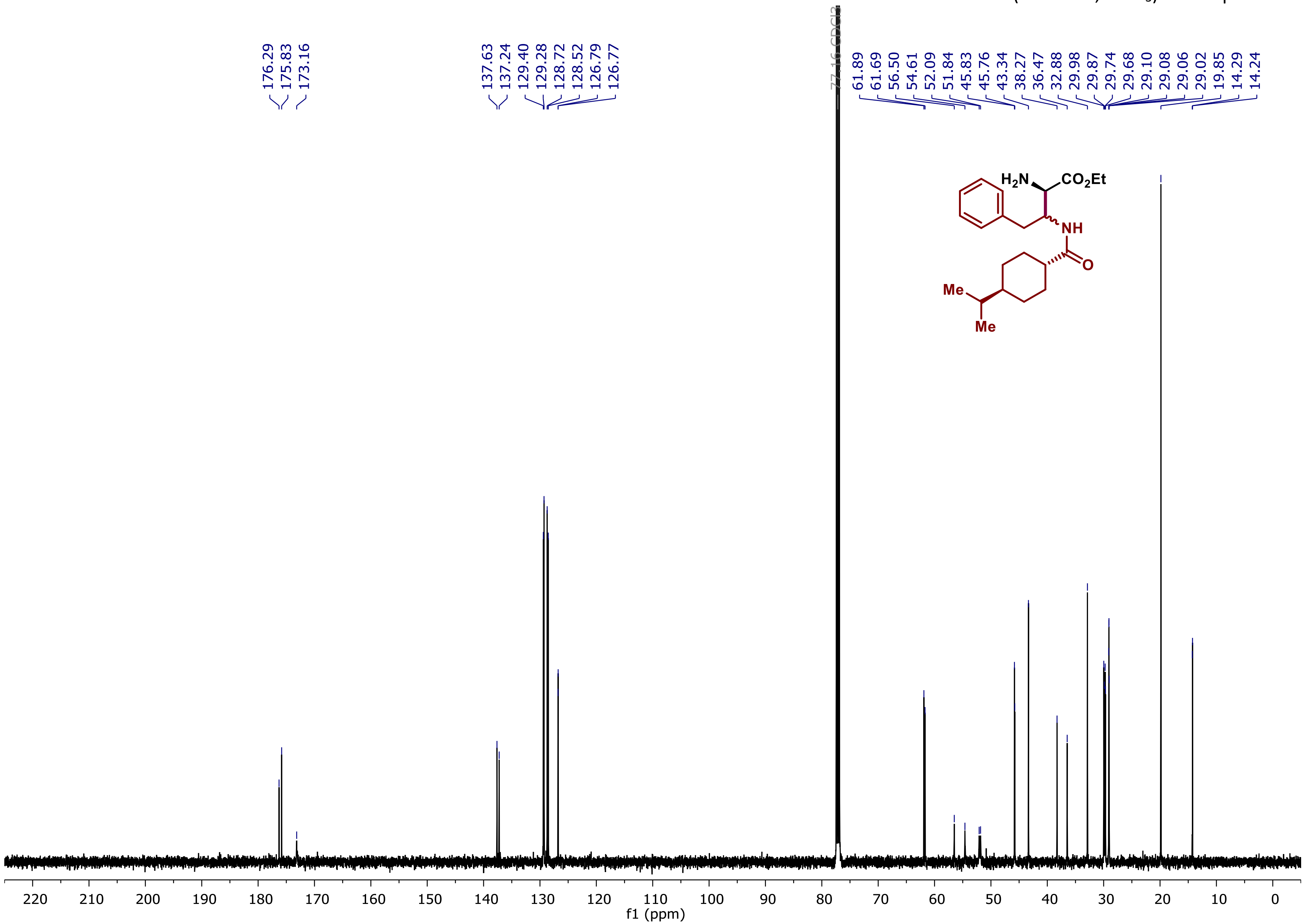

11. *Cartesian coordinates and energies*

The electronic energies (E), thermal correction to Gibbs free energy (ΔG_{corr}) and thermal correction to enthalpy (ΔH_{corr}) were calculated at the uM062X-D3/6-311+G(d,p)/SMD(chlorobenzene) level of theory. For ***re*-TS** and ***si*-TS** the single point electronic energies were also recalculated using the uB3LYP-D3 functional (EB3LYP-D3) and the uwB97XD (EwB97XD) level of theory including SMD solvation (chlorobenzene). The standard Gibbs free energy ($\Delta G°$) is obtained by the following $\Delta G° = E + \Delta G_{corr} + 0.0003027843$, where 0.003027843 is an entropic term to account for the change in the standard state (1 atm → 1 M). The standard enthalpy ($H°$) is obtained by the following: $H° = E + \Delta H_{corr}$. All energies are given in Hartree.

tert-butyl radical

Charge: 0

Multiplicity: 2

E = −157.749516456

ΔG_{corr} = 0.088724

ΔH_{corr} = 0.124235

$\Delta G°$ = −157.6577646

Cartesian coordinates:

C	0.0000000000	0.0000000000	-0.2013590000
C	0.0000000000	1.4774920000	0.0205060000
H	0.0000000000	1.7165170000	1.0971560000
H	0.8874690000	1.9509610000	-0.4087350000
H	-0.8874690000	1.9509610000	-0.4087350000
C	-1.2795450000	-0.7387460000	0.0205060000
H	-2.1333170000	-0.2069090000	-0.4087350000
H	-1.2458470000	-1.7440520000	-0.4087350000
H	-1.4865470000	-0.8582580000	1.0971560000
C	1.2795450000	-0.7387460000	0.0205060000
H	1.2458470000	-1.7440520000	-0.4087350000
H	2.1333170000	-0.2069090000	-0.4087350000
H	1.4865470000	-0.8582580000	1.0971560000

There are no imaginary frequencies

s-cis conformer of N-sulfinyl imine 1

Charge: 0

Multiplicity: 1

E = −1184.08398538

ΔG_{corr} = 0.232274

ΔH_{corr} = 0.303346

$\Delta G°$ = −1183.848684

Cartesian coordinates

C	-2.1624280000	0.4487600000	1.2067980000
C	-3.1403390000	1.2882390000	0.6775820000
C	-3.5058870000	1.2296320000	-0.6657980000
C	-2.8525560000	0.3197290000	-1.4950850000
C	-1.8609550000	-0.5413120000	-1.0241760000
C	-1.5445580000	-0.4641320000	0.3398570000
H	-3.6242070000	2.0054480000	1.3332360000
H	-3.1130850000	0.2793320000	-2.5484920000
C	-1.7826680000	0.5675390000	2.6626210000
H	-1.9671630000	-0.3632410000	3.2048550000
H	-0.7243590000	0.8151750000	2.7797170000
H	-2.3682610000	1.3559950000	3.1353330000
H	-1.5151010000	-1.2455580000	-3.0079390000
H	-0.0977380000	-1.3741910000	-1.9586200000
H	-1.4135220000	-2.5163230000	-1.7694390000
C	-1.1843370000	-1.4745950000	-1.9945570000
C	-4.5941670000	2.1163370000	-1.2066300000
H	-4.4314810000	2.3396350000	-2.2622490000
H	-5.5654330000	1.6205480000	-1.1173480000
H	-4.6489580000	3.0554520000	-0.6537810000
S	-0.3304240000	-1.5639840000	1.1082310000
O	-0.2899470000	-2.8681730000	0.3761870000
N	1.0330460000	-0.5817560000	0.6538180000

C	1.9134400000	-1.1667450000	-0.0376430000
H	1.8337190000	-2.2039050000	-0.3749060000
C	3.1589300000	-0.4432200000	-0.4766270000
O	3.9528110000	-0.9673000000	-1.2157720000
H	5.3222680000	0.9588800000	-0.0668240000
H	4.4415610000	1.6141440000	-1.4576050000
O	3.2640330000	0.7748850000	0.0205100000
C	4.4382480000	1.5232970000	-0.3697830000
C	4.3612390000	2.8667060000	0.3118220000
H	5.2351970000	3.4605790000	0.0371670000
H	3.4643670000	3.4069120000	0.0039150000
H	4.3487030000	2.7504350000	1.3968860000

There are no imaginary frequencies

s-trans-1 conformer of N-sulfinyl imine 1

Charge: 0

Multiplicity: 1

E = −1184.07751

ΔG_{corr} = 0.231867

ΔH_{corr} = 0.302997

$\Delta G°$ = −1183.842615

Cartesian coordinates:

C	-2.2975130000	0.2486640000	-1.1277980000
C	-3.2860240000	-0.7122190000	-0.9061460000
C	-3.4953520000	-1.2675770000	0.3525860000
C	-2.6846620000	-0.8534980000	1.4122610000
C	-1.6845250000	0.1005550000	1.2512830000
C	-1.5121520000	0.6395760000	-0.0357530000
H	-3.9025450000	-1.0313800000	-1.7404910000
H	-2.8349530000	-1.2888420000	2.3960280000
C	-2.1043890000	0.8129290000	-2.5151800000

H	-2.2782290000	1.8915090000	-2.5377710000
H	-1.0932110000	0.6349910000	-2.8903090000
H	-2.8048860000	0.3442540000	-3.2061990000
H	-0.9746170000	-0.2202520000	3.2401920000
H	0.2125040000	0.5727830000	2.1888280000
H	-1.1474010000	1.4914850000	2.7982310000
C	-0.8482140000	0.5061110000	2.4367180000
C	-4.5696450000	-2.2952730000	0.5815800000
H	-4.1479020000	-3.2050680000	1.0157220000
H	-5.3190470000	-1.9161830000	1.2814250000
H	-5.0720070000	-2.5570000000	-0.3501770000
S	-0.2779050000	1.9145510000	-0.3460810000
O	-0.2760750000	2.8675760000	0.8028920000
N	1.1901250000	0.9778140000	-0.1616170000
C	1.2469590000	-0.1745110000	-0.6727210000
H	0.4184210000	-0.6959940000	-1.1616690000
C	2.5243860000	-0.9758450000	-0.6350610000
O	2.5786710000	-2.0710070000	-1.1347060000
H	4.5878660000	-2.0321770000	0.5676050000
H	5.0951750000	-1.3039640000	-0.9662830000
O	3.5156960000	-0.3638570000	-0.0186210000
C	4.7666990000	-1.0868180000	0.0519270000
C	5.7488440000	-0.2128590000	0.7912020000
H	6.7076940000	-0.7299430000	0.8628980000
H	5.9019020000	0.7302170000	0.2638560000
H	5.3940410000	0.0009370000	1.8008090000

There are no imaginary frequencies

s-trans-2 conformer of N-sulfinyl imine 1

Charge: 0

Multiplicity: 1

E = −1184.077748

ΔG_{corr} = 0.230462

ΔH_{corr} = 0.303218

$\Delta G°$ = −1183.844258

Cartesian coordinates:

C	2.24741400	-0.78799400	-0.84433900
C	3.16948000	0.14779200	-1.31455400
C	3.38165500	1.35706300	-0.65876200
C	2.64718200	1.63025200	0.49661900
C	1.71343900	0.73626600	1.01347100
C	1.52996900	-0.47264400	0.31819200
H	3.73451600	-0.07924400	-2.21320100
H	2.80432800	2.57250600	1.01373500
C	2.05715300	-2.08219100	-1.59931600
H	2.27035100	-2.95128600	-0.97262200
H	1.03534900	-2.18735300	-1.97239100
H	2.72972900	-2.11131500	-2.45640700
H	1.10752400	2.15950900	2.48337100
H	-0.09929000	0.89302600	2.18064700
H	1.33393900	0.51672700	3.11793300
C	0.96693900	1.09915400	2.27105800
C	4.37186100	2.36248600	-1.17983700
H	3.85891400	3.27202700	-1.50413800
H	5.08092500	2.64799400	-0.39922900
H	4.93107300	1.96473900	-2.02723300
S	0.38326900	-1.72668000	0.91221600
O	0.44442100	-1.78050100	2.40124100
N	-1.05477100	-0.77005000	0.54499400
C	-1.73009800	-1.21790100	-0.42419700
H	-1.48283100	-2.11110600	-1.00579700
C	-2.98720900	-0.52050500	-0.88312700
O	-3.61433800	-0.93520700	-1.82443300
H	-4.37156900	1.58340100	-1.58713400

H	-5.33140400	0.56444500	-0.49999800
O	-3.29071100	0.54185400	-0.16479300
C	-4.48794000	1.25547300	-0.55239000
C	-4.64497100	2.41662300	0.39736600
H	-5.54201800	2.97960500	0.13231500
H	-4.74746500	2.06507600	1.42539000
H	-3.78519400	3.08606000	0.33711000

There are no imaginary frequencies

s-cis conformer of N-sulfinyl imidoyl fluoride

Charge: 0

Multiplicity: 1

E = −1283.321846

ΔG_{corr} = 0.221763

ΔH_{corr} = 0.296140

$\Delta G°$ = −1283.097055

Cartesian coordinates:

C	-2.2426380000	0.4898420000	1.2051090000
C	-3.2410640000	1.2967790000	0.6618580000
C	-3.6095850000	1.2020790000	-0.6785580000
C	-2.9413600000	0.2878940000	-1.4922640000
C	-1.9333680000	-0.5435740000	-1.0055830000
C	-1.6132650000	-0.4277900000	0.3542420000
H	-3.7374750000	2.0178230000	1.3038270000
H	-3.2043010000	0.2212570000	-2.5438040000
C	-1.8577790000	0.6458770000	2.6558690000
H	-2.0300360000	-0.2742880000	3.2199110000
H	-0.8017510000	0.9071800000	2.7623990000
H	-2.4501450000	1.4379480000	3.1136700000
H	-1.5089410000	-1.2425840000	-2.9771630000
H	-0.1576260000	-1.4478630000	-1.8545110000

H	-1.5339430000	-2.5259510000	-1.7487630000
C	-1.2440730000	-1.4938240000	-1.9497710000
C	-4.7165900000	2.0549350000	-1.2358180000
H	-4.5365400000	2.2963930000	-2.2847280000
H	-5.6704790000	1.5222570000	-1.1772030000
H	-4.8195110000	2.9847270000	-0.6744470000
S	-0.3805400000	-1.5055690000	1.1230570000
O	-0.4600090000	-2.8501540000	0.4876570000
N	0.9542270000	-0.5527240000	0.5747190000
C	1.9645650000	-1.0006760000	-0.0050260000
C	3.1627160000	-0.1436830000	-0.3795620000
O	4.1059400000	-0.6016150000	-0.9614910000
H	5.0020230000	1.6234840000	0.1707900000
H	4.2413270000	2.0011960000	-1.3864020000
O	3.0071290000	1.1020630000	0.0089830000
C	4.0968150000	2.0054670000	-0.3046140000
C	3.7106470000	3.3674920000	0.2140060000
H	4.5127010000	4.0746560000	-0.0057750000
H	2.7963050000	3.7200200000	-0.2660120000
H	3.5572640000	3.3424950000	1.2941190000
F	2.1461760000	-2.2608690000	-0.3842660000

There are no imaginary frequencies

s-trans conformer of N-sulfinyl imidoyl fluoride

Charge: 0

Multiplicity: 1

E = −1283.324329

ΔG_{corr} = 0.223275

ΔH_{corr} = 0.296205

$\Delta G°$= −1283.098026

Cartesian coordinates:

C	-2.4220750000	0.8177370000	-0.5396040000
C	-3.3395300000	-0.0973460000	-1.0601150000
C	-3.4151280000	-1.4048340000	-0.5911640000
C	-2.5522350000	-1.8012560000	0.4335140000
C	-1.6172790000	-0.9355020000	0.9920220000
C	-1.5616090000	0.3694290000	0.4707480000
H	-4.0102450000	0.2278040000	-1.8489680000
H	-2.6109620000	-2.8175330000	0.8124440000
C	-2.4047500000	2.2317800000	-1.0676150000
H	-2.6174250000	2.9547270000	-0.2760250000
H	-1.4399020000	2.4933700000	-1.5060720000
H	-3.1673000000	2.3456180000	-1.8379380000
H	-0.8219090000	-2.5034410000	2.2046560000
H	0.3034320000	-1.1528880000	1.9753180000
H	-1.0579820000	-0.9688070000	3.0641860000
C	-0.7438920000	-1.4192640000	2.1207090000
C	-4.4098910000	-2.3797250000	-1.1591140000
H	-3.9048350000	-3.2765700000	-1.5261960000
H	-5.1193210000	-2.6959670000	-0.3898660000
H	-4.9709860000	-1.9373640000	-1.9827580000
S	-0.4108980000	1.5872510000	1.1372760000
O	-0.4258390000	1.5048040000	2.6226100000
N	1.0619980000	0.7502600000	0.6674210000
C	1.3984840000	0.7508830000	-0.5329990000
C	2.6774140000	0.1401740000	-1.0748850000
O	2.9466230000	0.1768860000	-2.2425940000
H	4.4080330000	-1.8020510000	-1.2488090000
H	5.2513000000	-0.2594880000	-1.0102530000
O	3.3913640000	-0.4020870000	-0.1156250000
C	4.6389470000	-1.0202890000	-0.5230870000
C	5.2916970000	-1.5692750000	0.7200170000
H	6.2362020000	-2.0441450000	0.4478090000
H	5.4981630000	-0.7709840000	1.4346860000

H	4.6534850000	-2.3153330000	1.1962820000
F	0.6636140000	1.2825900000	-1.5136160000

There are no imaginary frequencies

re-precomplex for s-cis conformer of N-sulfinyl imine 1

Charge: 0

Multiplicity: 2

E = −1341.847452

ΔG_{corr} = 0.339999

ΔH_{corr}= 0.429737

$\Delta G°$ = −1341.504425

Cartesian coordinates:

C	-3.08240200	-0.57691100	1.15721800
C	-4.29157900	0.11300000	1.08569700
C	-4.65788300	0.83481300	-0.04828700
C	-3.77624400	0.87319200	-1.12743600
C	-2.54915700	0.21165800	-1.10866500
C	-2.22642600	-0.51536800	0.04746800
H	-4.96353600	0.08019000	1.93778800
H	-4.04567500	1.43980900	-2.01397200
C	-2.73923400	-1.34087400	2.41391100
H	-2.58782000	-2.40392000	2.21166600
H	-1.82681100	-0.96006800	2.87885600
H	-3.55095800	-1.24715000	3.13530900
H	-2.19884600	0.67469100	-3.16645900
H	-0.86256400	1.09960800	-2.09709000
H	-1.12992100	-0.59152900	-2.53404100
C	-1.63048500	0.34571900	-2.29597000
C	-5.98779900	1.53489300	-0.11725500
H	-5.93912300	2.41033100	-0.76679700
H	-6.74923100	0.86187900	-0.52278800

H	-6.31806600	1.85106100	0.87352100
S	-0.70365100	-1.48078700	0.18354000
O	-0.49295200	-2.26525100	-1.07994300
N	0.35719800	-0.11454000	0.22328300
C	1.24835500	-0.09684300	-0.67959400
H	1.30377700	-0.82547300	-1.49195900
C	2.24588800	1.02281600	-0.75765900
O	2.90815200	1.20876800	-1.74908800
H	4.21910600	2.47515400	0.11832600
H	2.94382500	3.54237800	-0.49150400
O	2.28907500	1.76521700	0.33676200
C	3.22070300	2.86778600	0.32121800
C	3.14115600	3.54203200	1.66876500
H	3.83203200	4.38711000	1.69178600
H	2.13245900	3.91377800	1.85720700
H	3.41536300	2.84888300	2.46621200
C	3.50328900	-1.50832700	0.18419900
C	2.63409500	-2.29899300	1.10365100
H	2.14089300	-1.65595200	1.84037300
H	1.87264100	-2.86124800	0.55522800
H	3.23023100	-3.03189100	1.66996600
C	4.46167200	-0.53882100	0.79429500
H	4.86087300	0.15432800	0.04755300
H	3.99973700	0.03436600	1.60333800
H	5.32316100	-1.07185500	1.22739600
C	3.84708600	-2.07263000	-1.15412700
H	3.02007500	-2.64861600	-1.57742500
H	4.12457600	-1.28247200	-1.85896900
H	4.71143200	-2.75175400	-1.07955700

There are no imaginary frequencies

***si*-precomplex for *s-cis* conformer of *N*-sulfinyl imine 1**

Charge: 0

Multiplicity: 2

E = −1341.845677

ΔG_{corr} = 0.341509

ΔH_{corr} = 0.429751

$\Delta G°$ = −1341.50114

Cartesian coordinates:

C	2.33395300	1.31747400	-0.71893600
C	3.24429000	1.75463900	0.23827600
C	3.84458300	0.87257800	1.13558300
C	3.52240500	-0.47793000	1.04959900
C	2.61374700	-0.98039900	0.11251300
C	2.01776700	-0.05256800	-0.75385300
H	3.49356200	2.81090300	0.27769900
H	3.99016700	-1.17697600	1.73601300
C	1.72622200	2.31955200	-1.66893600
H	1.84250500	2.01144200	-2.71149800
H	0.65778000	2.44258800	-1.48082500
H	2.21381500	3.28659200	-1.54681400
H	2.89248800	-2.92158800	0.94745000
H	1.27913000	-2.68257300	0.25254500
H	2.64269000	-2.93827400	-0.80903800
C	2.33945200	-2.46544900	0.12567900
C	4.80611200	1.37827200	2.17656300
H	5.50379100	2.10008600	1.74680900
H	4.26476100	1.88374400	2.98150600
H	5.37827800	0.56063600	2.61669700
S	0.87215800	-0.54003100	-2.07498900
O	0.73472900	-2.02925000	-2.11432100
N	-0.53751700	0.16697600	-1.34373100

C	-1.52845200	-0.61094800	-1.22061600
H	-1.51108400	-1.67174400	-1.48479600
C	-2.86640500	-0.08578700	-0.77589800
O	-3.87393300	-0.73077000	-0.93108300
H	-4.64346900	1.89595000	-0.86918900
H	-4.63882200	1.06829000	0.69592400
O	-2.81383700	1.14147900	-0.28263000
C	-4.07892000	1.75023500	0.05461400
C	-3.77455000	3.05767800	0.74387500
H	-4.70944300	3.55627000	1.00720700
H	-3.20218800	2.88852700	1.65834700
H	-3.20194600	3.71727000	0.08937700
C	-1.84249900	-1.24674600	1.68248400
C	-0.57519200	-0.49733900	1.92205200
H	-0.68916100	0.56457500	1.67614900
H	-0.27765400	-0.55171300	2.98157800
H	0.25087900	-0.89692500	1.33150200
C	-1.78789200	-2.70200200	1.35289800
H	-1.65986800	-3.30805900	2.26471600
H	-2.71530500	-3.03803800	0.87841200
H	-0.95150200	-2.93998100	0.69024300
C	-3.08471600	-0.73464200	2.33372300
H	-3.08982000	0.35766600	2.38684000
H	-3.98452600	-1.07015800	1.80830500
H	-3.16207100	-1.10823000	3.36734600

There are no imaginary frequencies

re-TS for s-cis conformer of N-sulfinyl imine 1

Charge: 0

Multiplicity: 2

E = −1341.84503847

EB3LYP-D3: −1342.351768

EwB97XD: −1341.967714

ΔG_{corr} = 0.345723

ΔH_{corr} = 0.429216

$\Delta G°$ = −1341.496288

Cartesian coordinates:

C	-3.0526770000	-0.7656440000	0.9979450000
C	-4.2429540000	-0.0362660000	0.9545970000
C	-4.4986750000	0.8874680000	-0.0536800000
C	-3.5283870000	1.0871730000	-1.0372640000
C	-2.3208570000	0.3946730000	-1.0391360000
C	-2.1057440000	-0.5346360000	-0.0075360000
H	-4.9865890000	-0.2030280000	1.7275690000
H	-3.7163750000	1.8067900000	-1.8292220000
C	-2.8362690000	-1.7585090000	2.1153970000
H	-2.6962880000	-2.7722580000	1.7326450000
H	-1.9563630000	-1.5066030000	2.7115980000
H	-3.7030230000	-1.7650710000	2.7765090000
H	-1.7941460000	1.1802640000	-2.9574580000
H	-0.5439520000	1.3838210000	-1.7277320000
H	-0.8027970000	-0.2037860000	-2.4608830000
C	-1.3051470000	0.6972610000	-2.1107310000
C	-5.7919500000	1.6555050000	-0.0993650000
H	-5.6028530000	2.7316630000	-0.1170480000
H	-6.3543230000	1.4073060000	-1.0034220000
H	-6.4171140000	1.4294220000	0.7651650000
S	-0.6163530000	-1.5610560000	0.0611310000
O	-0.4154320000	-2.2116260000	-1.2834130000
N	0.4870910000	-0.2724880000	0.2811660000
C	1.4668080000	-0.2257530000	-0.5754920000
H	1.4658300000	-0.8229700000	-1.4891210000
C	2.3015120000	1.0154180000	-0.6458480000
O	2.9949820000	1.2719880000	-1.6018410000

H	4.0342890000	2.7079490000	0.2986520000
H	2.6900010000	3.6124130000	-0.4143980000
O	2.1846730000	1.7908880000	0.4232020000
C	2.9868250000	2.9886130000	0.4311700000
C	2.7508670000	3.6764830000	1.7536540000
H	3.3403190000	4.5945040000	1.7962920000
H	1.6972180000	3.9348540000	1.8729960000
H	3.0508950000	3.0328690000	2.5828720000
C	3.1946490000	-1.5027530000	0.1660110000
C	2.4001080000	-2.6271690000	0.7480830000
H	1.7885530000	-2.2949150000	1.5905080000
H	1.7673600000	-3.1097590000	-0.0009600000
H	3.0965830000	-3.3871560000	1.1295930000
C	3.9028310000	-0.6326880000	1.1574370000
H	4.4359200000	0.1928990000	0.6792410000
H	3.2159060000	-0.2363640000	1.9092300000
H	4.6512770000	-1.2430680000	1.6829880000
C	3.9054270000	-1.7832990000	-1.1193810000
H	3.2775420000	-2.3449270000	-1.8148970000
H	4.2509380000	-0.8677440000	-1.6035130000
H	4.7912030000	-2.3980510000	-0.9022160000

1 imaginary frequency: −280.15 cm^{-1}

si-TS for s-cis conformer of N-sulfinyl imine 1

Charge: 0

Multiplicity: 2

E = −1341.83971014

EB3LYP-D3: −1342.346615

EwB97XD: −1341.963109

ΔG_{corr} = 0.346454

ΔH_{corr} = 0.429384

ΔG° = −1341.490228

Cartesian coordinates:

C	2.2592250000	1.2987110000	-0.7795730000
C	3.2973770000	1.8187320000	-0.0124400000
C	4.0587860000	1.0147330000	0.8341920000
C	3.7657560000	-0.3430560000	0.8910750000
C	2.7337290000	-0.9269700000	0.1472110000
C	1.9773250000	-0.0762380000	-0.6723240000
H	3.5200810000	2.8792570000	-0.0860950000
H	4.3602470000	-0.9842270000	1.5346900000
C	1.4852700000	2.2261730000	-1.6840060000
H	1.4097880000	1.8318540000	-2.7008110000
H	0.4676920000	2.3724510000	-1.3170250000
H	1.9832770000	3.1946130000	-1.7327460000
H	3.2726980000	-2.8115070000	0.9836250000
H	1.5339420000	-2.6386790000	0.7121860000
H	2.5896190000	-2.9370540000	-0.6505110000
C	2.5193910000	-2.4140340000	0.3025280000
C	5.1645250000	1.6103350000	1.6631620000
H	5.8051310000	2.2529850000	1.0550910000
H	4.7514400000	2.2258430000	2.4672990000
H	5.7821010000	0.8330520000	2.1148810000
S	0.6534450000	-0.6723080000	-1.7719670000
O	0.5579600000	-2.1695120000	-1.7012620000
N	-0.6377580000	0.0763370000	-0.9521590000
C	-1.7374320000	-0.6243640000	-0.8176570000
H	-1.8661930000	-1.6070460000	-1.2727160000
C	-3.0146480000	0.1336630000	-0.6255800000
O	-4.0795870000	-0.2883460000	-1.0057760000
H	-4.4695940000	2.3456730000	-0.7342570000
H	-4.7599230000	1.4513010000	0.7653330000
O	-2.8454890000	1.2950590000	-0.0040000000
C	-4.0373450000	2.0717370000	0.2303230000

C	-3.6296460000	3.2829820000	1.0333040000
H	-4.5072710000	3.9014190000	1.2314350000
H	-3.1940380000	2.9833700000	1.9887220000
H	-2.8989390000	3.8822070000	0.4871140000
C	-1.7677080000	-1.5152720000	1.2206260000
C	-0.6998640000	-0.6914510000	1.8684250000
H	-0.9646790000	0.3683290000	1.8802800000
H	-0.5875900000	-1.0256350000	2.9092370000
H	0.2655090000	-0.8048380000	1.3733420000
C	-1.4120760000	-2.9258480000	0.8589220000
H	-1.1830160000	-3.4753820000	1.7834820000
H	-2.2463400000	-3.4346590000	0.3696180000
H	-0.5392200000	-2.9726700000	0.2058340000
C	-3.1457800000	-1.3361180000	1.7849040000
H	-3.3837590000	-0.2883070000	1.9822170000
H	-3.9160370000	-1.7747520000	1.1478590000
H	-3.1801030000	-1.8649320000	2.7482530000

1 imaginary frequency: −359.06 cm^{-1}

si-TS for s-trans-1 conformer of N-sulfinyl imine 1

Charge: 0

Multiplicity: 2

E = −1341.839158

ΔG_{corr} = 0.345109

ΔH_{corr} = 0.429073

$\Delta G°$ = −1341.491021

Cartesian coordinates:

C	-2.55932800	1.07457600	-0.26840300
C	-3.58721700	0.60821600	-1.09183100
C	-4.00642100	-0.71774700	-1.05647900
C	-3.37087700	-1.59720000	-0.17681300

C	-2.34146400	-1.18804400	0.66615100
C	-1.95218900	0.16089500	0.60174600
H	-4.06726200	1.30225100	-1.77450600
H	-3.68559000	-2.63660800	-0.14754100
C	-2.13872000	2.52297500	-0.35934600
H	-2.20379000	3.02599600	0.60819200
H	-1.10784300	2.62262400	-0.71209400
H	-2.78321000	3.05275300	-1.06126700
H	-1.98040400	-3.19865000	1.29432700
H	-0.61123900	-2.10482300	1.58361200
H	-2.01933200	-2.01851100	2.62198500
C	-1.69920800	-2.18785700	1.59238500
C	-5.11874100	-1.20840500	-1.94324100
H	-5.95315300	-1.58100400	-1.34337500
H	-5.48992000	-0.41241100	-2.58975400
H	-4.77552900	-2.03286400	-2.57329700
S	-0.65428800	0.80737200	1.68589800
O	-0.85051700	0.23270700	3.05447000
N	0.68964600	-0.04763000	1.02328100
C	0.96278600	0.18947100	-0.22763200
H	0.29048200	0.71987800	-0.90669500
C	1.91311400	-0.71841400	-0.94871800
O	1.98223300	-0.74619000	-2.15514700
H	3.05162300	-3.04991800	-1.41695300
H	4.25515000	-1.75155600	-1.39316300
O	2.63346500	-1.47926600	-0.13886000
C	3.59014500	-2.35427100	-0.77022800
C	4.33952500	-3.06614000	0.32935100
H	5.07436400	-3.74289300	-0.11119300
H	4.86401200	-2.35054300	0.96540700
H	3.65656400	-3.65094600	0.94796200
C	2.41082200	1.97126900	-0.36633500
C	1.87138000	2.79864000	0.75405300

H	1.94388200	2.27868000	1.71252800
H	2.47685000	3.71310200	0.83106600
H	0.83706200	3.10444000	0.58304500
C	3.75632100	1.36271000	-0.12083200
H	4.09263700	0.74603200	-0.95783300
H	4.48610200	2.17625700	-0.00166600
H	3.77041600	0.77303100	0.79897500
C	2.12726300	2.44880100	-1.75429900
H	2.79168500	3.29736400	-1.97428900
H	2.32391500	1.67598900	-2.50004300
H	1.09900300	2.80292400	-1.86164500

1 imaginary frequency: −252.47 cm^{-1}

re-TS for s-trans-1 conformer of N-sulfinyl imine 1

Charge: 0

Multiplicity: 2

E = −1341.832121

ΔG_{corr} = 0.345970

ΔH_{corr} = 0.429309

$\Delta G°$ = −1341.483124

Cartesian coordinates:

C	-2.06870000	-1.43397700	-0.61981500
C	-3.09179900	-1.81862600	0.24391400
C	-3.90878600	-0.88137200	0.87154300
C	-3.68882400	0.46842900	0.60811700
C	-2.67344700	0.91606200	-0.24051800
C	-1.85225900	-0.05909200	-0.83322700
H	-3.25767600	-2.87703500	0.42038800
H	-4.33053300	1.20817600	1.07745200
C	-1.23744200	-2.50248400	-1.28865200
H	-1.25089400	-2.39899500	-2.37640800

H	-0.19151100	-2.47176400	-0.97143400
H	-1.62974500	-3.48767100	-1.03686800
H	-3.23976100	2.92166100	0.21010700
H	-1.53561400	2.76711400	-0.25375800
H	-2.76831100	2.67671300	-1.48481600
C	-2.54364400	2.40560100	-0.45253600
C	-4.98935800	-1.31505700	1.82414300
H	-4.59550800	-1.37354800	2.84332400
H	-5.81856100	-0.60575700	1.82912000
H	-5.37365900	-2.30171700	1.56043300
S	-0.55009200	0.32586800	-2.03931100
O	-0.64612300	1.77874800	-2.40671700
N	0.92907900	0.13234700	-1.20727100
C	1.00130200	-0.30893400	0.02400100
H	0.12867700	-0.63979900	0.58943800
H	4.73499800	-1.07926700	1.22162800
H	4.49409400	-2.33351700	-0.00478900
O	3.33208400	-0.63842500	-0.23332500
C	4.57628700	-1.25665600	0.15530900
C	1.33662400	1.44315200	1.35058400
C	2.56547300	1.20047200	2.17664000
H	2.48532000	0.30408300	2.79408500
H	3.47205700	1.15525100	1.57015100
C	0.05005300	1.38149700	2.11652300
H	0.01290100	2.24281900	2.79891900
H	-0.82172100	1.43683300	1.46512300
H	-0.01409400	0.47684300	2.72652000
C	1.46751100	2.52107300	0.32152000
H	2.37271000	2.39262700	-0.27578200
H	0.61353200	2.56266700	-0.35644600
H	1.54064500	3.48466800	0.84477000
C	5.66496500	-0.63984500	-0.68839500
H	5.72486600	0.43663100	-0.51639300

H	6.62571500	-1.08685400	-0.42539600
H	5.47918500	-0.81556200	-1.74935600
O	2.25962100	-1.82587000	1.33874100
C	2.25913100	-1.00468800	0.45285500
H	2.67362600	2.05613000	2.85824300

1 imaginary frequency: −335.46 cm^{-1}

si-TS for s-trans-2 conformer of N-sulfinyl imine 1

Charge: 0

Multiplicity: 2

E = −1341.838933

ΔG_{corr} = 0.343717

ΔH_{corr} = 0.429111

$\Delta G°$ = −1341.492188

Cartesian coordinates:

C	2.87250600	-0.63656400	-1.07957500
C	4.05553600	0.09782700	-1.18149300
C	4.49227300	0.92438400	-0.15092300
C	3.71522500	1.01831700	1.00567900
C	2.52621800	0.31103800	1.16195600
C	2.12531400	-0.51860900	0.09951300
H	4.64510600	0.01884700	-2.08948100
H	4.04383400	1.66718300	1.81267000
C	2.44196400	-1.50959000	-2.23452900
H	2.37785100	-2.56088900	-1.94436000
H	1.46269600	-1.21368300	-2.61888000
H	3.16124500	-1.42622500	-3.04946500
H	2.13550600	1.29722700	3.01600800
H	0.68276200	0.63947600	2.23160400
H	1.79750600	-0.44782600	3.03720600
C	1.73880700	0.46188200	2.43761100

C	5.76826500	1.71321500	-0.27048600
H	5.56398100	2.78593200	-0.21880000
H	6.45063300	1.47105500	0.54812500
H	6.27499000	1.50600200	-1.21378000
S	0.63040500	-1.51552700	0.19956100
O	0.53168100	-2.08283500	1.58839800
N	-0.47079400	-0.18054200	0.13847800
C	-1.42184000	-0.35311700	-0.74328500
H	-1.39201700	-1.12718900	-1.51472800
C	-2.25394500	0.83232000	-1.13742800
O	-2.70974100	0.94700900	-2.24887800
H	-2.69133900	3.42634300	-1.27950300
H	-4.17586000	2.56471900	-0.84376900
O	-2.41943200	1.71045600	-0.15943800
C	-3.19724700	2.88380300	-0.47842900
C	-3.30456700	3.70595500	0.78249000
H	-3.88096800	4.61026000	0.57757700
H	-3.81067500	3.14447000	1.57018100
H	-2.31544700	3.99863300	1.13899100
C	-3.07426900	-1.45665800	0.29577500
C	-2.60989900	-2.85928800	0.04803500
H	-1.63568000	-3.05467900	0.50196800
H	-3.32560000	-3.54540500	0.52313000
H	-2.58125900	-3.10444100	-1.01621000
C	-4.31538900	-1.04920200	-0.43583500
H	-4.58205800	-0.00532600	-0.25444000
H	-4.23714300	-1.22227800	-1.51166800
H	-5.14598800	-1.66667400	-0.06488400
C	-2.91734200	-0.95307000	1.69447700
H	-1.93368700	-1.19963000	2.09933700
H	-3.07281600	0.12557400	1.75705600
H	-3.67620600	-1.44213700	2.32181700

1 imaginary frequency: −270.63 cm^{-1}

re-TS for s-trans-2 conformer of N-sulfinyl imine 1

Charge: 0

Multiplicity: 2

E = −1341.838196

ΔG_{corr} = 0.345773

ΔH_{corr} = 0.429152

$\Delta G°$ = −1341.489396

Cartesian coordinates:

C	2.89333300	-0.89501500	0.20780100
C	3.87289100	-0.34112400	-0.61553600
C	3.89702500	1.02030300	-0.91144400
C	2.92148900	1.84113200	-0.34709800
C	1.91319500	1.34198700	0.47664700
C	1.89770500	-0.04280300	0.71549500
H	4.64249000	-0.98942100	-1.02319000
H	2.94523100	2.90853700	-0.54834300
C	2.96841200	-2.36567900	0.54906000
H	3.09422900	-2.51083400	1.62526800
H	2.07399000	-2.91662500	0.25097500
H	3.82580700	-2.81639200	0.04878800
H	1.34986600	3.30106700	1.10781000
H	0.02191900	2.36159300	0.40328000
H	0.56549400	2.00237900	2.03491900
C	0.90411100	2.30694400	1.04598800
C	4.98053800	1.60206400	-1.77835400
H	4.60545800	2.44091300	-2.36722400
H	5.80261200	1.97392400	-1.15941200
H	5.38809200	0.85173100	-2.45754000
S	0.64481100	-0.82708500	1.75201500
O	0.72498700	-0.20683100	3.11802500
N	-0.74908000	-0.10641400	1.02964800

C	-1.61421900	-0.96126800	0.55058600
H	-1.52083900	-2.04359200	0.67185000
C	-3.04047000	-0.51317000	0.40019600
O	-3.96258700	-1.28086000	0.52663900
H	-5.03371100	0.71241100	-0.75415900
H	-5.02943400	1.10784000	0.97197600
O	-3.16690300	0.77989400	0.13774000
C	-4.51658300	1.27883200	0.02351400
C	-4.42357500	2.74706900	-0.31264800
H	-5.42904300	3.16405800	-0.39711500
H	-3.88781900	3.28923000	0.46848100
H	-3.90704300	2.89615400	-1.26289900
C	-1.25682600	-1.12830200	-1.64818100
C	-0.79864100	0.25492200	-1.98465700
H	0.11316700	0.51785800	-1.44445700
H	-0.57409500	0.28973200	-3.06020100
H	-1.56781900	1.00075500	-1.77782800
C	-2.56667600	-1.56405700	-2.22708500
H	-2.40135000	-1.81900500	-3.28362100
H	-2.96042100	-2.45538700	-1.73362600
H	-3.31908200	-0.77279600	-2.19769400
C	-0.18666000	-2.17279400	-1.60366000
H	0.67248000	-1.83147300	-1.02450500
H	-0.54781700	-3.12401200	-1.20643400
H	0.16995300	-2.35225500	-2.62783800

1 imaginary frequency: −296.14 cm^{-1}

R,R-radical adduct from tert-butyl radical and N-sulfinyl imine 1

Charge: 0

Multiplicity: 2

E = −1341.87530755

ΔG_{corr} = 0.350074

ΔH_{corr} = 0.432371

$\Delta G°$ = −1341.522206

Cartesian coordinates:

C	-2.9584870000	-1.1819600000	0.6447990000
C	-4.1476940000	-0.4578970000	0.7313810000
C	-4.3178000000	0.7546330000	0.0661930000
C	-3.2628710000	1.2553540000	-0.6976790000
C	-2.0495730000	0.5806500000	-0.8119170000
C	-1.9277610000	-0.6352890000	-0.1271610000
H	-4.9607700000	-0.8568720000	1.3297540000
H	-3.3840260000	2.2006520000	-1.2184740000
C	-2.8215840000	-2.4969100000	1.3708410000
H	-2.5910320000	-3.3130030000	0.6812900000
H	-2.0256400000	-2.4589000000	2.1182740000
H	-3.7539110000	-2.7407240000	1.8798820000
H	-1.3288190000	1.8828410000	-2.3514210000
H	-0.2708900000	1.7575130000	-0.9429110000
H	-0.3346960000	0.4374660000	-2.1305120000
C	-0.9282780000	1.1913650000	-1.6092720000
C	-5.6228870000	1.4991180000	0.1436820000
H	-5.4593190000	2.5781930000	0.1514100000
H	-6.2442830000	1.2649970000	-0.7258340000
H	-6.1820920000	1.2228780000	1.0386280000
S	-0.4253270000	-1.6302360000	-0.2927490000
O	-0.2304730000	-2.0037580000	-1.7325620000
N	0.6595340000	-0.5459660000	0.2953050000
C	1.9164390000	-0.4485570000	-0.4135060000
H	1.8513370000	-0.8503370000	-1.4310890000
C	2.2869480000	1.0256300000	-0.5663990000
O	2.9117350000	1.4408090000	-1.5090090000
H	3.2819770000	3.2917390000	0.3703750000
H	1.8188500000	3.5800970000	-0.5839250000

O	1.8680080000	1.7836280000	0.4399240000
C	2.1948770000	3.1870000000	0.3630540000
C	1.5514700000	3.8607220000	1.5504970000
H	1.7734630000	4.9294260000	1.5267150000
H	0.4677930000	3.7305540000	1.5271280000
H	1.9342840000	3.4476330000	2.4854470000
C	3.0606870000	-1.2356960000	0.3293420000
C	2.6030200000	-2.6880990000	0.4946740000
H	1.7814990000	-2.7719310000	1.2105720000
H	2.2828250000	-3.1155420000	-0.4608870000
H	3.4336060000	-3.2900480000	0.8719550000
C	3.3492900000	-0.6251300000	1.7017830000
H	3.7936200000	0.3700810000	1.6154630000
H	2.4380070000	-0.5439010000	2.2985580000
H	4.0581340000	-1.2620360000	2.2382790000
C	4.3209210000	-1.2058510000	-0.5389550000
H	4.1321540000	-1.6416420000	-1.5241030000
H	4.6973750000	-0.1911980000	-0.6811690000
H	5.1045820000	-1.7923290000	-0.0515210000

There are no imaginary frequencies

R,S-radical adduct from tert-butyl radical and N-sulfinyl imine 1

Charge: 0

Multiplicity: 2

E = −1341.87038932

ΔG_{corr} = 0.348572

ΔH_{corr} = 0.432648

$\Delta G°$ = −1341.518789

Cartesian coordinates:

C	-2.7116400000	1.1329250000	0.7767620000
C	-3.9289100000	1.3931340000	0.1444600000

C	-4.4945370000	0.4938880000	-0.7546320000
C	-3.8141660000	-0.6932990000	-1.0357990000
C	-2.5944380000	-1.0021390000	-0.4423170000
C	-2.0699330000	-0.0686110000	0.4628800000
H	-4.4448910000	2.3213010000	0.3682600000
H	-4.2430110000	-1.3991080000	-1.7410850000
C	-2.1369050000	2.1361260000	1.7448150000
H	-2.0328190000	1.7123150000	2.7470010000
H	-1.1497360000	2.4776810000	1.4240090000
H	-2.7917010000	3.0047150000	1.8123320000
H	-2.5028970000	-2.8987730000	-1.4309800000
H	-0.9607850000	-2.0352950000	-1.3596670000
H	-1.5862640000	-2.8323590000	0.0881910000
C	-1.8722900000	-2.2698700000	-0.8026120000
C	-5.8124690000	0.7837530000	-1.4197570000
H	-5.7001330000	0.7977400000	-2.5068140000
H	-6.5443840000	0.0093430000	-1.1765940000
H	-6.2122810000	1.7472460000	-1.1021660000
S	-0.5357760000	-0.4040270000	1.3384410000
O	-0.6021150000	-1.7243700000	2.0419110000
N	0.5576240000	-0.2059760000	0.1840630000
C	1.9234330000	-0.5950510000	0.5183410000
H	2.0013740000	-1.0694190000	1.5021960000
C	2.7760370000	0.6701600000	0.6149110000
O	3.4767210000	0.9147920000	1.5645750000
H	3.1773890000	3.2539900000	0.4317570000
H	4.5134560000	2.3875560000	-0.3425480000
O	2.6707180000	1.4545110000	-0.4512930000
C	3.4608650000	2.6617590000	-0.4405120000
C	3.1839510000	3.3904070000	-1.7329360000
H	3.7624970000	4.3159770000	-1.7594260000
H	3.4694160000	2.7799360000	-2.5917430000
H	2.1249250000	3.6409960000	-1.8163780000

C	2.4966170000	-1.6162680000	-0.5177400000
C	2.1625790000	-1.2245620000	-1.9588760000
H	2.5682240000	-0.2449970000	-2.2162460000
H	2.5932340000	-1.9653960000	-2.6385260000
H	1.0831620000	-1.1973060000	-2.1224040000
C	1.8803890000	-2.9812990000	-0.1960710000
H	2.2143800000	-3.7216250000	-0.9282810000
H	2.1821740000	-3.3219030000	0.7983670000
H	0.7895690000	-2.9418870000	-0.2215800000
C	4.0158060000	-1.6949270000	-0.3380250000
H	4.5101850000	-0.7675870000	-0.6441890000
H	4.2849210000	-1.9006700000	0.7020190000
H	4.4141110000	-2.5024090000	-0.9579050000

There are no imaginary frequencies

tert-butyl radical, SMD(CH_2Cl_2)

Charge: 0

Multiplicity: 2

E = −157.7497414

ΔG_{corr} = 0.088609

ΔH_{corr} = 0.124157

$\Delta G°$ = −157.6581046

Cartesian coordinates:

C	0.00000000	0.00000000	0.20135900
C	0.00000000	1.47749200	-0.02050600
H	0.00000000	1.71651700	-1.09715600
H	-0.88746900	1.95096100	0.40873500
H	0.88746900	1.95096100	0.40873500
C	1.27954600	-0.73874600	-0.02050600
H	2.13331600	-0.20691000	0.40873500
H	1.24584700	-1.74405100	0.40873500

H	1.48654700	-0.85825800	-1.09715600
C	-1.27954600	-0.73874600	-0.02050600
H	-1.24584700	-1.74405100	0.40873500
H	-2.13331600	-0.20691000	0.40873500
H	-1.48654700	-0.85825800	-1.09715600

There are no imaginary frequencies

s-cis conformer of N-sulfinyl imine 1, SMD(CH_2Cl_2)

Charge: 0

Multiplicity: 1

E = −1184.085337

ΔG_{corr} = 0.231971

ΔH_{corr} = 0.303125

$\Delta G°$ = −1183.850338

Cartesian coordinates:

C	-2.18498100	0.38927400	1.21742600
C	-3.16217300	1.24269300	0.70987700
C	-3.50486600	1.24168900	-0.64113900
C	-2.82946500	0.37623600	-1.49994000
C	-1.83682700	-0.49601700	-1.05160200
C	-1.54303400	-0.47680900	0.31969700
H	-3.66407200	1.92521600	1.38870900
H	-3.07317700	0.38117300	-2.55809900
C	-1.83388100	0.44385100	2.68435400
H	-2.01484700	-0.51405800	3.17835700
H	-0.78176700	0.70004300	2.83413300
H	-2.44005700	1.20084800	3.18189000
H	-1.44431900	-1.10154400	-3.05941800
H	-0.05273200	-1.28869400	-1.98453200
H	-1.37809100	-2.43218700	-1.88406700
C	-1.13846000	-1.38164600	-2.05100300

C	-4.59271100	2.14337400	-1.15728300
H	-4.44085700	2.37976500	-2.21155500
H	-5.56638000	1.65330000	-1.06208500
H	-4.63385300	3.07456900	-0.58975400
S	-0.32993400	-1.59681100	1.05682900
O	-0.28859700	-2.87851000	0.28317400
N	1.03645500	-0.60347600	0.63993500
C	1.92656000	-1.16894700	-0.05561700
H	1.85693400	-2.19763800	-0.41934400
C	3.17649500	-0.43169500	-0.45760400
O	4.00023200	-0.94944700	-1.16907400
H	5.30856500	0.99808100	0.00857200
H	4.45770300	1.64799800	-1.40539700
O	3.25106000	0.78864800	0.03695300
C	4.42739200	1.55312200	-0.31835100
C	4.31402100	2.89280200	0.36541300
H	5.18704300	3.49878600	0.11532900
H	3.41851400	3.42216400	0.03515500
H	4.27517800	2.77239100	1.44950400

There are no imaginary frequencies

re-TS for s-cis conformer of N-sulfinyl imine 1, SMD(CH_2Cl_2)

Charge: 0

Multiplicity: 2

E = −1341,847432

ΔG_{corr} = 0,345131

ΔH_{corr} = 0.428940

$\Delta G°$ = −1341,499273

Cartesian coordinates:

C	-3.06194000	-0.78134200	0.98469100
C	-4.25165700	-0.05058700	0.94561000

C	-4.50024500	0.89020400	-0.04889000
C	-3.52403100	1.10502100	-1.02372900
C	-2.31682100	0.41176000	-1.02963100
C	-2.10816900	-0.53349900	-0.01070600
H	-5.00039700	-0.22983500	1.71084400
H	-3.70688500	1.83720700	-1.80531800
C	-2.85451900	-1.79406000	2.08598000
H	-2.71115300	-2.80072800	1.68596200
H	-1.97988600	-1.55290400	2.69434300
H	-3.72725300	-1.81216400	2.73891900
H	-1.78349300	1.22065400	-2.93588800
H	-0.53927900	1.41507700	-1.69834700
H	-0.78809800	-0.16441000	-2.45042000
C	-1.29634000	0.73018400	-2.09240400
C	-5.79282100	1.65954300	-0.08981600
H	-5.60249900	2.73556200	-0.10120500
H	-6.35458700	1.41736600	-0.99598400
H	-6.41814500	1.42783900	0.77311400
S	-0.61866500	-1.55822400	0.05442900
O	-0.42151400	-2.20400500	-1.29497000
N	0.48652900	-0.27233300	0.27551000
C	1.46737300	-0.22522800	-0.57977600
H	1.47102400	-0.82367900	-1.49233600
C	2.30352000	1.01447600	-0.64562100
O	3.00362400	1.27030200	-1.59793800
H	4.03484500	2.70071900	0.31274800
H	2.69571600	3.61512500	-0.40156500
O	2.18131800	1.78954100	0.42208300
C	2.98809800	2.98544900	0.44104800
C	2.74745300	3.66471300	1.76707400
H	3.33982600	4.58039200	1.81842200
H	1.69399300	3.92609900	1.88253700
H	3.04194100	3.01423800	2.59305400

C	3.20330800	-1.50408000	0.16740300
C	2.40470100	-2.62571000	0.74839100
H	1.78957100	-2.29112000	1.58729000
H	1.77631400	-3.11088900	-0.00268800
H	3.09963500	-3.38439800	1.13531200
C	3.90965300	-0.63217000	1.15785100
H	4.43821400	0.19633000	0.67961800
H	3.22360900	-0.24107500	1.91327200
H	4.66183800	-1.24130000	1.67964000
C	3.91349700	-1.78528300	-1.11768700
H	3.28105500	-2.33638900	-1.81753900
H	4.27211100	-0.87132400	-1.59522100
H	4.79115200	-2.41152700	-0.90012900

1 imaginary frequency: −255.970 cm^{-1}

si-TS for s-cis conformer of N-sulfinyl imine 1, SMD(CH_2Cl_2)

Charge: 0

Multiplicity: 2

E = −1341.841588

ΔG_{corr} = 0.346389

ΔH_{corr} = 0.429144

$\Delta G°$ = −1341.492172

Cartesian coordinates:

C	2.27473800	1.29602100	-0.77864300
C	3.31516600	1.80894300	-0.00948200
C	4.06795800	0.99976900	0.84019200
C	3.76376900	-0.35578700	0.89858000
C	2.72850600	-0.93235100	0.15342800
C	1.98147900	-0.07661400	-0.66969700
H	3.54679500	2.86748200	-0.08435700

H	4.35165500	-1.00074600	1.54451300
C	1.51158500	2.22847000	-1.68738700
H	1.44110800	1.83557700	-2.70511700
H	0.49246600	2.38149800	-1.32725400
H	2.01522900	3.19418400	-1.73142600
H	3.24344600	-2.81792000	1.00207000
H	1.50952200	-2.63410600	0.70607300
H	2.58352800	-2.94265100	-0.64139100
C	2.50207200	-2.41741500	0.30977700
C	5.17623100	1.58723900	1.67146900
H	5.81661300	2.23327300	1.06678100
H	4.76499900	2.19792000	2.48033100
H	5.79231900	0.80518500	2.11699400
S	0.65523200	-0.66091100	-1.77139800
O	0.55686900	-2.15969600	-1.71297200
N	-0.63507000	0.08243100	-0.94653200
C	-1.73847400	-0.61505100	-0.82348000
H	-1.86953200	-1.59367500	-1.28618900
C	-3.01263300	0.14597900	-0.62990300
O	-4.07953700	-0.27326800	-1.01106000
H	-4.45841100	2.37220800	-0.72684600
H	-4.75567800	1.45967800	0.76255300
O	-2.84093800	1.30475400	-0.00643600
C	-4.03087800	2.08418200	0.23568700
C	-3.61829000	3.28268600	1.05494800
H	-4.49357800	3.90243300	1.25926200
H	-3.18657100	2.96779000	2.00731000
H	-2.88389200	3.88553200	0.51757200
C	-1.78626400	-1.52066700	1.21257500
C	-0.71985100	-0.70470500	1.87179400
H	-0.98705300	0.35428000	1.90003700
H	-0.60895200	-1.05423000	2.90768300
H	0.24624500	-0.81090300	1.37643900

C	-1.43311600	-2.92855200	0.83969600
H	-1.21730900	-3.48782000	1.76155600
H	-2.26421700	-3.42784200	0.33554000
H	-0.55217100	-2.97213600	0.19746900
C	-3.16714300	-1.34043400	1.76851200
H	-3.40398500	-0.29299600	1.96949000
H	-3.93409100	-1.77546000	1.12506200
H	-3.20698800	-1.87359200	2.72928100

1 imaginary frequency: −345.980 cm^{-1}

12. *References*

[1] J. Luo and J. Zhang, *ACS Catal.,* 2016, **6**, 873–877.

[2] Q. Zhu, D. E. Graff and R. R. Knowles, *J. Am. Chem. Soc.,* 2018, **140**, 741–747.

[3] S. Ni, A. F. Garrido-Castro, R. R. Merchant, J. N. de Gruyter, D. C. Schmitt, J. J. Mousseau, G. M. Gallego, S. Yang, M. R. Collins, J. X. Qiao, K.-S. Yeung, D. R. Langley, M. A. Poss, P. M. Scola, T. Qin and P. S. Baran, *Angew. Chem. Int. Ed.,* 2018, **57**, 14560–14565; *Angew. Chem.,* 2018, **130**, 14768–14773.

[4] V. Leandri, P. Liu, A. Sadollahkhani, M. Safdari, L. Kloo and J. M. Gardner, *ChemPhysChem,* 2019, **20**, 618–626.

[5] (*a*) Y. Zhao and D. G. Truhlar, *Theor. Chem. Account.,* 2008, **120**, 215–241; (*b*) S. Grimme, J. Antony, S. Ehrlich and H. Krieg, *J. Chem. Phys.,* 2010, **132**, 154104; (*c*) R. Krishnan, J. S. Binkley, R. Seeger and J. A. Pople, *J. Chem. Phys.,* 1980, **72**, 650–654.

[6] Gaussian 16, Revision C.01, Frisch, M. J., Trucks, G. W., Schlegel, H. B., Scuseria, G. E., Robb, M. A., Cheeseman, J. R., Scalmani, G., Barone, V., Petersson, G. A., Nakatsuji, H., Li, X., Caricato, M., Marenich, A. V., Bloino, J., Janesko, B. G., Gomperts, R., Mennucci, B., Hratchian, H. P., Ortiz, J. V., Izmaylov, A. F., Sonnenberg, J. L., Williams-Young, D., Ding, F., Lipparini, F., Egidi, F., Goings, J., Peng, B., Petrone, A., Henderson, T., Ranasinghe, D., Zakrzewski, V. G., Gao, J., Rega, N., Zheng, G., Liang, W., Hada, M., Ehara, M., Toyota, K., Fukuda, R., Hasegawa, J., Ishida, M., Nakajima, T., Honda, Y., Kitao, O., Nakai, H., Vreven, T., Throssell, K., Montgomery, J. A., Jr., Peralta, J. E., Ogliaro, F., Bearpark, M. J., Heyd, J. J., Brothers, E. N., Kudin, K. N., Staroverov, V. N., Keith, T. A., Kobayashi, R., Normand, J., Raghavachari, K., Rendell, A. P., Burant, J. C., Iyengar, S. S., Tomasi, J., Cossi, M., Millam, J. M., Klene, M., Adamo, C., Cammi, R., Ochterski, J. W., Martin, R. L., Morokuma, K., Farkas, O., Foresman, J. B. & Fox, D. J. Gaussian, Inc., Wallingford CT, 2016.

[7] A. V. Marenich, C. J. Cramer, D. G. Truhlar, *J. Phys. Chem. B,* 2009, **113**, 6378–6396.

[8] E. R. Johnson, S. Keinan, P. Mori-Sánchez, J. Contreras-García, A. J. Cohen, W. Yang, *J. Am. Chem. Soc.,* 2010, **132**, 6498–6506.

[9] Maestro in Vol. Schrödinger, LLC, New York, NY, 2019.1

[10] (*a*) A. D. Bochevarov, E. Harder, T. F. Hughes, J. R. Greenwood, D. A. Braden, D. M. Philipp, D. Rinaldo, M. D. Halls, J. Zhang, R. A. Friesner, *Int. J. Quantum Chem.,* 2013, **113**, 2110–2142; (*b*) Jaguar in Vol. Schrödinger, LLC, New York, NY, 2017.1

[11] A. F. Garrido-Castro, H. Choubane, M. Daaou, M. C. Maestro, J. Alemán, *Chem. Commun.,* 2017, **53**, 7764–7767.

[12] J. A. Fernández-Salas, M. C. Maestro, M. M. Rodríguez-Fernández, J. L. García-Ruano, I. Alonso, *Org. Lett.,* 2013, **15**, 1658–1661.

[13] N. Halland, K. Anker Jørgensen, *J. Chem. Soc., Perkin Trans. 1,* 2001, 1290–1295.

[14] (*a*) A. D. Becke, *J. Chem. Phys.,* 1993, **98**, 5648–5652; (*b*) C. Lee, W. Yang, R. G. Parr, *Phys. Rev. B,* 1988, **37**, 785–789; (*c*) P. J. Stephens, F. J. Devlin, C. F. Chabalowski, M. J. Frisch, *J. Phys. Chem.,* 1994, **98**, 11623–11627; (*d*) S. H. Vosko, L. Wilk, M. Nusair, *Can. J. Phys.,* 1980, **58**, 1200–1211.

[15] J.-D. Chai, M. Head-Gordon, *Phys. Chem. Chem. Phys.,* 2008, **10**, 6615–6620.

[16] S. Grimme, *J. Comput. Chem.,* 2006, **27**, 1787–1799.

[17] W. Huang, J.-L. Ye, W. Zheng, H.-Q. Dong, B.-G. Wei, *J. Org. Chem.,* 2013, **78**, 11229–11237.

[18] J. L. M. Matos, S. Vásquez-Céspedes, J. Gu, T. Oguma, R. A. Shenvi, *J. Am. Chem. Soc.,* 2018, **140**, 16976–16981.
